# Impact of program characteristics on weight loss in adult behavioral weight management interventions: systematic review and component network meta‐analysis

**DOI:** 10.1002/oby.23505

**Published:** 2022-08-02

**Authors:** Jamie Hartmann‐Boyce, José M. Ordóñez‐Mena, Annika Theodoulou, Ailsa R. Butler, Suzanne C. Freeman, Alex J. Sutton, Susan A. Jebb, Paul Aveyard

**Affiliations:** ^1^ Nuffield Department of Primary Care Health Sciences University of Oxford Oxford UK; ^2^ National Institute for Health and Care Research Biomedical Research Centre Oxford University Hospitals National Health Service Foundation Trust Oxford UK; ^3^ Department of Health Sciences University of Leicester Leicester UK

## Abstract

**Objective:**

Behavioral weight management programs (BWMPs) for adults lead to greater weight loss at 12 months than minimal‐intervention control treatments. However, there is considerable heterogeneity in the content of BWMPs and outcomes of treatment. This study assessed the contribution of individual components of BWMPs, using Bayesian component network meta‐analysis.

**Methods:**

Randomized controlled trials of BWMPs in adults were identified (latest search: December 2019) and arms coded for presence or absence of 29 intervention components grouped by type, content, provider, mode of delivery, and intensity.

**Results:**

A total of 169 studies (41 judged at high risk of bias) were included in the main analysis. Six components had effect estimates indicating clinically significant benefit and credible intervals (CrIs) excluding no difference: change in diet (mean difference [MD] = −1.84 kg, 95% CrI: −2.91 to −0.80); offering partial (MD = −2.12 kg, 95% CrI: −3.39 to −0.89) or total meal replacements (MD = −2.63 kg, 95% CrI: −4.58 to −0.73); delivery by a psychologist/counselor (MD = −1.45 kg, 95% CrI: −2.81 to −0.06) or dietitian (MD = −1.31 kg, 95% CrI: −2.40 to −0.24); and home setting (MD = −1.05 kg, 95% CrI: −2.02 to −0.09).

**Conclusions:**

Future program development should consider including these components; other approaches continue to warrant evaluation of effectiveness.

**Study Importance**

**What is already known?**

- Previous systematic reviews have shown that behavioral weight management programs can lead to weight loss in adults with overweight and obesity at 12 months, but with considerable heterogeneity.
- Component network meta‐analysis has been developed to estimate the effects of the components included in complex interventions but has yet to be used in evaluation of behavioral weight management programs.

**What does this study add?**

- Our component network meta‐analysis of 169 trials found that change in diet, offering partial or total meal replacements, delivery by a psychologist/counselor or dietitian, and delivery in a home setting were associated with statistically significant benefit.

**How might these results change the direction of research?**

- Future program development should consider including these components; other approaches continue to warrant evaluation of effectiveness.

## INTRODUCTION

Behavioral weight management programs (BWMPs) aim to achieve weight loss through diet, physical activity, or both and they are mainstays of routine treatment for adults with overweight or obesity. Meta‐analyses of randomized controlled trials have shown that these programs can achieve greater weight loss than minimal‐intensity comparator groups at 12 months and longer [1, 2, 3]. However, there is considerable variation in both the content and outcomes of BWMPs [2].

BWMPs are, by their nature, complex interventions that can include a range of different components, including behavioral elements, varying provider characteristics, and different delivery modes and settings. Analysis of individual trials is not conducive to estimating the specific effects of any of these characteristics, as most trials compare relatively intensive multicomponent BWMPs with minimal‐intervention control groups and cannot identify which elements are enhancing or attenuating observed weight change. This presents challenges to people living with overweight or obesity, health care providers, commissioners, and BWMP providers when choosing services and optimizing service design.

Meta‐regression has been used to try to identify elements of BWMPs associated with effectiveness. In a previous review of a subset of BWMPs, we found that calorie counting, contact with a dietitian, and use of behavior‐change techniques comparing participants' behavior with others' were associated with greater weight loss [2]. However, a key limitation of meta‐regression is that it identifies observational differences between trials, not within randomized arms. Component network meta‐analysis (cNMA) has been developed to estimate the effects of the components included in complex interventions, drawing on both direct and indirect comparisons and allowing a wider range of intervention characteristics to be evaluated [4, 5]. In additive models, estimates from individual components can be combined to estimate the predicted effectiveness of a program containing those components. Interaction models can test whether the effectiveness of one component depends on the presence of one or more other components in the intervention. To the best of our knowledge, cNMA has not yet been used to disaggregate the effective components of BWMPs.

Here, we analyze the difference in body weight between intervention and control groups at 1 year (±2 months) from baseline, using cNMA.

## METHODS

This is a further analysis of a study‐level data set originally compiled for a previous review investigating weight regain and associated health impacts following completion of BWMPs. Full methods for search, screening, data extraction, and risk of bias assessment can be found in the preregistered protocol (PROSPERO ID CRD42018105744) and study report [6].

### Screening and data extraction

Randomized trials of BWMPs in adults with overweight or obesity reporting outcomes at ≥12 months, including at and after program end, were identified through searching trial registries, searching 11 electronic databases, and conducting forward citation searching (from database inception; latest search: December 2019). Studies also had to report a measure of weight change between 10 and 14 months after study start and had to be deemed “jointly randomizable,” meaning that, in principle, it would be reasonable for all of the intervention components included in the network to be delivered in a trial. This meant that we excluded interventions to which some of the participants in the network could not be reasonably assigned in a hypothetical scenario. This includes those interventions in which the program is tailored to specific population characteristics: for example, for patients with a preexisting medical condition. Studies were screened by two independent reviewers with discrepancies resolved by discussion. One reviewer extracted the data that were checked by a second reviewer. Risk of bias was assessed with Cochrane's risk of bias tool (version 1) [7].

### Measures of treatment effect

Mean weight change, SD, and number allocated (*n*) were extracted or estimated for each arm. If weight change was not available, we estimated it from weight at baseline and follow‐up. If the SD for weight change was not reported, it was estimated using SD at baseline and follow‐up [8]. If the SD at follow‐up was missing, it was assumed to be equal to the SD at baseline. If neither weight change nor weight at baseline and follow‐up was available, we used the reported percentage weight change. When *n* for weight change was not available, *n* at baseline was used. When SD weight change was not available, we estimated it using the SE obtained from the reported 95% CI. If medians were reported, we assumed they were means. If interquartile range (IQR) or minimum and maximum were reported, we estimated SD using methods that have been described elsewhere [9].

### Components

For every intervention, we considered whether any of the following 29 nonmutually exclusive components (classified into six groups) were present: intervention type (control, diet, or exercise); intervention content (partial or full meal replacement, financial incentives, or intermittent fasting); intervention provider (nurse, physician, psychologist/counselor, dietitian, nutritionist, exercise specialist, health trainer, or other provider); mode of delivery (individual, group, face‐to‐face, telephone, internet, app, print, video, SMS, or other/unclear); and intervention setting (health care, community, workplace, or home). We also considered two measures of intensity: length of contact in months (up to 12 months) and total contact time in minutes (up to 12 months). All components were compared against control. Prespecified components used in only one of the included studies were merged (nurse specialist and nurse general) and those used in a small number of studies for which treatment effects failed to converge were excluded (inpatient setting).

Two‐arm trials were excluded if both arms included the same components (as they contribute no comparative effectiveness information on components). If a trial had two arms that differed only in intensity, this trial was excluded for the analysis excluding intensity components but was included in the analysis including intensity components. For multiarm trials (i.e., including *n* arms and *n* > 2), if *n* − 1 arms (or fewer) shared the same components, they were combined for analysis [10].

### Synthesis

An analysis plan was registered in advance (https://osf.io/qaxcy/).

The cNMA models used were similar to those used by Freeman and colleagues [5]. These models estimate the impact of each component on weight change. Bayesian analyses were run using WinBUGS version 1.4.3 and R (version 4.0.0) using the R2WinBUGS package [11]. For each model, three different simulation chains with different initial values were run, each with at least 30,000 iterations, discarding the first 15,000 iterations, and with a thinning interval of three to compute summary estimates. We used trace plots to evaluate convergence for each chain for all component effects and all tested interactions between components. Minimally informative prior distributions for the trials' weight change in the control arms, component and interaction effects, and between‐trial (heterogeneity) SD (measured on the log‐odds scale) were chosen as in Freeman et al. [5]. We assumed a common between‐trial SD and we report results as odds ratios with 95% credible intervals (CrIs). All models were compared using the Deviance Information Criteria (DIC). We considered reductions in DIC greater than three to indicate a better fitting model. The main results model includes all components (excluding intensity variables) and assumes additive component effects (i.e., no interaction between component effects). For continuous variables, we assumed a linear (constant) increase in effect size per unit of continuous variable component.

We added multiplicative interaction terms to test for prespecified interactions between diet and exercise and for provider and intervention congruence, for example, dietitian in programs that involve diet and exercise specialist in programs that involve physical activity. We also ran an analysis including intensity variables, which had a different sample size as the main analysis, as it required complete data on intensity variables across arms for trials to be included.

Using component network meta‐regression (CNMR), we elaborated the main model to include study‐level covariates to adjust the component estimates for between‐study differences in baseline BMI, selection based on disease status (e.g., whether the study was restricted to people with a specific medical condition), recruitment method (self‐initiated vs. prompted), and weight change in control group. We assumed a common interaction between covariates and each component effect (i.e., the same for each component), with flat priors. The impact of each covariate on the model was considered one at a time. The sample size for these analyses was also dependent on complete data for the relevant covariate. We conducted a sensitivity analysis excluding studies that reported only percentage weight change and excluding studies judged to be at high risk of bias.

## RESULTS

Our initial searches retrieved 17,085 references, 4482 of which were screened for full text. The most common reason for exclusion after full text review was follow‐up less than 12 months. Another 246 relevant references were identified by forward citation searching and screening of trial websites. A total of 879 references representing 330 studies met the inclusion criteria for the initial review, with 249 of these studies providing extractable data (Supporting Information Figure S1). Of these, 169 studies with 382 arms were included in our main cNMA excluding intensity components (Supporting Information Figure S2) and 48 studies with 105 arms were included in the cNMA involving intensity components (Supporting Information Figure S3).

The majority of studies had two arms (*n* = 136, 80.5%), whereas the rest had three (*n* = 27, 16.0%), four (*n* = 4, 2.37%), six (*n* = 1, 0.59%), and seven arms (*n* = 1, 0.59%). In the main analysis including 382 arms, there were 322 unique combinations of the 29 components. A total of 25 arms had none of the components and these were labeled as control. The most common intervention type, content, provider, method of delivery, and setting were diet, partial meal replacement, dietitian, face‐to‐face, and the community, respectively. The average number of components per arm was seven, ranging from one to sixteen components (Supporting Information Figure S4). Supporting Information Figure S5 displays frequencies of combinations of components.

Characteristics of the included studies, including overall risk of bias judgments, are summarized in Table 1. Study characteristics, detailed risk of bias judgments, and primary references can be found in Supporting Information Tables S1 to S5. Overall, 41 studies were at high risk of bias, 93 were at unclear risk, and 35 at low risk. Later in this article, we describe the characteristics of the meal replacement interventions in more detail, as these were a central (and varied) element of our findings.

**TABLE 1:** Summary information on characteristics of studies contributing to main analysis

	*n*/median (%/IQR)
*N* total trials	169
Sample size, median (IQR)	126 (65‐251)
Age (y), median (IQR)	49 (45‐56)
% Female, median (IQR)	73 (51‐100)
Mean BMI (kg/m^2^), median (IQR)	34 (31‐36)
Number of arms contributing to analysis, *n* studies (%)	
2	136 (80)
3	27 (16)
4	4 (2)
6	1 (1)
7	1 (1)
Geographical region, *n* (%)	
North America	94 (56)
South America	2 (1)
Europe	52 (31)
Asia	7 (4)
Australia and New Zealand	0 (0)
Africa	12 (7)
Mixed (Australia and Europe)	1 (1)
Missing	1 (1)
Selection based on disease status, *n* (%)	
No	98 (58)
Yes	70 (41)
Missing	1 (1)
Recruitment method, *n* (%)	
Self‐initiated	67 (40)
Prompted	72 (43)
Missing	30 (18)
Random sequence generation (selection bias), *n* (%)	
Low	101 (60)
Unclear	67 (40)
High	1 (1)
Allocation concealment (selection bias), *n* (%)	
Low	63 (37)
Unclear	106 (63)
High	0 (0)
Blinding of outcome (detection bias), *n* (%)	
Low	147 (87)
Unclear	14 (8)
High	8 (5)
Incomplete outcome data (attrition bias), *n* (%)	169 (100)
Low	141 (83)
Unclear	7 (4)
High	21 (12)
Other bias, *n* (%)	
Low	2 (1)
Unclear	13 (8)
High	17 (10)

We coded interventions as “full meal replacements” if all meals were replaced. In studies reporting it, these programs provided 600 to 1,000 kcal/d or they were described as “very low‐calorie diets,” implying 800 kcal/d or fewer. Diets were most commonly provided via liquid forms (e.g., powders, shakes, soups), but a minority provided what they described as pre‐portioned packaged meals. Full meal replacements were most commonly provided for 8 weeks, but this ranged from 5 days to 17 weeks.

If only one or two meals were replaced in a day, we coded this as “partial meal replacements.” When reported, calorie intake ranged from 800 to 1800 kcal/d, often depending on initial weight. Meals most commonly comprised shakes, formulas, or bars, with instructions to replace two meals or one meal and a snack per day. Again, a minority of programs provided pre‐portioned packaged meals. Program length ranged from 8 weeks to 2 years, with most involving a step change in intensity (e.g., from two to one product per day) at 2 to 6 months.

### Main analysis (excluding interactions and intensity components)

**FIGURE 1:**
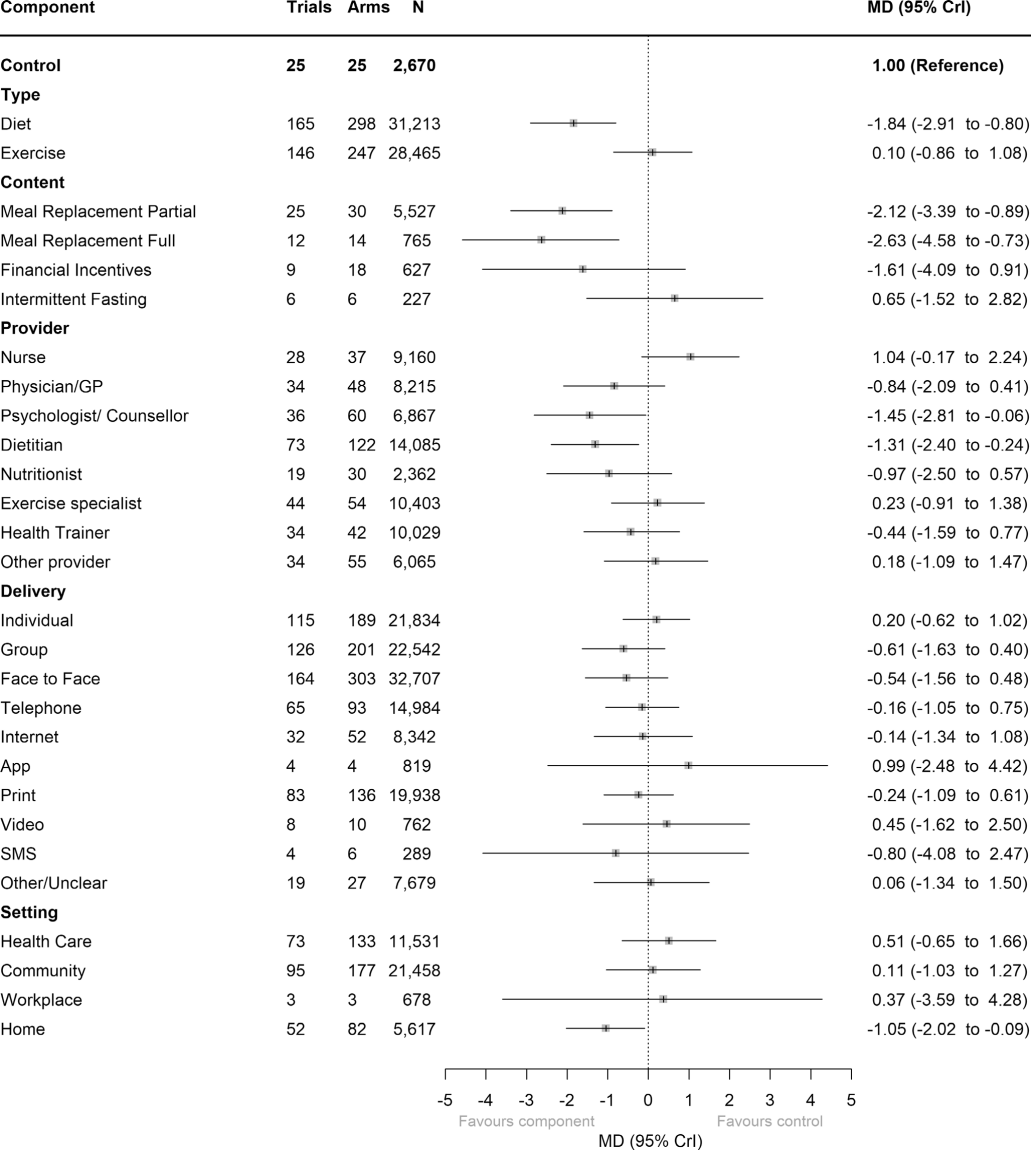
Component effect estimates from main model

Effect estimates for all components from the main analysis can be seen in Figure 1. Six components had effect estimates indicating clinically significant benefit and CrIs excluding no difference: diet (mean difference [MD] = −1.84 kg, 95% CrI: −2.91 to −0.80); partial meal replacements (MD = −2.12 kg, 95% CrI: −3.39 to −0.89); total (full) meal replacements (MD = −2.63 kg, 95% CrI: −4.58 to −0.73); intervention provision by psychologist/counselor (MD = −1.45 kg, 95% CrI: −2.81 to −0.06) or dietitian (MD = −1.31 kg, 95% CrI: −2.40 to −0.24); and home setting (MD = −1.05 kg, 95% CrI: −2.02 to −0.09). For all other component effect estimates, CrIs were wide and they included the possibility of no weight loss difference between components and control. For the delivery components, weight loss was greatest for group and SMS. When excluding studies at high risk of bias, provision by psychologist/counselor and by dietitian was still associated with greater weight loss, but 95% CrIs included no difference (MD = −0.36 kg, 95% CrI: −1.97 to 1.32 and MD = −1.50 kg, 95% CrI: −2.99 to 0.01, respectively); other components statistically significantly associated with benefit in the main model remained so after excluding studies at high risk (Supporting Information Figure S6). The few studies highlighted as outliers had low DIC contributions and, therefore, they were judged unlikely to affect the main model estimates. Component effect estimates were not sensitive to exclusion of studies reporting only percentage weight change (Supporting Information Figure S7).

### Analysis including intensity components

**FIGURE 2:**
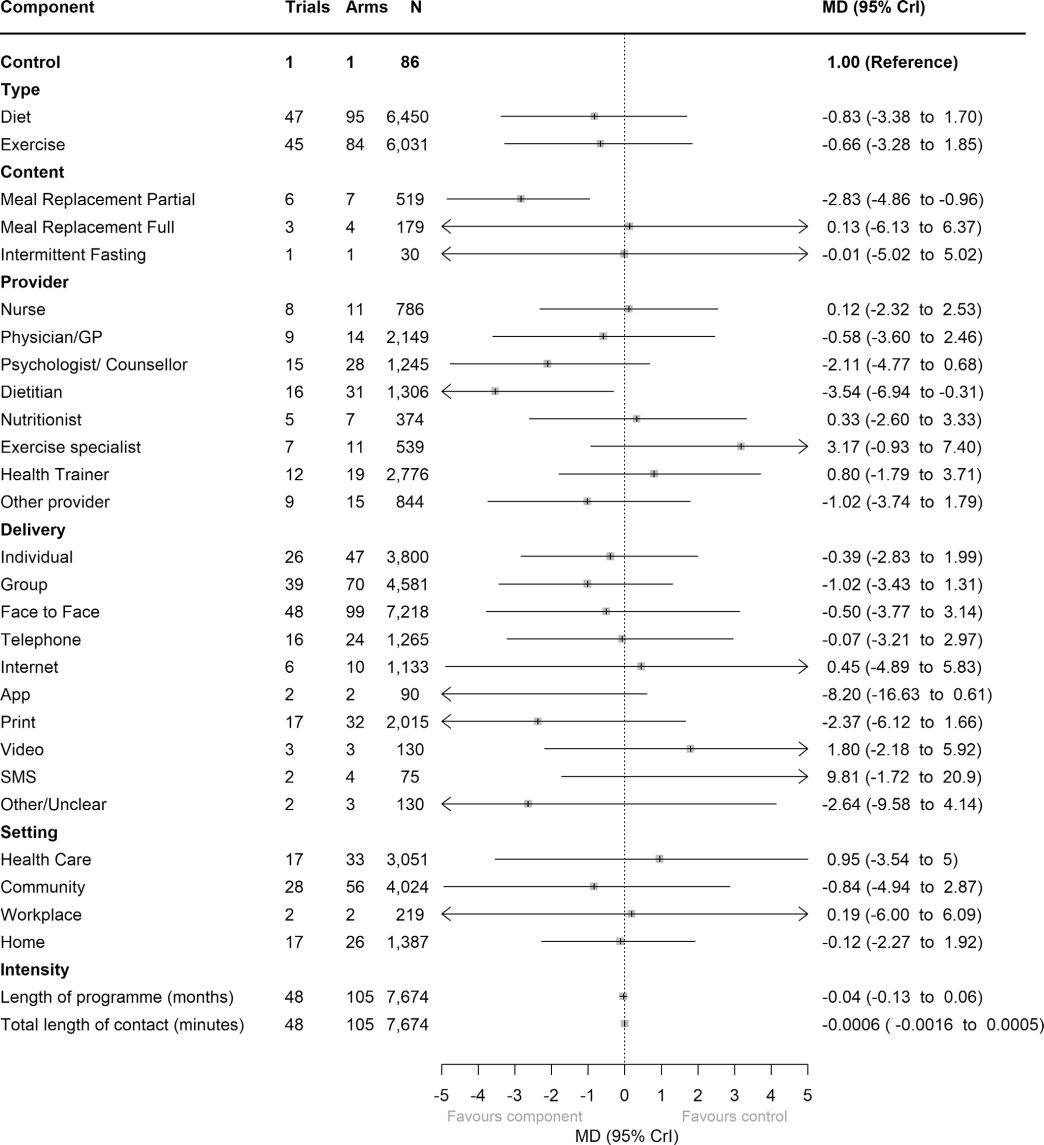
Component effect estimates in model including intensity covariates

The majority of studies did not provide sufficient detail on program length or number and length of sessions to be included in this model. Data were available for 48 trials (105 arms) with complete information on intensity components across all arms (Figure 2). Of those components associated with statistically significant benefit in the main model, both partial meal replacements and dietitian as provider remained associated with greater weight loss, with 95% CrIs excluding no difference. All of the other components that were associated with benefit in the main model still had point estimates indicating benefit, with the exception of full meal replacements, in which the point estimate was just above no difference (0.13 kg), but CrIs were very wide (−6.13 to 6.37 kg). Effect estimates for both intensity variables favored more intensive interventions, but CrIs included no difference and weight gain for both. Every additional month of a program (up to 12 months) was associated with 0.04 kg greater weight loss (95% CrI: −0.06 to 0.13 kg greater weight loss). Every additional hour of contact time over 12 months was also associated with 0.036 kg greater weight loss (95% CrI: −0.03 to 0.10 kg greater weight loss).

### Interactions

None of the interactions tested had CrIs excluding no difference (Supporting Information Table S6), providing no strong evidence that diet and exercise had synergistic or antagonistic effects. There was also no evidence that congruence between dietary advice and dietitian/nutritionist delivery or physical activity specialist and physical activity provision improved outcomes; although, in the case of exercise being provided by an exercise specialist, 95% CrIs only narrowly included the null (−2.81 kg, 95% CrI: −5.75 to 0.13). Including these predefined interactions between components did not result in any meaningful changes to main component effect estimates compared with the results of any of the other models (Supporting Information Table S6) and did increase the DIC from 1,473 to 1,475, indicating no evidence of model fit improvement.

### CNMR

Adjusting for our covariates in CNMR did not meaningfully influence component effect estimates; in some cases, including covariates meant CrIs included no difference where they previously had not, but the direction of effect remained consistent and CrIs overlapped with the main model in all instances for those six components for which clear benefit had been detected in the main model (Supporting Information Table S6). In all CNMR models, provision by dietitian remained associated with benefit, with 95% CrIs excluding no difference. Point estimates were close to no difference for all covariate effects, and all had CrIs including no difference (Supporting Information Table S6). Adding control group weight change into the model reduced DIC from 1,473 to 1,469, indicating a slight improvement in model fit. For the other covariates, the study set was smaller (as not all studies reported all covariates); therefore, DIC values were expected to be lower and cannot be compared across models.

## DISCUSSION

This cNMA included data from 169 randomized controlled trials of BWMPs in adults living with overweight and obesity. Of the components evaluated, changes to diet, partial meal replacements, and full meal replacements were all independently associated with greater weight loss at 1 year (±2 months), with CrIs excluding no difference. CrIs overlapped between partial and full meal replacements, although the point estimate showed greater weight loss for full meal replacement. Program provision by a psychologist/counselor or dietitian was also associated with greater weight loss, as was home‐based content. We did not find evidence of interactions between prespecified components, impact of our prespecified covariates, or of increasing program intensity; point estimates favored more contact and longer programs, but CrIs included no difference.

BWMPs are complex interventions, comprising several co‐occurring components, even for the simplest examples [12]. Understanding which components are influencing outcomes would require a practically infinite set of head‐to‐head trials, which would never be funded. In theory, cNMA offers a practical way to identify key components by comparing trials that differed on key components. It aims to isolate the effects of individual elements of programs while holding constant any other elements of the intervention that differ between intervention and control programs. The ability of this approach to achieve this goal and produce valid results thereby depends on several factors. First, trial reports need to fully report the components in the intervention as well as outcomes; we were unable to include 25% of trials in the initial review because we could not obtain data on weight in sufficient detail to allow extraction. Second, the extraction of intervention characteristics has to meaningfully capture not just variation in content but all meaningful contributors to variation in outcome. Arguably, both are incomplete in this instance. Although it would be possible to have created a more fine‐grained characterization of the components of a network, it would create further problems in coding the content of programs in our studies, risk model nonconvergence and colinearity, and limit the opportunity to provide usefully precise effect estimates. As it stands, however, there are likely unclassified differences between studies that add to the imprecision and cloud our estimates of treatment effects, including specifics of diet and exercise prescriptions and different behavioral techniques employed, which were not reported in sufficient detail in our included studies to be able to be included as components in our model. Accordingly, cNMA may best be viewed as an exploratory technique for identifying promising intervention components for testing in trials, for example, in multiphase optimization studies. Additionally, there is no way to test for publication bias in cNMA, and we cannot rule it out as a possibility, but it seems unlikely to be related to the presence/absence of specific components.

There are some further limitations to consider relating to our specific analyses. A total of 41 of the included studies were considered at high risk of bias; sensitivity analysis removing these did not meaningfully change effect estimates but did widen CrIs in some instances. As this was a secondary analysis of data extracted for a previous systematic review [6], we only included studies that measured weight at and after program end. This means that some studies that might have been eligible for the analysis presented here will not have been included (e.g., studies that only followed up participants at 12 months would have been excluded from the parent review). However, although this will have resulted in a smaller sample size, there is no reason to think such studies would differ in terms of component effects from those presented here; therefore, this is unlikely to have systematically biased our results. We did not consider effects over longer‐term follow‐up. Our base reviews showed that initial weight loss was the single most important factor contributing to long term benefit; therefore, our focus here was on the components delivered within program, which could conceivably maximize early weight loss [6]. Finally, analyses for less common components lacked power, particularly in the subset of studies reporting on intensity. Absence of evidence of an association for these should not be confused with evidence of absence.

The choice of classification system can have important implications for analyses and findings [13], and other classification systems may have produced other insights. The 93‐item Behavior Change Taxonomy concentrates on the methods used to convey the behavioral program [14]. The 117‐item Oxford Food and Activity Behaviours taxonomy concentrates on the behavioral techniques participants use when aiming to reduce their weight [15]. However, both would require considerably more detail on the description of interventions than are usually provided in studies. Future updates of this analysis would be greatly assisted by more detailed reporting of intervention components, preferably in a standardized format, such as that detailed in the Template for Intervention Reporting and Descrioption (TIDieR) checklist [16].

Our findings that both full and partial meal replacements are associated with greater weight loss are consistent with pairwise meta‐analyses [1, 17]. Programs employing meal replacements typically involve a greater energy deficit than those that do not; therefore, this finding could be a consequence of the lower energy intake in these studies rather than the restricted range of products replacing usual food consumption. In our original analysis of this data set, in which we investigated weight regain after program end, interventions involving meal replacements were associated with faster weight regain, but this association was no longer significant after adjusting for weight loss during the program. In that analysis, greater during‐program weight loss was associated with faster weight regain, but greater initial weight loss was still associated with lower weight for at least 5 years after program end, because of the relatively small differences in the rate of regain observed [6].

We found no evidence that programs that promoted exercise increased weight loss at 12 months. A previous meta‐analysis of a smaller number of trials that randomized participants to diet or diet plus exercise did find evidence of a modest benefit of exercise; our 95% CrIs are consistent with this benefit [18]. Like this cNMA, a previous meta‐regression of BWMPs also found that contact with a dietitian was associated with greater weight loss at 12 months (−1.5 kg, 95% CI: −2.9 to −0.2) [2].

One of the benefits of cNMA over traditional meta‐regression is the ability to include a greater number of components in analyses. We therefore cannot directly compare our results to a meta‐regression of the same studies, as this would require us to analyze the data based on a smaller list of components. Including additional components revealed that delivery by a psychologist or counselor was associated with improved outcomes; we are not aware of other reviews that have specifically examined this feature. There was no evidence of a difference in the type of training (e.g., dietitian vs. psychologist). It is possible that the presence of these professionals in a program signifies high intensity, well‐resourced, and comprehensive programs that were not captured by other aspects of our classification, and it is this, rather than the particular skills of the professionals involved, that improves weight loss. Delivery in a home setting, but not a health care, workplace, or community setting was more effective, but we are not aware of other studies that have examined this particular feature and therefore cannot compare our findings with other data.

These coefficients should be interpreted as additive. Including each of these particular elements (meal replacements, input from a dietitian and/or a psychologist/counselor, and home‐based elements) would add to the overall expected benefit over no intervention. Future trials may wish to explore the benefit of adding home‐based elements to in‐person delivery. Further studies may also consider evaluating program‐intensity variables as categorical components as opposed to continuous, as there may be a plateau effect after a certain amount of contact. The impact of professional input is also worthy of further investigation, particularly given that such input would likely increase resource requirements, and cost‐effectiveness would need to be analyzed. We found no evidence of interactions between components but cannot rule them out. This is the second largest cNMA data set, to our knowledge, in which few or no important interactions have been detected between components.

## CONCLUSION

Of the 29 components investigated, this cNMA, with data from 169 randomized controlled trials of BWMPs, found evidence that changes to diet and provision of partial or full meal replacements were independently associated with greater weight loss at 1 year, providing further support for their use in BWMPs. Professional support from a dietitian or psychologist and delivery at home also appear to offer some benefit, although the former in particular may have resource implications and therefore it requires cost‐effectiveness analyses. Future program development should consider including these components; other approaches continue to warrant careful evaluation of effectiveness. Improved standards of reporting of the behavioral, diet, and exercise content of weight loss programs would allow more fine‐grained definition of components in future cNMAs and enhance the development of effective interventions.

## FUNDING INFORMATION

This research was funded by the British Heart Foundation (BHF), PG/17/68/33247, and National Institute for Health and Care Research (NIHR) Oxford Biomedical Research Centre (BRC) Obesity, Diet, and Lifestyle Theme. Paul Aveyard, Jose M. Ordonez‐Mena, and Susan A. Jebb are part funded by NIHR Oxford BRC. Paul Aveyard and Susan A. Jebb are also funded by NIHR Oxford and Thames Valley Applied Research Collaboration. Suzanne C. Freeman and Alex J. Sutton are funded by the NIHR Complex Reviews Support Unit (project number 14/178/29) and supported by the NIHR Applied Research Collaboration East Midlands. The views expressed are those of the authors and not necessarily those of the BHF, National Health Service, NIHR, or the Department of Health and Social Care. The funders were not involved in study design, conduct, reporting, or decision to submit for publication.

## CONFLICT OF INTEREST

Paul Aveyard and Susan A. Jebb were investigators on a trial of a low‐energy total diet replacement program funded by Cambridge Weight Plan, and Paul Aveyard spoke at a seminar at a Royal College of General Practitioners' conference funded by Novo Nordisk A/S; neither of these led to personal payments. All other authors declared no conflict of interest.

## Supporting information


**Appendix S1** Supporting Information
