# Supplementary material for: Impact of program characteristics on weight loss in adult behavioral weight management interventions: systematic review and component network meta‐analysis
Source: Obesity (Silver Spring). 2022 Aug 2;30(9):1778–86. doi: 10.1002/oby.23505 (PMC9546221; doi:10.1002/oby.23505)
Supplement: Supplementary file 1 — Appendix S1 Supporting Information [file OBY-30-1778-s001.docx]

**Supplemental material**

The impact of behavioural, motivational, and delivery characteristics of behavioural weight management programmes in adults with overweight and obesity: systematic review and component network meta-analysis

**Jamie Hartmann-Boyce^1^, Jose Ordonez-Mena^1^, Annika Theodoulou^1^, Ailsa Butler^1^, Suzanne C Freeman^2^, Alex Sutton^2^, Nicola Cooper^2^, Susan A. Jebb^1^*, Paul Aveyard^1^***

*joint senior authors
^1^Nuffield Department of Primary Care Health Sciences, University of Oxford, Oxford, UK
^2^Department of Health Sciences, University of Leicester, Leicester, UK

Contents

[**Supplemental Figure S1**. PRISMA diagram of study flow for main review 2](#_Toc104560358)

[**Supplemental Figure S2**. PRISMA diagram of study flow for CNMA excluding intensity 3](#_Toc104560359)

[**Supplemental Figure S3**. PRISMA diagram of study flow for CNMA including intensity variables 4](#_Toc104560360)

[**Supplemental Figure S4.** Number of components per intervention arm 5](#_Toc104560361)

[**Supplemental Figure S5**. Frequencies of the 25 most common combinations of components 6](#_Toc104560362)

[**Supplemental Figure S6**. Effect estimates from main model, excluding studies at high risk of bias 7](#_Toc104560363)

[**Supplemental Figure S7.** Effect estimates from main model, excluding studies reporting only percentage weight change 8](#_Toc104560364)

[**Supplemental Table S1**. Characteristics of included studies 9](#_Toc104560365)

[**Supplemental Table S2.** Risk of bias judgements across included studies 25](#_Toc104560366)

[**Supplemental Table S3.** Included studies reference list 98](#_Toc104560367)

[**Supplemental Table S4.** Baseline demographics 107](#_Toc104560368)

[**Supplemental Table S5.** Intervention characteristics 119](#_Toc104560369)

[**Supplemental Table S6.** Component effect estimates (MD in kg, 95% CrI) across models 185](#_Toc104560370)

## **Supplemental Figure S1**. PRISMA diagram of study flow for main review


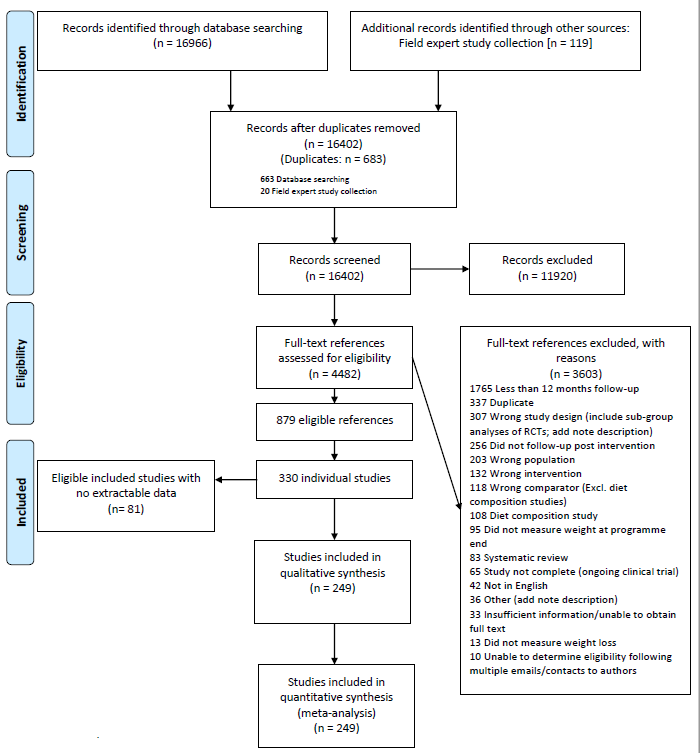


## **Supplemental Figure S2**. PRISMA diagram of study flow for CNMA excluding intensity


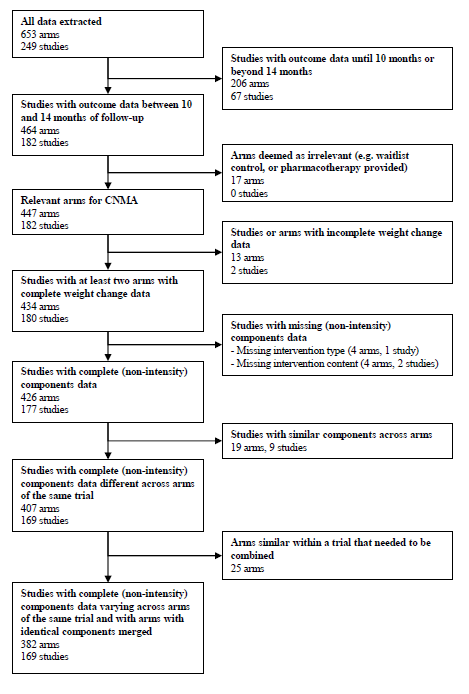


## **Supplemental Figure S3**. PRISMA diagram of study flow for CNMA including intensity variables


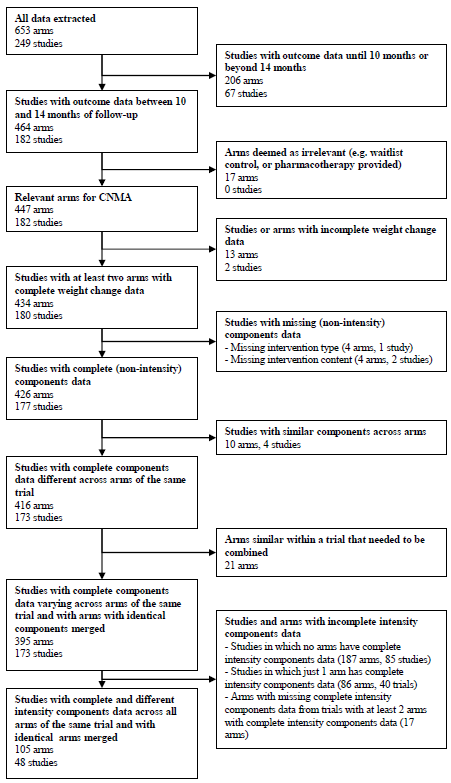


## **Supplemental Figure S4.** Number of components per intervention arm


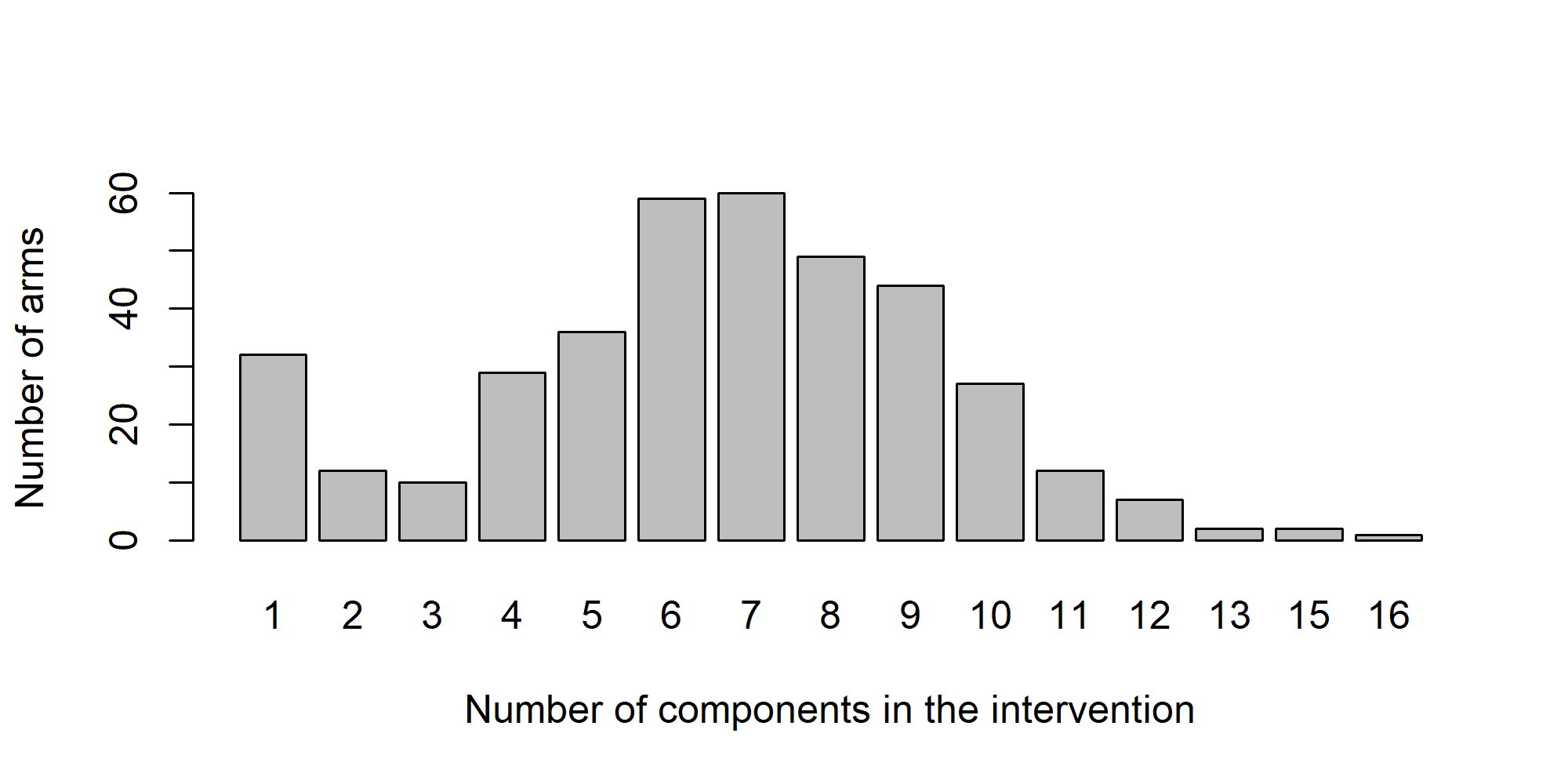


##
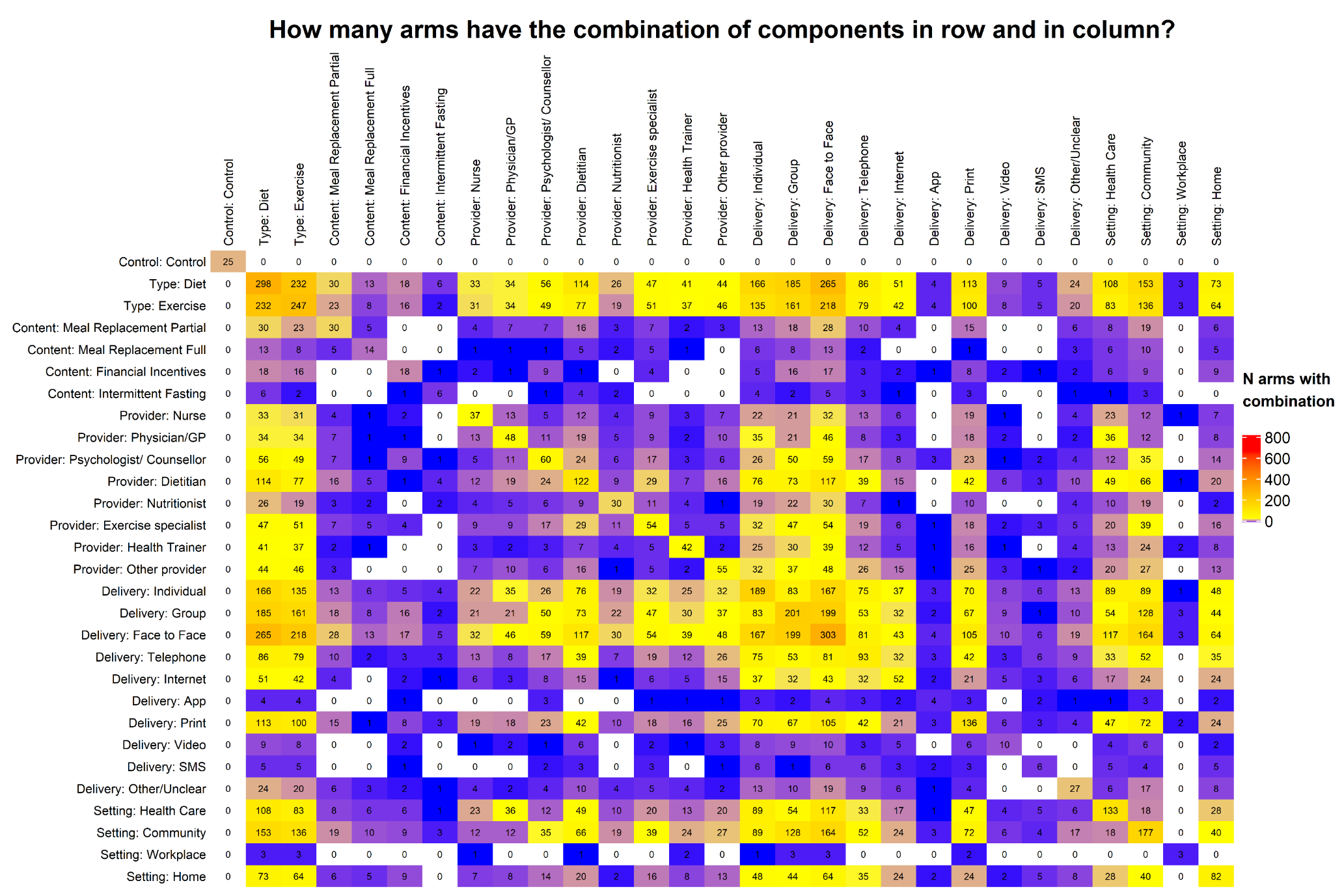
**Supplemental Figure S5**. Frequencies of the 25 most common combinations of components

## **Supplemental Figure S6**. Effect estimates from main model, excluding studies at high risk of bias


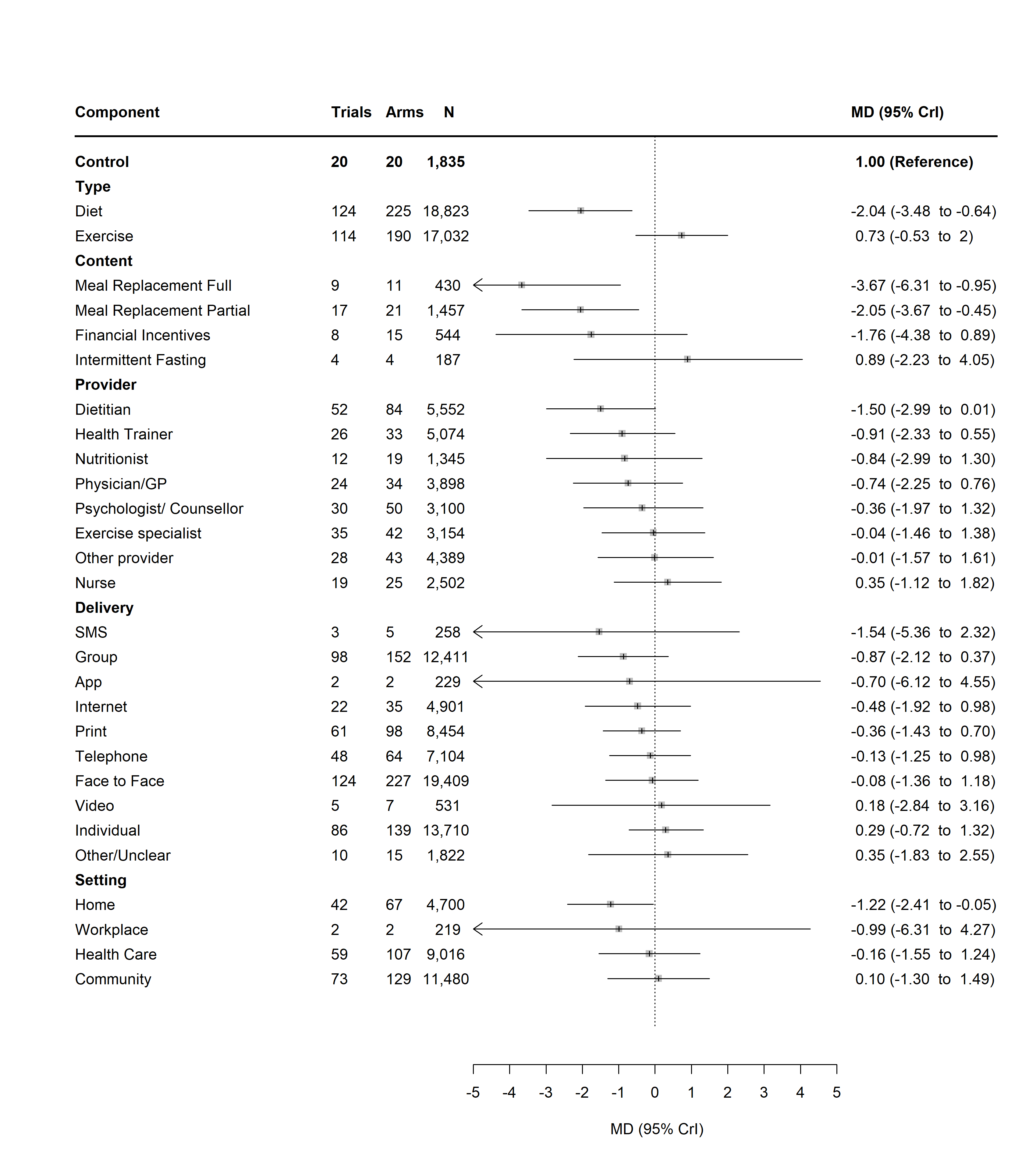


## **Supplemental Figure S7.** Effect estimates from main model, excluding studies reporting only percentage weight change


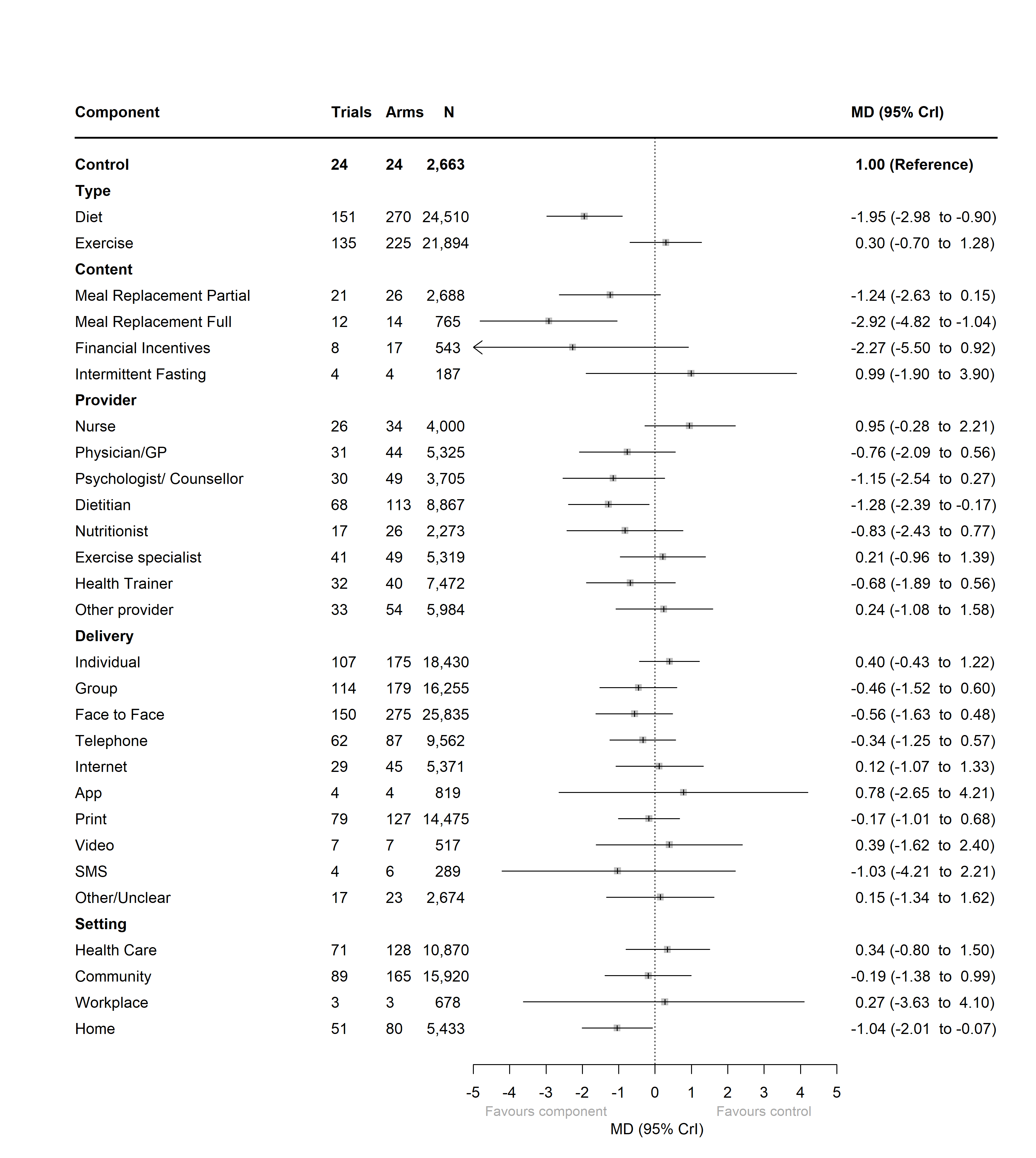


## **Supplemental Table S1**. Characteristics of included studies

| **Study ID** | **Country:** | **Follow-up time points (months):** | **Population** | **Study groups and number of participants randomised** | **Outcome measures extracted*** | **Overall Risk of Bias** | **Author contacted** | **Additional information obtained from author^#^:** | **Notes:** |
| --- | --- | --- | --- | --- | --- | --- | --- | --- | --- |
| Abed 2013 | Australia | 3, 6, 9, 12, 15 | Patients with symptomatic atrial fibrillation | Control = 75  Weight management = 75 | Weight; TC; HDL; SBP; FBG; Plasma insulin | High | Y | N |  |
| Ackermann 2011 | USA | 6, 14 | DPP population | Standard advice alone (controls) = 46  YMCA DPP intervention = 46 | Weight; TC; HDL; SBP; HBa1C | Unclear | N | N/A |  |
| Agras 1990 | USA | 3, 6, 12 | Overweight women without additional psychological disorders | Computer alone = 30  Computer + group support = 30 Behaviour therapy = 30 | Weight | Unclear | N | N/A |  |
| Ahern 2017 | UK | 3, 12, 24 | Adults with a BMI ≥ 28 | Brief intervention = 211  12-week behavioural weight-loss programme = 530  52-week behavioural weight-loss programme = 528 | Weight; TC; SBP; HBa1C; QoL | Low | N | N/A |  |
| Almanza -Aguilera 2018 | Spain | 3, 12 | Metabolically healthy obese women (definition based on the general criteria proposed by the International Diabetes Federation (IDF)) | Control (general recommendations) = 48  Treatment (lifestyle weight loss intervention) = 67 | Weight; TC; HDL; SBP; FG | High | Y | Y | Information provided. |
| Anderson 2014 | Scotland | 3, 12 | Overweight or obese adults (aged 50 to 74 years) who had undergone colonoscopy after a positive faecal occult blood test result, as part of the national bowel screening programme, and had a diagnosis of adenoma confirmed by histopathology. | Control (weight loss booklet only) = 166  Intervention (BeWEL) = 163 | Weight; TC; HDL; SBP; HBa1C; HOMA-IR; FG; Plasma insulin | Low | N | N/A |  |
| Annesi 2016 | USA | 3, 6, 12, 24 | Women who are obese | Comparison treatment = 55  Experimental treatment = 55 | Weight | Unclear | N | N/A |  |
| Annesi 2017 | USA | 3, 6, 12, 24 | Women with class 1 or 2 obesity (BMI ≥ 30 < 40 kg/m2). | Control comparison group = 54 Experimental group = 53 | Weight | Unclear | N | N/A | Information available from previous reviews. |
| Appel 2011 | USA | 6, 12, 24 | Adults who were at least 21 years of age with obesity and had one or more cardiovascular risk factors (hypertension, hypercholesterolemia, or diabetes). | Control (Self-directed) = 138 Remote Support Only (N/A) = 139 In-Person Support = 138 | Weight; TC; SBP; FG; QoL | Low | N | N/A | Information available from previous reviews. |
| Ard 2018 | USA | 6, 12 | General population of adults aged 65 and older who were at risk for cardiometabolic disease due to obesity and associated risk factors | Exercise Only = 54  Exercise + Diet Quality + Weight Maintenance = 55  Exercise + Diet Quality + Weight Loss = 55 | Weight; TC; HDL; SBP; FG; QoL; Incidence CV morbidity | Low | N | N/A | Information available from previous reviews. |
| Ash 2006 | Australia | 3, 6, 12 | General population and hospital referrals (one public hospital and one private hospital) overweight and obese | Control Group - Booklet only = 63 Individualised Dietetic Treatment = 66  Fat Booters Incorporated = 62 | Weight | High | N | N/A |  |
| Ashley 2001 | USA | 0.46, 0.69, 0.92, 1.15, 1.38, 1.61, 1.84, 2.07, 2.3, 2.53, 2.76, 3, 3.22, 3.45, 3.68, 3.91, 4.14, 4.37, 4.6, 4.83, 5.06, 5.29, 5.52, 5.75, 6, 6.21, 6.44, 6.67, 6.9, 7.36, 7.83, 8.29, 8.75, 9.21, 9.67, 10.13, 10.59, 11.05, 11.51, 12, 24 | General population (premenopausal women) | Control, Diet = 37 MR - Physician/Nurse led = 38  MR - Dietician lead = 38 | Weight; TC; HDL; SBP; FG; Plasma insulin | High | N | N/A | Information available from previous reviews. |
| Ashley 2007 | USA | 6, 12 | Generally healthy overweight/obese women | Control - Traditional Food Group = 35 Meal Replacement Group = 35 | Weight | Unclear | N | N/A | Information available from previous reviews. |
| Aveyard 2016 | England | 3, 12 | General population | Advice only = 942  Advice plus weight loss programme = 940 | Weight | Low | N | N/A |  |
| Bacon 2002 | USA | 3, 6, 12, 24 | Women from the general population | Health at Every Size – control = NS  Diet Group – intervention = NS | Weight; TC; HDL; SBP; QoL | Unclear | N | N/A | Information available from previous reviews. |
| Bartels 2015 | USA | 3, 6, 9, 12, 18 | People with serious mental illness | Control, Fitness club membership = 106 IN SHAPE = 104 | Weight; TC; HDL; SBP | Unclear | N | N/A | Information available from previous reviews. |
| Beeken 2017 | England | 3, 6, 12, 18, 24 | General population with obesity | Usual care = 270 10TT = 267 | Weight; TC; SBP; FBG | Low | Y | Y | Information provided. |
| Bennett 2012 | USA | 6, 12, 18, 24 | Obese patients receiving hypertensive treatment | Control, Usual care = 185 Be Fit, Be Well = 180 | Weight; SBP, QoL | Unclear | Y | Y | Data provided; Information available from previous reviews. |
| Bennett 2013 | USA | 6, 12, 18 | General population | Control, usual care = 97  Weight gain prevention intervention = 97 | Weight; TC; HDL; SBP; FG; QoL | Unclear | Y | Y | Data provided. |
| Berry 2014 | USA | 3, 12, 18 | Parent and child dyad with overweight or obesity | Control = 162  Family based. Nutrition, exercise and coping skills intervention = 184 | Weight; QoL | High | Y | Y | Data provided; Information available from previous reviews. |
| Bertz 2012 | Sweden | 3, 12 | Women, 8-12-week post-partum | Control = 17  Diet Only = 17 Exercise only = 18 Intervention = 16 | Weight; TC; SBP; FG; QoL | Unclear |  |  | Information available from previous reviews. |
| Beutel 2006 | Germany | 1.5, 12, 36 | Patients referred for inpatient psychosomatic rehabilitation by the health and pension insurance companies on the basis of obesity plus additional psychiatric morbidity and a reduced or threatened work capacity | Behavioural therapy = 130 Psychodynamic treatment = 137 | Weight | Unclear | N | N/A |  |
| Bliddal 2011 | Denmark | 7.4, 8.3, 12 | Overweight patients with primary knee osteoarthritis | Control, low-energy diet = 45  Intensive low-energy diet = 44 | Weight | High | N | N/A |  |
| Bo 2007 | Italy | 12, 24 | General population (70-72% with metabolic syndrome) | Control standard care = 188 Intervention lifestyle by trained professional = 187 | Weight; TC; HDL; SBP; FG | Low | N | N/A |  |
| Brown 2014 | USA | 3, 6, 12 | Clients at one of four community mental health programs, three in the Kansas City area and one in Las Vegas | Control = 66  RENEW = 70 | Weight | Unclear | N | N/A |  |
| Burke 2015 | USA | 6, 12, 18 | General population | Standard behavioural weight loss treatment = 72 Self-efficacy enhancement plus standard behavioural weight loss treatment = 58 | Weight; QoL | Unclear | N | N/A |  |
| Chee 2017 | Malaysia | 6, 12 | Patients with type 2 diabetes | Usual Care = 115 tDNA Conventional Counseling = 57  tDNA Motivational Interviewing = 58 | Weight; TC; HDL; SBP; HbA1c | Unclear | Y | N | Information available from previous reviews. |
| Cheyette 2007 | UK | 4, 6, 12 | Patients with type 2 diabetes on insulin treatment | Control = 20 Weight No More intervention group = 29 | Weight; HbA1c; QoL | Unclear | N | N |  |
| Christensen 2012 | Denmark | 3, 12 | Female overweight healthcare workers | Reference group = 44 Intervention group = 54 | Weight; SBP | Low | N | N/A |  |
| Cleo 2018 | Australia | 3, 12 | General population | Wait list control (N/A) = 25  TTT Top Ten Tips habit formation = 25 DSD Do Something Different online software = 25 | Weight | Unclear | N | N/A |  |
| Cole 2013 | USA | 3, 12 | Department of Defense beneficiaries enrolled in the TRICARE health care system living in the San Antonio, Texas, area; Diagnosis of pre -diabetes. | Control - individualised counselling = 31 Intervention- shared medical appointment = 34 | Weight; TC; HDL; SBP; HbA1C; FG | Unclear | N | N/A |  |
| Conroy 2015 | USA | 3, 12 | General population | Self-guided = 50 Interventionist led = 49 | Weight; SBP | Unclear | N | N/A |  |
| Cooper 2010 | UK | 6, 10, 16, 22, 34, 46 | General population | Guided Self-Help Control = 51 Behaviour Therapy = 50 Cognitive Behaviour Therapy = 49 | Weight | Low | N | N/A |  |
| Cousins 1992 | USA | 3, 6, 12 | General population | Control = 56 Individual = 56 Family = 56 | Weight | Unclear | N | N/A |  |
| Craighead 1989 | USA | 3, 12 | General population | Control, minimal contact = 20 Contracted Exercise = 20 Supervised Exercise = 22 | Weight | Unclear | N | N/A |  |
| Crowley 2017 | USA | 3.7, 7.4, 11.1 | Veterans with type 2 diabetes | Group Medical Visit = 136 Intensive Weight Management Group Medical Visit = 127 | Weight; TC; HDL; SBP; HbA1c | High | Y | N | Information provided. |
| Dale 2009 | New Zealand | 4, 8, 12, 24 | Insulin resistant adults | Control = 23 Modest = 31 Intensive intervention = 25 | Weight | Unclear | Y | N | Information available from previous reviews. |
| Dalziel 2006 | France | 2, 12, 48 | Patients who had experienced their first myocardial infarction. | Control = 303  Experimental = 302 | Weight; TC; HDL; SBP; Final follow-up only: Incidence CV morbidity; Incidence CV mortality | High | N | N/A |  |
| Damschroder 2014 | USA | 3, 12, 18, 24 | Veterans | Control, MOVE - usual care = 159  ASPIRE group, individual telephone counselling = 162  ASPIRE group, group counselling = 160 | Weight; HDL; SBP; HbA1c; QoL | Unclear | N | N/A | Information available from previous reviews. |
| Daubenmier 2016^ | USA | 3, 6, 12, 18 | Adults with obesity | Active control intervention = 94 Mindfulness Intervention = 100 | Weight; TC; HDL: SBP; HbA1c; | Low | N | N/A |  |
| Delahanty 2015 | USA | 6, 12 | Patients with type 2 diabetes | Dietitian Referral group = 29 Group lifestyle intervention = 28 | Weight; TC; SBP; HbA1c; One timepoint only: Remission HTN | High | Y | Y | Data provided. |
| deRoon 2017 | Netherlands | 4, 12 | General population | Control = 48 Diet = 97 Exercise = 98 | Weight; QoL | High | N | N/A |  |
| deVos 2016 | Netherlands | 6, 12, 18, 24, 30, 80 | Females 50 to 60 years | Control = 204 Tailor-made lifestyle intervention = 203 | Weight; TC; HbA1c; QoL | Unclear | Y | Y | Data provided. |
| Diabetes Prevention Program R G 2009 | USA | 6, 12, 18, 24, 30, 36, 42, 48, 54, 60, 66, 72, 78, 84, 90, 96, 102, 108, 114, 120, 126, 132, 138, 144, 150, 156, 162, 168, 174, 180, 186 | People at high risk for type 2 diabetes (impaired glucose tolerance) | Placebo = 1082 Metformin (N/A) = 1073  Lifestyle = 1079 | Weight; HDL; SBP; HbA1c; FG; QoL; Incidence T2DM | High |  |  | Information available from previous reviews. |
| Djuric 2002 | USA | 3, 6, 12 | Women with stage I or II breast cancer diagnosed within the past 4 years and free of any recurrence. | Control = 13 Weight Watchers = 11  Individualized group = 13 Comprehensive group = 11 | Weight; TC:HDL ratio; TC; HDL; FG; Plasma insulin | High | Y | N | Information available from previous reviews. |
| Duncan 2016 | New Zealand | 4, 12 | Primary health care patients with an elevated 5-year cardiovascular disease risk | Control = 162  Intervention = 158 | Weight; TC:HDL ratio; TC; HDL; SBP. | High | N | N/A |  |
| Eaton 2016 | United States | 6, 12, 18, 24 | General population | Control, Standard Intervention = 106  Enhanced Intervention = 105 | Weight | Low | N | N/A | Information available from previous reviews. |
| Fahey 2018 | Texas, United States | 4, 12 | Active duty military personnel | Self-paced condition = 124  Counselor- initiated condition = 124 | Weight | High | N | N/A |  |
| Fernandez-Ruiz 2018 | Spain | 6, 12, 24 | General population (Community Care Centre population (health centre patients)) | Control = 37  Intervention (healthy eating, exercise & CBT) = 37 | Weight; TC; SBP; HbA1C; QoL; Incidence CV mortality | Unclear | N | N/A |  |
| Finkelstein 2017 | Singapore | 4, 8, 12 | General population | Control = 54  Financial Reward = 107 | Weight | Low | N | N/A |  |
| Fisher 2011 | USA | 6, 12 | Community (overweight, premenopausal women) | Diet only = 29  Diet + aerobic training = 43  Diet + resistance training = 54 | Weight; FG; Plasma insulin | Unclear | N | N/A |  |
| Foley 2016 | USA | 6, 12 | Obese (BMI: 30.0-44.9 kg/m2) community health center patients with a diagnosis of hypertension, diabetes and/or hyperlipidemia | Usual care (Control) = 175  Weight loss intervention = 176 | Weight; TC; HDL; SBP; HbA1c; FG; QoL | Unclear | Y | Y | Data provided. |
| Foreyt 1993 | USA | 3, 12, 24 | General population | Control (N/A) N = 38  Exercise only = 43  Diet only = 42  Exercise plus diet = 42 | Weight | Unclear | N | N/A |  |
| Foster-Schubert 2012 | USA | 6, 12 | Post-menopausal women | Control- usual care = 87  Calorie reduced diet = 118  Aerobic exercise (N/A) = 117  Intervention - diet and exercise = 117 | Weight; HOMA-IR; FG; Plasma insulin; QoL | Unclear | N | N/A |  |
| Freitas 2017 | Brazil | 3, 6, 12 | 30 to 60-year-old patients with moderate/severe asthma | Weight loss program + Sham = 27  Weight loss program + Exercise = 28 | Weight | High | Y | Y | Data provided. |
| Fuller 2012 | Australia | 1, 2, 3, 6, 9, 12 | Male or female residents of inner western Sydney aged 18—65 years, with a BMI of  25—45 kg/m^2^ | Western diet group = 35  Korean diet group = 35 | Weight; TC; HDL; SBP; FG; One timepoint only: Plasma Insulin | Unclear | Y | N |  |
| Gold 2007 | USA | 6, 12 | General population | Commercial programme eDiets = 62 Structured VTrim = 62 | Weight | Unclear | N | N/A | Information available from previous reviews. |
| Goodwin 2014 | Canada; USA | 6, 12, 18, 24 | Postmenopausal women diagnosed with T1-3N0-3M0 breast cancer | Mailed-based intervention = 167 Individual lifestyle intervention = 171 | Weight; QoL | Unclear | Y | N | Author response received. |
| Hakala 1993 | Finland | 0.5, 3, 8 12, 24, 60 | General population | Individual community-based counselling = 28  Group in-patient rehabilitation = 30 | Weight | Unclear | N | N/A |  |
| Hanson 1976 | USA | 2.5, 5, 12 | General overweight and obese population. | No treatment control condition (N/A) = 10  Attention-placebo control condition = 11  Conventional self-management condition = 7 Programmed text with low therapist-group contact = 12 Programmed text with high therapist-group contact = 13 | Weight | High | N | N/A |  |
| Harrigan 2016 | USA | 6, 12 | Breast cancer survivors | Usual Care Group = 33 Telephone Weight Loss Counseling = 34 In-Person Weight Loss Counseling = 33 | Weight; FG | High | Y | Y | Data provided. |
| Hunt 2014 | UK | 3, 12 | Male football fans | Control, Wait-list = 373 FFIT = 374 | Weight; SBP; QoL | High | N | N/A | Information available from previous reviews. |
| Huseinovic 2016 | Sweden | 3, 12, 24 | Women 6–15 week postpartum | Control Group = 56  Diet behaviour modification Group = 54 | Weight; QoL | Low | N | N/A |  |
| Irwin 2003 | USA | 3, 12 | General postmenopausal female population | Control Group = 86  Exercise group = 87 | Weight; HOMA-IR; FG | Low | N | N/A | Information available from previous reviews. |
| Jakicic 2011 | USA | 6, 12, 18 | Overweight, sedentary adults | Self Help Group = 89 Moderate Physical Activity = 82 High Physical Activity = 98 | Weight | Unclear | N | N/A |  |
| Jakicic 2015 | USA | 6, 12, 18 | Adults up to 55 years of age who are overweight or obese. | Standard behavior weight loss interventions group = 71 ADOPT group = 71 MAINTAIN Group (N/A) = 71 | Weight | Low | N | N/A |  |
| Jebb 2011 | Australia, Germany, UK | 2, 4, 6, 9, 12, 18, 24 | Adults with a BMI 27-35 and at least one additional risk factor for obesity-related disease | Standard care = 395 Commercial programme = 377 | Weight; TC:HDL ratio; TC; SBP; FG; Incidence T2DM; Remission T2DM | Low | Y | Y | Data provided; Information available from previous reviews. |
| Jebb 2017 | UK | 3, 6, 12, 36 | Obese adults seeking support to lose weight | Usual care = 140 Low energy total diet replacement programme = 138 | Weight; TC; HDL; SBP; HbA1c; FG; Plasma insulin; QoL; One timepoint only: HOMA-IR | Low | N | N/A |  |
| Jeffery 2003 | USA | 6, 12, 18 30 | Overweight population | Standard behaviour therapy = 93 High physical activity = 109 | Weight | Unclear | Y | Y | Information provided. |
| Jolly 2011 | UK | 3, 12 | Obese or overweight men and women with a comorbid disorder identified from general practice records, with a raised BMI recorded within their primary care notes within the previous 15 months. The BMI threshold for invitation is that which makes them eligible for primary care obesity management services within the NHS and varies according to ethnic group and the presence or absence of comorbidities. | Minimal intervention comparator = 100 Choice (N/A) = 100  Pharmacy =70  General practice = 70 Weight Watchers = 100 NHS Size Down = 100 Rosemary Conley = 100  Slimming world = 100 | Weight | High | N | N/A | Information available from previous reviews. |
| Jones 1999 | USA | 3, 6, 12, 18, 24, 30 | Patients with hypertension above the age of 50 | Control Group = NS  Weight Loss Group = NS | Weight | Unclear | Y | Y | Information provided. |
| Katula 2013 | USA | 6, 12, 18, 24 | People with pre-diabetes (fasting blood glucose=95 mg/dl ≤FBG ≤125) | Enhanced Usual Care Comparison Condition = 150  Lifestyle Weight-Loss Intervention = 151 | Weight; HDL; SBP; HOMA-IR; Incidence T2DM | Low | Y | N |  |
| Keogh 2014 | Australia | 2, 12 | General population | Intermittent dieting = 39 Continuous dieting = 36 | Weight | High | N | N/A |  |
| Keranen 2009 | Finland | 1,3, 4.6, 5, 6, 12, 18 | General population | Short-term counselling = 47  Intensive counselling = 35 | Weight | Low | N | N/A | Information available from previous reviews. |
| King 1989 | USA | 7, 12, 24 | Men aged 30 - 59 years. | Control (N/A) = 52 Exercise only = 52 Diet only = 51 | Weight; TC/HDL ratio; TC | High | N | N/A |  |
| Knauper 2018 | Canada | 3, 12, 24 | Individuals with overweight or obesity | Standard DPP = 101  Enhanced DPP = 107 | Weight; TC/HDL ratio; SBP; HbA1c | High | Y | Y | Data provided. |
| Kumanyika 2012 | USA | 12, 24 | General population (65% African American, non-Hispanic black) | Basic programme = 137 Basic plus programme = 124 | Weight; SBP | Low | N | N/A | Information available from previous reviews. |
| Leahey 2014 | USA | 3, 6, 12 | General population | SURI alone = 46 SURI plus Internet behavioral weight loss program = 90  SURI plus Internet behavioral weight loss program plus optional group sessions = 94 | Weight | Unclear | N | N/A |  |
| Leahey 2015 | USA | 3, 12 | Adults | SURI1 Internet behavioral weight loss = 91 SURI1 Internet behavioral weight loss1incentives = 89 SURI1 Internet behavioral weight loss 1 group option = 88 | Weight | Unclear | N | N/A |  |
| Lejeune 2003 | The Netherlands | 3, 12.2 | Men with obesity | Diet = 20 Diet plus exercise = 20 | Weight | Unclear | Y | N | Information available from previous reviews. |
| Ley 2004 | New Zealand | 6, 12, 24, 36, 60 | Workers with impaired glucose tolerance ((2 h blood glucose 7.8–11.0 mmol/l) and a further 114 (2%) had high normal blood glucose concentrations (7.0–7.8 mol/l)) | Control diet = 70  Reduced-fat = 66 | Weight; TC/HDL ratio; TC; HDL; SBP | High | N | N/A |  |
| Li 2016 | China | 1, 12 | Adults with Type 2 Diabetes Mellitus who are overweight (BMI ≥ 24 kg/m2) | Usual care group = 60  Diet group = 79  50g-oats group = 80  100g-oats group = 79 | Weight; TC; HbA1c | Unclear | Y | N |  |
| Li 2005 | USA | 0.5, 1.5, 2, 3, 4, 5, 6, 7, 8, 9, 10, 11, 12 | Adults previously diagnosed and being treated for type II Diabetes Mellitus who are obese | Individualized diet plan = 52 Soy-based meal replacement = 52 | Weight; TC; HbA1c | High | Y | N |  |
| Lindstrom 2003 | Finland | 12, 24, 36, 48, 60, 72, 86, 96, 108, 120 | Impaired glucose tolerance (IGT); People at high risk for type 2 diabetes | Control = 257 Intervention = 265 | Weight; TC/HDL ratio; TC; HDL; SBP; HbA1c; FBG; Incidence DM; Incidence CVD morbidity; One timepoint only: Plasma insulin | High | Y | N | Information available from previous reviews. |
| Liss 2016 | USA | 6, 12 | Adults with type 2 diabetes and a BMI ≥ 24 kg/m2 | Standard care arm = 167 Standard care plus group-based lifestyle intervention = 164 | Weight; TC; SBP; HbA1c | Low | Y | Y | Data provided. |
| Little 2016 | UK | 6, 12 | General population | Control, Nurse follow-up = 279 Web-based support with minimal support (Remote) = 270 Web-based + nurse support (face to face) = 269 | Weight; TC; HDL; SBP; HbA1c; FG; QoL | Unclear | N | N/A | Information available from previous reviews. |
| Lowe 2018 | USA | 6, 12, 18, 24 | Community | Behavior therapy = 90 Behavior therapy plus meal replacements = 91 Home food environment = 81 | Weight | Unclear | N | N/A | Information available from previous reviews. |
| Ma 2015 | USA | 6, 12 | Obese adults with uncontrolled asthma | Control, Enhanced usual care = 165 Diet and counselling = 165 | Weight | Low | N | N/A | Information available from previous reviews. |
| Manning 1994 | UK | 3, 6, 12, 48 | Diabetic males and females | Clinic visit = 37  Behavioural = 38 Home visits = 35  Dexfenfluramine (N/A) = NS Routine usual care (N/A) = NS | Weight; HbA1c | Unclear | N | N/A |  |
| Manzoni 2016 | Italy | 1.4, 12, | Obese patients admitted to the obesity unit of the Istituto Auxologico Italiano, Verbania, Italy for the treatment of obesity and related comorbidities | Control, Standard behavioral inpatient program = 52  Cognitive–behavioral therapy = 54  CBT + Virtual reality = 57 | Weight | High | N | N/A | Information available from previous reviews. |
| Marniemi 1990 | Finland | 2.5, 6, 12 | General obese and overweight population | Control group = 42  Lactovegetarian weight reduction group = 31  Mixed diet weight reduction = 37 | Weight | Unclear | N | N/A |  |
| Martin 2008 | USA | 6, 9, 12, 18 | African American women | Control, Standard Care = 69 Tailored physician/lifestyle counselling = 68 | Weight | High | N | N/A | Information available from previous reviews. |
| Mefferd 2007 |  | 4, 12 | Adult breast cancer survivors with a BMI ≥ 25.0 kg/m^2^ | Control = 29 Intervention = 56 | Weight; TC | Unclear | Y | Y | Data provided; Information available from previous reviews. |
| Mengham 1999 | UK | 6, 12 | Patients with diabetes, aged less than 75 years, with BMI above 25kg/m^2^ | Control = NS  Intervention = NS | Weight; TC | Unclear | N | N/A | Information available from previous reviews. |
| Messier 2013 | USA | 1, 2, 3, 4, 5, 6, 7, 8, 9, 10, 11, 12, 13, 14, 15, 16, 17, 18 | Ambulatory, community-dwelling persons age 55 years or older with mild or moderate knee osteoarthritis | Exercise only = 150  Diet-induced weight loss only = 152  Diet-induced weight loss plus exercise = 152 | Weight; QoL | Unclear | Y | Y | Data provided. |
| Miller 2002 | USA | 2, 12 | NS | Control Group (Monitoring) = 23 Lifestyle Intervention = 22 | Weight; SBP | Low | Y | N | Information available from previous reviews. |
| Mitsui 2008 | Japan | 3, 12 | 50-69-year-old adults | Control = 22  Intervention = 24 | Weight; TC; HDL; SBP; FG | Unclear | Y | N |  |
| Molenaar 2010 | Netherlands | 6, 12 | General overweight or obese population | Nutritional counselling group (diet D, group) = 67 Nutritional plus exercise counselling group (diet + exercise (D + E) group) = 67 | Weight | Unclear | N | N/A |  |
| Moreno 2014 | Spain | 0.5, 2, 4, 6, 8, 10, 12, 18, 24 | Patients with obesity and prediabetes attending a hospital obesity unit (Obesity Unit, Hospital Gregorio Maranon, Madrid) | Low-calorie diet = 39  Very low-calorie-ketogenic diet = 40 | Weight; TC; HDL; HbA1c; FG | Unclear | Y | N | Information available from previous reviews. |
| Morgan 2010 | Australia | 3, 6, 12 | Males 18-60 years of age who are overweight or obese. | Control (Information and self-help) = 31 SHED-IT (Internet) group = 34 | Weight; SBP | Low | N | N/A | Information available from previous reviews. |
| Muggia 2014 | Italy | 6, 12 | Overweight and obese patients | Standard care group = 83 Brief CBT group = 80 | Weight; TC; HDL; SBP; HOMA-IR; FG; Plasma insulin | High | N | N/A |  |
| Ng 2015 | UK | 4, 12 | Chinese patients with moderate to severe obstructive sleep apnoea (OSA) diagnosed on portable home sleep monitoring. | Control group = 43 Lifestyle modification program = 61 | Weight; TC; FG; QoL | Unclear | Y | Y | Data provided. |
| Nicklas 2009 | USA | 4.6, 10.6, 16.6 | Postmenopausal women with abdominal obesity | Calorie restriction (CR) Only = 34 CR + Moderate-Intensity = 40 CR + Vigorous-Intensity = 38 | Weight; HDL; Glucose tolerance | Unclear | Y | Y | Data provided. |
| Nilsen 2011 | Norway | 6, 12, 18 | Individuals at high risk for type 2 Diabetes | Control, Individual Physician Group = 104  Individual Plus Interdisciplinary Group = 109 | Weight; TC; HDL; SBP; HbA1c; FG | Low | Y | Y | Data provided; Information available from previous reviews. |
| Oldroyd 2006 | UK | 6, 12, 24 | Men and women of European origin | Control group = 39 Intervention group = 39 | Weight; TC; FG | Unclear | N | N/A | Information available from previous reviews. |
| Parikh 2010 | USA | 3, 6, 12 | Adults with BMI ≥ 25 kg/m^2^ and prediabetes | Control = 49 Intervention = 50 | Weight; SBP; HbA1c | Unclear | Y | N |  |
| Pedersen 2013 | Copenhagen | 3, 12 | Adults with stable coronary artery disease who are overweight or obese | Aerobic interval training = 35 Low energy diet = 35 | Weight; TC/HDL ratio; SBP; HbA1c | Unclear | N | N/A |  |
| Perri 1986 | USA | 4.6, 7.6, 10.6, 14.6, 22.6 | Adults 22 to 60 years of age who were between 20%-100% over ideal body weight based on Metropolitan Life Insurance Company norms (1959) | Behavior therapy = NS  Behavior therapy plus maintenance = NS  Behavior therapy plus aerobic exercise = NS  Behavior therapy behavior therapy plus aerobic exercise plus maintenance = NS | Weight | Unclear | N | N/A |  |
| Perri 1997 | USA | 6, 12, 15 | Women 40-60 years of age | WL + Home-based exercise = 24 WL + Group-based exercise = 25 | Weight | Unclear | N | N/A |  |
| Perri 2001 | USA | 5, 11, 17 | General population. | Control, Standard Behavioural Therapy (BT) = NS   BT + Relapse prevention training = NS  BT + problem-solving therapy = NS | Weight | Unclear | N | N/A | Information available from previous reviews. |
| Pettman 2009 | Australia | 4, 12 | Adults with metabolic syndrome | Control = 50 Intervention B - Passive follow-up = 54  Intervention A - Active follow-up = 49 | Weight; TC; SBP; HOMA-IR; One timepoint only: FG | Low | Y | Y | Data provided. |
| Poelman 2015 | Netherlands | 3, 6, 12 | General population | Control Condition = 139 Intervention condition = 139 | Weight | High | Y | Y | Data provided. |
| Promrat 2010 | USA | 3, 6, 9, 12 | Adults who were overweight or obese and diagnosed with Nonalcoholic steatohepatitis | Control = 10 Lifestyle Intervention = 21 | Weight; TC; HbA1c; HOMA-IR | Low | Y | Y | Data provided. |
| Provencher 2009 | Canada | 4, 10, 16 | Premenopausal women | Control group = 48 Social support = 48  Health-At-Every-Size = 48 | Weight; TC; One timepoint only: HDL; | Unclear | Y | Y | Data provided. |
| Rock 2015 | USA | 6, 12, 18, 24 | Patients with early-stage breast cancer | Control = 349 Intervention = 348 | Weight; SBP | Low | N | N/A |  |
| Rolls 2005 | USA | 0.92, 1.8, 2.8, 3.7, 4.6, 5.5, 6.4, 7.4, 8.3, 9.2, 10.1, 11, 12 | Overweight and obese women and men | Comparison-control = 50 Two snacks = 50  One soup = 50  Two soups = 50 | Weight; TC; SBP | Unclear | Y | Y | Data provided. |
| Rolls 2017 | USA | 1, 2, 3, 4, 5, 6, 7, 8, 9, 10, 11, 12 | Women aged 20–65 years BMI of 28–45 kg/m2 | Standard advice = 62  Pre-portioned foods group = 62  Portion selection group = 62 | Weight; TC; SBP; HOMA-IR; FG | Unclear | Y | Y | Data provided; Information available from previous reviews. |
| Rosas 2015 | California | 6, 12, 24 | Participants are obese Spanish-speaking adults with at least one cardiovascular risk factor recruited from a community health center in a low-income neighborhood of San Mateo County, California. | Usual care = 41  Case-management intervention = 84 Case-management + Community health worker intervention = 82 | Weight; TC; HDL; SBP; HbA1c; FG | Unclear | N | N/A |  |
| Ross 2012 | Canada | 6, 12, 18, 24 | General population | Control condition = 241 Behavioral intervention group = 249 | Weight; TC; HDL; SBP; FG | Unclear | N | N/A | Information available from previous reviews. |
| Samaras 1997 | Australia | 6, 12 | Mature-aged people, performing less than 1 hour of exercise per week | Control =13  Intervention = 13 | Weight; TC; HDL; HbA1c; FG; Plasma insulin | Unclear | N | N/A | Information available from previous reviews. |
| Santanasto 2011 | USA | 6, 12 | Community dwelling older men and woman age 60 and over, who were overweight to moderately obese and living a sedentary lifestyle (formal exercise less than 3x/week for a total of less than 90 min/week). | Physical Activity plus Successful Ageing = 15 Physical Activity plus Weight Loss = 21 | Weight | Unclear | N | N/A |  |
| Sattin 2016 | USA | 3, 12 | Obese and overweight, and/or prediabetic (FPG of 100 mg/dl to 125 mg/dl). | Health Education intervention = 287  Fit body and soul intervention = 317 | Weight; FBG | Unclear | N | N/A |  |
| Schubel 2016 | Germany | 3, 5.5, 11.5 | Adults between 35-65 years, non-smokers and who are overweight or obese. | Control group = 52  Continuous Calorie Restriction = 49  Intermittent Calorie Restriction = 49 | Weight; TC; HbA1c; HOMA-IR | Low | Y | Y | Data provided. |
| Seligman 2011 | Brazil | 3, 12 | General population (patients with metabolic syndrome - no diabetics, more than half of the participants were hypertensive) | Standard-of-care strategy = 25 Healthy diet and step counter = 25 Healthy diet and fitness = 26 | Weight; TC; HDL; SBP; HOMA-IR | Low | Y | Y | Data provided; Information available from previous reviews. |
| Shikany 2013 | USA | 6, 12 | General population | Food-based diet = 60 Meal replacement = 60 | Weight; TC; HDL; SBP; FG | Low | N | N/A | Information available from previous reviews. |
| Silva 2010 | Portugal | 4, 12, 36 | Women with overweight or obesity, aged 25 to 50 (and pre-menopausal) | Comparison group = 116 Intervention = 123 | Weight | Unclear | Y | N | Information available from previous reviews. |
| Solbrig 2019 | UK | 6, 12 | General overweight and obese adult population | Motivational interviewing = 58  Functional imagery training = 63 | Weight; QoL | Unclear | N | N/A |  |
| Somers 2012 | USA | 6, 12, 18 | Patients with knee pain and osteoarthritis | Standard Care = 51 Lifestyle behavioral weight management intervention only = 59 Lifestyle behavioral weight management intervention + Pain Coping Skills Training = 62  Pain Coping Skills Training only (N/A) = 60 | Weight | Low | Y | Y | Data provided. |
| Spring 2013 | USA | 3, 6, 9, 12 | Veterans receiving medical care from Veterans' Affairs department | MOVE standard care = 35  MOVE + personal digital assistant = 34 | Weight | Unclear | N | N/A | Information available from previous reviews. |
| Spring 2017 | USA | 3, 6, 12 | General adult population with obesity | Control self-guided program = 32 Standard weight loss program = 32 Technology-supported = 32 | Weight | High | N | N/A |  |
| Stahre 2005 | Sweden | 2.3, 6, 12, 18 | General female population | Control = 43 Cognitive treatment = 62 | Weight | Unclear | N | N/A | Information available from previous reviews. |
| Stenius-Aarniala 2000 | Finland | 3.2, 6, 12 | People with asthma | Control = 19 Treatment with VLCD = 19 | Weight; QoL | Unclear | Y | N | Information available from previous reviews. |
| Stevens 1993 | USA | 3, 6, 12, 18, 276 | Men and women aged 30 to 54 years with high-normal diastolic blood pressure from 80 through 89 mm hg. | Control = 256 Intervention = 308 | Weight; SBP | Unclear | Y | Y | Data provided. |
| Stevens 2001 | USA | 6, 12, 18, 24, 30, 36 | Adults 30 to 54 years of age who had nonmedicated diastolic blood pressure of 83 to 89 mm Hg and systolic blood pressure less than 140 mm Hg and were 110% to 165% of their ideal body weight at baseline. | Control = 596 Intervention = 595 Sodium only intervention (N/A) = 594 Combined intervention (N/A) = 597 | Weight; SBP | Unclear | Y | Y | Data provided. Information available from previous reviews. |
| Strobl 2013 | Germany | 6, 12 | General population | Control, Usual care = 239 Telephone aftercare =228 | Weight | Low | N | N/A | Information available from previous reviews. |
| Sundfor 2018 | Norway | 3, 6, 12 | Men and women aged 21-70 years with BMI 30-45.0 | Continuous energy restriction = 58 Intermittent energy restriction = 54 | Weight; TC; SBP; HbA1c; FG | Low | N | N/A |  |
| Tapsell 2017 | Australia | 3, 12 | Adult residents, 25-54 years, BMI 25-40kg/m2 | Usual care (Control) = 126  Intervention Group = 125 Intervention plus food supplement group (N/A) = 126 | Weight; TC/HDL ratio; TC; SBP; HbA1c; QoL; Remission HTN | High | N | N/A |  |
| TarragaMarcos 2017 | Spain | 0.5, 1, 3, 6, 12 | Adult general obese/overweight population. | G3 = 55 G2 = 61 G1 = 60 | Weight; TC; HDL | Unclear | Y | N |  |
| Teeriniemi 2018 | Finland | 12, 24 | Residents aged 20–60 years living in the city of Oulu who were overweight or obese. | Control = 89  SHG Counselling = 87 CBT Counselling = 85 Control plus HBCSS = 91 SHG Counselling plus HBCSS = 92 CBT Counselling plus HBCSS = 88 | Weight; HDL; SBP; FG | Unclear | Y |  |  |
| ter Bogt 2009 | Netherlands | 12, 36 | Patients 40–70 years of age with BMI: 25 to 40 and either hypertension or dyslipidemia or both. | GP usual care = 232 Lifestyle counselling from NP = 225 | Weight; TC; HDL; SBP; FG | Unclear | N | N/A |  |
| The Look AHEAD Research Group 2010 | USA | 12, 24, 36, 48, 60, 72, 84, 96, 108, 115, 120 | Adults with Type 2 Diabetes Mellitus | Diabetes support and education = 2575 Intensive lifestyle intervention = 2570 | Weight; TC; HDL; SBP: QoL; Incidence CV morbidity; Incidence CV mortality; Incidence T2DM; Remission T2DM | High | N | N/A | Information available from previous reviews. |
| Trepanowski 2017 | USA | 1, 2, 3, 4, 5, 6, 7, 8, 9, 10, 11, 12 | General population with a BMI ≥ 25. | No-intervention control group = 31  Daily calorie restriction group = 35  Alternate-day fasting group = 34 | Weight; HOMA-IR | High | Y | Y | Data provided. |
| Tsai 2010 | USA | 3, 6, 12 | General population | Control = 26 Brief counselling = 24 | Weight; TC; HDL; SBP; FG | Unclear | N | N/A | Information available from previous reviews. |
| Tuomilehto 2009 | Finland | 3, 12, 24, 60 | Patients with mild obstructive sleep apnoea | Control = 41 Intervention = 40 | Weight; TC; HDL; SBP; FBG; Plasma insulin; QoL | Low | N | N/A | Information available from previous reviews. |
| van de Glind 2017 | England, The Netherland, Norway, Portugal | 3, 12 | Males | Comparison group = 553 EuroFIT group = 560 | Weight; TC; SBP; HbA1c; QoL; Final timepoint only: Incidence CV morbidity | High | Y | Y | Information/data received. |
| Viegener 1990 | USA | 1, 2, 3, 4, 5, 6, 12 | General population | Intermittent diet = 42 Standard treatment = 43 | Weight | Unclear | N | N/A |  |
| Vissers 2010 | Belgium | 3, 6, 12 | General overweight or obesity patients. | Control = 21  Diet only group (Diet) = 20  Diet + fitness training group (Fitness) = 20 Diet + WBV group (Vibration) = 18 | Weight; HDL; SBP; FBG; Final follow-up only: Incidence T2DM | Unclear | N | N/A | Information available from previous reviews. |
| Volpe 2008 | USA | 3, 6, 9, 12 | Adults with overweight/obesity | Exercise only = 34  Diet only = 28  Combination of diet and exercise = 28 | Weight; TC/HDL ratio; TC; SBP | High | Y | Y | Data provided. |
| von Gruenigen 2012 | USA | 3, 6, 12 | Women who are overweight with histologically confirmed Stage I or II endometrial cancer | Control = 34 Intervention = 41 | Weight | Unclear | N | N/A | Information available from previous reviews. |
| vonGruenigen 2008 | USA | 3, 6, 12 | Women with endometrial cancer | Control, Usual care = 22 Lifestyle intervention = 23 | Weight; QoL | Unclear | N | N/A |  |
| Wadden 1994 | USA | 0.32, 1, 2, 3, 4, 6, 12, 18 | Women from general population | Balanced deficit diet = 21  Very low-calorie diet = 28 | Weight | Unclear | N | N/A | Information available from previous reviews. |
| Wadden 1998 | USA | 1.85, 3.9, 5.5, 11.1, 23 | Women | Diet alone, Control = NS Diet plus aerobic exercise = NS  Diet plus strength training = NS  Diet plus aerobic and strength training = NS | Weight | Unclear | N | N/A | Information available from previous reviews. |
| Wadden 2004 | USA | 5, 10, 16 | Community obese females | Nondieting approach = 39  Balanced-deficit diet = 43  Meal replacement plan = 41 | Weight | Unclear | N | N/A |  |
| Waleekhachonloet 2007 | Thailand | 3, 6, 12 | Women from a rural community | Individual behavior therapy = 67  Group behavior therapy = 65 | Weight | Unclear | N | N/A |  |
| Weinstock 2013 | USA | 6, 12, 24, 36 | Adults with metabolic syndrome | Conference Call DPP = 128 Individual Call DPP = 129 | Weight; TC; SBP; FG | Unclear | Y | N |  |
| West 2007 | USA | 6, 12, 18 | Women with type 2 diabetes treated by oral diabetes medications but not insulin | Attention control = 108 Motivational interviewing = 109 | Weight; HbA1c | Unclear | N | N/A |  |
| West 2011 | USA | 4, 12 | Older-adult participants | Control = 112 Lifestyle Intervention = 116 | Weight | Unclear | Y | Y | Data provided. |
| Whelton 1998 | USA | 6, 9, 12, 15, 18, 21, 24, 27, 30 | Adults 60-80 years, with systolic blood pressure lower than 145 mm Hg and diastolic blood pressure lower than 85 mm Hg while receiving treatment with a single antihypertensive medication. | Non-weight loss (Usual lifestyle, control group plus sodium reduction) = NS Weight loss (Weight loss alone plus weight loss and sodium reduction combined intervention) = NS | Weight | High | N | N/A | Information available from previous reviews. |
| Wilson 2016 | USA | 3, 6, 12 | General population | Control - Self Study Group = 242  Phone Fuel Your Life = 182  Group Fuel Your Life = 236 | Weight | Unclear | Y | N |  |
| Wilson 2016b | USA | 6, 12 | General population | Control = 457 FUEL Your Life peer health coaches + nurse education = 459 | Weight | High | Y | Y | Data provided. |
| Wing 1988 | USA | 2, 14 | Type 2 diabetes | Diet plus placebo exercise = 13 Diet plus moderate exercise = 12 | Weight; TC; HDL; SBP; HbA1c | Unclear | N | N/A |  |
| Wing 1988b | USA | 2, 14 | Type 2 diabetes patients | Diet only = 15 Diet plus exercise = 15 | Weight; TC; HDL; SBP; HbA1c; FG; Plasma insulin | Unclear | N | N/A |  |
| Wing 1998 | USA | 6, 12, 24 | Overweight participants who had one or two parents with diabetes | Control = 40 Diet = 37 Exercise = 37 Diet plus exercise = 40 | Weight; TC/HDL ratio; TC; HDL; SBP; HbA1c; FG; At final follow-up only: Incidence T2DM | Unclear | N | N/A | Information available from previous reviews. |
| Wing 2010 | USA | 6, 12, 18 | Women over 30 years who are overweight or obese with at least 10 urinary incontinence episodes per week. | Structured Education Program = 112  Weight Loss Intervention (Skills Based maintenance) = 113 Weight Loss Intervention (Motivation Based maintenance) = 113 | Weight | Low | N | N/A |  |
| Yannakoulia 2008 | Greece | 2, 12 | Type 2 diabetes mellitus patients | Usual care group = 15 Intensive care group = 15 | Weight; HbA1c | High | Y | Y | Data provided; Information available from previous reviews. |
| Yardley 2014 | UK | 6, 12 | GP patients | Usual care = 43 Web-based only = 45  Basic nurse support = 44 Regular nurse support = 47 | Weight | High | N | N/A | Information available from previous reviews. |
| Yates 2018 | USA | 4, 12 | Obese, postmenopausal women with prediabetes and normal endometrial biopsy; Participants were recruited from the community, Harris Health System, and employees at MD Anderson Cancer Center | Placebo + no lifestyle = 8 Metformin + no lifestyle (N/A) = 7 Placebo + lifestyle = 7 Metformin + lifestyle (N/A) = 7 | Weight | High | Y | N | Information available from previous reviews. |
| Yeh 2003 | USA | 6, 12, 24 | General population (women) | Counseling based intervention = 40 Skills based intervention = 40 | Weight; | High | N | N/A |  |
| Yin 2018 | China | 6, 12 | Women with pre-diabetes | Comparison-Control Group = 75 Intervention Group = 109 | Weight; HbA1c; FG | Unclear | N | N/A |  |
| Zhang 2016 | China | 6, 12, 24 | Patients with Nonalcoholic Fatty Liver Disease | Control = 74 Moderate exercise = 73 Vigorous-moderate exercise = 73 | Weight; TC; HDL; SBP; FG | Low | N | N/A | Information available from previous reviews. |
| **BMI:** Body Mass Index (kg/m^2^); **CBT:** Cognitive Behaviour Therapy; **CV:** Cardiovascular; **DPP:** Diabetes Prevention Program; **FG:** Fasting glucose (including fasting plasma glucose and other glucose measures)**; HbA1c:**  Haemoglobin A1C**; HDL:** High-density lipoprotein cholesterol; **N:** No; **HOMA-IR:** Homeostatic Model Assessment of Insulin Resistance**; HTN:** Hypertension; **MI:** Motivational interviewing; **MR:** Meal replacement; **N/A:** Not applicable; **NS:** Not specified; **QoL:** Quality of Life; **SBP:** Systolic Blood Pressure; **T2DM:** Type 2 Diabetes Mellitus; **TC:** Total cholesterol; **TC/HDL**: Total cholesterol/High-density lipoprotein ratio; **VLCD:** Very low calorie diet; **Y:** Yes.  *Not all outcomes measures collected at all follow-up time points; Outcome measures collected at baseline only not listed.  ^#^ Additional information or data obtained from study authors.  ^Study only included in the CNMA involving intensity components. | | | | | | | | | |

## **Supplemental Table S2.** Risk of bias judgements across included studies

| **Study ID** | | **Random sequence generation  (Selection bias)** | **Allocation concealment**  **(Selection bias)** | **Blinding of outcome assessment**  **(Detection bias)** | **Incomplete outcome data**  **(Attrition bias)** | **Other bias** |
| --- | --- | --- | --- | --- | --- | --- |
| **1** | **Abed 2013** | UNCLEAR | UNCLEAR | LOW | LOW | HIGH |
|  | Assessment justification: | Single-centre, partially blinded RCT. | No explicit information regarding allocation concealment provided:  Study coordinators, treating physicians, and other personnel, with the exception of weight loss counselors, were blinded to randomization. Patients were instructed not to disclose their status. Patient records contained generic statements without indicating group allocation. | Weight objectively measured. | At 12 months, 109 (73%) had completed the study (57 in the intervention group and 52 in the control group). By 15 months, 81 (54%) remained (42 in the intervention group and 39 in the control group). | Weight data at 3, 6, 9 and 12 months from a sub-study (Abed 2015). 87 participants agreed to CMR (cardiac magnetic resonance) imaging (43 in control group, 44 in intervention group at baseline). 69 participants had baseline and 12-month follow-up (33 in control group, 36 in intervention group at 12 months). |
| **2** | **Ackermann 2011** | UNCLEAR | UNCLEAR | LOW | LOW |  |
|  | Assessment justification: | "matched-pair, group-randomized pilot intervention trial involving two YMCA facilities in greater Indianapolis."  No further information provided regarding matching. | NS | Body weight was measured using a calibrated, beam-balanced scale with participants wearing light clothing and no shoes. | Less than 50% attrition at 12-month follow-up. |  |
| **3** | **Agras 1990** | LOW | UNCLEAR | LOW | LOW |  |
|  | Assessment justification: | "computer generated table of random numbers" | NS | Weight objectively measured. | 12-months follow-up:  29/30 computer alone; 29/30 computer + group;  30/30 behaviour therapy |  |
| **4** | **Ahern 2017** | LOW | LOW | LOW | LOW |  |
|  | Assessment justification: | "The randomisation sequence was generated by the trial statistician and allocates participants in a 2:5:5 allocation stratified by centre and gender, with a block size of 12." | " The sequence is unknown to research staff and participants.” | Weight objectively measured.  "Weight and fat mass will be measured in kg using a Tanita segmental body composition analyser." | 3-months retention rate:  Brief intervention: 68%;  12-week programme: 76.4%;  52-week programme: 86.2%;  12-month retention rate:  Brief intervention: 58.7%;  12-week programme: 63.9%;  52-week programme: 68.2%;  24-month retention rate:  Brief intervention: 63%;  12-week programme: 67%;  52-week programme: 69.7%; |  |
| **5** | **Almanza -Aguilera 2018** | UNCLEAR | UNCLEAR | LOW | HIGH |  |
|  | Assessment justification: | Participants were randomly allocated to either the control or the treatment group.  No further information given. | NS | "Anthropometric measurements, including weight, height, waist circumference (WC), and BMI, were taken by trained nurses..." | "Of the 115 participants recruited, 58 were excluded due to dropout or failure to show at all visits (n = 43), illness (n = 6), unavailable sample at some time point (at baseline, 3 or 12 months, n = 7), or change of residence (n = 2). Therefore, 57 participants were included in the present data analyses." Control n = 27 analysed out of 48 randomised; Treatment n = 30 analysed out of 67 (44.7% retained). |  |
| **6** | **Anderson 2014** | LOW | LOW | LOW | LOW |  |
|  | Assessment justification: | “A statistician, independent of the analysis of study outcomes, had previously generated a randomisation list (site specific identification numbers and group allocation) by using a permuted block technique, with block sizes of four and eight, stratified by trial site.” | “This list was emailed to the study administrator and trial manager. Research nurses allocated participants a site specific identification number sequentially and notified the study administrator on completion of baseline measures for each participant. The study administrator then identified the participant’s group allocation from the randomisation list and notified the lifestyle counsellor of participants allocated to the intervention group or sent the weight loss booklet to participants allocated to usual care.” | Weight objectively measured.  “The study team, including the research nurses, were blinded to the participant’s group allocation until completion of the primary outcome analysis. Exceptions were the trial manager, study administrator, lifestyle counsellors, and participants who could not be blinded owing to the nature of the intervention. None of these unblinded staff had a role in data analysis.” | “The remaining 329 were randomised (163 to intervention, 166 to control). At three months 314 (94% intervention, 97% control) participants had completed the primary outcome measures, and 305 (91% intervention, 95% control) completed the trial at 12 months (93%).” |  |
| **7** | **Annesi 2016** | UNCLEAR | UNCLEAR | LOW | LOW |  |
|  | Assessment justification: | NS | NS | Weight objectively measured. | "Attrition from initial study acceptance to actual treatment participation was minimal at 7% and also did not significantly differ by group." |  |
| **8** | **Annesi 2017** | UNCLEAR | UNCLEAR | LOW | LOW |  |
|  | Assessment justification: | "To avoid cross-contamination of participants and instructors, randomisation to either the experimental (n=53) or comparison (n=54) condition was by the participating community wellness centres (3 for each condition).”  Unclear if cluster randomised.  No further information given. | To minimise expectation and cross-contamination effects, wellness leaders were trained in only 1 of the protocols by study staff and blinded to study goals.  No further information given. | Weight objectively measured. | “Because the requirement of data being missing at random (no systematic bias) was present, the expectation-maximisation algorithm was used for the 12% of cases necessitating imputation within the present intention-to-treat format.” Indicates 12% drop out, so 94/107 completed |  |
| **9** | **Appel 2011** | LOW | LOW | LOW | LOW |  |
|  | Assessment justification: | "Randomization was stratified according to sex and was generated in blocks of 3 and 6 with the use of a Web-based program." | Web-based program. | "Participants were asked to make in-person follow-up visits 6, 12, and 24 months after randomization. At each of these visits, weight was measured on a high-quality, calibrated digital scale, with the participant wearing light, indoor clothes and no shoes." | 6-month follow-up: Control: 113/138*100= 81.9%;  Remote: 129/139*100 = 92.8%;  In-person: 124/138*100 = 89.9%  12-month follow-up: Control: 108/138*100= 78.3%;  Remote: 124/139*100 = 89.2%;  In-person: 123/138*100 = 89.1%  24-month follow-up : Control: 129/138*100= 93.5%;  Remote: 132/139*100 = 95%;  In-person: 133/138*100 = 96.4% |  |
| **10** | **Ard 2018** | LOW | LOW | LOW | LOW |  |
|  | Assessment justification: | The statistician generated blocked random assignments using a computer-based algorithm, stratified by age category (65–74, 75+), sex, and race. | Allocations were concealed in sealed envelopes that were opened by a research assistant at the time of randomization. | Body weight was measured in light clothing on calibrated electronic scales to the nearest 0.1 pound and converted to kilograms. | Less than 50% attrition. |  |
| **11** | **Ash 2006** | LOW | UNCLEAR | LOW | HIGH |  |
|  | Assessment justification: | Randomised by the project manager, using a random number table, into one of three intervention groups at one of two hospital sites. The allocation ratio for the two hospital sites (public and private) was 2:1 due to available resources for implementing the intervention. | NS | Weight objectively measured. | BO = 20/54 complete data 37% IDT = 44/65 complete data 66.7% FBI = 26/57 complete data 45.6% Significant between group difference in drop out and people who dropped out had significantly higher baseline BMI. At 12 months 24 BO, 49 IDT and 29 FBI had weight measurements. |  |
| **12** | **Ashley 2001** | UNCLEAR | UNCLEAR | LOW | HIGH |  |
|  | Assessment justification: | NS | NS | Weight objectively measured.  Certified technicians took blood pressure and body composition measurements. Fasting blood was taken for measuring serum lipids (total cholesterol, low density lipoprotein [LDL] cholesterol, high-density lipoprotein [HDL] cholesterol, and triglycerides), glucose, and insulin by a certified phlebotomist. Blood values were analyzed by standard methods at a statewide, certified clinical laboratory. | 12-months: 74/113 completed all assessments: LOW  24-months:  39/113 completed all assessments: HIGH |  |
| **13** | **Ashley 2007** | UNCLEAR | UNCLEAR | LOW | LOW |  |
|  | Assessment justification: | NS | NS | Weight objectively measured.  Waist circumference was measured at the narrowest point of the torso using a nonstretchable measuring tape. Blood pressure was measured while the subject was seated using a digital manometer machine. | 12-months:  70/96 completed (35 from each group) |  |
| **14** | **Aveyard 2016** | LOW | LOW | LOW | LOW |  |
|  | Assessment justification: | “An independent statistician used Stata Software version 12 to produce a randomisation list that was stratified by physician, with random permuted blocks of four.” | “Randomisation was done via preprepared randomisation cards labelled with a code representing the allocation, which were placed in opaque sealed envelopes and given to physicians to open at the time of treatment assignment.” | Weight objectively measured. | “We weighed 1419 (75%) of participants at the 12-month follow-up.” |  |
| **15** | **Bacon 2002** | UNCLEAR | UNCLEAR | LOW | LOW |  |
|  | Assessment justification: | “To ensure balance in the treatment groups, the enrolled subjects (n = 78) were divided into BMI quartiles, and high/ low sets for dietary restraint, 34 degrees of flexible and rigid control of eating, 35 age, and self-reported activity level. The subjects in these subgroups were then randomly assigned to one of two treatment groups.” | NS | Weight objectively measured.  Blood pressure was assessed in duplicate using the oscillometric technique. Fasting blood samples were analyzed for blood lipids (total cholesterol, low-density lipoprotein [LDL] cholesterol, and high-density lipoprotein [HDL] cholesterol). | 52-weeks:  Diet group: 23/39 completed testing; HAES group: 34 attended (29 completed testing)/36 |  |
| **16** | **Bartels 2015** | LOW | UNCLEAR | LOW | LOW |  |
|  | Assessment justification: | “Randomization between In SHAPE and the comparison condition was stratified by age (21 to 44 years versus 45 years and older) and psychiatric diagnosis (mood disorders versus schizophrenia spectrum disorders). Each combination of stratification categories had its own randomization schedule that was blocked on every fourth assignment to ensure balance between treatment arms. Randomization was conducted sequentially across all sites (not within sites).” | NS | Weight objectively measured.  'Blood pressure was measured before (resting heart rate) and after completing the 6-MWT'  'Lipids were measured using the CardioChek PA Analyzer, a portable testing system that produces reliable values for total cholesterol, LDL, HDL, and triglycerides using a multi-panel test strip and a single drop of blood acquired with a finger prick.' | 18-months:  Control: 83/106 Intervention: 80/104 |  |
| **17** | **Beeken 2017** | LOW | LOW | LOW | LOW |  |
|  | Assessment justification: | “A computer-generated list of random permuted blocks of size 2–4 was used. Randomisation was stratified by PCP to ensure socioeconomic balance between groups.” | “A central telephone-based randomisation service was used to randomise at the level of the patient ensuring allocation concealment” | “All measurements at 3 months were with a health professional blind to group allocation.”  Weight objectively measured. | At 12-months, 61% (Control) and 57% (10TT) were followed up.  "At 24 months, 312 (58.1%) patients were followed up. There remained very little difference in attrition between arms (41.5% in the usual care group vs 42.3% in the 10TT group)." |  |
| **18** | **Bennett 2012** | LOW | UNCLEAR | LOW | LOW |  |
|  | Assessment justification: | Participants were randomized to treatment arm using computer-generated allocations, blocked by clinic and sex. | NS | Weight objectively measured. | 24-months:  Usual care: 166/185 Intervention: 148/180 |  |
| **19** | **Bennett 2013** | LOW | UNCLEAR | LOW | LOW |  |
|  | Assessment justification: | A computer-generated randomization algorithm to allocate participants equally (1:1) across the 2 treatment arms (intervention and usual care); those in the intervention arm were further randomized to 1 of 2 interventionists. | NS | Weight objectively measured.  Secondary measures included waist circumference, blood pressure, and fasting glucose, triglyceride, and cholesterol level. | Usual care: 90/97 Intervention: 86/97 |  |
| **20** | **Berry 2014** | UNCLEAR | LOW | LOW | LOW | HIGH |
|  | Assessment justification: | Cluster randomization. “The sequence of each school was randomized before the start of the study and was stratiﬁed by county. A total of 18 months had passed and the ﬁrst group had completed their time in the study prior to the second enrollment in each school. This design preserved a balance of treatment groups within each site to avoid confounding site effects with intervention effects.” | “Participants and staff were blinded to group assignment from enrollment until implementation.” | Weight objectively measured. | 59% of control group and 57% of intervention group at last follow up.  "To assess the extent of selection bias owing to attrition, the mean values for BMI percentiles were compared between those participants who did not contribute data beyond the Phase I intervention and those who did. There were no signiﬁcant differences between these groups, either overall or by experimental group (P.0.35). " | Wait-list control |
| **21** | **Bertz 2012** | LOW | UNCLEAR | LOW | LOW |  |
|  | Assessment justification: | Random number table | Allocation method not reported but described as ‘concealed’. | Weight objectively measured.  Body composition was measured by using dual-energy X-ray absorptiometry (DXA) (Lunar Prodigy; GE Lunar Corp). Muscle mass was calculated from DXA. | 92% followed up at 12-months, intervention 100%, D 76%, E 83%, control 76%. 4 missing (6%); 2 medical reasons (3%). |  |
| **22** | **Beutel 2006** | UNCLEAR | UNCLEAR | LOW | LOW |  |
|  | Assessment justification: | “These 396 patients were externally randomized (random digits) either to BT or to PD. A minority of patients who were directly referred to a specific setting (usually behavioral) were excluded from randomization.” | NS | Upon intake, BMI was checked based on the current weight and height. Follow-up GPs assessed blood pressure, weight and laboratory data. | Behaviour treatment: 154/175 at approximately 7-weeks;  At 12-months:  97/175  Psychdynamic treatment: 168/179; 97/179 at 1 year. |  |
| **23** | **Bliddal 2011** | UNCLEAR | UNCLEAR | LOW | HIGH |  |
|  | Assessment justification: | "Each randomisation list was drawn up by the study statistician (RC) and given to the secreteriat at the Parker Institute, who subsequently informed patients when to meet the dietician." | "The random allocation sequence was concealed until interventions were assigned: Each randomisation list was drawn up by the study statistician (RC) and given to the secreteriat at the Parker Institute, who subsequently informed patients when to meet the dietician." | Weight objectively measured. | 12-months:  Control 23/44; LED 33/44 |  |
| **24** | **Bo 2007** | LOW | LOW | LOW | LOW |  |
|  | Assessment justification: | “The randomization procedure was automatically performed by a statistician using an SAS program developed to minimize the differences between the two groups for all stratifying variables. The patients were randomly allocated to receive either standard lifestyle recommendations from their physicians (control group, n=188) or a structured lifestyle intervention program for 1 year carried out by health professionals (intervention group, n=187).” | “Random allocation with a minimization algorithm was centrally performed in a single step. The researchers then received the two lists of nominative data. The possibility for researchers to predict or influence the allocation of participants was thus completely prevented.” | Weight, waist circumference, and blood pressure were measured. Fasting glucose, insulin, triglycerides, high-density lipoprotein (HDL) cholesterol, uric acid, and hs-CRP values were measured before and after the study in both groups. | 12-months:  Control: 166/188 Intervention: 169/187 |  |
| **25** | **Brown 2014** | LOW | UNCLEAR | LOW | LOW |  |
|  | Assessment justification: | Randomised block design. Computer-generated random assignment was used to assign an equal number of individuals from each risk group to the weight loss program (RENEW) or to a control group. | NS | Weight objectively measured. | 136 at baseline. 92 at 12 months (47 intervention; 45 control) |  |
| **26** | **Burke 2015** | LOW | UNCLEAR | LOW | LOW |  |
|  | Assessment justification: | Randomization used the minimization method. Treatment assignments were determined considering gender and ethnicity (White vs. non-White) to ensure balance across the treatment groups. | NS | "Data were collected at the research center by trained staff using standardized procedures and questionnaires. Equipment was standardized and routinely calibrated" | 79.2% in SBT arm and 81% in SBT+SE arm completed 18-month assessment. |  |
| **27** | **Chee 2017** | LOW | UNCLEAR | LOW | LOW |  |
|  | Assessment justification: | Patients were randomized using a random allocation software. | NS | Weight objectively measured. | UC:  3mth: 105/115;  6mth: 101/115;  9mth: 99/115;  12mth 98/115  tDNA-CC:  3mth: 48/57;  6mth: 40/57;  9mth: 40/57;  12mth: 40/57  tDNA-MI:  3mth: 51/58;  6mth: 51/58;  9mth: 51/58;  12mth: 51/58. |  |
| **28** | **Cheyette 2007** | UNCLEAR | UNCLEAR | LOW | LOW |  |
|  | Assessment justification: | "Non probability volunteer sampling was used to assign people to either the intervention or the control group." | NS | Weight objectively measured. | " At six and 12 months follow up a total of eight people dropped out from the intervention group and two from the control group." |  |
| **29** | **Christensen 2012** | LOW | LOW | LOW | LOW | LOW |
|  | Assessment justification: | Cluster-randomization procedure. A cluster formation of the groups was performed to assure equal allocation in the intervention and reference groups balanced on sex, age, job seniority or job type with cluster size varying from 3 to 15. | The randomization was done by an external research group, which had no knowledge of the work place or the participants. Clusters were randomly allocated to intervention and control by the drawing of sealed envelopes from a bag. | The test manager was blinded regarding the participants intervention status, and whenever possible the same test manager tested the subject at all three rounds of tests | 98 participants --> 83 participants. | Clusters were created based on information from the screening questionnaire and the management of working teams, day and evening/night shifts and close working relations. This approach was chosen to avoid contamination, and to benefit from the social support in work teams, thereby in- creasing compliance. |
| **30** | **Cleo 2018** | LOW | UNCLEAR | LOW | LOW |  |
|  | Assessment justification: | “Computer-generated randomization occurred after baseline assessment to allocate participants to either: TTT, DSD, or WL control (allocation ratio 1:1:1). We used minimization stratiﬁed on BMI categories (overweight, obese class I, II, III); age (18–32, 33–47, 48–62, 63–75 years); and gender.” | NS | Weight objectively measured. | At 12-months:  21/25 (84%) 22/25 (88%) |  |
| **31** | **Cole 2013** | LOW | LOW | LOW | UNCLEAR |  |
|  | Assessment justification: | “Randomization occurred by a computer-generated random-numbers list (SPSS version 15.0.1; IBM Corporation) with assignments placed in sealed envelopes, numbered sequentially, and allocated to participants in the order of recruitment.” | Sealed envelopes used. | Weight objectively measured. | “…94 were randomized into the 2 study groups, 80% remained at 3 months, and 69% completed the 1-year assessment (n = 34 SMA, n = 31 control, n = 29 lost to follow-up).”  “Limitations of the study resulted from a high attrition (31%).”  Data only given at all points for those who completed year 1 – loss to follow-up from each group is unclear. |  |
| **32** | **Conroy 2015** | UNCLEAR | LOW | UNCLEAR | LOW |  |
|  | Assessment justification: | “…randomization occurred in a 1:1 allocation. Each woman was allowed to draw a sealed envelope that contained a designation assignment, either interventionist-led (IL) or self-guided (SG).” | Sealed envelopes. | Weight was measured by a trained staff member in clinic using a standard balance beam scale (SECA Medichoice) and following a written protocol.  " For the 12-month followup, 62 (74 %) of 84 participating women had an in-person assessment (with study-measured weight), with the remainder of the outcomes assessed by phone." Breakdown by group not clear | “Follow-up was better in the IL group (90 % at 3 months and 96 % at 12 months) than in the SG group (63 % at 3 months and 76 % at 12 months), but otherwise did not differ by other participant characteristics.” |  |
| **33** | **Cooper 2010** | LOW | LOW | LOW | LOW |  |
|  | Assessment justification: | “Participants were allocated to the three treatments by HAD (who had no involvement in participant recruitment) using a stratified computer-generated randomization scheme with random permuted blocks of varying size within two strata. Participants were assigned to the two strata on the basis of their binge eating frequency with those reporting 12 or more episodes over the previous 12 weeks being classed as belonging to a binge eating subgroup.” | “The allocation sequence was concealed in numbered sealed opaque envelopes. At the point of randomization, the next envelope in the sequence was opened by one of the two senior clinicians.” | Weight objectively measured. | At 36-months the following completed assessment:  44/51 GSH  44/50 BT  46/49 CBT |  |
| **34** | **Cousins 1992** | UNCLEAR | UNCLEAR | LOW | LOW |  |
|  | Assessment justification: | “Subjects were stratified according to weight and randomly assigned to one of the three treatment groups.” No further information given | NS | Weight and height measured "using a standard physician's scale." | Total at start 168.  82 excluded because of missing data. 86 completed >50% LOW The remaining 82 subjects were excluded because of missing data at any of the 3-, 6-, and 12-month measurement sessions. Preliminary ANOVA revealed no significant differences on any of the baseline measures, including initial BMI, initial weight, age, acculturation, years of education, or income between the 86 subjects included in these analyses and the 82 who were excluded. |  |
| **35** | **Craighead 1989** | UNCLEAR | UNCLEAR | LOW | LOW |  |
|  | Assessment justification: | “The subjects who met the qualifications for the study were rank-ordered according to pounds overweight and randomly assigned within blocks of three to one of the experimental conditions. Two groups were formed within each condition depending on subject's availability for meeting times.” | NS | 'subjects were weighed' at baseline, at each session, at end of 12 weeks and at 1 year. | 62 recruited, 42 at 12 weeks, 38 at 1 year 14 dropped out. 'The dropout rate was not significantly different among the groups' The treatment analyses were conducted on the 42 subjects who fully participated in the treatment condition to which they had been assigned. |  |
| **36** | **Crowley 2017** | LOW | LOW | LOW | LOW | HIGH |
|  | Assessment justification: | “Eligible participants were randomized using a computerized random number generator in blocks of 2 (study personnel other than statisticians blinded to block size) within strata defined by baseline HbA1c level (7.5%-8.9% vs ≥9%) and insulin use (multiple types vs 1 type or none).” | After a patient’s screening information has been reviewed and found to meet eligibility criteria, the study coordinator will access a computer program in which to enter the values of the stratification variables; in turn, the computer program will provide the participant’s randomly assigned study arm: WM/SMA or SMA. | Body weight was measured at every visit using a standardized digital scale. | 222/263 at 16-weeks; 198/263 at 32-weeks; 209/263 at 48-weeks  GMV:  117/136 at 48-weeks.  WM:  109/127 at 48-weeks | Baseline data for Systolic BP, total cholesterol and HDL-C is identical for both study groups. Emailed author to query whether these measures were taken before groups were randomised as this is unclear. |
| **37** | **Dale 2009** | UNCLEAR | UNCLEAR | LOW | LOW |  |
|  | Assessment justification: | NS | NS | Weight objectively measured.  “At the time of the euglycemic insulin clamp study, fasting blood samples were taken for lipid measurements, and anthropometry and blood pressure measurements were repeated.” | 87% followed up at 12 months (87% MI, 92% II, 87% control). Reasons for attrition not reported. Reviewers assumed equal loss to follow-up between intervention arms. |  |
| **38** | **Dalziel 2006** | UNCLEAR | UNCLEAR | UNCLEAR | LOW | HIGH |
|  | Assessment justification: | NS | NS | NS | "Shortly after randomisation, 21 (8 in the controls and 13 in the experimental group refused follow-up) (table 1)." The mean rate of withdrawal from follow-up was similar in the experimental (8%) and control (7%) groups. | Did not explicitly aim for weight loss so may introduce clinical heterogeneity into the review. Included after discussion as dietary intervention versus control. |
| **39** | **Damschroder 2014** | LOW | UNCLEAR | LOW | LOW |  |
|  | Assessment justification: | “…a biostatistician provided block randomized assignments (by medical center and two BMI categories [o35 or Z35] to ensure balance between groups) using random permutated blocks constructed by Stata’s *ralloc* command; block sizes ranged from 3 to 9…” | “Investigators were blind to assignments until baseline assessments were complete.” | Anthropometric measures (height, weight, and waist circumference); blood pressure; and self-reported measures including a Food Frequency Questionnaire; EuroQoL-5D utility assessment (with level of painsubscale); Satisfaction with Life Scale; demographic characteristics; laboratory testing for cholesterol and glucose metabolism; and a 6-minute walk test were collected in baseline, 3-month, and 12-month assessments. | Move:  3mth: 115/159;  12mth: 119/159  Aspire phone:  3mth: 131/162;  12mth: 120/162  Aspire group:  3mth: 127/160;  12mth: 122/160;  Follow up 332/481 consented to long term follow up. Move:  18mth: 92/112;  24mth: 90/112  Aspire phone:  18mth: 95/105;  24mth: 92/105  Aspire group:  18mth: 102/115;  24mth: 104/115 |  |
| **40** | **Daubenmier 2016** | LOW | LOW | LOW | LOW |  |
|  | Assessment justification: | "A computer-generated random allocation sequence using random block sizes of four to eight was programmed by a database manager not involved in enrollment." | "No other staff had access to the randomization sequence. The project director (PM) accessed the allocation sequence using a programmed database that could not be altered once randomized condition was revealed." | "Weight was measured to the nearest 0.1 kg on a calibrated digital scale (Wheelchair Scale 6002, Scale-Tronix, Carol Stream IL), with participants wearing a hospital gown. The same scale was used for measurements throughout the study." | At 18-months follow-up 81% of participants from the mindfulness group and 71% from the control group were followed up. |  |
| **41** | **Delahanty 2015** | UNCLEAR | UNCLEAR | HIGH | LOW |  |
|  | Assessment justification: | NS | NS | "Participants’ height and weight were measured twice and averaged on a stadiometer (baseline only) and digital scale, respectively."  "To evaluate sustainability of weight loss, clinically obtained weights were abstracted from medical records, if available, 1 year after randomization date with a 10- to 14-month window." | "95% retention at 6 months." At 12 months, 2 GLI and 5 MNT participants had missing clinical data. |  |
| **42** | **deRoon 2017** | LOW | UNCLEAR | HIGH | LOW |  |
|  | Assessment justification: | “After baseline measurements, women were stratified for municipality randomized by computer.” | NS | “At baseline and end of study body weight was measured using an identical balance scale, but at follow-up, body weight was self-reported by the participants.” | Anthropometrics:  At 16-weeks:  control 45/48;  diet 94/97;  exercise 93/98  At 12-months:  control: NS;  diet: 78/97; exercise: 77/98. |  |
| **43** | **deVos 2016** | UNCLEAR | LOW | LOW | LOW | LOW |
|  | Assessment justification: | "...subjects were randomized using consecutive case numbers. For the diet-and exercise program, subjects were randomized 1:1 using block randomization with block size 20." | "A research assistant not involved in the trial provided a sealed envelope that was opened by the subject in the presence of the researcher." | "For the first 2.5 y, all participants were home-visited every 6 mo by a research assistant..." "Body weight was also measured during these visits." "After 6.6 y, participants were visited once more for measurements and a questionnaire." | After 2.5 y, 10.1% of the participants were lost to follow-up. After 6.6 y, 247 participants (60.7%) agreed to additional measurements and questions. "No significant difference in attrition rate was found between the randomly assigned groups." | Original study design included 4 groups ((1) Lifestyle intervention plus placebo; (2) Lifestyle intervention plus Glucosamine; (3) Control plus placebo; (4) Control plus Glucosamine) which were combined into two groups. "The preventive effects of a weight-loss program and of oral glucosamine sulfate compared with placebo on the incidence of knee osteoarthritis were investigated in a 2x2 factorial design with a follow-up time of 6.6 y." No effects of glucosamine on these outcomes were expected or detected. Therefore, the glucosamine intervention will be disregarded in the present manuscript." |
| **44** | **Diabetes Prevention Program R G 2009** | LOW | LOW | LOW | LOW | HIGH |
|  | Assessment justification: | “The randomization was done centrally by computer…”  Random treatment assignments were stratified according to clinical center and were generated by the coordinating center through computer linkup to the field center at time of randomization. Therefore, assignment was unknown until randomization. Assignments to metformin and placebo were double-blinded. | “…assignments to the lifestyle group were blinded until randomization, while assignments to the medication groups were blinded until the end of the study.” | Lifestyle intervention participants were weighed privately at the start of every individual session and were encouraged to weigh themselves at home daily or a minimum of once per week. | Placebo yr. 1 - 1027/1082;  yr. 2 - 1015/1082;  yr. 3 - 975/1082.  Bridge period DPPOS - 1085 eligible, 935 enrolled. DPPOS  yr. 1 882/935;  yr. 2 874/935;  yr. 3 844/935;  yr. 4 - 827/935;  yr. 5 - 846/935;  yr. 6 808/935;  yr. 7 - 789/935;  yr. 8 766/935;  yr 9. 760/935;  yr. 10 - 763/935;  yr. 11- 769/935.  Lifestyle  yr. 1 - 1026/1079;  yr. 2 - 1001/1079;  yr. 3 - 972/1079.  Bridge period DPPOS - 1068 eligible, 914 enrolled. DPPOS  yr. 1 855/914;  yr. 2 827/914;  yr. 3 816/914; yr. 4 - 810/914;  yr. 5 - 824/914;  yr. 6 783/914;  yr. 7 - 763/914;  yr. 8 757/914;  yr. 9 738/914;  yr. 10 - 725/914; yr. 11.- 738/914. | 0-3 years LOW  From year 4 HIGH DPP was a 3-year randomized clinical trial followed by open-label modified intervention follow-up. |
| **45** | **Djuric 2002** | UNCLEAR | UNCLEAR | HIGH | LOW | HIGH |
|  | Assessment justification: | 48 patients were randomly assigned into four research groups by random block design. At baseline: “There were no differences among the four groups in body weight and BMI. Nevertheless, there were significant differences in percentage body fat, total cholesterol, and LDL-C at baseline, indicating that the block randomization process did not equalize all parameters among groups. However, the highest values of these parameters were not consistently found in any one group.” | NS | 'Weighed in clothing but without shoes using a professional beam scale (model 402KLS; Health-o-Meter, Bridgeview, IL), and percentage of body fat was measured using tetrapolar bioelectrical impedance (model BIA101S; RJL Systems, Clinton Township, MI). Height was measured at baseline only.' | 18.75% dropped out by end of study.  At 12-months:  Control: 12/13  WW: 8/11  Individualised: 9/13  Comprehensive: 10/11 | Missing outcome data - study states intention to follow up to 30 months but 30-month data not available. Data for 3 and 6 months extracted from graphs but some inconsistency between graphs and what is reported in text. |
| **46** | **Duncan 2016** | LOW | LOW | LOW | HIGH |  |
|  | Assessment justification: | “…participants were randomized into one of two groups using a simple randomization procedure stratified by clinic with a 1:1 allocation ratio.”  “…the order of control and intervention envelopes was distributed at each practice using a computer-generated randomization list.” “Participants were randomized within practices such that some within a practice were assigned to treatment and some to control conditions. Participating practices were not used as the unit of randomization to avoid between-practice effects confounding between-group differences.” | “Practice nurses and physicians were blinded to the designation of the envelopes…” (notifying participants of allocation to either control or intervention) | Weight objectively measured. | While participant drop-out from baseline to 4 months was 49%, the lack of between-group differences in baseline demographic and health indicators in individuals that dropped out of the study indicates that systematic bias was not introduced.  "Of the 320 participants randomly assigned to control and intervention groups, 156 (48.8%) were followed-up at 4 months, with 157 (49.1%) at 12 months." |  |
| **47** | **Eaton 2016** | LOW | LOW | LOW | LOW |  |
|  | Assessment justification: | “After the baseline visit was completed, participants were block randomized within practice in pairs using a random number generator created by the data manager with SPSS for Windows, version 11.0 (IBM).” | “After completion of the initial lifestyle counseling session, the research assistant gave each participant an envelope that revealed the study arm to which the participant was assigned.” | Height, weight, waist circumference, resting heart rate, and resting blood pressure were measured at each visit. | Control:  78/106 at 6mths; 75/106 at 12mths; 77/106 at 18mths; 75/106 at 24mths Enhanced intervention: 88/105 at 6mths; 84/105 at 12mths; 75/105 at 18mths; 73/105 at 24mths |  |
| **48** | **Fahey 2018** | LOW | LOW | HIGH | HIGH |  |
|  | Assessment justification: | Participants were individually randomly assigned, using a computerized block design (six blocks of four), to one of the two intervention conditions (1:1 allocation) with allocation concealment to ensure balanced assignment to both conditions throughout the study duration. | Refer to ‘Random sequence generation (selection bias)’. | All measures were obtained by unblinded data collectors at baseline, 4 months, and 12 months unless otherwise indicated. | We had originally planned to recruit 204 participants; however, because of greater than expected attrition, we increased the sample size to 248 in order to retain power to detect our planned effect. To avoid introducing bias associated with attrition because of failure to lose weight, missing weight values were imputed conservatively using baseline observation carried forward.   Retention rate n (%): 4-months:  CI: 109/124*100 = 87.9%;  SP: 90/124*100 = 72.6% 12-months:  CI: 95/124*100 = 76.6%;  SP: 77/124*100 = 62.1% |  |
| **49** | **Fernandez-Ruiz 2018** | UNCLEAR | UNCLEAR | LOW | LOW |  |
|  | Assessment justification: | Randomisation was performed using a simple table of numbers: 37 patients in the control group and 37 in the experimental group.  “A random allocation sequence was generated by a member of the scientific staff through extraction of successive numbered balls from an opaque container, alternating between the experimental and the control group' | Refer to ‘Random sequence generation (selection bias)’. | “The efficacy of the intervention was evaluated through anthropometric (body mass index, weight, different parameters, and skinfolds, as stated in Section 2) and cardiovascular measures taken before, during, and after intervention.' 'Anthropometric and cardiovascular measures were taken at the pretest stage, every 6 months during the programme, and 1 year after it finished.” | No loss to follow up reported. |  |
| **50** | **Finkelstein 2017** | LOW | LOW | LOW | LOW |  |
|  | Assessment justification: | “…a computer generated assignment schedule prepared by the statistician. Participants were randomized in a ratio of 1:2 (control: reward) using a block size of 6, with stratification based on gender, ethnicity (Chinese, Non-Chinese), and BMI (<32.5 kg/m2 , 32.5 kg/m2). Randomization was stratified by BMI to ensure that differences between treatment arms could not potentially be driven by differences in proportion of very high vs. moderate/high BMI.” | “Randomization envelopes containing a slip of paper indicating the study arm were prepared by research staff not involved in random allocation. The envelopes were arranged in sequential order for each stratum, and the top-most envelope was picked by the study coordinator based on the specific stratum to which the participant belonged.” | Weight measured objectively. | A total of 123 (76.4%) participants completed the month 12 assessment. 13 participants were lost to follow up at month 4; 5 (9.3%) in the control arm and 8 (7.5%) in the reward arm. At month 8, 35 participants were lost to follow-up; 16 (29.6%) in the control arm and 19 (17.8%) in the reward arm. At month 12, 38 participants were lost to follow-up; 17 (31.5%) in the control arm and 21 (19.6%) in the reward arm. |  |
| **51** | **Fisher 2011** | UNCLEAR | UNCLEAR | LOW | UNCLEAR | UNCLEAR |
|  | Assessment justification: | NS | NS | Weight objectively measured.   Total and regional body composition, including total fat mass, percent body fat, leg fat mass, and lean body mass were measured by dual-energy X-ray absorptiometry | NS | The weight loss programme varied in length based on when weight loss target was achieved. However, weight taken when weight loss target achieved, at approximately 6mths.  Subjects were evaluated in the overweight state (prior to any intervention). Weight was stabilized for 4 weeks through dietary control. All testing was conducted following the weight stabilization period, and in the follicular phase of the menstrual cycle. During the weight stabilization period, body weights were measured three to five times per week. Fisher et al. Page 2 Obesity (Silver Spring). Author manuscript; available in PMC 2011 June 1 NIH-PA Author Manuscript NIH-PA Author Manuscript NIH-PA Author Manuscript General Clinical Research Center (GCRC) at UAB. A macronutrient-controlled diet was provided during the final 2 weeks of weight maintenance. |
| **52** | **Foley 2016** | LOW | UNCLEAR | LOW | LOW |  |
|  | Assessment justification: | “Randomization occurred at the baseline visit, using a computer-based algorithm. The randomization algorithm allocated participants equally (1:1) across treatment arms, after accounting for CHC, gender and ethnicity (Hispanic vs. non-Hispanic) in order to ensure the equal representation of these characteristics across arms.” | NS | Weight measured objectively. | Less than 50% attrition at 12-month follow-up. |  |
| **53** | **Foreyt 1993** | LOW | UNCLEAR | LOW | LOW |  |
|  | Assessment justification: | Random number table | NS | Weight measured objectively. | 86/127 at 12mths (in 3 intervention groups (not control)) LOW 61/127 at 2 years HIGH (61/86 that completed to 12mths)  In each treatment condition, about one third of subjects dropped out of the study before the one-year assessment; 13 from the diet-only, 13 from the exercise-only, and 15 from the exercise-plus-diet groups. The differential dropout rate across treatment groups was not significant, ~z = .34, p = .84. At baseline, dropouts were not significantly heavier than those who were available for one-year follow-up, 103.3 kg versus 96.3 kg, respectively, F(1,121) = 2.49, p = .12. |  |
| **54** | **Foster-Schubert 2012** | LOW | LOW | LOW | LOW | UNCLEAR |
|  | Assessment justification: | “The random assignment was generated by a computerized program, stratified according to BMI (<30 kg/m2 or ≥30 kg/m2) and participants’ self-reported race/ethnicity (non-Hispanic white, black, or other). In addition, to achieve a proportionally smaller number of women assigned to the control group, a permuted blocks randomization with blocks of four was used, wherein the control assignment was randomly eliminated from each block with a probability of ~1 in 4.” Blocked-randomisation. (Permuted-block randomization (ratio 0.75 : 1 : 1 : 1) to assign a proportionally smaller number of women to the control group.) | Central computerised allocation. | Weight measured objectively. | 91% followed up at 12m overall: 92% D+E, 89% D only, 91% E only, 92% usual care. 2 unavoidable losses (<1%); 8% missing; 1% medical reason. | Control group received intervention at 12m, unclear if they knew in advance. |
| **55** | **Freitas 2017** | LOW | LOW | HIGH | UNCLEAR |  |
|  | Assessment justification: | "Randomization schedule was computer-generated and implemented by an investigator blinded to the recruitment, evaluation and treatment of the participants." | " Each patient’s allocation was concealed using sequential numbering and then sealed and placed in opaque envelopes..." | "The nutritionist and psychologist, as well as the outcome assessors, were blinded throughout the duration of the study."  "The long-term effect (6 and 12 mo after randomization) was evaluated by obtaining body weight from patients’ medical records." | No information given on n followed up at 6- and 12-months. |  |
| **56** | **Fuller 2012** | UNCLEAR | UNCLEAR | LOW | LOW |  |
|  | Assessment justification: | The randomisation process was completed by the study database (Filemaker Pro), upon entry of the participant’s initials and fulfilment of trial requirements. | NS | Weight objectively measured. | 22/35 Korean group and 28/35 Western group completed the study. |  |
| **57** | **Gold 2007** | UNCLEAR | UNCLEAR | LOW | LOW |  |
|  |  | NS | NS | Weight was objectively measured. | 71% followed up at 12m; 65% intervention, 77% control. 2% unavoidable; 25% missing; 2% medical. |  |
| **58** | **Goodwin 2014** | LOW | UNCLEAR | LOW | LOW |  |
|  | Assessment justification: | "Random assignment was performed centrally by the Ontario Clinical Oncology Group, and a computer-generated block randomization scheme with blocks of various size was used." | NS | Weight objectively measured. | "Six patients in the mail-based intervention and seven in the LI arm did not complete the 24-month intervention period because of a primary outcome event (new disease, metastases, death); of the remaining patients, 14 (8.7%) and 16 (9.9%), respectively, withdrew (including patients who transferred care, those who were lost to follow-up, or those with noncompliance). Month-24 weight measurements were available from 264 (90.1%) of 293 participants still on the study." |  |
| **59** | **Hakala 1993** | UNCLEAR | UNCLEAR | LOW | LOW |  |
|  | Assessment justification: | 'randomly divided' | NS | Weight objectively measured. | Group 28/30 at 2 yrs., 28/30 at 5yrs. Individual 27/28 at 2 yrs., 25/28 at 5yrs.  'Adherence 97% at 2 yrs., 88% at 5 years' |  |
| **60** | **Hanson 1976** | UNCLEAR | UNCLEAR | UNCLEAR | HIGH |  |
|  | Assessment justification: | NS | NS | NS | Attrition greater than 25% at 5-month follow-up and approximately 50% at 1-year follow-up. |  |
| **61** | **Harrigan 2016** | LOW | LOW | HIGH | HIGH |  |
|  | Assessment justification: | "Permuted-block randomization with random block size was performed by the study biostatistician" | "...blinded study staff using unmarked envelopes." | "Height (using a stadiometer) and weight were measured at baseline and 6 months." "... self-reported weight from baseline to 12 month" | Because there were 15 (15%) individuals who were missing body weight measurements at 6 months, multiple imputation with data augmentation under the multivariate normal model was conducted using SAS PROC MI, as described by Allison.15 The final results were consistent with the results without multiple imputations.  Completed 12-Months: Usual care: N = 19 (58%);  Telephone: N = 15 (44%);  In-Person: N = 22 (67%) |  |
| **62** | **Hunt 2014** | LOW | LOW | LOW | LOW | HIGH |
|  | Assessment justification: | “After baseline measurement, the randomisation sequence was generated by the Tayside Clinical Trials Unit (TCTU) statistician (with no day to day role in the study at this point) with SAS (version 9.2), blocked (block size between two and nine dependent on how many participants were recruited at a club), and stratified by club.” | “The allocation sequence was sent in a password protected file to a database manager (not part of the research team) who assigned individuals to each group.” | Weight measured objectively. | Comparison (control): 347/374 at 12-weeks; 355/374 at 12-months. Intervention:  330/374 at 12-weeks; 333/374 at 12-months | 12 month wait-list control. |
| **63** | **Huseinovic 2016** | LOW | LOW | LOW | LOW |  |
|  | Assessment justification: | "...simple randomization procedure that used numbered and sealed envelopes generated through a random number table prepared by the project coordinator." | Refer to ‘Random sequence generation (selection bias)’. | "All study measures and administration of intervention were completed by 2 dietitians at the primary health care clinics. Blinding of the study dietitians was not possible..." | D Group:  Baseline n=54;  12-wk: n=47;  1-year: n=44 C Group:  Baseline n=56;  12-wk n=53;  1-year: n=45 |  |
| **64** | **Irwin 2003** | LOW | LOW | LOW | LOW |  |
|  | Assessment justification: | “Randomization was performed by random number generation…”  “Randomization was stratified by BMI (<27.5 vs >27.5) to ensure equal numbers of heavier and lighter women in each study group.” | “…group assignment was placed in a sealed envelope…” | Weight measured objectively. | Less than 50% attrition at 12-months follow-up. |  |
| **65** | **Jakicic 2011** | LOW | UNCLEAR | LOW | LOW |  |
|  | Assessment justification: | "Randomization was performed by the study statistician using a computer program with randomization blocked by gender." | NS | "Body weight was assessed using a calibrated balance-beam scale to the nearest 0.1kg (0.25 pounds) with the subject clothed in a cloth hospital gown." | "There was no significant difference in attrition rates between groups based on χ2 analysis."  At the 18-month assessment 82.1% from Self-help group, 72.4% from the Moderate PA group and 81.8% from the High PA group contributed to assessment data. |  |
| **66** | **Jakicic 2015** | LOW | LOW | LOW | LOW |  |
|  | Assessment justification: | "The randomization sequence was generated by the study biostatistician (W.L.). Randomization was stratified based on gender (male or female), using a computer generated allocation, and only occurred after the participant had successfully completed baseline assessments." | Computer generated allocation. | Weight measured objectively. | Retention rate:  Intervention end: SBWP: 97.1%;  ADOPT: 90.1%  6-Month:  SBWP: 78.9%;  ADOPT: 73.2%  12-Month:  SBWP: 60.6%;  ADOPT: 62%  18-Month:  SBWP: 66.2%;  ADOPT: 69% |  |
| **67** | **Jebb 2011** | LOW | LOW | LOW | LOW |  |
|  |  | "The randomisation sequence was computer generated with Stata (version 9.0) by APM and built into the database by the data manager, who was independent from the study team, and was stratified by country, sex, and diabetes status, with an upper limit of 50% of participants with diabetes." | "Treatment allocation was concealed by use of an online database (Filemaker Pro 9, version 3)." | "In the UK and Australia, bodyweight (in light clothes without shoes) and fat mass were measured with a Tanita BC-418 segmental body composition analyser (Tanita Corporation of America, Arlington Heights, IL, USA). In Germany, weight was measured in GP practices with standard scales, and fat mass was measured at the research centre with the Tanita BC-418."  "All participants who did not complete the 24-month visit but had not formally withdrawn from the study were asked to provide self-reported weights in a telephone follow-up survey (Australia and the UK) or a postal survey (Germany)." Majority was objectively reported. | 12-month retention rate: Standard care: 214/395*100 = 54.2% Commercial programme: 230/377*100 = 61%  18-month retention rate:  Standard care: 115/395*100 = 29.1% Commercial programme: 121/377*100 =32.1%  24-month retention rate: Standard care: 98/395*100 = 24.8% Commercial programme: 105/377*100 = 27.9% |  |
| **68** | **Jebb 2017** | LOW | LOW | LOW | LOW |  |
|  | Assessment justification: | "An independent statistician produced a computer generated randomisation list with 1:1 allocation using stratified block randomisation" | "After the nurse had confirmed eligibility, participants were enrolled in the study and the allocation was revealed using an online randomisation programme to ensure full allocation concealment. " | Weight was objectively measured. | 95/140 UC and 104/138 TDR followed up at 12 months. |  |
| **69** | **Jeffery 2003** | UNCLEAR | UNCLEAR | LOW | LOW |  |
|  | Assessment justification: | "randomly assigned" | NS | Weight measured objectively. | "Retention of study participants was good over 18 mo of followup in both study conditions. Expressed as a percentage of randomly assigned subjects who returned for follow-up visits, retention rates at 6, 12, and 18 mo of the study were 90%, 82% and 87% in the SBT group and 94%, 79% and 80% in the HPA treatment group, respectively." " At 30 mo, retention of study participants as a proportion of those randomly assigned was 79% (74 of 93) in the SBT group and 77% (84 of 109) in the HPA group.” |  |
| **70** | **Jolly 2011** | LOW | LOW | HIGH | LOW |  |
|  | Assessment justification: | "The randomisation sequence was prepared by an independent statistician..." | "An independent statistician prepared two separate randomisation sequences, and, to ensure blinding, the allocations were placed in opaque, consecutively numbered envelopes, which the call centre staff used in order. The block sizes were determined to achieve one to one randomisation across groups, except for the two primary care arms, for which spaces were limited and allocation was in a ratio of 1 to 0.7 compared with the other groups." | "When participants attend their first weight-loss session in the six interventions, the leader/counsellor measures participants’ height and weight. Scales are validated by the research team using standardised weights, unless evidence of recent independent validation is provided. The commercial providers often use self-reported height, so this will be re-measured at follow-up by the blinded assessor. People in the comparator control group and people who are randomised but who do not attend their allocated programme are contacted and a researcher makes an appointment to measure height and weight. During the 12-week programmes the service providers record weights on each visit. The comparator group are weighed at baseline only."  "At three months after programme start (programme end) the service providers weigh participants. Participants who are no longer attending their allocated programme are contacted and offered follow-up at home or another convenient location. If participants decline to be followed-up in person, they are asked to provide a self-reported weight, which is recorded as self-report. "  Flow of participants through trial figure highlights that over a third of weight measures were self-reported. | "At programme end, 658 (88.9%) participants were followed up; 522 (70.5%) were followed up at one year (fig 1)." Attrition rate at intervention end (3 months):  Weight watches: 95%; slimming world: 93%; Rosemary Conley: 88%; NHS Size down: 87%; GP: 82.6%;  Pharmacy: 82.3%; Choice: 95%; Comparator: 83% Weight watches: 82%; slimming world: 62%; Rosemary Conley: 74%; NHS Size down: 66%; GP: 65.7%;  Pharmacy: 58.6%; Choice: 79%; Comparator: 72% |  |
| **71** | **Jones 1999** | UNCLEAR | UNCLEAR | UNCLEAR | LOW |  |
|  | Assessment justification: | "Patients were randomized in a single blind fashion to either the weight loss intervention group or the control group. Randomization was done in a blocked fashion to ensure that equal numbers of the three HOT treatment groups were in both the weight loss intervention group and the control group." | NS | "Weights for both groups were measured at 6-month intervals during follow-up required by the HOT protocol." | "Four patients in the weight loss group and five patients in the control group did not complete the study and were excluded from the data analysis." |  |
| **72** | **Katula 2013** | LOW | LOW | LOW | LOW |  |
|  | Assessment justification: | "Eligible participants were randomly assigned, if equal probability, to either the lifestyle intervention or the enhanced usual care arm using a web-based data management system that verifies eligibility." | Refer to ‘Random sequence generation (selection bias)’. | "Assessments are performed at 6 month intervals (baseline, 6-, 12-, 18- and 24-months post-randomization) at the GCRC. Psychosocial measures are self-administered and remaining measures are completed by trained study staff or clinic staff." | 6-month assessment visit: UC: 141 attended (94%); LWL: 139 attended (92%); 12-month assessment visit: UC: 138 attended (92%); LWL: 135 attended (89%); 18-month assessment visit: UC: 132 attended (88%); LWL: 125 attended (83%); 24-month assessment visit: UC: 134 attended (89%)L LWL: 127 attended (84%) |  |
| **73** | **Keogh 2014** | LOW | LOW | LOW | HIGH |  |
|  | Assessment justification: | “Participants were divided into two groups according to age and BMI and allocated 1:1 to treatments using computerized random number generation.” “The two groups were randomized using Microsoft Excel random number after being blocked according to age and BMI.” Participants will be randomized using the minimization method - (Trial registration ACTRN12612000197831) | Sealed envelopes containing the diet allocation will be used (Trial registration ACTRN12612000197831) | Height and weight were measured at the initial visit and BMI was calculated (weight [kg]/height [m2]).  Weight was measured at all visits (2 weeks apart for 8 weeks and at 12 month).  Subjects were asked to remove shoes prior to both measurements. Although subjects were not given pre-measurement instructions regarding fluid and food intake, effort was made to measure weight at approximately the same time each visit. | “…high drop-out rate experienced early in the study may have limited our ability to detect a statistical difference. Forty percent of the drop-outs occurred between baseline and week 8, thereafter only 8 of 44, i.e. 20% which is the usual attrition seen in such studies.” Intermittent dieting 19/39  continuous dieting 17/36 |  |
| **74** | **Keranen 2009** | LOW | LOW | LOW | LOW |  |
|  | Assessment justification: | Simple randomization without blocking (Fig. 2). Random sampling numbers | “The random sampling numbers were unknown to any of the investigators, study nurses or nutritionists and were contained in a set of sealed envelopes, each bearing on the outside only the number. After acceptance of a patient by the physician, the appropriate numbered envelope was opened by the study nurse.” | Weight measured objectively. | Short counseling: 36/47 received intervention; 29/47 at follow up LOW Intensive counseling: 26/35 received intervention; 20/35 at follow up LOW |  |
| **75** | **King 1989** | LOW | LOW | LOW | LOW | HIGH |
|  | Assessment justification: | The subjects were randomly assigned within each cohort by selecting one envelope for a set of sealed envelopes. | Sealed envelopes. | "Subjects' height and weight were measured using a balance-beam scale, with subjects wearing normal indoor clothing (without shoes). Subjects were weighed at the start and at months 6 and 12 of year 2 by staff members blind to each subject's year-2 condition assignment." | "Of the 51 subjects initially randomized to weight loss through energy restriction during year 1, 44 (86.3%) participated in the year-2 maintenance study. Of the 52 subjects initially randomized to weight loss through exercise during year 1,46 (88.5%) participated in the year-2 maintenance study."  "Of the 90 subjects participating in the maintenance study, complete year-2 total body weight data were obtained for 36 (81.8%) of 44 dieters and 36 (78.3%) of 46 exercisers." | The study randomised to three groups 1. Diet, 2. exercise or 3. control. At the end of the 1-year intervention, participants in the diet group and exercise group were re-randomised within each condition to either a maintenance condition or control condition, forming four groups for the follow-up period (the original control group was not followed up after intervention end.) Data reported was broken down into these 4 groups (formed following re-randomisation after intervention end), however we have extracted and combined data for the original 2 groups (the control group data was not extracted as this group was not followed up after intervention end). |
| **76** | **Knauper 2018** | LOW | UNCLEAR | LOW | HIGH |  |
|  | Assessment justification: | "To randomize participants to the two intervention arms, a randomization sheet generated by a random digit generator is used (www.randomizer.org)." | "Throughout the recruitment process, the list of randomized numbers will be assigned to participants by the research coordinator in sequential order from 1 to 154 in the order in which participants completes the baseline CHIP appointment." | "However, the staff assessing the outcome variables (e.g. weight, EST) is blind to which intervention the participants were assigned." | 44.9% of the Enhanced DPP group attended the 12-month follow-up versus 70% of the standard DPP arm.  24-Month follow-up: Enhanced DPP: 51/107*100 = 47.7%; Standard DPP: 51/101*100 = 50.5% |  |
| **77** | **Kumanyika 2012** | LOW | LOW | LOW | LOW |  |
|  | Assessment justification: | “Eligible participants stratified by gender and age (~35 or over 35 year) were randomized to one of two treatment groups in a 1:1 ratio with randomly permuted blocks (block sizes of 2-6).” | “Random assignments were concealed from both participant and study staff prior to implementation.” | Weight objectively measured. | Descriptive analysis: Basic Program: 98/137 at 12mths Basic Plus Program: 89/124 at 12mths Weight change (>= 1 wt measurement after baseline) Basic program 133/137 at 12mths Basic plus program 124/124 at 12mths |  |
| **78** | **Leahey 2014** | LOW | UNCLEAR | LOW | LOW |  |
|  | Assessment justification: | With a random number generator, we assigned participants by using a 1:2:2 randomization scheme. | NS | Weight objectively measured. | Less than 50% attrition at 6- and 12-month follow-up. |  |
| **79** | **Leahey 2015** | LOW | UNCLEAR | LOW | LOW |  |
|  | Assessment justification: | "Approximately 50% of participants were the only participant within their team, but to avoid contamination, when multiple individuals were recruited from the same SURI team they were assigned as a single unit within the simple randomization procedure to ensure that all team members were randomized to the same study arm. The study statistician completed all randomization procedures." | NS | "Weight was measured to the 0.1 kg using a digital scale and height was measured at baseline using a wallmounted stadiometer." "Assessments were conducted by blinded staff." | Drop out at 12-months: SI: 76/91*100 = 86%; SII: 84/89*100 = 94%; SIG: 82/88*100 = 93% |  |
| **80** | **Lejeune 2003** | UNCLEAR | UNCLEAR | LOW | LOW |  |
|  | Assessment justification: | NS | NS | Weight objectively measured. | Retention rate: Week 13:  D Group: 85%;  DE group: 100%  Week 53:  D Group: 75%;  DE group: 70% |  |
| **81** | **Ley 2004** | HIGH | UNCLEAR | LOW | LOW |  |
|  | Assessment justification: | “They were then individually randomised to either an intervention group that was asked to consume a reduced-fat (RF), but otherwise ad libitum diet, or a control diet (CD) group that continued with their usual diet. An exception to this individual randomisation was made at one work-site where all six participants were Pacific Island’s women who worked closely together. They were all assigned to the RF group because individual randomisation was impractical. All those found to have diabetes on re-testing were referred to their general practitioners for management, but were still randomised for the study.”  Broken randomisation. | All those found to have diabetes on re-testing were referred to their general practitioners for management but were still randomised for the study. | Weight objectively measured. | less than 50% attrition at 1 - and 5-year follow-up. |  |
| **82** | **Li 2016** | LOW | UNCLEAR | LOW | LOW | UNCLEAR |
|  | Assessment justification: | "After a one‐week run‐in period, participants were randomly allocated to one of the following four groups by computer‐generated random numbers." | NS | "All measurements were conducted with standard procedures by the same clinical staff in the third hospital of Inner Mongolia medical college, who were blinded to the group allocation." | Percentage of participants retained at follow-up at 1 year: Usual care: 98.3%; Diet: 96.2%; 50g oats: 96.3%; 94.9%; "Eleven patients dropped out during the 1-year follow-up due to personal reasons with no difference in drop-out rates among the four groups (p = 0.774)." | Unclear whether groups were stratified by BMI in parent study. "A subgroup of 298 subjects, meeting the Chinese criteria of overweight (body mass index ≥ 24 kg/m2), was selected from 445 adult patients with T2DM, who had participated in the 30-day centralized management of a dietary program and the 1-year free-living follow-up in Baotou, China." |
| **83** | **Li 2005** | LOW | UNCLEAR | LOW | LOW | HIGH |
|  | Assessment justification: | "A random, permuted, block design was utilized for placement of subjects into the two treatment groups." | NS | Weight measured objectively. | Retention rate: Baseline:  MR: 49/52*100 = 94.2%;  IDP: 44/52*100 = 84.6%  6-months:  MR: 46/52*100 = 88.5%;  IDP: 36/52*100 = 69.2%  12-months: MR: 42/52*100 = 80.8%; IDP: 35/52*100 = 67.2% | MR group continued to received the meal replacements for the 12 month study duration at lower volume.  "For the first 5 days of the study, subjects randomized into the MR group replaced three meals per day with a soy MR (Slim Fast Food Company, Inc. West Palm Beach, FL 33401, USA). They also were instructed to add fruits and vegetables to their dietary intake. Thereafter, the MR group replaced two meals with the soy MR with continuing use of fruits and vegetables as snacks, plus a sensible third meal for three additional months. After the 3 months, subjects in the MR group were instructed to replace one to two meals per day with the soy shakes and consume correspondingly one to two sensible meals for the duration of the study." |
| **84** | **Lindstrom 2003** | LOW | UNCLEAR | LOW | LOW | HIGH |
|  | Assessment justification: | A randomization list was used | The nurses scheduling visits were blinded to randomisation. Study staff were not blinded. | Weight objectively measured. | At 3 years: 203/ 257 Control 231/ 265 Intervention  At 4 years: 170/257 Control 198/265 Intervention  From 5 years:  166/257 Control  200/265 Intervention | After the decision to end the intervention period, the intervention was continued until each participant’s next scheduled annual clinic visit. The end date thus varied from March 2000 to December 2001. After active intervention (median 4 years, range 1–6 years), participants still free of diabetes and willing to continue their participation (from year 6 - 200 in the intervention group and 166 in the control group) were further followed until diabetes diagnosis, dropout or the end of 2009, with a median total follow-up of 9 years and a time span of 13 years from baseline. |
| **85** | **Liss 2016** | LOW | LOW | LOW | LOW |  |
|  | Assessment justification: | "Prior to the study enrollment phase, randomization lists were generated by a senior statistician using SAS, version 9.2 (Cary, NC). Lists were created using 1-to-1 allocation, with blocks of 4, stratified by Y study site and race (non-Hispanic White; African-American; Other).” | "Randomization blocks were implemented by the study programmer (AC) and pre-loaded into a back-end field of a Microsoft Access (Redmond, WA) database table that was not available to study RAs. After the study RA collected data required for randomization at each participant's screening/enrollment visit, she clicked a button in Access to execute the randomization." | Weight objectively measured. | At 12 months, 78% of participants from the GLI group and 76% from the standard care group returned for outcome assessment. |  |
| **86** | **Little 2016** | LOW | LOW | UNCLEAR | LOW |  |
|  | Assessment justification: | “Upon completion of baseline questionnaires, the website automatically randomly assigned patients (1:1:1) via computer-generated random numbers…” | “Participants and investigators were masked to group allocation at the point of randomisaton…” | “Weight loss was measured with participants lightly clothed without shoes, at the same time every day when possible, with automated digital scales (Tanita Europe BV, Amsterdam, the Netherlands).” “When a blinded weight measurement could not be obtained, we used practice nurses’ recorded weights, and when that was not possible, we used participants’ reported weights.” | Weight loss averaged over 12 months was recorded in 666 (81%) participants.” Control: 136/279 weight at 6mths (HIGH); 227/279 weight at 12mths (LOW). Power + face to face: 148/269 wt at 6mths (LOW); 221/269 wt at 12mths (LOW). Power + remote: 155/270 wt at 6mths (LOW); 218/270 wt at 12mths (LOW). |  |
| **87** | **Lowe 2018** | LOW | UNCLEAR | LOW | LOW |  |
|  | Assessment justification: | Simple randomization via a table of random numbers was used to evenly assign participants to 1 of 3 conditions: | NS | Weight measured objectively. | Less than 50% attrition at 36-month follow-up. |  |
| **88** | **Ma 2015** | LOW | LOW | LOW | LOW |  |
|  | Assessment justification: | We applied our published dynamic block randomization method to assure better than chance between-treatment balance across six prognostic factors (study site, age, sex, race/ethnicity, BMI, and ACQ score). The method automatically ensures allocation concealment. Participants were randomly assigned to one of two treatment conditions | This randomization procedure not only minimizes imbalance for the chosen baseline covariates between treatment groups and correlated characteristics, but also ensures concealment of treatment allocation, with recruitment staff completely unable to influence allocation. A designated research staff member who is not involved in follow-up data collection or data analysis will carry out randomization using a computerized program. (Ma 2010) The trial design precluded blinding participants or interventionists to treatment assignment; however, the investigators, Data and Safety Monitoring Board members, outcome assessors, and data analyst were masked throughout the trial. Ma 2015 | 'Both groups given a weight scale.' 'Published protocols were used to obtain height (baseline only), weight, waist circumference, and blood pressure measurements.' Indicates weighed. | Control:  157/165 at 6mths; 147/165 at 12mths. Intervention:  154/165 at 6mths; 142/165 at 12mths. |  |
| **89** | **Manning 1994** | UNCLEAR | UNCLEAR | LOW | LOW |  |
|  | Assessment justification: | NS | NS | “Weight measurements were available irrespective of whether the patient completed the defined study group, scale weights were comparable throughout.” | Less than 50% attrition at 12- and 48-month follow-up. |  |
| **90** | **Manzoni 2016** | LOW | UNCLEAR | HIGH | HIGH |  |
|  | Assessment justification: | Authors only mention that the randomization scheme used for selecting the condition was generated by using the Web site www.randomization.com. However, no further information regarding the sequence generation is offered. | NS | Participants’ data were obtained 1 week after the start of the inpatient program, during the last week of hospitalization, and at 1-year follow-up (by postal mail) (LOW). Data at follow-up were self-reported (HIGH). | Control 29/52 at follow up (55.7%) LOW CBT 38/54 at follow up (70%) LOW VR 46/57 at follow up (80.7%) LOW Between group HIGH |  |
| **91** | **Marniemi 1990** | UNCLEAR | UNCLEAR | UNCLEAR | UNCLEAR |  |
|  | Assessment justification: | NS | NS | NS | All participant data used in the analysis but it's unclear how many were lost to follow-up. |  |
| **92** | **Martin 2008** | UNCLEAR | UNCLEAR | LOW | HIGH |  |
|  | Assessment justification: | “Eight physicians practicing at two clinics were randomly assigned to provide one of the two treatment conditions, with four physicians providing personalized weight loss interventions and four providing standard care treatment (two intervention and two control at each clinic). Specifically, the randomization utilized clinic as a stratification variable, and the practices within each stratum were assigned to level of treatment under a balanced randomization. The basis of the randomization was the within-stratum ranks of a uniform (0,1) (pseudo-) random deviate generated for each participating physician. This resulted in a nested design, with participants recruited for the study being identified with the randomization assignment of their primary care physician. The purpose of using a nested design was to reduce the likelihood of treatment contamination in the control subjects, and for this reason, only the intervention physicians were trained in the use of the active treatment components.” | NS | Trained personnel measured participants’ weight and height at each assessment in the physician’s office using a standardized protocol with a calibrated scale and stadiometer. | Completed 6mth programme 106/144 (or 106/137 as 7 become not eligible). At 6mths Standard care: 58/73 (or 58/69) LOW At 6 mths Intervention: 48/71 (or 48/68) LOW 'After accounting for medical exclusions and women lost to follow-up for other reasons, 105 completed the 6-month program, which resulted in an attrition rate of 27%. Another 42 participants discontinued the study by month 9, 51 discontinued by month 12, and 53 discontinued by month 18. Thus, the attrition rates at the 9-month, 12-month, and 18-month follow-up assessments were 29, 35, and 37%, respectively. Comparing between the two treatment groups, attrition was significantly greater (29%) in the intervention group immediately following the active treatment phase (i.e., month 6) as compared with standard care (12%), P < 0.01. Attrition was also greater among intervention participants (44%) at the final follow-up (i.e., month 18) as compared with standard care (23%), P < 0.02.' |  |
| **93** | **Mefferd 2007** | UNCLEAR | UNCLEAR | LOW | LOW | UNCLEAR |
|  | Assessment justification: | NS | NS | "Anthropometric measurements (obtained at baseline and 16 weeks) included height and weight (measured without shoes)..." | A little over ten percent of the participants dropped out of the study during the 16 weeks under analysis in this report, yielding a final sample size of (n = 76) at 16 weeks. All nine dropouts had been assigned to the intervention group.  Retention rate:  16-weeks:  Control: 100%; Intervention: 47/56*100= 83.9% 12-Months:  Control: 25/29*100 = 86%;  Intervention: 44/56*100 = 78.6% | Wait-list control; unclear if control group participants were aware they were wait-listed. |
| **94** | **Mengham 1999** | UNCLEAR | UNCLEAR | UNCLEAR | LOW |  |
|  | Assessment justification: | NS | NS | NS | 74/75 LOW 'Seventy-five patients were recruited and randomised. ' 'Of the 74 patients who completed the study' |  |
| **95** | **Messier 2013** | LOW | UNCLEAR | LOW | LOW |  |
|  | Assessment justification: | "A stratified-block randomization method was used to assign all eligible persons to 1 of the 3 intervention groups, stratified by BMI and sex." | NS | Weight, height, and BMI were obtained at baseline, 6months, and 18months using standard techniques. | "Of the 454 participants, 399 (88%) completed the study (returned for 18- month follow-up). Retention did not differ significantly among the groups (E, 89%; D, 85%; D+E, 89%)..." |  |
| **96** | **Miller 2002** | LOW | LOW | LOW | LOW |  |
|  | Assessment justification: | Randomization was stratified by race (African American and other), with an allocation ratio of 1:1 and a block size of 4. The order of randomization was constructed from a published list of random numbers; | assignments were issued by the study coordinator who opened sealed opaque envelopes that contained the group assignment. | Weight was measured biweekly by blinded personnel using a certified balance-beam scale. | Less than 50% attrition at 12-months follow-up. |  |
| **97** | **Mitsui 2008** | UNCLEAR | UNCLEAR | UNCLEAR | LOW |  |
|  | Assessment justification: | "...were randomly assigned to the intervention group..." | NS | NS | "Two participants in the intervention group and 1 in the control group dropped out of the program after week 12 for personal reasons." |  |
| **98** | **Molenaar 2010** | LOW | UNCLEAR | LOW | LOW |  |
|  | Assessment justification: | Computerized randomization | NS | Weight objectively measured. | Twenty-four participants (18%) did not complete the 6-month intensive intervention period and an additional nine participants (7%) dropped out during the 6-month follow-up period |  |
| **99** | **Moreno 2014** | UNCLEAR | UNCLEAR | LOW | LOW |  |
|  | Assessment justification: | "Patients were randomised and allocated to receive ... " | NS | Weight measured objectively.  WC was recorded with a standard flexible nonelastic metric tape over the midpoint between the last rib and the iliac crest, with the patient standing and exhaling. | At 12-months:  LC: 26/40 VLCK: 27/39  At 24-months:  LC: 23/40  VLCK: 22/39 |  |
| **100** | **Morgan 2010** | LOW | LOW | LOW | LOW |  |
|  | Assessment justification: | "The random allocation sequence was generated by a computer-based random number-producing algorithm in block lengths of six to ensure an equal chance of allocation to each group." | "To ensure concealment, the sequence was generated by a statistician and given to the project manager. Randomization was completed by a research assistant who was not involved in the assessment of participants and the allocation sequence was concealed when enrolling participants." | "Weight was measured without shoes on a digital scale to 0.1kg (model CH-150 kp; A&D Mercury, Adelaide, Australia)"  Systolic and diastolic blood pressure and resting heart rate were measured using a NISSEI/DS-105E digital electronic blood pressure monitor (Nihon Seimitsu Sokki, Gunma, Japan) under standardized procedures. | Retention rate: 3-month follow-up: Control: 27/31*100 = 87.1%;  SHED-IT: 28/34*100 = 82.4%  6-month follow-up: Control: 26/31*100 = 83.9%;  SHED-IT: 28/34*100 = 82.4%  12-month follow-up: Control: 20/31*100 = 64.5%;  SHED-IT: 26/34*100 = 76.5% |  |
| **101** | **Muggia 2014** | LOW | LOW | UNCLEAR | HIGH |  |
|  | Assessment justification: | “Participants were randomly allocated (allocation ratio 1:1) to the standard care or cognitive behavioral therapy group, using a computer-generated randomization application of STATA statistical package.” | “Randomization list was kept at Biometric Unit and clinicians were unaware of the treatment group until the subjects were enrolled. The treatment allocation was communicated by phone to the clinician every time a new patient was enrolled.” | Information on how weight was measured not stated. | A high attrition rate is observed and although multiple imputations are performed to reduce its impact bias, this cannot be excluded as also reported in similar studies.  At the six month, 114 patients (69.9% of the total) attended the follow-up visit, with an attrition rate of 30.1%. The percentage of visits attended was significantly greater in group A (83.3% vs 70.4% in group B, p < 0.001). At the 12 month, 78 patients (47.8% of the total) attended the follow-up visit, with an attrition rate at 1 year of 47.8 per 100 person-year. A total of 44 patients in the treatment arm A (53.0%) and 34 (42.5%) in group B completed the follow-up, with no significant differences between the two groups. |  |
| **102** | **Ng 2015** | LOW | UNCLEAR | LOW | LOW |  |
|  | Assessment justification: | “Recruited patients were randomized in 1:1 ratio to participate in the LMP or usual care through the use of a computer-generated list of random numbers.” | NS | "Anthropometric measurements, ESS, and laboratory tests, which included liver and renal function, fasting glucose, and lipids, were performed at baseline, 4 months, and 12 months." | "Sixteen participants in the intervention group were excluded after randomization, as six had never attended dietician visits, seven attended fewer than four dietician visits, and three maintained their high-energy and -fat food intake. There were six subjects in the control group lost to follow-up."  LMP Group:  45/61*100 = 73.8%;  Control:  37/43*100 = 86% |  |
| **103** | **Nicklas 2009** | LOW | UNCLEAR | LOW | LOW |  |
|  | Assessment justification: | "A total of 112 women met all study criteria and were randomly assigned (before baseline assessments) to 1 of the 3 interventions (Figure 1) by random number generation." | NS | Staff that measured the primary (abdominal visceral fat volume) and secondary (CVD risk factors) outcomes were blinded to group assignment. | Post-intervention:  CR only: 73%;  CR + Mod: 70%;  CR + Vigorous: 71%  6-month:  CR only: 67.6%;  CR + Mod: 72.5%; CR + Vigorous: 68.4%  12-month:  CR only: 58.8%;  CR + Mod: 67.5%;  CR + Vigorous: 68% |  |
| **104** | **Nilsen 2011** | LOW | LOW | LOW | LOW |  |
|  | Assessment justification: | Groups were randomly assigned to an “individual physician group” (IG) or an “individual plus interdisciplinary group” (IIG) by use of closed envelope method with unknown block sizes. | Closed envelope method. | “At every visit to the study physician, the following assessments were performed: fasting blood sample, systolic and diastolic blood pressure (SBP and DBP) according to recommended standards [18], waist circumference at a level midway between the lowest rib and the iliac crest to the nearest cm, height without shoes to the nearest cm (only first visit) and weight in indoor clothes to the nearest 100 g. Blood pressures were measured by an Omron M41 and weight with a Seca 771.” | Control IG: 89/104 at follow up Intervention IIG 93/109 |  |
| **105** | **Oldroyd 2006** | LOW | UNCLEAR | LOW | LOW |  |
|  | Assessment justification: | Eligible participants who agreed to take part were randomly allocated using a random number table to the intervention or control group at the first baseline appointment. | “Researchers performing the randomisation were blind to the group allocation.” | Weight was measured to the nearest 0.1 kg with the participants lightly clothed on SECA scales (Alpha Model 770 digital, SECA Limited, Birmingham, UK) | 6-month follow-up: Control: 32/39 * 100 = 82%; Intervention: 37/39*100 = 94.9%  12-month follow-up: Control: 30/39 * 100 = 77%; Intervention: 32/39*100 = 82%  24-month follow-up: Control: 24/39 * 100 = 62%; Intervention: 30/39*100 = 77% |  |
| **106** | **Parikh 2010** | UNCLEAR | UNCLEAR | LOW | LOW |  |
|  | Assessment justification: | "Participants were randomized to intervention or delayed intervention (in 1 year) by blocked randomization (block size=4) by recruitment site."  No further information given. | NS | Weight measured objectively. | "The study had some attrition: 83 participants returned at 3 months, 79 at 6 months, and 72 at 12 months (37 control, 35 intervention). Four participants became ineligible because of pregnancy. The 23 participants lost to follow-up at 12 months did not differ from those who returned for the final check-up in age, gender, weight, BMI, or family history of diabetes.” |  |
| **107** | **Pedersen 2013** | LOW | UNCLEAR | LOW | LOW | UNCLEAR |
|  | Assessment justification: | "Randomization was stratified according to BMI (≤ 32.5; > 32.5). A third party unrelated to the study performed en bloc randomization with bloc size 2, 4 and 6 using Stata 11.1 software (StataCorp, 4905 Lakeway Drive, College Station, TX, USA)." | NS | "All participants are examined at baseline, after 12 weeks and after a year. Most examinations were performed at University Hospital of Bispebjerg, Department of Cardiology, except the MRI that was performed at University Hospital of Herlev and PET that was performed at Rigshospitalet." This included body composition assessed by anthropometry. | Dropout rate at 12-weeks follow-up:  AIT: 31/35*100 = 88.6%;  LED: 34/35*100 = 97.1%  Drop out rate at 1-year follow-up:  AIT: 26/35*100 = 74.3%;  LED: 29/35*100 = 82.9% | "Drop-out rates (26% and 17% in the AIT and LED+AIT group, respectively) imply that intensive lifestyle changes require physical and mental strength and support from relatives and employers especially when considering long-term interventions." "However, drop-out rates introduce a risk of bias due to small sample size and challenges related to generalisability as discussed above. " |
| **108** | **Perri 1986** | UNCLEAR | UNCLEAR | LOW | UNCLEAR |  |
|  | Assessment justification: | "Subjects were assigned from blocks stratified by percentage over ideal weight to one of four experimental conditions in a 2 × 2 factorial design. Two treatment conditions (behavior therapy or behavior therapy plus aerobic exercise) were crossed with two posttreatment conditions (no posttreatment contact or a multicomponent posttreatment maintenance program)." |  | "Changes in weight were assessed at posttreatment and at 3-, 6-, 12-, and 18-month follow-up sessions." | "Of the 90 subjects who began the program, 18 dropped out during the initial treatment phase, representing an attrition rate of 20%. Rates of attrition did not differ significantly among groups, and subjects who dropped out did not differ significantly from subjects who completed the program in either initial body weight or percentage overweight. During the follow-up phase of the study, 4 subjects elected not to participate in the maintenance program and 1 person was hospitalized (for a problem unrelated to obesity) and withdrew from the study." |  |
| **109** | **Perri 1997** | UNCLEAR | UNCLEAR | LOW | LOW |  |
|  | Assessment justification: | NS | NS | "Weight was measured on a balance beam scale at each weight loss program session, with the participant in indoor clothing without shoes." | "Forty of the 49 (81.6%) participants completed the 12-month treatment program. One participant in the home-based program became pregnant during Month 2, and her data were excluded from all analyses. There were 7 dropouts in the group-based exercise condition and 1 dropout in the home-based exercise condition" |  |
| **110** | **Perri 2001** | UNCLEAR | UNCLEAR | UNCLEAR | UNCLEAR |  |
|  | Assessment justification: | The authors only mention that participants were assigned randomly to one of three conditions, however no information regarding the methods of randomization are provided. | NS | "The primary outcome measure was change in body weight assessed over the course of 17 months." | Total number of participants at baseline: 103; 88 completed the 5-month program, yielding an initial treatment completion rate of 85%. At the conclusion of initial treatment, the small number of male participants was unevenly distributed across conditions, and the data from these participants (n = 8) were excluded from further analysis. Thus, the study sample consisted of 80 women. 17 Month follow-up: BT = 15; RPT = 20; PST = 23 |  |
| **111** | **Pettman 2009** | LOW | LOW | LOW | LOW |  |
|  | Assessment justification: | "Using a random number generator (MS Excel), participant data were then distributed into three groups of approximately equal numbers. Unidentifiable individuals were block-matched to achieve an even gender balance and distribution of MetS risk factors over the 3 groups by calculating means for waist, DBP and age together with counts of males and females for each group. The groups were checked for significant differences between variables using independent samples t-tests. The three groups were then randomly assigned to ‘A’, ‘B’ or ‘C’ corresponding to INT-A, INT-B or CON respectively.” | "Study personnel generating the sequence were not aware of participant details, due to obscuring of identification numbers. Final group assignment was conducted by an impartial person." | "Body weight was measured to the nearest 0.1 kg (Tanita Ultimate scales™ Model 2000, Tanita Corporation, Tokyo, Japan), except for individuals weighing over 150 kg, who were weighed on a single set of electronic glass scales (Model 3200, Propert Pty Ltd, Castle Hill, NSW, Australia). The same set of scales was used at subsequent measurements for each participant." | Retention rate: 4-months:  Control: 86%;  INT-A: 98%;  INT-B: 92.6%  12-months: Control: 36/43*100 83.7%;  INT-A: 44/48*100 = 91.6%;  INT-B: 35/49*100 = 92.6% |  |
| **112** | **Poelman 2015** | LOW | UNCLEAR | HIGH | LOW |  |
|  | Assessment justification: | “Randomization lists were generated with standard statistical computer software (IBM SPSS Statistics 20.0). Based on the randomization list, the researcher (M.P.P) allocated subjects to one of the groups.” | “Due to the nature of the intervention, it was not possible to blind participants to their allocated condition.” | At baseline (T0), weight was measured using two different scales: a professional one (the Marsden MPMS-250 digital scale, Oxfordshire, UK) and the participant’s scale, in light clothes and with shoes removed. Measurements were highly correlated (regression coefficient= 0.99; intercept=-0.10), indicating that both scales yield largely similar results. At T2, the weight was also objectively measured using the professional scale, during a home visit from the researchers. At T1 and T3, participants were asked again to weigh themselves.  T0 = Baseline T1 = 3-months T2 = 6-months T3 = 12-months  T1 (3-months) time point was intervention end and participants self-weighed. | Less than 50% at 12-month follow-up. Control 73.4% Intervention 64% at 12-month follow-up  At 3-months:  usual care: 111/139; intervention: 85/138  At 6-months: usual care: 118/139; intervention: 105/138  At 12-months: usual care: 102/139; intervention: 89/138 |  |
| **113** | **Promrat 2010** | LOW | LOW | LOW | LOW |  |
|  | Assessment justification: | "Randomization was performed using a random number generator developed by the project statistician, with a target enrollment of 30 participants." | "The randomization process was conducted by a project staff who was blinded to the randomization sequence." | "Data collection was obtained by trained staff who were not aware of the group assignment or sequence of measurement." | "Thirty participants (97%) completed the study. One participant (3%) in the lifestyle intervention group withdrew from the study after 3 months. All other participants adhered to the study protocol follow-up schedule." |  |
| **114** | **Provencher 2009** | UNCLEAR | UNCLEAR | LOW | LOW |  |
|  | Assessment justification: | "Randomization was performed within each phase, and women were then assigned to one of the 3 treatment conditions: HAES group (N = 48), SS group (N = 48), or control group (N = 48). | NS | "Height was measured to the nearest millimeter with a stadiometer, and body weight was measured to the nearest 0.1 kg on a calibrated balance. Participants were asked to dress lightly and to remove their shoes for these measurements." | Baseline (T=0):  Control: 46/48*100 = 95.8%;  SS Group: 46/48*100 = 95.8%;  HAES: 100%  4-months (T=4): Control: 38/48*100 = 79.2%;  SS Group: 39/48*100 = 81.3%;  HAES: 44/48*100 =91.7%  10-months (T=10): Control: 34/48*100 = 70.8%;  SS Group: 38/48*100 = 79.2%;  HAES: 45/48*100 =93.8%  16-months (T=16): Control: 32/48*100 = 66.7%;  SS Group: 33/48*100 = 78.8%;  HAES: 41/48*100 =85.4% |  |
| **115** | **Rock 2015** | LOW | LOW | LOW | LOW |  |
|  | Assessment justification: | "Random assignment was performed by a centralized computer process, assigning participants in a 1:1 ratio to either the intervention arm or the less intensive intervention control arm, stratified by age (or 55 years), stage (I v others [II and III]), and study site. | "Randomization was performed by a centralized computer process" | Weight was measured at baseline and at 6-, 12-, 18- and 24-month follow-up visits, using a calibrated scale. | "Weight was not available for 44 intervention group and 61 control group participants at 24 months" |  |
| **116** | **Rolls 2005** | UNCLEAR | UNCLEAR | LOW | LOW |  |
|  | Assessment justification: | “…a stratified randomization scheme was used to balance the distribution of subject sex and age across the groups.” | NS | “Body weight was measured at each counseling session, with the subject wearing light clothing without shoes, using a scale that was regularly calibrated.” | Less than 50% attrition at 12-month follow-up. |  |
| **117** | **Rolls 2017** | LOW | UNCLEAR | LOW | LOW |  |
|  | Assessment justification: | "Participants were stratified by body mass index and age, and randomly assigned to one of three groups, using blocks of six sequences from a random number generator." | NS | "Subjects were weighed to the nearest 0.1 kg while wearing a lightweight outfit kept for them at the center. Height was measured with a stadiometer; waist circumference was measured at the right iliac crest. | 3-month:  total sample follow-up: 170/ 186* = 91.4%; Standard advice group: 59/62*100 = 95%; Portion selection: 58/62*100 = 93.5%; Pre-portioned foods: 59/62*100 = 95%  6-month:  total sample follow-up: 149/ 186* = 80.1%; Standard advice group: 52/62*100 = 83.9%; Portion selection: 51/62*100 = 82.3%; Pre-portioned foods: 53/62*100 = 85.5%  12-month:  total sample follow-up: 136/ 186* = 73%; Standard advice group: 49/62*100 = 79%; Portion selection: 51/62*100 = 82.3%; Pre-portioned foods: 51/62*100 = 82.3% |  |
| **118** | **Rosas 2015** | LOW | UNCLEAR | LOW | LOW |  |
|  | Assessment justification: | Participants are randomized to one of three arms according to the ratio 1 UC: 2 CM: 2 CM+CHW. After all baseline data were collected, a blinded data analyst/biostatistician confirmed study data completion and randomizes the participant to one of the three arms in permuted blocks stratified by sex, BMI (30-34.9, 35-39.9, or ± 40), and diabetes status. | The data analyst/ biostatistician was blinded. | Data collection staff were blinded to treatment assignment.  Weight was measured at each assessment visit in duplicate using a Detecto scale, whereas height was measured in duplicate using a wall-mounted stadiometer at baseline only. Participants’ anthropometric measures were assessed without their shoes and coats. | As in other lifestyle intervention trials, all participants did not attend all planned intervention activities (one-on-one case management, groups sessions, and home visits). This limited our ability to test whether the planned intervention had the intended effect. Nevertheless, the percentage of participants attending each activity was within the expected range. Body weight was collected from 207 participants (100%) at baseline, followed by 190 (91.8%) at 6 months, 171 (82.6%) at 12 months, and 177 (85.5%) at 24 months. |  |
| **119** | **Ross 2012** | LOW | UNCLEAR | LOW | LOW |  |
|  | Assessment justification: | Eligible participants were randomized on the basis of a computer automated randomization sequence after the acquisition of primary outcome data. Randomization was stratified by sex, age, and WC measurement | NS | Weight objectively measured. | Of the 490 participants, 396 (80.8%) returned for follow-up testing at 24 months. |  |
| **120** | **Samaras 1997** | UNCLEAR | UNCLEAR | LOW | LOW | UNCLEAR |
|  | Assessment justification: | 'Subjects were randomised into two groups'  No further information given. | NS | All anthropometric measures were performed by a trained investigator (AMM). Body height was measured to the nearest cm using a stadiometer with the subject barefoot; body weight to the nearest 0.1 kg in light street clothing. B | Control:  13/13 at 12-months Intervention:  13/13 at 12-months  0% dropout by end of study | After the 6-month programme, the exercise sessions remained available to subjects in the intervention group. |
| **121** | **Santanasto 2011** | LOW | UNCLEAR | LOW | LOW |  |
|  | Assessment justification: | "Randomization was done using a Microsoft Access-based random-number generating algorithm with stratification by age and sex to further ensure balance between groups (Microsoft Redmond, Washington)." | NS | "At the baseline (BL) screening visit and followup visits, body height (cm) was measured using a wall-mounted stadiometer and body weight (kg)with a standard certified calibrated scale and were used to calculate BMI (weight (kg)/height (m2))." | "All participants, with the exception of one in the PA+SA group, were followed up to their 6FU visits." 12 Month follow-up: PA +LW = 18/21*100 = 85.7%; PA + SA = 93.3% |  |
| **122** | **Sattin 2016** | UNCLEAR | UNCLEAR | UNCLEAR | LOW |  |
|  | Assessment justification: | “Churches were recruited as pairs in the study based on congregation size. These pairs were included in six cohorts with each cohort including either two or four churches. Each church pair was then randomized to the Fit Body and Soul (FBAS) behavioral lifestyle intervention or Health Education (HE) comparison group.”  No further information given. | Allocation concealment through pastor but no further detail given. | NS | No attrition. |  |
| **123** | **Schubel 2016** | LOW | LOW | LOW | LOW |  |
|  | Assessment justification: | "They sequentially enter the study and are randomly allocated to the three dietary programs (ICR, CCR, or HD) by RANDI2 [9], a web-based software using a block size of six. Randomization is stratified by age (<50 years/ ≥ 50 years) and sex." | Refer to ‘Random sequence generation (selection bias)’. | "All outcome assessments (see Table 2) are performed by trained study personnel following standard operating procedures." | Overall, 144 participants (96.0%) completed the 12-wk intervention phase, 143 (95.3%) the 12-wk maintenance phase, and 136 (90.7%) the 26-wk follow-up phase (Figure 1). Across the entire study period of 50 wk there were 4 dropouts in the ICR (91.8%), 7 in the CCR (85.7%), and 2 (96.2%) in the Control group. |  |
| **124** | **Seligman 2011** | LOW | LOW | LOW | LOW |  |
|  | Assessment justification: | “Randomization was performed using a computer sequence with centrally concealed allocation.” | Refer to ‘Random sequence generation (selection bias)’. | “Body mass index was calculated as weight/height2 (kilograms per square meter). Waist was measured between the last rib and the iliac crest. Body fat mass was assessed with bioelectrical impedance (Omron HBF 306 Bioimpedance Analyzer).” | Less than 25% attrition at 12-months follow-up. |  |
| **125** | **Shikany 2013** | LOW | LOW | LOW | LOW |  |
|  | Assessment justification: | "At the baseline visit, eligible participants were randomly assigned to the MD or FB group via a pseudorandom number generator with a 1:1 allocation ratio" | “The allocated group was indicated on cards contained in sequentially numbered, opaque, sealed envelopes prepared in the Department of Biostatistics, UAB School of Public Health. To randomize a participant, the study coordinator opened the next consecutively numbered envelope in the presence of the participant.” | “…fasting serum glucose concentration was assessed; height, weight and blood pressure were measured; Body weight was assessed at baseline and at the 26- and 52-week clinic visits as outcome measures (and at the 8-, 16-, 32- and 40-week clinic visits as a check of participant progress), with participants in light clothing and no shoes using a Tanita model BC-418 digital scale/body composition analyzer (Tanita Corporation of America, Inc., Arlington Heights, IL, USA).” | 6-month retention rate:  FB: 49/60 = 81.6%;  MD: 56/60 = 93.3%  12-month retention rate: FB: 56/60 = 93.3%;  MD: 57/60 = 95%  Food based: 45/60 Medifast: 50/60 |  |
| **126** | **Silva 2010** | LOW | UNCLEAR | LOW | LOW | UNCLEAR |
|  | Assessment justification: | "...using the random number generator function for Microsoft Excel 2007 for Windows." | NS | "Assessments included lab-measured body weight and body composition (assessed at baseline, 4 and 12 months (end of the intervention program))..."  At 2 and 3 year follow-ups: "Body weight was measured twice, using an electronic scale calibrated on site and accurate to 0.1 kg (SECA, Hamburg, Germany)." | Retention rates at each follow-up (not reported for 2-year follow-up):  12-months:  Comparison group: 80%; Intervention group: 93%;  3-year:  Comparison group: 80%; Intervention group: 79%*  *"For the 36-month analyses reported herein, 2 women without 36-month anthropometric data were excluded, leaving a final sample of 154 women." | "A total of 258 women completed initial assessments and were randomized to intervention and comparison groups. Thirty-seven women were subsequently excluded from all analyses because they started taking medication (antidepressant, anxiolytic, and antiepileptic) susceptible to affect weight (n = 13) or because of serious chronic disease diagnosis or severe illness/injury (n = 4). Others were excluded because of pregnancy (n = 11) or because they entered menopause (n = 9). These 37 women were of similar age (P = 0.737) and BMI (P = 0.852) as the 221 participants who were considered as the valid initial sample for this study." |
| **127** | **Solbrig 2018** | LOW | UNCLEAR | LOW | LOW | UNCLEAR |
|  | Assessment justification: | “Participants were randomized to MI or FIT by the lead researcher using https://www.randomizer.org/ (random pairs option).” | NS | In the two posttreatment assessment sessions, research assistants (RAs) who were blind to the intervention group, collected and recorded primary outcomes. RAs blind to intervention measured waist and weight, and participants completed process measures online. | Attrition: less than 25% of participants in each arm at 6-month (programmes' end) and 12month. | Both interventions were delivered individually by the lead author. Potential for contamination bias. |
| **128** | **Somers 2012** | LOW | LOW | LOW | LOW |  |
|  | Assessment justification: | "...a data technician unfamiliar with the research protocol used a random allocation computer software program to assign participants in blocks (minimum = 27, maximum = 39) to 1 of 4 treatment conditions." | Refer to ‘Random sequence generation (selection bias)’. | "Weight was measured to the nearest 0.1 kg without shoes in the standing position. " | "Seventy percent (n = 163) of all randomized participants completed the 2-year study..."; "Twenty-four participants (6 from PCST-only, 10 from BWM-only, 4 from PCST + BWM, 4 from standard care) dropped out of the study after randomization but before treatment..."; "Participant dropouts at other study intervals were as follows: 20 participants dropped out before the posttreatment assessment (6 from PCST-only, 3 from BWM-only, 5 from PCST + BWM, 6 from standard care); 15 participants dropped out before the 6-month follow-up assessment (5 from PCST-only, 2 from BWMonly, 4 from PCST + BWM only, 4 from standard care); and 10 participants dropped out before the 12-month posttreatment assessment (4 from PCST-only, 4 from BWM-only, 2 from PCST + BWM only, 0 from standard care)." |  |
| **129** | **Spring 2013** | LOW | UNCLEAR | LOW | LOW |  |
|  | Assessment justification: | “…computer generated using the method of randomly permuted blocks.” | NS | “Weight was measured with the participant dressed in light clothing with shoes off on a calibrated balance beam scale at randomization and at 3-, 6-, 9-, and 12-month follow-up.” | Standard Group:  30/35 at 3mths;  28/35 at 6mths;  29/35 at 9mths;  27/35 at 12mths.  + Mobile group:  30/34 at 3mths;  29/34 at 6mths;  27/34 at 12mths.  'The proportion of missing data ranged from 13.0% to 21.7% across post randomization assessment periods, and the proportion of participants who attended all 4 outcome assessments was 73.9%.' |  |
| **130** | **Spring 2017** | LOW | LOW | LOW | HIGH |  |
|  | Assessment justification: | "Once all eligible participants of a cohort were assigned to a group, the three groups within each stratum were randomized by a statistician using a randomly permuted block with three cells." | "The statistician notified the project staff, who then revealed the treatment assignment (STND, TECH, or SELF) to participants during the first in-person group session." | "Body weight was measured without shoes on a calibrated balance beam scale at baseline and at 3, 6, and 12 months." | "Attrition at the final 12-month follow-up assessment was greater for SELF (25.0%) than either STND (12.5%) or TECH (3.1%)." 21.9% difference in attrition rate at 12 months follow-up. |  |
| **131** | **Stahre 2005** | UNCLEAR | UNCLEAR | LOW | LOW |  |
|  | Assessment justification: | NS | NS | “Weighing was always done without shoes and with light clothing using a calibrated scale. Those hospital personnel who were not participating in the study checked all the final weight measures.” | Control:  36/43 at 6mths; 33/43 at 12mths; 31/43 at 18mths.  Treatment: 57/57 at 10-weeks; 47/57 at 6mths,  40/57 at 12mths;  34/57 at 18mths. |  |
| **132** | **Stenius-Aarniala 2000** | LOW | UNCLEAR | UNCLEAR | LOW |  |
|  | Assessment justification: | Randomisation was by "shuffling cards", with the help of someone not involved in the study. | Refer to ‘Random sequence generation (selection bias)’. | NS | Control: 19/19 Treatment: 19/19 |  |
| **133** | **Stevens 1993** | UNCLEAR | LOW | LOW | LOW |  |
|  | Assessment justification: | "At clinics using the weight reduction intervention, randomization was conducted within high- and low-weight strata, with only high-weight participants eligible for the weight reduction group." | Centralized allocation by telephone; if not possible, sealed opaque envelopes. | "In addition, weights and blood pressures were recorded for all participants during official clinic visits 3, 6, 12, and 18 months after they entered the study." | 93% followed up at 12 months overall:  93% intervention; 93% control.  Reasons for attrition not reported. |  |
| **134** | **Stevens 2001** | UNCLEAR | LOW | LOW | LOW |  |
|  | Assessment justification: | NS | Centralized allocation via telephone to central randomizing centre or via sealed opaque envelopes. | "Blood pressure and weight were measured every 6 months after randomization to the end of follow-up at 36, 42, or 48 months, depending on randomization date. Clinic staff who were blinded to study group assignment made these assessments." | 92% followed up at 18 months overall: 92% intervention, 92% control.  Reasons for attrition not reported. |  |
| **135** | **Strobl 2013** | LOW | LOW | LOW | LOW |  |
|  | Assessment justification: | “The random sequence was generated at the University of Würzburg by staff not working at the rehabilitation clinic, using a computer program.” | “After having recruited a participant, clinic staff requested the randomization result from the scientific staff by phone (telephone randomization) thus guaranteeing concealment of randomization up to recruitment.” | “Body weight was assessed by both self-reports and physician measurements (at 12 months). Both assessments were highly concordant (intraclass correlation coefficient 0.99), with patients reporting slightly lower weight than did physicians (mean difference = –0.61, (standard deviation (SD) 1.88)). For the outcome analysis, the physician measurement of body weight was used whenever possible.” | Usual care:  203/239 at 6mths; 164/239 at 12mths  Intervention:  201/228 at 6mths; 177/228 at 12mths. |  |
| **136** | **Sundfor 2018** | LOW | LOW | LOW | LOW |  |
|  | Assessment justification: | "A statistician prepared a computer-generated random number list." | "The project leader (TS) opened numbered and sealed envelopes consecutively with no exception." | "Body weight was measured following a 10-h fast using the same calibrated digital scale to the nearest 0.1 kg." | "As shown in the Consolidated Standards of Reporting Trials flow chart four dropouts occurred in the intermittent versus three in the continuous energy restriction group." Greater than 90% of participants returned for all follow-up time points. |  |
| **137** | **Tapsell 2017** | LOW | LOW | LOW | HIGH |  |
|  | Assessment justification: | "Randomisation was conducted after the second screen for eligibility and performed remotely by an investigator unrelated to the clinic using a computer generated randomisation sequence (STATA V12, StataCorp LP, College Station, TX). The randomisation was stratified according to sex and BMI (low BMI: ≤30 and high BMI: >30). Randomisation was performed in randomly allocated blocks of 3, 6 or 9. " | "The randomisation list was provided to the study team who added eligible participants sequentially for each of the strata. The randomisation and participant database was only accessible by the HealthTrack study co-ordinator and administrator for security." | "Body weight (kg) was measured in an upright position in minimal clothing and without shoes using scales with a bio-electrical impedance component to also estimate body fat (%) (Tanita TBF-662, Wedderburn Pty Ltd., Ingleburn, NSW, Australia)." | "The intensive phase was completed by 298 participants (withdrawal rate 18%) and the 12 months follow-up by n=178 participants (withdrawal rate 39%)."  Total sample withdrawal rate at 12 months = 178/377*100 = 47% 12-month follow-up rate per group: Control: 61/126*100 = 48%;  Intervention: 45/120*100 = 36%; Intervention plus walnut: 72/126*100 = 57% |  |
| **138** | **TarragaMarcos 2017** | UNCLEAR | UNCLEAR | LOW | LOW |  |
|  | Assessment justification: | NS | NS | Weight objectively measured.   Blood pressure was measured using an automated and calibrated electronic device, according to the recommendations of the Spanish Society of Arterial Hypertension. | There were no dropouts in G1 or G2 during the follow-up period, however 4 patients left G3 for personal reasons, leaving this group with 55 patients. |  |
| **139** | **Teeriniemi 2018** | LOW | UNCLEAR | LOW | LOW |  |
|  | Assessment justification: | "MS Excel was used by an independent researcher to produce a randomization list with random permuted blocks of 24." | NS | Weight was measured by a study nurse. | "A total of 108 participants (20.3%) did not return to the study centre for the 1-year visit (Fig. 2), and 49 participants dropped out between the 1-year and 2-year visits. Thus, 375 study subjects completed the study per protocol, and the attrition rate at 24 months was 29.5% (n = 157). No statistically significant differences amongst the dropouts were found between the study arms..." |  |
| **140** | **ter Bogt 2009** | LOW | UNCLEAR | LOW | LOW |  |
|  | Assessment justification: | "... patients were allocated using computer-generated random numbers...". | NS | "Body weight was measured on an electronic scale with subjects wearing light clothing and no shoes..." | Low dropout rate after 1 year (9%). |  |
| **141** | **The Look AHEAD Research Group 2010** | LOW | LOW | LOW | LOW | HIGH |
|  | Assessment justification: | "Eligible participants are randomly assigned to either diabetes support and education or lifestyle intervention using a web-based data management system that verifies eligibility. Randomization is stratified by clinical center and blocked with random block sizes." (protocol) | "Eligible participants are randomly assigned to either diabetes support and education or lifestyle intervention using a web-based data management system that verifies eligibility. Randomization is stratified by clinical center and blocked with random block sizes." (protocol) | "Weight was measured in duplicate on a digital scale." | Retention rate:  Year 1:  DSE: 95.7%; ILI: 97.1%; Year 2:  DSE: 93.5%; ILI: 94.9%; Year 3:  DSE: 93.8%; ILI: 94.0%; Year 4:  DSE: 93.0%; ILI: 94.1%; Year 5:  DSE: 92.2%; ILI: 93.3%; Year 6:  DSE: 90.6%; ILI: 92.0%; Year 7:  DSE: 89.3%; ILI: 90.6%; Year 8:  DSE: 88.3%; ILI: 89.9%. | Participants in the intervention arm who, during the first 6 months, failed to lose 10% of their initial weight were offered a weight loss medication (orlistat). Those who lost <5% were encouraged by their lifestyle counselor to try pharmacotherapy, whereas those who lost 5.0% to 9.9% were informed of medication and could receive it on request. Medication was not offered to individuals who lost greater than or equal to 10% of initial weight and maintain the loss. 523 out of 2570 participants in the ILI study arm took Orlistat as part of the intervention. |
| **142** | **Trepanowski 2017** | LOW | UNCLEAR | LOW | LOW | HIGH |
|  | Assessment justification: | "Randomization was performed by a stratified random sampling procedure by sex, age (18-42 years and 43-65 years), and body mass index (25.0-32.5 and 32.6- 39.9). Block size ranged from 1 to 11 participants." | NS | "...body weight, which was measured monthly via a digital scale while the participant was in a hospital gown." | 69.0% of participants completed the study. "The dropout rate was highest in the alternate-day fasting group (13 of 34 [38%]), relative to the daily calorie restriction group (10 of 35 [29%]) and control group (8 of 31 [26%])." | "Participants in the control group were instructed to maintain their weight throughout the trial and not to change their eating or physical activity habits... Controls who completed the 12-month trial received 3 months of free weight-loss counseling and a 12-month gym membership at the end of the study." |
| **143** | **Tsai 2010** | UNCLEAR | LOW | LOW | LOW |  |
|  | Assessment justification: | Randomization was blocked in groups of six. | Sealed envelopes. | Weight was assessed by a research assistant (B.J.I.), who was not masked to treatment assignment. | Control: 24/26 at 6mths; 25/26 at 12mths Brief counselling: 21/24 at 6mths; 22/24 at 12mths |  |
| **144** | **Tuomilehto 2009** | LOW | LOW | LOW | LOW |  |
|  | Assessment justification: | “…the subjects were allocated randomly to two study groups by a study nurse according to a previously generated randomization plan. | Randomised by study nurse who did not take part in subsequent intervention. | The weight was measured at every visit. | Control: 10% drop out Intervention: 13% drop out |  |
| **145** | **van de Glind 2017** | LOW | LOW | LOW | LOW | HIGH |
|  | Assessment justification: | "The allocation sequence for each football club was generated by a computer programme written by a statistician not involved in the final analysis. The sequence was generated using randomised permuted blocks, stratified by club, with block lengths of 4 and 6, at random. The sequence was securely stored, with access restricted to those responsible for maintaining the randomisation system." | "Trial coordinators accessed randomisation allocation via a secure online portal." "It was not possible to mask participants or the fieldwork team to allocation, but the primary outcome measurements could not be accessed by either, and allocation was not known by study statisticians until after database lock." | "Body weight was measured using an electronic flat scale (Tanita HD366) with light clothing." | 91% and 92% of participants per group attended the post program follow-up time point; 88% and 92% attended the 12-month follow-up. | Wait-list control, no blinding. |
| **146** | **Viegener 1990** | UNCLEAR | LOW | UNCLEAR | LOW |  |
|  | Assessment justification: | "randomly assigned"  No further information given. | NS | NS | Of the 85 subjects who began the program, 22 (11 from each condition) dropped out during the treatment phase of the study; and 3 clients (2 from the standard, 1 from the intermittent group) who completed treatment declined participation in the maintenance phase meetings. The between-group differences in both initial and overall attrition rates were not significant, and subjects who dropped out did not differ significantly on pretreatment measures of body weight or percentage overweight from those who completed the program (all ps > .20). |  |
| **147** | **Vissers 2010** | UNCLEAR | UNCLEAR | LOW | LOW |  |
|  | Assessment justification: | NS | NS | Body weight was measured with a digital scale to the nearest 0.1 kg. | Less than 50% attrition at 12-month follow-up. |  |
| **148** | **Volpe 2008** | UNCLEAR | UNCLEAR | LOW | UNCLEAR | HIGH |
|  | Assessment justification: | "Participants were randomly assigned, in a stratified manner based on BMI, to one of three treatment conditions..." | NS | "Body weight was measured on a balance-beam scale accurate to 0.5 kg while the subject was wearing a swimsuit and no shoes." | NS | Raw data doesn't match up with information presented in graphs. Used raw data. |
| **149** | **von Gruenigen 2012** | UNCLEAR | UNCLEAR | LOW | LOW |  |
|  | Assessment justification: | "Randomization was stratified using block sizes of 6 or 8 by baseline BMI (25.0–39.9 versus >40)." | NS | "The RD weighed participants in private at the beginning of each session and weekly food/activity records were reviewed." | "Attrition in the trial overall was 21.3%. Six (14.6%) patients in the LI group versus 10 (29.4%) in UC did not complete the twelve-month assessments, p=0.159." |  |
| **150** | **vonGruenigen 2008** | LOW | UNCLEAR | LOW | LOW |  |
|  | Assessment justification: | “Participants were randomly assigned to LI or UC. Randomization was stratified according to patient BMI (25- 39.9 versus >40 kg/m2) using a stratified blocked randomization scheme in order to achieve comparability between the study groups based on BMI…” | NS | “Participants were weighed in street clothes without shoes on a Detecto hand rail scale (model #6855) and weight was recorded to the nearest 0.1 kg.” | At 12-months: Control: 18/22 Intervention: 17/23 |  |
| **151** | **Wadden 1994** | UNCLEAR | UNCLEAR | LOW | LOW |  |
|  | Assessment justification: | 'randomly assigned'  No further information given. | NS | Weight measured on a balance scale. | BDD:  17/21 at week 26 and 52; 16/21 week 78 VLCD:  28/28 week 9;  26/28 at week 26; 23/28 at week 52; 21/28 at week 78. |  |
| **152** | **Wadden 1998** | UNCLEAR | UNCLEAR | LOW | LOW |  |
|  | Assessment justification: | 'randomly assigned'  No further information given. | NS | Weight measured objectively. | 119/128 at week 8; 115/128 at week 17; 113/128 at week 24; 99/128 at week 48; 77/128 at 23-months. |  |
| **153** | **Wadden 2004** | UNCLEAR | UNCLEAR | LOW | LOW | UNCLEAR |
|  | Assessment justification: | NS | NS | “Body weight was measured using a balance-beam scale or an electronic digital scale (model 6800; Detecto). Participants were weighed in light clothing, without shoes, and were measured on the same scale throughout the study.” | Of the 43 women who were randomized to the BDD group, 37 (ie, 86%) remained in the study at week 20, 30 (70%) at week 40, and 26 (60%) at week 65. Of the 41 MR participants, 37 (90%), 31 (76%), and 28 (68%) remained at weeks 20, 40, and 65, respectively. Corresponding numbers for the 39 participants assigned to ND were 38 (97%), 34 (89%), and 28 (74%), respectively. | "(We note that an additional 21 women were recruited into the study and randomly assigned to a pilot intervention. It examined the efficacy in inducing weight loss of individual brief behavioral treatment [ie 20–25 min sessions] provided once a month. Results of this group [which was included to assess a new method of managing obesity in primary care practice] will be described in a separate report.)" |
| **154** | **Waleekhachonloet 2007** | UNCLEAR | UNCLEAR | LOW | LOW | UNCLEAR |
|  | Assessment justification: | NS | NS | Weight and waist circumference was assessed at baseline, months 3, 6 and 12 by a digital weighing scale and tape meter. | Less than 50% attrition at 12-month follow-up and less than 20% attrition per group at 6-month follow-up. | "Contamination of interventions could occur because participants in each setting were randomly assigned to the two groups, and they were aware of their groups".  "The program providers shifted between individual behavior therapy and group behavior therapy every other week in order to eliminate the possible personal differences in providing the interventions." |
| **155** | **Weinstock 2013** | UNCLEAR | UNCLEAR | LOW | LOW |  |
|  | Assessment justification: | NS | NS | "At baseline, 6 months, 1 and 2 years, a research nurse performed standardized assessments at the practice sites and measured height, weight..." | Percentage of sample followed up:  6-months:  CC: 71%; IC: 65% 1 Year:  CC: 62%; IC: 57% 2 Years:  CC: 56%; IC: 48%  3 year:  Total sample: 51.4% |  |
| **156** | **West 2007** | UNCLEAR | LOW | LOW | LOW |  |
|  | Assessment justification: | NS | "women were randomized using a sequentially numbered, closed-envelope procedure." | "All assessments were conducted by trained interviewers blind to experimental condition. Body weight was measured without shoes using a calibrated balance beam scale." | Less than 20% and 50% of attrition at mid- and longer follow-ups. |  |
| **157** | **West 2011** | LOW | UNCLEAR | LOW | LOW |  |
|  | Assessment justification: | "Senior centers were randomized by computer-generated random numbers to either a Lifestyle weight-loss program or to a cognitive training program designed to serve as an attention control, matched in contact time, duration, and structure." | NS | "Body weight was measured in street clothes without shoes using a calibrated digital scale (Tanita BWB 800)..." | "Follow-up assessments were conducted with 211 older adults (93%) at 4 months."  4-months:  Intervention: 106/116*100 = 91.4%; Control:  96/112*100 = 85.7%  12-months:  overall retention rate was 86% and there was no difference between arms. |  |
| **158** | **Whelton 1998** | UNCLEAR | UNCLEAR | LOW | HIGH | HIGH |
|  | Assessment justification: | "Overweight participants are randomly assigned, in a 2 x 2 factorial design, to one of the following four groups..." "Using a computer program, each participant’s eligibility was confirmed prior to enrollment in the trial. Randomization was stratified by clinic and weight status to provide an even distribution of participants among the treatment groups at each site, and blocking of variable length (2, 4, and 8) was used to ensure temporal balance." | NS | "Detailed information was collected at baseline, and an interval medical history (including medication information and symptoms) and measurements of body weight and BP were obtained quarterly." | The protocol clearly states consistent follow-up for 36-months, however the primary paper describes an average follow-up for the 585 patients in the weight loss/no weight loss groups.  “Study data were collected at the 4 eligibility and randomization contacts and at quarterly visits during follow-up from August 1992 until December 1995. Follow-up ranged from 15 to 36 months (median, 29 months)." The number of participants followed up for each of the groups of interest at each time point in unclear. | Weight change data is only reported as two of the four groups, combined as follows: the non-weight loss group (Sodium Reduction and Usual Care) and the Weight loss group (Weight loss intervention and combined [sodium intervention and weight loss intervention). |
| **159** | **Wilson 2016** | UNCLEAR | UNCLEAR | LOW | LOW | UNCLEAR |
|  | Assessment justification: | Randomization of worksites into conditions.  No further information given. | NS | Weight objectively measured. | Control: 147/234; Phone 165/233;  Group 106/182  “drop out of approximately 40% of enrolled participants” | No indication that results were adjusted for clustering, and one worksite per condition |
| **160** | **Wilson 2016b** | UNCLEAR | UNCLEAR | LOW | HIGH | HIGH |
|  | Assessment justification: | “Six sites were matched based on the number of employees and randomly assigned to treatment or control groups.' 'Randomization of worksites into intervention or control groups” | NS | Weight objectively measured.  Self-reported secondary outcomes (e.g. food intake and physical activity). | 199 participants in the intervention sites and 46 participants at the control sites did not complete additional measures (post-test or follow-up) and were excluded from the ﬁnal analyses. At the post-test, 236 participated in the intervention (39 who joined after baseline) and 359 participated in the control (52 who joined after baseline). At follow-up, 136 participants in the intervention group and 211 in the control group completed the surveys and measures. Removing the one control group from the ﬁnal analysis because of contamination resulted in 227 (49.5%) participants in the ﬁnal intervention and 135 (69.9%) in the ﬁnal control cohort for outcome analyses.  “LGM analyses controlled for group differences by examining change over time and maximized the number of participants in the ﬁnal cohort.”  “LGM analyses that controlled for group differences by examining change over time and maximized the number of participants in the ﬁnal cohort. “ “Limitations of the study included ...dropout of approximately half of the participants from the study; ... LGM analyses, which enable imputation of data based on two data points “ | 'An additional 39 employees at the intervention sites and 76 employees from the control sites joined the study prior to the post-test, resulting in 236 and 359 participants respectively at post-test.'? 'Need to include all interested participants regardless of risk status, which likely diluted the impact.'  '204 participants were excluded from analysis due to site deviation from protocol.' ('One control group experienced what Cook and Campbell referred to as compensatory rivalry. Contrary to study protocols, the site coordinator initiated a variety of intervention strategies (i.e., biggest loser contest, motivational interviewing sessions, group educational sessions) to ‘‘make their site look better,’’ according to an interview that was conducted with the site coordinator. This created a threat to the internal validity of the study, and as a result the site was removed from the ﬁnal analyses. It could not be included as part of the treatment condition, as the strategies used differed from the planned intervention.') |
| **161** | **Wing 1988** | UNCLEAR | UNCLEAR | LOW | LOW | UNCLEAR |
|  | Assessment justification: | NS | NS | Weight was measured in street clothes, without shoes, using a balance beam scale. | Attrition less than 20% at program's end and less than 50% at 1-year follow-up | Participants have been omitted from the analysis both at baseline and 10-week program end. |
| **162** | **Wing 1988b** | UNCLEAR | UNCLEAR | LOW | LOW |  |
|  | Assessment justification: | NS | NS | Weight was measured in street clothes, without shoes, using a balance beam scale. | Less than 20% attrition at post-intervention follow-up and less than 50% of attrition at 1-year follow-up. |  |
| **163** | **Wing 1998** | UNCLEAR | UNCLEAR | LOW | LOW |  |
|  | Assessment justification: | NS | NS | Weight objectively measured. | Less than 50% attrition at 12-month follow-up. |  |
| **164** | **Wing 2010** | LOW | LOW | LOW | LOW |  |
|  | Assessment justification: | "Randomization was performed with the use of randomly permuted blocks of three or six, stratified according to clinical center, with random assignment concealed in tamper-proof envelopes." | Refer to ‘Random sequence generation (selection bias)’. | Weight objectively measured. | "Assessments were attended by 94%, 90% and 86% of women at 6, 12 and 18 months, respectively, with no significant differences between treatment groups." |  |
| **165** | **Yannakoulia 2008** | UNCLEAR | UNCLEAR | UNCLEAR | HIGH | UNCLEAR |
|  | Assessment justification: | NS | NS | NS | Fifty percent of patients were dropouts. Comparisons between completers and dropouts revealed no statistically significant differences between the two groups (with regards to history of diabetes, sex, HbA1c, BMI or waist circumference), apart from their age, with those not completing the intervention being younger compared to completers (53 ± 9 vs. 60 ± 9 yr, p = 0.05) (Table 2). A trend for an association between group and dropout was observed: 66.7% in the UC and 33.3% in the IC were dropouts (p = 0.07).  To explore the effect of several factors in relation to the likelihood of being a dropout, a logistic regression was performed. Older people (p = 0.03) and those with newly diagnosed T2DM (p = 0.05) were more likely to complete the program, whereas a tendency for a negative association between attendance of the IC group and the likelihood of dropping out was found (p = 0.08). | "They were also informed about smoking risks and encouraged to stop or limit smoking" but no information on how many smoked and if smoking behaviour changed and no other mentions of smoking in the paper. This could be a potential confounder but without this information difficult to assess. |
| **166** | **Yardley 2014** | LOW | LOW | HIGH | LOW | HIGH |
|  | Assessment justification: | “Participants were then automatically randomised to one of the four groups by a computer algorithm that employed stratification by waist (allocating to the lower weight group if waist < 88 cm for women, < 102 cm for men), and a block size of 60 within each practice.” | “The computer system immediately informed participants which group they had been allocated to, and sent an email to inform the practice nurse.” | Intention was to weigh all participants in practice but due to low levels of attendance, self-report measures were completed at 12 months by a little less than half the sample. | Majority of participants followed-up at 12m | Two practices deviated from protocol by providing considerable weight management support to their usual care patients. Having detected substantial deviations from trial protocol in two practices, these analyses were repeated for the three practices that had followed the protocol correctly. We therefore carried out additional analyses of outcomes in the three practices that had followed protocol by not offering additional nurse support to those in the usual care group (see per protocol analyses below). Even in the per protocol practices, the level of nurse contact was somewhat less than intended, especially in the regular nurse support group, and the level of phone and email contact was very low. There was a skewed distribution of nurse support. Using our revised follow-up procedures, the follow-up for the primary outcome was increased slightly to 68.7% at 12 months, but the proportion having blood tests dropped to 36.9%. |
| **167** | **Yates 2018** | UNCLEAR | UNCLEAR | LOW | HIGH |  |
|  | Assessment justification: | NS | NS | Weight objectively measured. | 7/8 no lifestyle and 4/7 lifestyle followed up at one year. |  |
| **168** | **Yeh 2003** | LOW | LOW | LOW | HIGH |  |
|  | Assessment justification: | Randomly assigned to one of two behavioral interventions using SAS software. Assignment to treatment group was made by a data analyst who had no contact with study subjects. | Subjects and investigators neither knew of treatment assignment in advance of eligibility assessment nor exercised any control over treatment assignment. | All outcome measures were collected by research assistants blinded to subjects’ treatment assignment. | CBI  At 6mths 25/37. LOW. At 12mths 14/37. HIGH.  At 24mths 14/37. HIGH SBI At 6mths 24/35. LOW At 12mths 14/35. HIGH.  At 24mths 13/35. HIGH  “Of the 80 women randomly assigned, 72 (90%) women were available for baseline assessments, 49 (61.3%). remained at the 6-month assessment, 28 (35%) at 1 y and 27 (33.8%) at the end of the 2-y period. Of the subjects who returned at the 2y follow-up, 14 (51.9%) were from the CBI (control) group and 13 (48.1%) were from the SBI (intervention) group. The proportion of dropouts did not differ significantly between the groups at the 2-y follow-up (chi2, P = 0.95). t-Test analysis revealed that those who returned for follow-up and those who were lost to follow-up did not differ significantly with regard to age, baseline BMI, baseline weight, or baseline nutritional variables including total calories consumed, total fat, percent fat, and cholesterol intakes (data not shown).' |  |
| **169** | **Yin 2018** | LOW | UNCLEAR | LOW | LOW |  |
|  | Assessment justification: | Participants were randomised in blocks of 10, using a randomization table by the study statistician. Enrollment and randomization were performed by trained research staff. | NS | Trained research staff measured the participant’s weight, height and waist circumference with light clothes twice and the average was used. Participant’s weight was recorded at each meeting. | Less than 50% of attrition (at 12 months, 19 int. and 5 cont. loss-to-follow-up) |  |
| **170** | **Zhang 2016** | LOW | UNCLEAR | LOW | LOW |  |
|  | Assessment justification: | The randomization schedules were generated using SAS PROC PLAN in SAS statistical software (SAS Institute Inc) and concealed until an eligible participant was ready for enrollment. | NS | Weight measured objectively. | Of 220, 211 (95.9%) completed the 6-month follow-up visit, and 208 (94.5%) completed the 12-month follow-up visit. ITT was followed, undertaking MCMC imputation method. |  |
| **BP:** Blood pressure; **HDL-C:** High density lipoprotein cholesterol; **Mths:** Months; **NS:** Not specified; **PR**: Pulse rate; **RCT**: Randomised controlled trial; **Wk/s**: week/s; **Yr/s:** Year/s.  ^Study only included in the CNMA involving intensity components. | | | | | | |

## **Supplemental Table S3.** Included studies reference list

| **Study ID** | **Primary reference** |
| --- | --- |
| Abed 2013 | Abed HS, Wittert GA, Leong DP, et al. Effect of weight reduction and cardiometabolic risk factor management on symptom burden and severity in patients with atrial fibrillation: a randomized clinical trial. *JAMA* 2013; **310**(19): 2050-60. |
| Ackermann 2011 | Ackermann RT, Finch EA, Caffrey HM, Lipscomb ER, Hays LM, Saha C. Long-term effects of a community-based lifestyle intervention to prevent type 2 diabetes: the DEPLOY extension pilot study. *Chronic Illn* 2011; **7**(4): 279-90. |
| Agras 1990 | Agras WS, Taylor CB, Feldman DE, Losch M, Burnett KF. Developing computer-assisted therapy for the treatment of obesity. *Behavior Therapy* 1990; **21**(1): 99-109 |
| Ahern 2017 | Ahern AL, Wheeler GM, Aveyard P, et al. Extended and standard duration weight-loss programme referrals for adults in primary care (WRAP): a randomised controlled trial. *Lancet* 2017; **389**(10085): 2214-25 |
| Almanza -Aguilera 2018 | Almanza-Aguilera E, Brunius C, Bernal-Lopez MR, et al. Impact in Plasma Metabolome as Effect of Lifestyle Intervention for Weight-Loss Reveals Metabolic Benefits in Metabolically Healthy Obese Women. *J Proteome Res* 2018; **17**(8): 2600-10. |
| Anderson 2014 | Anderson AS, Craigie AM, Caswell S, et al. The impact of a bodyweight and physical activity intervention (BeWEL) initiated through a national colorectal cancer screening programme: randomised controlled trial. *BMJ* 2014; **348**: g1823. |
| Annesi 2016 | Annesi JJ, Johnson PH, Tennant GA, Porter KJ, McEwen KL. Weight Loss and the Prevention of Weight Regain: Evaluation of a Treatment Model of Exercise Self-Regulation Generalizing to Controlled Eating. *Perm J* 2016; **20**(3): 15-146. |
| Annesi 2017 | Annesi JJ. Mediation of the relationship of behavioural treatment type and changes in psychological predictors of healthy eating by body satisfaction changes in women with obesity. *Obes Res Clin Pract* 2017; **11**(1): 97-107. |
| Appel 2011 | Appel LJ, Clark JM, Yeh HC, et al. Comparative effectiveness of weight-loss interventions in clinical practice. *N Engl J Med* 2011; **365**(21): 1959-68. |
| Ard 2018 | Ard JD, Gower B, Hunter G, et al. Effects of Calorie Restriction in Obese Older Adults: The CROSSROADS Randomized Controlled Trial. *J Gerontol A Biol Sci Med Sci* 2017; **73**(1): 73-80 |
| Ash 2006 | Ash S, Reeves M, Bauer J, et al. A randomised control trial comparing lifestyle groups, individual counselling and written information in the management of weight and health outcomes over 12 months.  *Int J Obes (Lond)* 2006; **30**(10): 1557-64. |
| Ashley 2001 | Ashley JM, St Jeor ST, Perumean-Chaney S, Schrage J, Bovee V. Meal replacements in weight intervention. *Obes Res* 2001; **9 Suppl 4**: 312s-20s. |
| Ashley 2007 | Ashley JM, Herzog H, Clodfelter S, Bovee V, Schrage J, Pritsos C. Nutrient adequacy during weight loss interventions: a randomized study in women comparing the dietary intake in a meal replacement group with a traditional food group. *Nutr J.* 2007; **6**: 12. |
| Aveyard 2016 | Aveyard P, Lewis A, Tearne S, et al. Screening and brief intervention for obesity in primary care: a parallel, two-arm, randomised trial. *Lancet* 2016; **388**(10059): 2492-500. |
| Bacon 2002 | Bacon L, Keim NL, Van Loan MD, et al. Evaluating a 'non-diet' wellness intervention for improvement of metabolic fitness, psychological well-being and eating and activity behaviors. *Int J Obes Relat Metab Disord* 2002; **26**(6): 854-65. |
| Bartels 2015 | Bartels SJ, Pratt SI, Aschbrenner KA, et al. Pragmatic replication trial of health promotion coaching for obesity in serious mental illness and maintenance of outcomes. *Am J Psychiatry* 2015; **172**(4): 344-52. |
| Beeken 2017 | Beeken RJ, Leurent B, Vickerstaff V, et al. A brief intervention for weight control based on habit-formation theory delivered through primary care: results from a randomised controlled trial. *Int J Obes (Lond)* 2017; **41**(2): 246-54. |
| Bennett 2012 | Bennett GG, Warner ET, Glasgow RE, et al. Obesity treatment for socioeconomically disadvantaged patients in primary care practice. *Arch Intern Med* 2012; **172**(7): 565-74. |
| Bennett 2013 | Bennett GG, Foley P, Levine E, et al. Behavioral treatment for weight gain prevention among black women in primary care practice: a randomized clinical trial. *JAMA Intern Med* 2013; **173**(19): 1770-7. |
| Berry 2014 | Berry DC, Schwartz TA, McMurray RG, et al. The family partners for health study: a cluster randomized controlled trial for child and parent weight management. *Nutr Diabetes* 2014; **4**(1): e101. |
| Bertz 2012 | Bertz F, Brekke HK, Ellegård L, Rasmussen KM, Wennergren M, Winkvist A. Diet and exercise weight-loss trial in lactating overweight and obese women. *Am J Clin Nutr 2012;* **96**(4): 698-705. |
| Beutel 2006 | Beutel ME, Dippel A, Szczepanski M, Thiede R, Wiltink J. Mid-term effectiveness of behavioral and psychodynamic inpatient treatments of severe obesity based on a randomized study. *Psychother Psychosom* 2006; **75**(6): 337-45. |
| Bliddal 2011 | Bliddal H, Leeds AR, Stigsgaard L, Astrup A, Christensen R. Weight loss as treatment for knee osteoarthritis symptoms in obese patients: 1-year results from a randomised controlled trial. *Ann Rheum Dis* 2011; **70**(10): 1798-803. |
| Bo 2007 | Bo S, Ciccone G, Baldi C, et al. Effectiveness of a lifestyle intervention on metabolic syndrome. A randomized controlled trial. *J Gen Intern Med* 2007; **22**(12): 1695-703. |
| Brown 2014 | Brown C, Goetz J, Hamera E, Gajewski B. Treatment response to the RENEW weight loss intervention in schizophrenia: impact of intervention setting. *Schizophr Res* 2014; **159**(2-3): 421-5. |
| Burke 2015 | Burke LE, Ewing LJ, Ye L, et al. The SELF trial: A self-efficacy-based behavioral intervention trial for weight loss maintenance. *Obesity (Silver Spring)* 2015; **23**(11): 2175-82. |
| Chee 2017 | Chee WSS, Gilcharan Singh HK, Hamdy O, et al. Structured lifestyle intervention based on a trans-cultural diabetes-specific nutrition algorithm (tDNA) in individuals with type 2 diabetes: a randomized controlled trial. *BMJ Open Diabetes Res Care* 2017; **5**(1): e000384. |
| Cheyette 2007 | Cheyette C. Weight No More: a randomised controlled trial for people with type 2 diabetes on insulin therapy. *Pract Diab Int* 2007; **24**(9): 450-6. |
| Christensen 2012 | Christensen JR, Overgaard K, Carneiro IG, Holtermann A, Søgaard K. Weight loss among female health care workers--a 1-year workplace based randomized controlled trial in the FINALE-health study. *BMC Public Health* 2012; **12**: 625. |
| Cleo 2018 | Cleo G, Glasziou P, Beller E, Isenring E, Thomas R. Habit-based interventions for weight loss maintenance in adults with overweight and obesity: a randomized controlled trial. *Int J Obes (Lond)* 2019; **43**(2): 374-83. |
| Cole 2013 | Cole RE, Boyer KM, Spanbauer SM, Sprague D, Bingham M. Effectiveness of prediabetes nutrition shared medical appointments: prevention of diabetes. *Diabetes Educ* 2013; **39**(3): 344-53. |
| Conroy 2015 | Conroy MB, Sward KL, Spadaro KC, et al. Effectiveness of a physical activity and weight loss intervention for middle-aged women: healthy bodies, healthy hearts randomized trial. *J Gen Intern Med* 2015; **30**(2): 207-13. |
| Cooper 2010 | Cooper Z, Doll HA, Hawker DM, et al. Testing a new cognitive behavioural treatment for obesity: A randomized controlled trial with three-year follow-up. *Behav Res Ther* 2010; **48**(8): 706-13. |
| Cousins 1992 | Cousins JH, Rubovits DS, Dunn JK, Reeves RS, Ramirez AG, Foreyt JP. Family versus individually oriented intervention for weight loss in Mexican American women. *Public Health Rep* 1992; **107**(5): 549-55. |
| Craighead 1989 | Craighead LW, Blum MD. Supervised exercise in behavioral treatment for moderate obesity. *Behavior Therapy* 1989; **20**(1): 49-59. |
| Crowley 2017 | Crowley MJ, Edelman D, Voils CI, et al. Jump starting shared medical appointments for diabetes with weight management: Rationale and design of a randomized controlled trial. *Contemp Clin Trials* 2017; **58**: 1-12. |
| Dale 2009 | Dale KS, Mann JI, McAuley KA, Williams SM, Farmer VL. Sustainability of lifestyle changes following an intensive lifestyle intervention in insulin resistant adults: Follow-up at 2-years. *Asia Pac J Clin Nutr* 2009; **18**(1): 114-20. |
| Dalziel 2006 | Dalziel K, Segal L, de Lorgeril M. A mediterranean diet is cost-effective in patients with previous myocardial infarction. *J Nutr* 2006; **136**(7): 1879-85. |
| Damschroder 2014 | Damschroder LJ, Lutes LD, Kirsh S, et al. Small-changes obesity treatment among veterans: 12-month outcomes. *Am J Prev Med* 2014; **47**(5): 541-53. |
| Daubenmier 2016^ | Daubenmier J, Moran PJ, Kristeller J, et al. Effects of a mindfulness-based weight loss intervention in adults with obesity: A randomized clinical trial. *Obesity (Silver Spring)* 2016; **24**(4): 794-804. |
| Delahanty 2015 | Delahanty LM, Dalton KM, Porneala B, et al. Improving diabetes outcomes through lifestyle change--A randomized controlled trial. *Obesity (Silver Spring)* 2015; **23**(9): 1792-9. |
| deRoon 2017 | de Roon M, van Gemert WA, Peeters PH, Schuit AJ, Monninkhof EM. Long-term effects of a weight loss intervention with or without exercise component in postmenopausal women: A randomized trial. *Prev Med Rep* 2017; **5**: 118-23. |
| deVos 2016 | de Vos BC, Runhaar J, van Middelkoop M, Krul M, Bierma-Zeinstra SM. Long-term effects of a randomized, controlled, tailor-made weight-loss intervention in primary care on the health and lifestyle of overweight and obese women. *Am J Clin Nutr* 2016; **104**(1): 33-40. |
| Diabetes Prevention Program R G 2009 | Knowler WC, Fowler SE, Hamman RF, et al. 10-year follow-up of diabetes incidence and weight loss in the Diabetes Prevention Program Outcomes Study. *Lancet* 2009; **374**(9702): 1677-86. |
| Djuric 2002 | Djuric Z, DiLaura NM, Jenkins I, et al. Combining weight-loss counseling with the weight watchers plan for obese breast cancer survivors. *Obes Res* 2002; **10**(7): 657-65. |
| Duncan 2016 | Duncan S, Goodyear-Smith F, McPhee J, Zinn C, Grøntved A, Schofield G. Family-centered brief intervention for reducing obesity and cardiovascular disease risk: A randomized controlled trial. *Obesity (Silver Spring)* 2016; **24**(11): 2311-8. |
| Eaton 2016 | Eaton CB, Hartman SJ, Perzanowski E, et al. A Randomized Clinical Trial of a Tailored Lifestyle Intervention for Obese, Sedentary, Primary Care Patients. *Ann Fam Med* 2016; **14**(4): 311-9. |
| Fahey 2018 | Fahey MC, Hare ME, Talcott GW, et al. Characteristics Associated With Participation in a Behavioral Weight Loss Randomized Control Trial in the U.S. Military. *Mil Med* 2019; **184**(3-4): e120-e6. |
| Fernandez-Ruiz 2018 | Fernández-Ruiz VE, Armero-Barranco D, Paniagua-Urbano JA, Sole-Agusti M, Ruiz-Sánchez A, Gómez-Marín J. Short-medium-long-term efficacy of interdisciplinary intervention against overweight and obesity: Randomized controlled clinical trial. *Int J Nurs Prac* 2018; **24**(6): e12690. |
| Finkelstein 2017 | Finkelstein EA, Tham KW, Haaland BA, Sahasranaman A. Applying economic incentives to increase effectiveness of an outpatient weight loss program (TRIO) - A randomized controlled trial. *Soc Sci Med (1982)* 2017; **185**: 63-70. |
| Fisher 2011 | Fisher G, Hyatt TC, Hunter GR, Oster RA, Desmond RA, Gower BA. Effect of diet with and without exercise training on markers of inflammation and fat distribution in overweight women. *Obesity (Silver Spring)* 2011; **19**(6): 1131-6. |
| Foley 2016 | Foley P, Steinberg D, Levine E, et al. Track: A randomized controlled trial of a digital health obesity treatment intervention for medically vulnerable primary care patients. *Contemp Clin Trials* 2016; **48**: 12-20. |
| Foreyt 1993 | Foreyt JP, Goodrick GK, Reeves RS, et al. Response of free-living adults to behavioral treatment of obesity: Attrition and compliance to exercise. *Behavior Therapy* 1993; **24**(4): 659-69. |
| Foster-Schubert 2012 | Foster-Schubert KE, Alfano CM, Duggan CR, et al. Effect of diet and exercise, alone or combined, on weight and body composition in overweight-to-obese postmenopausal women. *Obesity (Silver Spring)* 2012; **20**(8): 1628-38. |
| Freitas 2017 | Freitas PD, Ferreira PG, Silva AG, et al. The Role of Exercise in a Weight-Loss Program on Clinical Control in Obese Adults with Asthma. A Randomized Controlled Trial. *Am J Respir Crit Care Med* 2017; **195**(1): 32-42. |
| Fuller 2012 | Fuller NR, Lau NS, Denyer G, Caterson ID. A 12-month, randomised, controlled trial to examine the efficacy of the Korean diet in an Australian overweight and obese population - A follow up analysis. *Obes Res Clin Pract* 2012; **6**(4): e263-346. |
| Gold 2007 | Gold BC, Burke S, Pintauro S, Buzzell P, Harvey-Berino J. Weight loss on the web: A pilot study comparing a structured behavioral intervention to a commercial program. *Obesity (Silver Spring)* 2007; **15**(1): 155-64. |
| Goodwin 2014 | Goodwin PJ, Segal RJ, Vallis M, et al. Randomized trial of a telephone-based weight loss intervention in postmenopausal women with breast cancer receiving letrozole: the LISA trial. *J Clin Oncol* 2014; **32**(21): 2231-9. |
| Hakala 1993 | Hakala P, Karvetti RL, Rönnemaa T. Group vs. individual weight reduction programmes in the treatment of severe obesity--a five year follow-up study. *Int J Obes Relat Metab Disord* 1993; **17**(2): 97-102. |
| Hanson 1976 | Hanson RW, Borden BL, Hall SM, Hall RG. Use of programmed instruction in teaching self-management skills to overweight adults. *Behavior Therapy* 1976; **7**(3): 366-73. |
| Harrigan 2016 | Harrigan M, Cartmel B, Loftfield E, et al. Randomized Trial Comparing Telephone Versus In-Person Weight Loss Counseling on Body Composition and Circulating Biomarkers in Women Treated for Breast Cancer: The Lifestyle, Exercise, and Nutrition (LEAN) Study. *J Clin Oncol* 2016; **34**(7): 669-76. |
| Hunt 2014 | Hunt K, Wyke S, Gray CM, et al. A gender-sensitised weight loss and healthy living programme for overweight and obese men delivered by Scottish Premier League football clubs (FFIT): a pragmatic randomised controlled trial. *Lancet* 2014; **383**(9924): 1211-21. |
| Huseinovic 2016 | Huseinovic E, Bertz F, Leu Agelii M, Hellebö Johansson E, Winkvist A, Brekke HK. Effectiveness of a weight loss intervention in postpartum women: results from a randomized controlled trial in primary health care. *Am J Clin Nutr* 2016; **104**(2): 362-70. |
| Irwin 2003 | Irwin ML, Yasui Y, Ulrich CM, et al. Effect of exercise on total and intra-abdominal body fat in postmenopausal women: a randomized controlled trial. *JAMA* 2003; **289**(3): 323-30. |
| Jakicic 2011 | Jakicic JM, Otto AD, Lang W, et al. The effect of physical activity on 18-month weight change in overweight adults. *Obesity (Silver Spring)* 2011; **19**(1): 100-9. |
| Jakicic 2015 | Jakicic JM, Rickman AD, Lang W, et al. Time-based physical activity interventions for weight loss: a randomized trial. *Med Sci Sports Exerc* 2015; **47**(5): 1061-9. |
| Jebb 2011 | Jebb SA, Ahern AL, Olson AD, et al. Primary care referral to a commercial provider for weight loss treatment versus standard care: a randomised controlled trial. *Lancet* 2011; **378**(9801): 1485-92. |
| Jebb 2017 | Jebb SA, Astbury NM, Tearne S, Nickless A, Aveyard P. Doctor Referral of Overweight People to a Low-Energy Treatment (DROPLET) in primary care using total diet replacement products: a protocol for a randomised controlled trial. *BMJ open* 2017; **7**(8): e016709. |
| Jeffery 2003 | Jeffery RW, Wing RR, Sherwood NE, Tate DF. Physical activity and weight loss: does prescribing higher physical activity goals improve outcome? *Am J Clin Nutr* 2003; **78**(4): 684-9. |
| Jolly 2011 | Jolly K, Lewis A, Beach J, et al. Comparison of range of commercial or primary care led weight reduction programmes with minimal intervention control for weight loss in obesity: lighten Up randomised controlled trial. *BMJ* 2011; **343**: d6500. |
| Jones 1999 | Jones DW, Miller ME, Wofford MR, et al. The effect of weight loss intervention on antihypertensive medication requirements in the hypertension Optimal Treatment (HOT) study. *Am J Hypertens* 1999; **12**(12 Pt 1-2): 1175-80. |
| Katula 2013 | Katula JA, Vitolins MZ, Morgan TM, et al. The Healthy Living Partnerships to Prevent Diabetes study: 2-year outcomes of a randomized controlled trial. *Am J Prev Med* 2013; **44**(4 Suppl 4): S324-32. |
| Keogh 2014 | Keogh JB, Pedersen E, Petersen KS, Clifton PM. Effects of intermittent compared to continuous energy restriction on short-term weight loss and long-term weight loss maintenance. *Clin Obes* 2014; **4**(3): 150-6. |
| Keranen 2009 | Keränen AM, Savolainen MJ, Reponen AH, et al. The effect of eating behavior on weight loss and maintenance during a lifestyle intervention. *Prev Med* 2009; **49**(1): 32-8. |
| King 1989 | King AC, Frey-Hewitt B, Dreon DM, Wood PD. Diet vs exercise in weight maintenance. The effects of minimal intervention strategies on long-term outcomes in men. *Arch Intern Med* 1989; **149**(12): 2741-6. |
| Knauper 2018 | Knäuper B, Carrière K, Frayn M, et al. The Effects of If-Then Plans on Weight Loss: Results of the McGill CHIP Healthy Weight Program Randomized Controlled Trial. *Obesity (Silver Spring)* 2018; **26**(8): 1285-95. |
| Kumanyika 2012 | Kumanyika SK, Fassbender JE, Sarwer DB, et al. One-year results of the Think Health! study of weight management in primary care practices. *Obesity (Silver Spring)* 2012; **20**(6): 1249-57. |
| Leahey 2014 | Leahey TM, Thomas G, Fava JL, et al. Adding evidence-based behavioral weight loss strategies to a statewide wellness campaign: a randomized clinical trial. *Am J Public Health* 2014; **104**(7): 1300-6. |
| Leahey 2015 | Leahey TM, Subak LL, Fava J, et al. Benefits of adding small financial incentives or optional group meetings to a web-based statewide obesity initiative. *Obesity (Silver Spring)* 2015; **23**(1): 70-6. |
| Lejeune 2003 | Lejeune MP, Van Aggel-Leijssen DP, Van Baak MA, Westerterp-Plantenga MS. Effects of dietary restraint vs exercise during weight maintenance in obese men. *Eur J Clin Nutr* 2003; **57**(10): 1338-44. |
| Ley 2004 | Ley SJ, Metcalf PA, Scragg RK, Swinburn BA. Long-term effects of a reduced fat diet intervention on cardiovascular disease risk factors in individuals with glucose intolerance. *Diabetes Res Clin Pract* 2004; **63**(2): 103-12. |
| Li 2016 | Li X, Cai X, Ma X, et al. Short- and Long-Term Effects of Wholegrain Oat Intake on Weight Management and Glucolipid Metabolism in Overweight Type-2 Diabetics: A Randomized Control Trial. *Nutrients* 2016; **8**(9). |
| Li 2005 | Li Z, Hong K, Saltsman P, et al. Long-term efficacy of soy-based meal replacements vs an individualized diet plan in obese type II DM patients: relative effects on weight loss, metabolic parameters, and C-reactive protein. *Eur J Clin Nutr* 2005; **59**(3): 411-8 |
| Lindstrom 2003 | Lindström J, Louheranta A, Mannelin M, et al. The Finnish Diabetes Prevention Study (DPS): Lifestyle intervention and 3-year results on diet and physical activity. *Diabetes care* 2003; **26**(12): 3230-6. |
| Liss 2016 | Liss DT, Finch EA, Gregory DL, Cooper A, Ackermann RT. Design and participant characteristics for a randomized effectiveness trial of an intensive lifestyle intervention to reduce cardiovascular risk in adults with type 2 diabetes: The I-D-HEALTH study. *Contemp Clin Trials* 2016; **46**: 114-21. |
| Little 2016 | Little P, Stuart B, Hobbs FR, et al. An internet-based intervention with brief nurse support to manage obesity in primary care (POWeR+): a pragmatic, parallel-group, randomised controlled trial. *Lancet Diabetes Endocrinol* 2016; **4**(10): 821-8. |
| Lowe 2018 | Lowe MR, Butryn ML, Zhang F. Evaluation of meal replacements and a home food environment intervention for long-term weight loss: a randomized controlled trial. *Am J Clin Nutr* **107**(1): 12-9. |
| Ma 2015 | Ma J, Strub P, Xiao L, et al. Behavioral weight loss and physical activity intervention in obese adults with asthma. A randomized trial. *Ann Am Thorac Soc* 2015; **12**(1): 1-11. |
| Manning 1994 | Manning RM, Jung RT, Leese GP, Newton RW. The comparison of four weight reduction strategies aimed at overweight diabetic patients. *Diabet Med*; **12**(5): 409-15. |
| Manzoni 2016 | Manzoni GM, Cesa GL, Bacchetta M, et al. Virtual Reality-Enhanced Cognitive-Behavioral Therapy for Morbid Obesity: A Randomized Controlled Study with 1 Year Follow-Up. *Cyberpsychol Behav Soc Netw* 2016; **19**(2): 134-40. |
| Marniemi 1990 | Marniemi J, Seppänen A, Hakala P. Long-term effects on lipid metabolism of weight reduction on lactovegetarian and mixed diet. *Inter J Obes* 1990; **14**(2): 113-25. |
| Martin 2008 | Martin PD, Dutton GR, Rhode PC, Horswell RL, Ryan DH, Brantley PJ. Weight loss maintenance following a primary care intervention for low-income minority women. *Obesity (Silver Spring)* 2008; **16**(11): 2462-7. |
| Mefferd 2007 | Mefferd K, Nichols JF, Pakiz B, Rock CL. A cognitive behavioral therapy intervention to promote weight loss improves body composition and blood lipid profiles among overweight breast cancer survivors. *Breast Cancer Res Treat* 2007; **104**(2): 145-52. |
| Mengham 1999 | Mengham L, Morris B, Palmer C, White A. Is intensive dietetic intervention effective for overweight patients with diabetes mellitus? A randomized controlled study in a general practice. *Pract Diab Int* 1999; **16**(1): 5-8. |
| Messier 2013 | Messier SP, Mihalko SL, Legault C, et al. Effects of intensive diet and exercise on knee joint loads, inflammation, and clinical outcomes among overweight and obese adults with knee osteoarthritis: the IDEA randomized clinical trial. *JAMA* 2013; **310**(12): 1263-73. |
| Miller 2002 | Miller ER, 3rd, Erlinger TP, Young DR, et al. Results of the Diet, Exercise, and Weight Loss Intervention Trial (DEW-IT). *Hypertension* 2002; **40**(5): 612-8. |
| Mitsui 2008 | Mitsui T, Shimaoka K, Tsuzuku S, Kajioka T, Sakakibara H. Gentle exercise of 40 minutes with dietary counseling is effective in treating metabolic syndrome. *Tohoku J Exp Med* 2008; **215**(4): 355-61 |
| Molenaar 2010 | Molenaar EA, van Ameijden EJ, Vergouwe Y, Grobbee DE, Numans ME. Effect of nutritional counselling and nutritional plus exercise counselling in overweight adults: a randomized trial in multidisciplinary primary care practice. *Fam Pract* 2010; **27**(2): 143-50. |
| Moreno 2014 | Moreno B, Bellido D, Sajoux I, et al. Comparison of a very low-calorie-ketogenic diet with a standard low-calorie diet in the treatment of obesity. *Endocrine* 2014; **47**(3): 793-805. |
| Morgan 2010 | Morgan PJ, Lubans DR, Collins CE, Warren JM, Callister R. 12-month outcomes and process evaluation of the SHED-IT RCT: an internet-based weight loss program targeting men. *Obesity (Silver Spring)* 2011; **19**(1): 142-51. |
| Muggia 2014 | Muggia C, Falchi AG, Michelini I, et al. Brief group cognitive behavioral treatment in addition to prescriptive diet versus standard care in obese and overweight patients. A randomized controlled trial. *e-SPEN* 2014; **9**(1): e26-e33. |
| Ng 2015 | Ng SSS, Chan RSM, Woo J, et al. A Randomized Controlled Study to Examine the Effect of a Lifestyle Modification Program in OSA. *Chest* 2015; **148**(5): 1193-203. |
| Nicklas 2009 | Nicklas BJ, Wang X, You T, et al. Effect of exercise intensity on abdominal fat loss during calorie restriction in overweight and obese postmenopausal women: a randomized, controlled trial. *Am J Clin Nutr*; **89**(4): 1043-52. |
| Nilsen 2011 | Nilsen V, Bakke PS, Gallefoss F. Effects of lifestyle intervention in persons at risk for type 2 diabetes mellitus - results from a randomised, controlled trial. *BMC public health* 2011; **11**: 893. |
| Oldroyd 2006 | Oldroyd JC, Unwin NC, White M, Mathers JC, Alberti KG. Randomised controlled trial evaluating lifestyle interventions in people with impaired glucose tolerance. *Diabetes Res Clin Pract* 2006; **72**(2): 117-27. |
| Parikh 2010 | Parikh P, Simon EP, Fei K, Looker H, Goytia C, Horowitz CR. Results of a pilot diabetes prevention intervention in East Harlem, New York City: Project HEED. *Am J Public Health* 2010; **100 Suppl 1**(Suppl 1): S232-9. |
| Pedersen 2013 | Pedersen LR, Olsen RH, Frederiksen M, et al. Copenhagen study of overweight patients with coronary artery disease undergoing low energy diet or interval training: the randomized CUT-IT trial protocol. *BMC Cardiovasc Disord* 2013; **13**: 106. |
| Perri 1986 | Perri MG, McAdoo WG, McAllister DA, Lauer JB, Yancey DZ. Enhancing the efficacy of behavior therapy for obesity: effects of aerobic exercise and a multicomponent maintenance program. *J Consult Clin Psychol* 1986; **54**(5): 670-5. |
| Perri 1997 | Perri MG, Martin AD, Leermakers EA, Sears SF, Notelovitz M. Effects of group- versus home-based exercise in the treatment of obesity. *J Consult Clin Psychol* 1997; **65**(2): 278-85. |
| Perri 2001 | Perri MG, Nezu AM, McKelvey WF, Shermer RL, Renjilian DA, Viegener BJ. Relapse prevention training and problem-solving therapy in the long-term management of obesity. *J Consult Clin Psychol* 2001; **69**(4): 722-6. |
| Pettman 2009 | Pettman TL, Buckley JD, Misan GM, Coates AM, Howe PR. Health benefits of a 4-month group-based diet and lifestyle modification program for individuals with metabolic syndrome. *Obes Res Clin Pract* 2009; **3**(4): 221-35. |
| Poelman 2015 | Poelman MP, de Vet E, Velema E, de Boer MR, Seidell JC, Steenhuis IH. PortionControl@HOME: results of a randomized controlled trial evaluating the effect of a multi-component portion size intervention on portion control behavior and body mass index Ann Behav Med 2015; **49**(1): 18-28. |
| Promrat 2010 | Promrat K, Kleiner DE, Niemeier HM, et al. Randomized controlled trial testing the effects of weight loss on nonalcoholic steatohepatitis. *Hepatology* 2010; **51**(1): 121-9. |
| Provencher 2009 | Provencher V, Bégin C, Tremblay A, et al. Health-At-Every-Size and eating behaviors: 1-year follow-up results of a size acceptance intervention. *J Am Diet Assoc* 2009; **109**(11): 1854-61. |
| Rock 2015 | Rock CL, Flatt SW, Byers TE, et al. Results of the Exercise and Nutrition to Enhance Recovery and Good Health for You (ENERGY) Trial: A Behavioral Weight Loss Intervention in Overweight or Obese Breast Cancer Survivors. *J Clin Oncol*  2015; **33**(28): 3169-76. |
| Rolls 2005 | Rolls BJ, Roe LS, Beach AM, Kris-Etherton PM. Provision of foods differing in energy density affects long-term weight loss. *Obes Res* 2005; **13**(6): 1052-60. |
| Rolls 2017 | Rolls BJ, Roe LS, James BL, Sanchez CE. Does the incorporation of portion-control strategies in a behavioral program improve weight loss in a 1-year randomized controlled trial? *Int J Obes (Lond)* 2017; **41**(3): 434-42. |
| Rosas 2015 | Rosas LG, Thiyagarajan S, Goldstein BA, et al. The effectiveness of two community-based weight loss strategies among obese, low-income US Latinos. *J Acad Nutr Diet* 2015; **115**(4): 537-50.e2. |
| Ross 2012 | Ross R, Lam M, Blair SN, et al. Trial of prevention and reduction of obesity through active living in clinical settings: a randomized controlled trial. *Arch Intern Med* 2012; **172**(5): 414-24. |
| Samaras 1997 | Samaras K, Ashwell S, Mackintosh AM, Fleury AC, Campbell LV, Chisholm DJ. Will older sedentary people with non-insulin-dependent diabetes mellitus start exercising? A health promotion model. *Diabetes Res Clin Pract* 1997; **37**(2): 121-8. |
| Santanasto 2011 | Santanasto AJ, Glynn NW, Newman MA, et al. Impact of weight loss on physical function with changes in strength, muscle mass, and muscle fat infiltration in overweight to moderately obese older adults: a randomized clinical trial. *J Obes* 2011; **2011**. |
| Sattin 2016 | Sattin RW, Williams LB, Dias J, et al. Community Trial of a Faith-Based Lifestyle Intervention to Prevent Diabetes Among African-Americans. *J Community Health* 2016; **41**(1): 87-96. |
| Schubel 2016 | Schübel R, Graf ME, Nattenmüller J, et al. The effects of intermittent calorie restriction on metabolic health: Rationale and study design of the HELENA Trial. *Contemp Clin Trials* 2016; **51**: 28-33. |
| Seligman 2011 | Seligman BG, Polanczyk CA, Santos AS, et al. Intensive practical lifestyle intervention improves endothelial function in metabolic syndrome independent of weight loss: a randomized controlled trial. *Metabolism* 2011; **60**(12): 1736-40. |
| Shikany 2013 | Shikany JM, Thomas AS, Beasley TM, Lewis CE, Allison DB. Randomized controlled trial of the Medifast 5 & 1 Plan for weight loss. *Int J Obes (Lond)* 2013; **37**(12): 1571-8. |
| Silva 2010 | Silva MN, Vieira PN, Coutinho SR, et al. Using self-determination theory to promote physical activity and weight control: a randomized controlled trial in women. *J Behav Med* 2010; **33**(2): 110-22. |
| Solbrig 2019 | Solbrig L, Whalley B, Kavanagh DJ, et al. Functional imagery training versus motivational interviewing for weight loss: a randomised controlled trial of brief individual interventions for overweight and obesity. *Int J Obes (Lond)* 2019; **43**(4): 883-94. |
| Somers 2012 | Somers TJ, Blumenthal JA, Guilak F, et al. Pain coping skills training and lifestyle behavioral weight management in patients with knee osteoarthritis: a randomized controlled study. *Pain* 2012; **153**(6): 1199-209. |
| Spring 2013 | Spring B, Duncan JM, Janke EA, et al. Integrating technology into standard weight loss treatment: a randomized controlled trial. *JAMA Intern Med* 2013; **173**(2): 105-11. |
| Spring 2017 | Spring B, Pellegrini CA, Pfammatter A, et al. Effects of an abbreviated obesity intervention supported by mobile technology: The ENGAGED randomized clinical trial. *Obesity (Silver Spring)* 2017; **25**(7): 1191-8. |
| Stahre 2005 | Stahre L, Hällström T. A short-term cognitive group treatment program gives substantial weight reduction up to 18 months from the end of treatment. A randomized controlled trial. *Eat Weight Disord* 2005; **10**(1): 51-8. |
| Stenius-Aarniala 2000 | Stenius-Aarniala B, Poussa T, Kvarnström J, Grönlund EL, Ylikahri M, Mustajoki P. Immediate and long term effects of weight reduction in obese people with asthma: randomised controlled study. *BMJ* 2000; **320**(7238): 827-32. |
| Stevens 1993 | Stevens VJ, Corrigan SA, Obarzanek E, et al. Weight loss intervention in phase 1 of the Trials of Hypertension Prevention. The TOHP Collaborative Research Group. *Arch Intern Med* 1993; **153**(7): 849-58. |
| Stevens 2001 | Stevens VJ, Obarzanek E, Cook NR, et al. Long-term weight loss and changes in blood pressure: results of the Trials of Hypertension Prevention, phase II. *Ann Intern Med* 2001; **134**(1): 1-11. |
| Strobl 2013 | Ströbl V, Knisel W, Landgraf U, Faller H. A combined planning and telephone aftercare intervention for obese patients: effects on physical activity and body weight after one year. *J Rehabil Med* 2013; **45**(2): 198-205. |
| Sundfor 2018 | Sundfør TM, Svendsen M, Tonstad S. Effect of intermittent versus continuous energy restriction on weight loss, maintenance and cardiometabolic risk: A randomized 1-year trial. *Nutr Metab Cardiovasc Dis* 2018; **28**(7): 698-706. |
| Tapsell 2017 | Tapsell LC, Lonergan M, Batterham MJ, et al. Effect of interdisciplinary care on weight loss: a randomised controlled trial. *BMJ open* 2017; **7**(7): e014533. |
| TarragaMarcos 2017 | Tárraga Marcos ML, Panisello Royo JM, Carbayo Herencia JA, Rosich Domenech N, Alins Presas J, Tárraga López PJ. Effect on the lipid parameters of an intervention to reduce weight in overweight and obese patients. *Clin Investig Arterioscler* 2017; **29**(3): 103-10. |
| Teeriniemi 2018 | Teeriniemi AM, Salonurmi T, Jokelainen T, et al. A randomized clinical trial of the effectiveness of a Web-based health behaviour change support system and group lifestyle counselling on body weight loss in overweight and obese subjects: 2-year outcomes. *J Intern Med* 2018; **284**(5): 534-45. |
| ter Bogt 2009 | ter Bogt NC, Bemelmans WJ, Beltman FW, Broer J, Smit AJ, van der Meer K. Preventing weight gain: one-year results of a randomized lifestyle intervention. *Am J Prev Med* 2009; **37**(4): 270-7. |
| The Look AHEAD Research Group 2010 | Wing RR. Long-term effects of a lifestyle intervention on weight and cardiovascular risk factors in individuals with type 2 diabetes mellitus: four-year results of the Look AHEAD trial. *Arch Intern Med* 2010; **170**(17): 1566-75. |
| Trepanowski 2017 | Trepanowski JF, Kroeger CM, Barnosky A, et al. Effect of Alternate-Day Fasting on Weight Loss, Weight Maintenance, and Cardioprotection Among Metabolically Healthy Obese Adults: A Randomized Clinical Trial. *JAMA Intern Med* 2017; **177**(7): 930-8. |
| Tsai 2010 | Tsai AG, Wadden TA, Rogers MA, Day SC, Moore RH, Islam BJ. A primary care intervention for weight loss: results of a randomized controlled pilot study. *Obesity (Silver Spring)* 2010; **18**(8): 1614-8. |
| Tuomilehto 2009 | Tuomilehto HP, Seppä JM, Partinen MM, et al. Lifestyle intervention with weight reduction: first-line treatment in mild obstructive sleep apnea. *Am J Respir Crit Care Med* 2009; **179**(4): 320-7. |
| van de Glind 2017 | van de Glind I, Bunn C, Gray CM, et al. The intervention process in the European Fans in Training (EuroFIT) trial: a mixed method protocol for evaluation. *Trials* 2017; **18**(1): 356. |
| Viegener 1990 | Viegener BJ, Renjilian DA, McKelvey WF, Schein RL, Perri MG, Nezu AM. Effects of an intermittent, low-fat, low-calorie diet in the behavioral treatment of obesity. *Behavior Therapy* 1990; **21**(4): 499-509. |
| Vissers 2010 | Vissers D, Verrijken A, Mertens I, et al. Effect of long-term whole body vibration training on visceral adipose tissue: a preliminary report. *Obes Facts* 2010; **3**(2): 93-100. |
| Volpe 2008 | Volpe SL, Kobusingye H, Bailur S, Stanek E. Effect of diet and exercise on body composition, energy intake and leptin levels in overweight women and men. *J Am Coll Nutr* 2008 **27**(2): 195-208. |
| von Gruenigen 2012 | von Gruenigen V, Frasure H, Kavanagh MB, et al. Survivors of uterine cancer empowered by exercise and healthy diet (SUCCEED): a randomized controlled trial. *Gynecologic oncology* 2012; **125**(3): 699-704. |
| vonGruenigen 2008 | von Gruenigen VE, Courneya KS, Gibbons HE, Kavanagh MB, Waggoner SE, Lerner E. Feasibility and effectiveness of a lifestyle intervention program in obese endometrial cancer patients: a randomized trial. *Gynecol Oncol* 2008; **109**(1): 19-26. |
| Wadden 1994 | Wadden TA, Foster GD, Letizia KA. One-year behavioral treatment of obesity: comparison of moderate and severe caloric restriction and the effects of weight maintenance therapy. *J Consult Clin Psychol* 1994; **62**(1): 165-71. |
| Wadden 1998 | Wadden TA, Vogt RA, Foster GD, Anderson DA. Exercise and the maintenance of weight loss: 1-year follow-up of a controlled clinical trial. *J Consult Clin Psychol 1998;* **66**(2): 429-33. |
| Wadden 2004 | Wadden TA, Foster GD, Sarwer DB, et al. Dieting and the development of eating disorders in obese women: results of a randomized controlled trial. *Am J Clin Nutr* 2004; **80**(3): 560-8. |
| Waleekhachonloet 2007 | Waleekhachonloet OA, Limwattananon C, Limwattananon S, Gross CR. Group behavior therapy versus individual behavior therapy for healthy dieting and weight control management in overweight and obese women living in rural community. *Obes Res Clin Pract* 2007; **1**(4): 223-90. |
| Weinstock 2013 | Weinstock RS, Trief PM, Cibula D, Morin PC, Delahanty LM. Weight loss success in metabolic syndrome by telephone interventions: results from the SHINE Study. *J Gen Intern Med* 2013; **28**(12): 1620-8. |
| West 2007 | West DS, DiLillo V, Bursac Z, Gore SA, Greene PG. Motivational interviewing improves weight loss in women with type 2 diabetes. *Diabetes Care* 2007; **30**(5): 1081-7. |
| West 2011 | West DS, Bursac Z, Cornell CE, et al. Lay health educators translate a weight-loss intervention in senior centers: a randomized controlled trial. *Am J Prev Med* 2011; **41**(4): 385-91. |
| Whelton 1998 | Whelton PK, Appel LJ, Espeland MA, et al. Sodium reduction and weight loss in the treatment of hypertension in older persons: a randomized controlled trial of nonpharmacologic interventions in the elderly (TONE). TONE Collaborative Research Group. *JAMA* 1998; **279**(11): 839-46. |
| Wilson 2016 | Wilson MG, DeJoy DM, Vandenberg RJ, Corso P, Padilla H, Zuercher H. Effect of Intensity and Program Delivery on the Translation of Diabetes Prevention Program to Worksites: A Randomized Controlled Trial of Fuel Your Life. *J Occup Environ Med* 2016; **58**(11): 1113-20. |
| Wilson 2016b | Wilson MG, DeJoy DM, Vandenberg R, Padilla H, Davis M. FUEL Your Life: A Translation of the Diabetes Prevention Program to Worksites. *Am J Health Promot* 2016; **30**(3): 188-97. |
| Wing 1988 | Wing RR, Epstein LH, Paternostro-Bayles M, Kriska A, Nowalk MP, Gooding W. Exercise in a behavioural weight control programme for obese patients with Type 2 (non-insulin-dependent) diabetes. *Diabetologia* 1988; **31**(12): 902-9. |
| Wing 1988b | Wing RR, Epstein LH, Paternostro-Bayles M, Kriska A, Nowalk MP, Gooding W. Exercise in a behavioural weight control programme for obese patients with Type 2 (non-insulin-dependent) diabetes. *Diabetologia* 1988; **31**(12): 902-9. |
| Wing 1998 | Wing RR, Venditti E, Jakicic JM, Polley BA, Lang W. Lifestyle intervention in overweight individuals with a family history of diabetes. *Diabetes Care* 1998; **21**(3): 350-9. |
| Wing 2010 | Wing RR, West DS, Grady D, et al. Effect of weight loss on urinary incontinence in overweight and obese women: results at 12 and 18 months. *J Urol* 2010; **184**(3): 1005-10. |
| Yannakoulia 2008 | Yannakoulia M, Poulia KA, Mylona E, Kontogianni MD. Effectiveness of an intensive nutritional intervention in patients with type 2 diabetes mellitus: results from a pilot study. *Rev Diabet Stud* 2007; **4**(4): 226-30. |
| Yardley 2014 | Yardley L, Ware LJ, Smith ER, et al. Randomised controlled feasibility trial of a web-based weight management intervention with nurse support for obese patients in primary care. *Int J Behav Nutr Phys Act* 2014; **11**: 67. |
| Yates 2018 | Yates MS, Coletta AM, Zhang Q, et al. Prospective Randomized Biomarker Study of Metformin and Lifestyle Intervention for Prevention in Obese Women at Increased Risk for Endometrial Cancer. *Cancer Prev Res (Phila)* 2018; **11**(8): 477-90 |
| Yeh 2003 | Yeh MC, Rodriguez E, Nawaz H, Gonzalez M, Nakamoto D, Katz DL. Technical skills for weight loss: 2-y follow-up results of a randomized trial. *Int J Obes Relat Metab Disord* 2003; **27**(12): 1500-6. |
| Yin 2018 | Yin Z, Perry J, Duan X, et al. Cultural adaptation of an evidence-based lifestyle intervention for diabetes prevention in Chinese women at risk for diabetes: results of a randomized trial. *Int Health* 2018; **10**(5): 391-400. |
| Zhang 2016 | Zhang HJ, He J, Pan LL, et al. Effects of Moderate and Vigorous Exercise on Nonalcoholic Fatty Liver Disease: A Randomized Clinical Trial. *JAMA Internal Med* 2016; **176**(8): 1074-82. |
| ^Study only included in the CNMA involving intensity components. | |

## **Supplemental Table S4.** Baseline demographics

| **Study ID** | **Groups:** | **Randomised** | **Number of participants reported at baseline** | **Gender**  **(%F)** | **Age** | | **BMI** | | **Comorbidities at baseline (%) *** | | |
| --- | --- | --- | --- | --- | --- | --- | --- | --- | --- | --- | --- |
|  |  |  |  |  | ***Mean*** | ***SD*** | ***Mean*** | ***SD*** | ***CV morbidity*** | ***Type II DM*** | ***Hypertension*** |
| Abed 2013 | Control | 75 | 75 | 33 | 60.3 | 10.3 | 33.8 | 4.1 | 100 | 28 | 87 |
|  | Weight Management | 75 | 75 | 32 | 59.8 | 9.5 | 32.8 | 3.5 | 100 | 24 | 83 |
| Ackermann 2011 | Standard advice alone (controls) | 46 | 46 | 61 | 60.1 | 10.5 | 30.8 | 5.1 | NR | NR | NR |
|  | YMCA DPP intervention | 46 | 46 | 50 | 56.5 | 9.7 | 32.0 | 4.8 | NR | NR | NR |
| Agras 1990 | Computer alone | 30 | 30 | 100 | 45.2 | 12.4 | 29.7 | 4.3 | NR | NR | NR |
|  | Computer + group support | 30 | 30 |  |  |  |  |  | NR | NR | NR |
|  | Behaviour therapy | 30 | 30 |  |  |  |  |  | NR | NR | NR |
| Ahern 2017 | Brief intervention | 211 | 211 | 68 | 51.9 | 14.1 | 34.4 | 4.6 | NR | 13.5 | 49.8 |
|  | 12-week behavioural weight-loss programme | 530 | 528 | 68 | 53.6 | 13.3 | 34.7 | 5.4 |  |  |  |
|  | 52-week behavioural weight-loss programme | 528 | 528 | 68 | 53.3 | 14.0 | 34.5 | 5.1 |  |  |  |
| Almanza -Aguilera 2018 | Control (general recommendations) | 48 | 27 | 100 | 44.4 | 3.3 | 36.3 | 5.7 | NR | 0 | NR |
|  | Treatment (lifestyle weight loss intervention) | 67 | 30 | 100 | 45.7 | 3.5 | 35.4 | 4.1 | NR | 0 | NR |
| Anderson 2014 | Control (weight loss booklet only) | 166 | 166 | 26 | 63.6 | 6.7 | 30.4 | 3.9 | NR | 14.3 | NR |
|  | Intervention (BeWEL) | 163 | 163 | 26 | 63.5 | 7.0 | 31.0 | 4.5 | NR |  | NR |
| Annesi 2016 | Comparison treatment | 55 | 110 | 100 | 48.2 | 7.8 | 35.3 | 3.2 | NR | NR | NR |
|  | Experimental treatment | 55 |  |  |  |  |  |  | NR | NR | NR |
| Annesi 2017 | Control comparison group | 54 | 107 | 100 | 48.6 | 7.1 | 35.4 | 3.3 | NR | NR | NR |
|  | Experimental group | 53 |  |  |  |  |  |  | NR | NR | NR |
| Appel 2011 | Control (Self-directed) | 138 | 138 | 63.8 | 52.9 | 10.1 | 36.8 | 5.1 | NR | 23.8 | 76.8 |
|  | Remote Support Only (N/A) | 139 | N/A | N/A | N/A | N/A | N/A | N/A | N/A | N/A | N/A |
|  | In-Person Support | 138 | 138 | 63.8 | 53.3 | 10.5 | 36.8 | 5.2 | NR | 23.9 | 71.0 |
| Ard 2018 | Exercise Only | 54 | 54 | 68.5 | 69.9 | 4.5 | 33.9 | 0.4 | NR | NR | NR |
|  | Exercise + Diet Quality + Weight Maintenance | 55 | 55 | 60 | 70.5 | 4.8 | 33.8 | 0.4 | NR | NR | NR |
|  | Exercise + Diet Quality + Weight Loss | 55 | 55 | 58.2 | 70.3 | 4.8 | 33.3 | 0.4 | NR | NR | NR |
| Ash 2006 | Control Group - Booklet only | 63 | 54 | 77.8 | 47 | 14 | 35.8 | 6.2 | NR | NR | NR |
|  | Individualised Dietetic Treatment | 66 | 65 | 75.4 | 48 | 13 | 34.2 | 5.9 | NR | NR | NR |
|  | Fat Booters Incorporated | 62 | 57 | 66.7 | 49 | 13 | 33.7 | 4.6 | NR | NR | NR |
| Ashley 2001 | Control, Diet | 37 | 37 | 100 | 42.3 | 4.1 | 29.9 | 2.6 | NR | NR | NR |
|  | MR - Physician/Nurse led | 38 | 38 | 100 | 41 | 5.7 | 30.1 | 3.7 | NR | NR | NR |
|  | MR - Dietician lead | 38 | 38 | 100 | 41 | 4.3 | 30.1 | 2.9 | NR | NR | NR |
| Ashley 2007 | Control, TFG - Traditional Food Group | 35 | 35 | 100 | 39.8 | 6.1 | 29.5 | 3.1 | NR | NR | NR |
|  | MRG - Meal Replacement Group | 35 | 35 | 100 | 36.7 | 6.3 | 29.1 | 2.4 | NR | NR | NR |
| Aveyard 2016 | Advice only | 942 | 942 | 57 | 56.2 | 15.6 | 35.1 | 5.1 | NR | NR | NR |
|  | Advice plus weight loss programme | 940 | 940 | 57.3 | 55.8 | 16.5 | 34.8 | 4.6 | NR | NR | NR |
| Bacon 2002 | Health at Every Size - control | NR | 29 | 100 | 39.3 | 4.5 | 35.9 | 4.1 | NR | NR | NR |
|  | Diet Group - intervention | NR | 23 |  |  |  | 36.6 | 4.1 | NR | NR | NR |
| Bartels 2015 | Control, Fitness club membership | 106 | 106 | 55 | 43.5 | 11.6 | 37.5 | 8.8 | NR | NR | NR |
|  | IN SHAPE | 104 | 104 | 47 | 44.3 | 10.9 | 36.2 | 7.5 | NR | NR | NR |
| Beeken 2017 | Usual care | 267 | 267 | 64.8 | 60^a^ | 48.9-67.1^a^ | 34.8^a^ | 32.6-39.4^a^ | NR | NR | NR |
|  | 10TT | 270 | 270 | 66.7 | 59.1^a^ | 48.1-66.1^a^ | 35^a^ | 32.6-38.7^a^ | NR | NR | NR |
| Bennett 2012 | Control, Usual care | 185 | 185 | 65.9 | 54.7 | 11.0 | 36.99 | 5.2 | NR | NR | NR |
|  | Be Fit, Be Well | 180 | 180 | 71.1 | 54.6 | 10.8 | 37.03 | 5.0 | NR | NR | NR |
| Bennett 2013 | Control, usual care | 97 | 94 | 100 | 35.2 | 5.5 | 30.2 | 2.4 | NR | 5.3 | 36.2 |
|  | Weight gain prevention intervention | 97 | 91 | 100 | 35.6 | 5.5 | 30.1 | 2.7 | NR | 5.8 | 36.3 |
| Berry 2014 | Control | 162 | 162 | 92.6 | 36.8 | 8.1 | 39.1 | 8.3 | NR | NR | NR |
|  | Family based. Nutrition, exercise and coping skills intervention | 184 | 184 | 92.9 | 36.9 | 8.1 | 36.4 | 8.3 | NR | NR | NR |
| Bertz 2012 | Control | 17 | 17 | 100 | 32.2 | 4.6 | 30.2 | 3.4 | NR | NR | NR |
|  | Diet Only | 17 | 17 | 100 | 33.7 | 4.2 | 33.7 | 2.6 | NR | NR | NR |
|  | Exercise only | 18 | 18 | 100 | 33.2 | 3.7 | 33.2 | 3.1 | NR | NR | NR |
|  | Intervention | 16 | 16 | 100 | 33.9 | 4.5 | 33.9 | 2.2 | NR | NR | NR |
| Beutel 2006 | Behavioural therapy | 130 | 130 | 85 | 42.3 | 20-60^c^ | 43.9 | 35.2, 73.3^c^ | NR | NR | NR |
|  | Psychodynamic treatment | 137 | 137 | 86 | 40.3 | 20-64^c^ | 44.6 | 35.1, 73.5^c^ | NR | NR | NR |
| Bliddal 2011 | Control, low-energy diet | 45 | 45 | 88.9 | 64.1 | 10.5 | 35.2 | 4.5 | NR | NR | NR |
|  | Intensive low-energy diet | 44 | 44 | 88.6 | 61.1 | 11.1 | 36 | 5.5 | NR | NR | NR |
| Bo 2007 | Control standard care | 188 | 166 | 57.8 | 55.7 | 5.6 | 29.8 | 4.6 | NR | NR | 36.1 |
|  | Intervention lifestyle by trained professional | 187 | 169 | 58.6 | 55.7 | 5.7 | 29.7 | 4.1 | NR | NR | 36.1 |
| Brown 2014 | Control | 66 | 66 | 70 | 44.9 | 10.1 |  | 3.4 | NR | NR | NR |
|  | RENEW | 70 | 70 | 64 | 44.4 | 11.7 |  | 2.6 | NR | NR | NR |
| Burke 2015 | Standard behavioural weight loss treatment | 72 | 72 | 83.1 | 53 | 9.6 | 33.2 | 4.11 | NR | NR | NR |
|  | Self-efficacy enhancement plus standard behavioural weight loss treatment | 58 | 58 |  |  |  |  |  |  |  |  |
| Chee 2017 | Usual Care | 115 | 115 | 48.7 | 54 | 8 | 28.9 | 6.3 | NR | 100 | NR |
|  | tDNA Conventional Counseling | 57 | 57 | 87.4 | 55 | 8 | 29.4 | 7.3 | NR | 100 | NR |
|  | tDNA Motivational Interviewing | 58 | 58 | 67.2 | 55 | 8 | 30.7 | 8.2 | NR | 100 | NR |
| Cheyette 2007 | Control | 20 | 20 | 40 | 58 | 10.7 | 31.7 | 5.4 | NR | 100 | NR |
|  | Weight No More intervention group | 29 | 29 | 51.7 | 56.7 | 9.7 | 34.1 | 4.7 | NR | 100 | NR |
| Christensen 2012 | Reference group | 44 | 44 | 100 | 46 | 8.6 | 30.4 | 4.9 | NR | NR | NR |
|  | Intervention group | 54 | 54 | 100 | 45.7 | 6.36 | 30.5 | 5.4 | NR | NR | NR |
| Cleo 2018 | Wait list control (N/A) | 25 | N/A | N/A | N/A | N/A | N/A | N/A | N/A | N/A | N/A |
|  | TTT Top Ten Tips habit formation | 25 | 25 | 80 | 48.2 | 11.3 | 34.6 | 5.2 | NR | NR | NR |
|  | DSD Do Something Different online software | 25 | 25 | 76 | 51.3 | 10.0 | 35.2 | 7.4 | NR | NR | NR |
| Cole 2013 | Control - individualised counselling | 31 | 31 | 51 | 55 | 9.9 | 31.4 | 4.8 | NR | NR | 19 |
|  | Intervention- shared medical appointment | 34 | 34 | 41 | 61.2 | 8.4 | 30.3 | 5 | NR | NR | 25 |
| Conroy 2015 | Self-guided | 50 | 49 | 100 | 54 | 5.6 | 33.4 | 5.4 | NR | 23.5 | 56.1 |
|  | Interventionist led | 49 | 49 | 100 | 53.8 | 5.3 | 36.1 | 5.4 | NR |  |  |
| Cooper 2010 | Guided Self-Help Control | 51 | 51 | 100 | 41.86 | 8.67 | 35.41 | 2.71 | NR | 0 | NR |
|  | Behaviour Therapy | 50 | 50 | 100 | 41.38 | 9.90 | 34.79 | 3.06 | NR | 0 | NR |
|  | Cognitive Behaviour Therapy | 49 | 49 | 100 | 41.2 | 8.77 | 33.85 | 2.71 | NR | 0 | NR |
| Cousins 1992 | Control | 56 | 27 | 100 | 33.8 | 7.0 | 31.6 | 4.9 | NR | NR | NR |
|  | Individual | 56 | 32 | 100 | 33.6 | 6.4 | 31.7 | 5.0 | NR | NR | NR |
|  | Family | 56 | 27 | 100 | 32.8 | 6.1 | 30.3 | 4.5 | NR | NR | NR |
| Craighead 1989 | Control, minimal contact | 20 | 11 | NR | NR | NR | NR | NR | NR | NR | NR |
|  | Contracted Exercise | 20 | 14 | NR | NR | NR | NR | NR | NR | NR | NR |
|  | Supervised Exercise | 22 | 17 | NR | NR | NR | NR | NR | NR | NR | NR |
| Crowley 2017 | Group Medical Visit | 136 | 136 | 8.1 | 60.4 | 8.3 | 35 | 4.8 | 75.7 | 100 | 87.5 |
|  | Intensive Weight Management Group Medical Visit | 127 | 127 | 13.4 | 61 | 8.1 | 35.6 | 5.4 | 83.5 | 100 | 91.3 |
| Dale 2009 | Control | 23 | 23 | 74 | 45 | 30, 68^c^ | 36.5 | 4.3 | NR | NR | NR |
|  | Modest | 31 | 31 | 61 | 48 | 32, 62^c^ | 33.9 | 4.4 | NR | NR | NR |
|  | Intensive intervention | 25 | 25 | 68 | 46 | 31, 68^c^ | 32.5 | 5.2 | NR | NR | NR |
| Dalziel 2006 | Control | 303 | 303 | 7.9 | 53.5 | 10 | 25.8 | 3.4 | 100 | NR | NR |
|  | Experimental | 302 | 302 | 10.6 | 53.5 | 10 | 25.8 | 3.4 | 100 | NR | NR |
| Damschroder 2014 | Control, MOVE - usual care | 159 | 159 | 12.6 | 54.6 | 10.5 | 36.8 | 6.4 | NR | 37.7 | 65.4 |
|  | ASPIRE group, individual telephone counselling | 162 | 162 | 16 | 55.4 | 10.0 | 36.3 | 6.2 | NR | 32.7 | 67.9 |
|  | ASPIRE group, group counselling | 160 | 160 | 16.2 | 54.9 | 9.5 | 36.2 | 6.1 | NR | 40.0 | 65.6 |
| Daubenmier 2016^ | Active control intervention | 94 | 94 | 86 | 47.8 | 12.4 | 35.6 | 3.8 | NR | NR | 22.3 |
|  | Mindfulness Intervention | 100 | 100 | 79 | 47.2 | 13.0 | 35.4 | 3.5 | NR | NR | 16 |
| Delahanty 2015 | Dietitian Referral group | 29 | 29 | 41 | 61 | 11.4 | 33.8 | 5.0 | 27.6 | 100 | 82.6 |
|  | Group lifestyle intervention | 28 | 28 | 39 | 62 | 9.6 | 36.3 | 12.4 | 25.0 | 100 | 71.4 |
| deRoon 2017 | Control | 48 | 48 | 100 | 60 | 4.9 | 29.5 | 2.6 | NR | NR | NR |
|  | Diet | 97 | 97 | 100 | 61 | 4.6 | 29.5 | 2.6 | NR | NR | NR |
|  | Exercise | 98 | 98 | 100 | 59 | 4.9 | 29 | 2.9 | NR | NR | NR |
| deVos 2016 | Control | 204 | 204 | 100 | 55.7 | 3.2 | 32.5 | 4.5 | NR | NR | 74.5 |
|  | Tailor-made lifestyle intervention | 203 | 203 | 100 | 55.7 | 3.2 | 32.2 | 4.1 | NR | NR | 68.5 |
| Diabetes Prevention Program R G 2009 | Placebo | 1082 | 1082 | 69 | 50.3 | 10.4 | 32.2 | 6.7 | NR | All at high risk of T2DM | 30.0 |
|  | Metformin (N/A) | 1073 | 1073 | N/A | N/A | N/A | N/A | N/A | N/A |  |  |
|  | Lifestyle | 1079 | 1079 | 68 | 50.6 | 11.3 | 33.9 | 6.8 | NR |  |  |
| Djuric 2002 | Control | 13 | 48 | 100 | 51.7 | 8.4 | 34.9 | 1.2^b^ | NR | 6.3 | NR |
|  | Weight Watchers | 11 |  | 100 |  |  | 35 | 1.2^b^ | NR |  | NR |
|  | Individualized group | 13 |  | 100 |  |  | 35.5 | 1.1^b^ | NR |  | NR |
|  | Comprehensive group | 11 |  | 100 |  |  | 36.8 | 1^b^ | NR |  | NR |
| Duncan 2016 | Control | 162 | 159 | 42.8 | 54.8 | 8.48 | 31.8 | 6.91 | NR | NR | NR |
|  | Intervention | 158 | 154 | 45.1 | 53.1 | 9.83 | 33.8 | 7.14 | NR | NR | NR |
| Eaton 2016 | Control, Standard Intervention | 106 | 106 | 83 | 48.6 | 11.2 | 37.8 | 6.7 | NR | NR | NR |
|  | Enhanced Intervention | 105 | 105 | 75.2 | 48.5 | 11.9 | 37.7 | 6.5 | NR | NR | NR |
| Fahey 2018 | Self-paced condition | 124 | 124 | 50.8 | 33.8 | 6.8 | NR | NR | NR | NR | NR |
|  | Counselor- initiated condition | 124 | 124 | 50.8 | 35.3 | 8.2 | NR | NR | NR | NR | NR |
| Fernandez-Ruiz 2018 | Control | 37 | 37 | 51.4 | 62.8 | 8.9 | 34.3 | 4.5 | NR | 62.2 | 86.5 |
|  | Intervention (healthy eating, exercise & CBT) | 37 | 37 | 48.6 | 59.4 | 9.1 | 32.4 | 3.8 | NR | 43.2 | 78.4 |
| Finkelstein 2017 | Control | 54 | 54 | 55.6 | 45 | 10.2 | 29.5 | 3.5 | NR | NR | NR |
|  | Financial Reward | 107 | 107 | 55.7 | 43.4 | 9.8 | 29.8 | 3.1 | NR | NR | NR |
| Fisher 2011 | Diet only | 29 | NR | 100 | NR | NR | 28 | 3 | NR | NR | NR |
|  | Diet + aerobic training | 43 | NR |  | NR | NR |  |  | NR | NR | NR |
|  | Diet + resistance training | 54 | NR |  | NR | NR |  |  | NR | NR | NR |
| Foley 2016 | Usual care (Control) | 175 | 175 | 68 | 50.5 | 8.7 | 35.9 | 3.7 | NR | 3.4 | 29.1 |
|  | Weight loss intervention | 176 | 176 | 68 | 50.9 | 9.1 | 35.9 | 4.1 | NR | 3.4 | 29.5 |
| Foreyt 1993 | Control (N/A) | 38 | 38 | 48 | NR | NR | NR | NR | N/A | N/A | N/A |
|  | Exercise only | 43 | 43 |  |  |  |  |  | NR | NR | NR |
|  | Diet only | 42 | 42 |  |  |  |  |  | NR | NR | NR |
|  | Exercise plus diet | 42 | 42 |  |  |  |  |  | NR | NR | NR |
| Foster-Schubert 2012 | Control- usual care | 87 | 87 | 100 | 57.4 | 4.4 | 30.7 | 3.9 | NR | NR | NR |
|  | Calorie reduced diet | 118 | 118 | 100 | 58.1 | 5.9 | 31 | 3.9 | NR | NR | NR |
|  | Aerobic exercise (N/A) | 117 | 117 | N/A | N/A | N/A | N/A | N/A | N/A | N/A | N/A |
|  | Intervention - diet and exercise | 117 | 117 | 100 | 58.0 | 4.5 | 31 | 4.3 | NR | NR | NR |
| Freitas 2017 | Weight loss program + Sham | 27 | 25 | 100 | 48.5 | 9.6 | 37.2 | 2.1 | NR | NR | 48.0 |
|  | Weight loss program + Exercise | 28 | 26 | 96 | 45.9 | 7.7 | 38.1 | 2.8 | NR | NR | 38.5 |
| Fuller 2012 | Western diet group | 35 | 35 | 54.3 | 47.1 | 11.1 | 31.0 | 3.8 | NR | NR | NR |
|  | Korean diet group | 35 | 35 | 71.4 | 43.7 | 11 | 31.2 | 4.0 | NR | NR | NR |
| Gold 2007 | Commercial programme eDiets | 62 | 62 | 86 | 48.9 | 9.9 | 32.5 | 4.2 | NR | NR | NR |
|  | Structured VTrim | 62 | 62 | 77 | 46.5 | 10.7 | 32.3 | 3.9 | NR | NR | NR |
| Goodwin 2014 | Mailed-based intervention | 167 | 167 | 100 | 60.4 | 7.8 | 31.1 | 5.3 | NR | 0.0 | NR |
|  | Individual lifestyle intervention | 171 | 171 | 100 | 61.6 | 6.7 | 31.4 | 5.0 | NR | 0.0 | NR |
| Hakala 1993 | Individual community-based counselling | 28 | 28 | 7.4 | 38.1 | NR | 42.8 | 4.5 | NR | NR | NR |
|  | Group in-patient rehabilitation | 30 | 30 | 8.1 | 40.3 | NR | 43.3 | 4.8 | NR | NR | NR |
| Hanson 1976 | No treatment control condition (N/A) | 10 | 66 | 87.9 | 40 | NR | NR | NR | NR | NR | NR |
|  | Attention-placebo control condition | 11 |  |  |  |  |  |  |  |  |  |
|  | Conventional self-management condition | 7 |  |  |  |  |  |  |  |  |  |
|  | Programmed text with low therapist-group contact | 12 |  |  |  |  |  |  |  |  |  |
|  | Programmed text with high therapist-group contact | 13 |  |  |  |  |  |  |  |  |  |
| Harrigan 2016 | Usual Care Group | 33 | 33 | 100 | 58 | 7.5 | 34 | 7.5 | NR | NR | NR |
|  | Telephone Weight Loss Counseling | 34 | 34 | 100 | 60 | 7.7 | 31.8 | 5.4 | NR | NR | NR |
|  | In-Person Weight Loss Counseling | 33 | 33 | 100 | 58.9 | 7.3 | 33.5 | 6.7 | NR | NR | NR |
| Hunt 2014 | Control, Wait-list | 373 | 373 | 0 | 47.2 | 7.89 | 35.1 | 4.8 | NR | NR | NR |
|  | FFIT | 374 | 374 | 0 | 47 | 8.07 | 35.5 | 5.1 | NR | NR | NR |
| Huseinovic 2016 | Control Group | 56 | 56 | 100 | 32.6 | 4.7 | 31^a^ | 26.3–48.7^c^ | NR | NR | NR |
|  | Diet behaviour modification Group | 54 | 54 | 100 | 31.8 | 4.5 |  |  | NR | NR | NR |
| Irwin 2003 | Control Group | 86 | 86 | 100 | 60.6 | 59.1, 62.1^d^ | 30.6 | 29.8, 31.4^d^ | NR | NR | NR |
|  | Exercise group | 87 | 87 |  | 61 | 59.6, 62.5^d^ | 30.5 | 29.6, 31.4^d^ | NR | NR | NR |
| Jakicic 2011 | Self Help Group | 89 | 89 | 92 | 44.7 | 7.9 | 27.1 | 1.7 | NR | NR | NR |
|  | Moderate Physical Activity | 82 | 82 | 90 | 43.5 | 8.8 | 27.1 | 1.7 | NR | NR | NR |
|  | High Physical Activity | 98 | 98 | 92 | 45 | 8.4 | 27 | 1.6 | NR | NR | NR |
| Jakicic 2015 | Standard behavior weight loss interventions group | 71 | 69 | 79.7 | 43.0 | 9.3 | 32.7 | 3.7 | NR | NR | NR |
|  | ADOPT group | 71 | 64 | 76.6 | 43.3 | 8.6 | 33.3 | 2.9 | NR | NR | NR |
|  | MAINTAIN Group (N/A) | 71 | N/A | N/A | N/A | N/A | N/A | N/A | N/A | N/A | N/A |
| Jebb 2011 | Standard care | 395 | 395 | 86 | 48.2 | 12.2 | 31.3 | 2.6 | NR | 6.8 | 25.1 |
|  | Commercial programme | 377 | 377 | 88 | 46.5 | 13.5 | 31.5 | 2.6 | NR | 6.4 | 25.5 |
| Jebb 2017 | Usual care | 140 | 140 | 60.0 | 47.4 | 12.8 | 36.8 | 5.1 | NR | 14.3 | 21.4 |
|  | Low energy total diet replacement programme | 138 | 138 | 60.5 | 48.2 | 11.5 | 37.6 | 5.7 | NR | 15.2 | 23.9 |
| Jeffery 2003 | Standard behaviour therapy | 93 | 202 | 58 | 42.2 | 6.4 | 31.7 | 2.6 | NR | NR | NR |
|  | High physical activity | 109 |  |  |  |  |  |  | NR | NR | NR |
| Jolly 2011 | Minimal intervention comparator | 100 | 100 | 75 | 49.67 | 13.83 | 33.9 | 4.4 | NR | NR | NR |
|  | Choice (N/A) | 100 | N/A | N/A | N/A | N/A | N/A | N/A | N/A | N/A | N/A |
|  | Pharmacy | 70 | 70 | 73 | 48.9 | 15.8 | 33.4 | 3.5 | NR | NR | NR |
|  | General practice | 70 | 70 | 67 | 50.5 | 13.8 | 33.1 | 3.5 | NR | NR | NR |
|  | Weight Watchers | 100 | 100 | 72 | 50.7 | 14.6 | 34.0 | 3.9 | NR | NR | NR |
|  | NHS Size Down | 100 | 100 | 64 | 48.8 | 15.6 | 33.8 | 3.9 | NR | NR | NR |
|  | Rosemary Conley | 100 | 100 | 69 | 49.8 | 49.8 | 33.4 | 3.5 | NR | NR | NR |
|  | Slimming world | 100 | 100 | 65 | 48.8 | 14.9 | 33.8 | 3.8 | NR | NR | NR |
| Jones 1999 | Control Group | NS | 51 | 49.0 | 59 | 7 | 34 | 6 | NR | NR | 100 |
|  | Weight Loss Group | NS | 51 | 54.9 | 57 | 6 | 34 | 6 | NR | NR | 100 |
| Katula 2013 | Enhanced Usual Care Comparison Condition | 150 | 150 | 57.3 | 58.5 | 9.0 | 32.6 | 4.1 | NR | 0.0 | 52.0 |
|  | Lifestyle Weight-Loss Intervention | 151 | 151 | 57.6 | 57.3 | 10.1 | 32.8 | 3.9 | NR | 0.0 | 51.7 |
| Keogh 2014 | Intermittent dieting | 39 | 19 | 100 | 59.5 | 8.7 | 33.1 | 3.8 | NR | 1 | NR |
|  | Continuous dieting | 36 | 17 | 100 | 60.8 | 12.5 | 33.0 | 7.5 | NR |  | NR |
| Keranen 2009 | Short-term counselling | 47 | 47 | 70 | 49 | 9 | 35 | 5 | NR | NR | NR |
|  | Intensive counselling | 35 | 35 | 74 | 50 | 8 | 35 | 5 | NR | NR | NR |
| King 1989 | Control (N/A) | 52 | N/A | N/A | N/A | N/A | N/A | N/A | N/A | N/A | N/A |
|  | Exercise only | 52 | 36 | 0 | 44.8 | 7.4 | NR | NR | NR | NR | NR |
|  | Diet only | 51 | 36 | 0 | 45 | 7.6 | NR | NR | NR | NR | NR |
| Knauper 2018 | Standard DPP | 101 | 85 | 76.5 | 49.4 | 11.8 | NR | NR | NR | NR | NR |
|  | Enhanced DPP | 107 | 87 | 83.9 | 50.9 | 12.1 | NR | NR | NR | NR | NR |
| Kumanyika 2012 | Basic programme | 137 | 137 | 82.5 | 46.8 | 11.6 | 37.3 | 6.4 | 10.9 | 19.7^#^ | 41.6 |
|  | Basic plus programme | 124 | 124 | 86.3 | 47.6 | 11.9 | 37.2 | 6.5 | 6.5 | 16.9^#^ | 46.0 |
| Leahey 2014 | SURI alone | 46 | 46 | 82.6 | 46.5 | 1.7 ^b^ | 35.1 | 1.3 ^b^ | NR | NR | NR |
|  | SURI plus Internet behavioral weight loss program | 90 | 90 | 82.2 | 46.2 | 1.2^b^ | 34.7 | 0.7 ^b^ | NR | NR | NR |
|  | SURI plus Internet behavioral weight loss program plus optional group sessions | 94 | 94 | 86.2 | 47.7 | 1.1 ^b^ | 33.4 | 0.7 ^b^ | NR | NR | NR |
| Leahey 2015 | SURI1 Internet behavioral weight loss | 91 | 91 | 83.5 | 45.1 | 11.0 | 32.9 | 5.5 | NR | NR | NR |
|  | SURI1 Internet behavioral weight loss1incentives | 89 | 89 | 79.8 | 46.3 | 9.4 | 33.5 | 6.5 | NR | NR | NR |
|  | SURI1 Internet behavioral weight loss 1 group option | 88 | 88 | 84.1 | 47.4 | 11.4 | 34.3 | 6.8 | NR | NR | NR |
| Lejeune 2003 | Diet | 20 | 37 | 0 | 39 | 7.1 | 32.3 | 2.3 | NR | NR | NR |
|  | Diet plus exercise | 20 |  |  |  |  |  |  | NR | NR | NR |
| Ley 2004 | Control diet | 70 | 70 | 20 | 52 | 0.8 ^b^ | 29.1 | 0.6 ^b^ | NR | NR | NR |
|  | Reduced-fat | 66 | 66 | 31.8 | 52.5 | 0.8 ^b^ | 29.3 | 0.6 ^b^ | NR | NR | NR |
| Li 2016 | Usual care group | 60 | 60 | 35 | 59 | 3.9 | 25.2 | 0.9 | NR | 100 | NR |
|  | Diet group | 79 | 79 | 46.8 | 59.7 | 6.5 | 27.2 | 2.8 | NR | 100 | NR |
|  | 50g-oats group | 80 | 80 | 48.8 | 59.7 | 6.1 | 26.9 | 2.7 | NR | 100 | NR |
|  | 100g-oats group | 79 | 79 | 58.2 | 59.4 | 6.8 | 27.4 | 2.4 | NR | 100 | NR |
| Li 2005 | Individualized diet plan | 52 | 36 | 33.3 | 56.6 | 10.4 ^b^ | 33.7 | 3.6 ^b^ | NR | 100 | NR |
|  | Soy-based meal replacement | 52 | 46 | 41.3 | 54.4 | 9.3 ^b^ | 32.8 | 3.7 ^b^ | NR | 100 | NR |
| Lindstrom 2003 | Control | 257 | 257 | 68.5 | 55 | 7 | 31.4 | 4.5 | NR | NR | 31.1 |
|  | Intervention | 265 | 265 | 65.7 | 55 | 7 | 31.1 | 4.5 | NR | NR | 29.1 |
| Liss 2016 | Standard care arm | 167 | 167 | 48.5 | 56.6 | 12.2 | 34.9 | 7.3 | NR | 100 | 76.6 |
|  | Standard care plus group-based lifestyle intervention | 164 | 164 | 51.8 | 57.1 | 10.6 | 36.2 | 7.8 | NR | 100 | 80.5 |
| Little 2016 | Control, Nurse follow-up | 279 | 279 | 66 | 52.7 | 13.3 | 37.1 | 6.0 | NR | NR | NR |
|  | Web-based support with minimal support (Remote) | 270 | 270 | 60 | 54.7 | 13.0 | 36.3 | 5.7 | NR | NR | NR |
|  | Web-based + nurse support (face to face) | 269 | 269 | 65 | 53.7 | 13.2 | 36.7 | 5.4 | NR | NR | NR |
| Lowe 2018 | Behavior therapy | 90 | 90 | 83 | 47.7 | 12.57 | NR | NR | NR | NR | NR |
|  | Behavior therapy plus meal replacements | 91 | 91 | 85 | 50.38 | 9.39 | NR | NR | NR | NR | NR |
|  | Home food environment | 81 | 81 | 73 | 51.5 | 9.42 | NR | NR | NR | NR | NR |
| Ma 2015 | Control, Enhanced usual care | 165 | 165 | 70.9 | 47.7 | 12.1 | 37.6 | 5.7 | NR | NR | NR |
|  | Diet and counselling | 165 | 165 | 70.3 | 47.5 | 12.6 | 37.4 | 6.0 | NR | NR | NR |
| Manning 1994 | Clinic visit | 37 | 37 | 56.8 | 57.3 | 54.1, 60.5 | 31.2 | 30.1, 32.3 | NR | NR | NR |
|  | Behavioural | 38 | 38 | 47.4 | 58.8 | 55.9, 61.7 | 32.2 | 30.5, 33.9 | NR | NR | NR |
|  | Home visits | 35 | 35 | 42.7 | 55.2 | 51.6, 58.8 | 32 | 30.9, 33.1 | NR | NR | NR |
|  | Dexfenfluramine (N/A) | N/A | N/A | N/A | N/A | N/A | N/A | N/A | N/A | N/A | N/A |
|  | Routine usual care (N/A) | N/A | N/A | N/A | N/A | N/A | N/A | N/A | N/A | N/A | N/A |
| Manzoni 2016 | Control, Standard behavioral inpatient program | 52 | 158 | 100 | 35.6 | 8.04 | 42.2 | 6.01 | NR | NR | NR |
|  | Cognitive–behavioral therapy | 54 |  |  |  |  |  |  | NR | NR | NR |
|  | CBT + Virtual reality | 57 |  |  |  |  |  |  | NR | NR | NR |
| Marniemi 1990 | Control group | 42 | 42 | 76.2 | 38.0 | NR | 33.6 | NR | NR | NR | NR |
|  | Lactovegetarian weight reduction group | 31 | 31 | 74.2 |  |  | 34.4 | NR | NR | NR | NR |
|  | Mixed diet weight reduction | 37 | 37 | 73.0 |  |  | NR | NR | NR | NR | NR |
| Martin 2008 | Control, Standard Care | 69 | 69 | 100 | 42.6 | 11.4 | 39.8 | 7.8 | NR | NR | NR |
|  | Tailored physician/lifestyle counselling | 68 | 68 | 100 | 40.8 | 12.7 | 38.3 | 7.5 | NR | NR | NR |
| Mefferd 2007 | Control | 29 | 29 | 100 | 56.4 | 7.5 | 31.3 | 4.8 | NR | NR | NR |
|  | Intervention | 56 | 56 | 100 | 55.9 | 8.7 | 31 | 3.7 | NR | NR | NR |
| Mengham 1999 | Control | NR | 36 | 44.4 | 63.5 | 10.9 | 31.7 | 4.9 | NR | 89.2 | NR |
|  | Intervention | NR | 38 | 44.7 | 57.8 | 13.5 | 31.4 | 4.4 | NR |  | NR |
| Messier 2013 | Exercise only | 150 | 150 | 72 | 66 | 6 | 33.5 | 3.7 | 8.0 | 12.0^#^ | 59.3 |
|  | Diet-induced weight loss only | 152 | 152 | 71 | 66 | 6 | 33.7 | 3.8 | 12.5 | 11.8^#^ | 61.2 |
|  | Diet-induced weight loss plus exercise | 152 | 152 | 72 | 65 | 6 | 33.6 | 3.7 | 7.2 | 15.1^#^ | 59.9 |
| Miller 2002 | Control Group (Monitoring) | 23 | 23 | 68 | 54 | 8 | 34.2 | 6.2 | NR | NR | 23 |
|  | Lifestyle Intervention | 22 | 22 | 57 | 53 | 11 | 32.8 | 5.4 | NR | NR | 22 |
| Mitsui 2008 | Control | 22 | 21 | 54.5 | 67.4 | 10.6 | 25.6 | 2.5 | NR | NR | 18.6 |
|  | Intervention | 24 | 22 | 54.2 | 64 | 8.9 | 24.8 | 2.2 | NR | NR |  |
| Molenaar 2010 | Nutritional counselling group (diet D, group) | 67 | 67 | 42 | 43 | 9 | 31.3 | 2 | NR | NR | NR |
|  | Nutritional plus exercise counselling group (diet + exercise (D + E) group) | 67 | 67 | 42 | 43 | 10 | 30.8 | 1.9 | NR | NR | NR |
| Moreno 2014 | Low-calorie diet | 39 | 26 | 96.1 | 46.3 | 9.3 | 35.1 | 5.3 | NR | 3.8 | 19.2 |
|  | Very low-calorie-ketogenic diet | 40 | 27 | 81.4 | 44.4 | 8.6 | 35.1 | 4.5 | NR | 7.4 | 14.8 |
| Morgan 2010 | Control (Information and self-help) | 31 | 31 | 0 | 34 | 11.6 | 30.5 | 3.0 | NR | NR | NR |
|  | SHED-IT (Internet) group | 34 | 34 | 0 | 37.5 | 10.4 | 30.6 | 2.7 | NR | NR | NR |
| Muggia 2014 | Standard care group | 83 | 83 | 71.1 | 43.5 | 10.0 | 32.5 | 3.7 | NR | NR | NR |
|  | Brief CBT group | 80 | 80 | 76.3 | 46.2 | 11.7 | 31.9 | 3. | NR | NR | NR |
| Ng 2015 | Control group | 43 | 43 | 30.7 | 52 | 9.3 | 30.5 | 4.2 | NR | 25.6^#^ | 20.9 |
|  | Lifestyle modification program | 61 | 61 | 21.3 | 51.4 | 9.1 | 30.2 | 3.9 | NR | 23.0^#^ | 26.2 |
| Nicklas 2009 | Calorie restriction (CR) Only | 34 | 34 | 100 | 58.4 | 6.0 | 33.9 | 4.0 | 17.6 | NR | 32.4 |
|  | CR + Moderate-Intensity | 40 | 40 | 100 | 57.7 | 5.5 | 33.7 | 3.5 | 17.5 | NR | 25.0 |
|  | CR + Vigorous-Intensity | 38 | 38 | 100 | 59 | 5.0 | 32.9 | 3.7 | 18.4 | NR | 21.1 |
| Nilsen 2011 | Control, Individual Physician Group | 104 | 104 | 47 | 45.9 | 11 | 35.9 | 6 | NR | NR | NR |
|  | Individual Plus Interdisciplinary Group | 109 | 109 | 53 | 47 | 11 | 37.6 | 6 | NR | NR | NR |
| Oldroyd 2006 | Control group | 39 | 32 | 31.25 | 57.5 | 41 – 73^c^ | 29.9 | 4.9 | NR | NR | NR |
|  | Intervention group | 39 | 37 | 54.05 | 58.2 | 41 – 75^c^ | 30.4 | 5.6 | NR | NR | NR |
| Parikh 2010 | Control | 49 | 49 | 84 | 50 | 18 | 31 | 5.0 | NR | 0.0 | NR |
|  | Intervention | 50 | 50 | 86 | 46 | 15 | 32 | 4.0 | NR | 0.0 | NR |
| Pedersen 2013 | Aerobic interval training | 35 | 26 | 15 | 62.3 | 5.7 | 31.6^a^ | 29.6, 34.8^a^ | 26 | 0 | 96.4 |
|  | Low energy diet | 35 | 29 | 28 | 63.8 | 6.8 | 31.1^a^ | 29.9, 32.7^a^ | 29 | 0 |  |
| Perri 1986 | Behavior therapy | NR | 90 | 84.4 | 43.3 | NR | NR | NR | NR | NR | NR |
|  | Behavior therapy plus maintenance | NR |  |  |  | NR | NR | NR | NR | NR | NR |
|  | Behavior therapy plus aerobic exercise | NR |  |  |  | NR | NR | NR | NR | NR | NR |
|  | Behavior therapy behavior therapy plus aerobic exercise plus maintenance | NR |  |  |  | NR | NR | NR | NR | NR | NR |
| Perri 1997 | WL + Home-based exercise | 24 | 23 | 100 | 48.9 | 5.0 | 33.1 | 2.9 | NR | NR | NR |
|  | WL + Group-based exercise | 25 | 25 | 100 | 48.7 | 6.2 | 34.0 | 4.5 | NR | NR | NR |
| Perri 2001 | Control, Standard Behavioural Therapy (BT) | NR | 18 | 100 | 45.2 | 10.1 | 36.4 | 4.7 | NR | NR | NR |
|  | BT + Relapse prevention training | NR | 28 | 100 | 49.2 | 7.2 | 35 | 4.0 | NR | NR | NR |
|  | BT + problem-solving therapy | NR | 34 | 100 | 45.4 | 9.3 | 36.1 | 4.9 | NR | NR | NR |
| Pettman 2009 | Control | 50 | 50 | 72 | NR | NR | 36.5 | 6.5 | NR | NR | NR |
|  | Intervention B - Passive follow-up | 54 | NR | NR | NR | NR | 37.3 | 6.2 | NR | NR | NR |
|  | Intervention A - Active follow-up | 49 | NR | NR | NR | NR | 36.1 | 6.6 | NR | NR | NR |
| Poelman 2015 | Control Condition | 139 | 139 | 84.2 | 45.4 | 9.2 | 32.9 | 5.0 | NR | NR | NR |
|  | Intervention condition | 139 | 139 | 84.9 | 45.9 | 9.2 | 32 | 4.6 | NR | NR | NR |
| Promrat 2010 | Control | 10 | 10 | 20 | 47.6 | 12.0 | 33.7 | 4.7 | NR | 40.0 | NR |
|  | Lifestyle Intervention | 21 | 21 | 33.3 | 48.9 | 10.9 | 33.9 | 5.3 | NR | 52.4 | NR |
| Provencher 2009 | Control group | 48 | 47 | 100 | 41.8 | 6.0 | 30.5 | 3.0 | NR | 0.0 | NR |
|  | Social support | 48 | 46 | 100 | 42.3 | 5.5 | 30.6 | 3.1 | NR | 0.0 | NR |
|  | Health-At-Every-Size | 48 | 48 | 100 | 42.8 | 5.5 | 30.1 | 3.0 | NR | 0.0 | NR |
| Rock 2015 | Control | 349 | 348 | 100 | 56.5 | 9.5 | 31.4 | 4.6 | NR | NR | 28.7 |
|  | Intervention | 348 | 345 | 100 | 56.1 | 9.4 | 31.6 | 4.7 | NR | NR | 30.7 |
| Rolls 2005 | Comparison-control | 50 | 50 | NR | 45.2 | 1.2^b^ | 31.3 | 0.4^b^ | NR | NR | NR |
|  | Two snacks | 50 | 50 | NR | 44.5 | 1.2 ^b^ | 31.4 | 0.4^b^ | NR | NR | NR |
|  | One soup | 50 | 50 | NR | 45.1 | 1.2 ^b^ | 30.9 | 0.5^b^ | NR | NR | NR |
|  | Two soups | 50 | 50 | NR | 43.8 | 1.2 ^b^ | 30.8 | 0.5^b^ | NR | NR | NR |
| Rolls 2017 | Standard advice | 62 | 62 | 100 | 49.5 | 12.0 | 34.1 | 4.3 | NR | NR | NR |
|  | Pre-portioned foods group | 62 | 62 | 100 | 50.1 | 10.1 | 34.2 | 4.1 | NR | NR | NR |
|  | Portion selection group | 62 | 62 | 100 | 50.4 | 9.6 | 33.6 | 4.2 | NR | NR | NR |
| Rosas 2015 | Usual care | 41 | 41 | 78 | 47.6 | 10.5 | 34.9 | 4.4 | NR | 43.9^#^ | NR |
|  | Case-management intervention | 84 | 84 | 76.2 | 47.9 | 11.9 | 36 | 5.7 | NR | 44.0^#^ | NR |
|  | Case-management + Community health worker intervention | 82 | 82 | 76.8 | 46 | 10.7 | 35.5 | 5.1 | NR | 41.5^#^ | NR |
| Ross 2012 | Control condition | 241 | 241 | 70.1 | 52.4 | 11.8 | 32 | 4.2 | NR | NR | 33.2 |
|  | Behavioral intervention group | 249 | 249 | 70.28 | 51.3 | 11 | 32.6 | 4.1 | NR | NR | 25.7 |
| Samaras 1997 | Control | 13 | 13 | 53.8 | 60.5 | 2.1^b^ | 35.7 | 1.6^b^ | NR | NR | NR |
|  | Intervention | 13 | 13 | 69.2 | 60.5 | 7.8^b^ | 32.3 | 1.1^b^ | NR | NR | NR |
| Santanasto 2011 | Physical Activity plus Successful Ageing | 15 | 15 | 86.7 | 69.9 | 5.9 | 32 | 3.1 | NR | NR | NR |
|  | Physical Activity plus Weight Loss | 21 | 21 | 81 | 70.6 | 5.9 | 33.6 | 3.3 | NR | NR | NR |
| Sattin 2016 | Health Education intervention | 287 | 287 | 82.6 | 46.4 | 10.9 | 35.6 | 7.6 | NR | 0 | NR |
|  | Fit body and soul intervention | 317 | 317 | 84.2 | 46.6 | 10.9 | 35.8 | 7 | NR | 0 | NR |
| Schubel 2016 | Control group | 52 | 52 | 52 | 50.7 | 7.1 | 31.1 | 3.6 | NR | 0 | NR |
|  | Continuous Calorie Restriction | 49 | 49 | 49 | 50.5 | 8.0 | 31.2 | 4.0 | NR | 0 | NR |
|  | Intermittent Calorie Restriction | 49 | 49 | 49 | 49.4 | 9.0 | 32 | 3.8 | NR | 0 | NR |
| Seligman 2011 | Standard-of-care strategy | 25 | 25 | 32 | 42 | 8^b^ | 34.7 | 0.6^b^ | NR | NR | 52.0 |
|  | Healthy diet and step counter | 25 | 25 | 36 | 44 | 7^b^ | 34.4 | 0.6^b^ | NR | NR | 64.0 |
|  | Healthy diet and fitness | 26 | 25 | 36 | 43 | 8^b^ | 35.2 | 0.5^b^ | NR | NR | 64.0 |
| Shikany 2013 | Food-based diet | 60 | 60 | 90 | 39.7 | 9.1 | 41.3 | 3.8 | NR | NR | NR |
|  | Meal replacement | 60 | 60 | 86.7 | 40.2 | 9.2 | 40.6 | 3.8 | NR | NR | NR |
| Silva 2010 | Comparison group | 116 | 116 | 100 | 37.1 | 6.99 | 31.3 | 4.00 | NR | NR | NR |
|  | Intervention | 123 | 123 | 100 | 38.1 | 7.04 | 31.7 | 4.24 | NR | NR | NR |
| Solbrig 2019 | Motivational interviewing | 58 | 55 | 72.72 | 42 | 19–70^c^ | 32.54 | 24.5–53.3^c^ | NR | NR | NR |
|  | Functional imagery training | 63 | 59 | 72.88 | 45 | 20–72^c^ | 33.21 | 26.0–48.0c | NR | NR | NR |
| Somers 2012 | Standard Care | 51 | 51 | 78 | 57.9 | 10.1 | 34.1 | 4.6 | NR | NR | NR |
|  | Lifestyle behavioral weight management intervention only | 59 | 59 | 80 | 58.3 | 11.0 | 33.5 | 4.4 | NR | NR | NR |
|  | Lifestyle behavioral weight management intervention + Pain Coping Skills Training | 62 | 62 | 92 | 57.5 | 9.43 | 34.1 | 4.3 | NR | NR | NR |
|  | Pain Coping Skills Training only (N/A) | 60 | 60 | N/A | N/A | N/A | N/A | N/A | N/A | N/A | N/A |
| Spring 2013 | MOVE standard care | 35 | 35 | 14.2 | 57.7 | 10.2 | 35.8 | 3.8 | NR | NR | NR |
|  | MOVE + personal digital assistant | 34 | 34 | 14.7 | 57.7 | 13.5 | 36.9 | 5.4 | NR | NR | NR |
| Spring 2017 | Control self-guided program | 32 | 32 | 84.4 | 40.1 | 11.1 | 34.3 | 3.2 | NR | NR | NR |
|  | Standard weight loss program | 32 | 32 | 81.3 | 37.3 | 13.3 | 34.8 | 3.0 | NR | NR | NR |
|  | Technology-supported | 32 | 32 | 87.5 | 40.4 | 10.7 | 34.8 | 2.8 | NR | NR | NR |
| Stahre 2005 | Control | 43 | 43 | 100 | 45.2 | 11.3 | 39.2 | NR | NR | NR | NR |
|  | Cognitive treatment | 62 | 62 | 100 | 45.4 | 9.8 | 40.4 | NR | NR | NR | NR |
| Stenius-Aarniala 2000 | Control | 19 | 19 | 68.7 | 48.3 | 23-60^c^ | 36.7 | 32.8-41.8^c^ | NR | NR | NR |
|  | Treatment with VLCD | 19 | 19 | 68.4 | 49.7 | 34-60^c^ | 35.8 | 31.3-39.4^c^ | NR | NR | NR |
| Stevens 1993 | Control | 256 | 256 | 37 | 42.4 | 6.2 | 29.5 | 2.8 | NR | NR | 0.0 |
|  | Intervention | 308 | 308 | 17 | 43.1 | 6.0 | 29.5 | 2.9 | NR | NR | 0.0 |
| Stevens 2001 | Control | 596 | 596 | 31.7 | 43.2 | 6.1 | 30.9 | 3.2 | NR | NR | NR |
|  | Intervention | 595 | 595 | 37 | 43.4 | 6.1 | 31 | 3.3 | NR | NR | NR |
|  | Sodium only intervention (N/A) | 594 | N/A | N/A | N/A | N/A | N/A | N/A | N/A | N/A | N/A |
|  | Combined intervention (N/A) | 597 | N/A | N/A | N/A | N/A | N/A | N/A | N/A | N/A | N/A |
| Strobl 2013 | Control, Usual care | 239 | 239 | 44 | 48.03 | 9.8 | 36.3 | 3.4 | NR | NR | NR |
|  | Telephone aftercare | 228 | 228 | 46 | 48.54 | 9.8 | 35.4 | 3.6 | NR | NR | NR |
| Sundfor 2018 | Continuous energy restriction | 58 | 58 | 51.7 | 47.5 | 11.6 | 35.3 | 3.5 | NR | 6.9^#^ | 92.0 |
|  | Intermittent energy restriction | 54 | 54 | 48.1 | 49.9 | 10.1 | 35.1 | 3.9 | NR | 1.9^#^ | 37.0 |
| Tapsell 2017 | Usual care (Control) | 126 | 126 | 73 | 43.8 | 7.46 | 32.49 | 4.12 | NR | NR | 11.1 |
|  | Intervention Group | 125 | 124 | 73 | 43.79 | 7.97 | 32.59 | 4.25 | NR | NR | 16.1 |
|  | Intervention plus food supplement group (N/A) | 126 | N/A | N/A | N/A | N/A | N/A | N/A | N/A | N/A | N/A |
| TarragaMarcos 2017 | G3 | 55 | 55 | 32.4 | 49.8 | 6.6 | 30.7 | 3.4 | NR | NR | NR |
|  | G2 | 61 | 61 | 34.3 | 49.7 | 6.4 | 30.8 | 3.6 | NR | NR | NR |
|  | G1 | 60 | 60 | 33.3 | 50.1 | 7.2 | 30.3 | 3.2 | NR | NR | NR |
| Teeriniemi 2018 | Control | 89 | 89 | 51.7 | 46.5 | 10.2 | 30.5 | 2.3 | NR | 2.2^#^ | 25.8 |
|  | SHG Counselling | 87 | 87 | 48.3 | 44.4 | 10.2 | 30.7 | 2.2 | NR | 2.3^#^ | 26.4 |
|  | CBT Counselling | 85 | 85 | 49.4 | 46.4 | 9.7 | 30.5 | 1.9 | NR | 3.5^#^ | 20.0 |
|  | Control plus HBCSS | 91 | 91 | 47.3 | 47 | 9.4 | 30.3 | 2.0 | NR | 3.3^#^ | 22.0 |
|  | SHG Counselling plus HBCSS | 92 | 92 | 48.9 | 46.4 | 10.5 | 30.4 | 2.1 | NR | 1.1^#^ | 25.0 |
|  | CBT Counselling plus HBCSS | 88 | 88 | 50 | 44.8 | 9.6 | 30.3 | 2.1 | NR | 2.3^#^ | 15.9 |
| ter Bogt 2009 | GP usual care | 232 | 232 | 53.9 | 56.9 | 7.8 | 29.6 | 3.6 | NR | NR | 62.5 |
|  | Lifestyle counselling from NP | 225 | 225 | 49.8 | 55.3 | 7.7 | 29.5 | 3.1 | NR | NR | 60.9 |
| The Look AHEAD Research Group 2010 | Diabetes support and education | 2575 | 2575 | 59.6 | 58.9 | 6.9 | 36 | 5.8 | NR | 100.0 | 84.0 |
|  | Intensive lifestyle intervention | 2570 | 2570 | 59.3 | 58.6 | 6.8 | 35.9 | 6.0 | NR | 100.0 | 84.5 |
| Trepanowski 2017 | No-intervention control group | 31 | 31 | 87 | 44 | 11 | 34 | 4 | 0 | 0 | NR |
|  | Daily calorie restriction group | 35 | 35 | 83 | 43 | 12 | 35 | 4 | 0 | 0 | NR |
|  | Alternate-day fasting group | 34 | 34 | 88 | 44 | 10 | 34 | 4 | 0 | 0 | NR |
| Tsai 2010 | Control | 26 | 26 | 88 | 47.6 | 12.7 | 37.6 | 5.6 | NR | NR | NR |
|  | Brief counselling | 24 | 24 |  | 51.3 | 11.3 | 35.4 | 5.9 | NR | NR | NR |
| Tuomilehto 2009 | Control | 41 | 41 | 27 | 50.9 | 8.6 | 31.4 | 2.7 | NR | 7.3 | 36.6 |
|  | Intervention | 40 | 40 | 25.7 | 51.8 | 9.0 | 33.4 | 2.8 | NR | 10 | 45.0 |
| van de Glind 2017 | Comparison group | 553 | 553 | 0.0 | 45.6 | 8.7 | 33.4 | 4.7 | NR | NR | NR |
|  | EuroFIT group | 560 | 560 | 0.0 | 45.9 | 9.0 | 33.1 | 4.6 | NR | NR | NR |
| Viegener 1990 | Intermittent diet | 42 | 32 | 100 | 47.1 | 8.9 | NR | NR | NR | NR | NR |
|  | Standard treatment |  | 31 | 100 | 47.1 | 7.5 | NR | NR | NR | NR | NR |
| Vissers 2010 | Control | 21 | 21 | 74.7 | 44.8 | 11.4 | 30.8 | 3.4 | NR | NR | NR |
|  | Diet only group (Diet) | 20 | 20 |  | 45.5 | 13.1 | 32.9 | 3.1 | NR | NR | NR |
|  | Diet + fitness training group (Fitness) | 20 | 20 |  | 44.7 | 13 | 33.1 | 3.4 | NR | NR | NR |
|  | Diet + WBV group (Vibration) | 18 | 18 |  | 43.3 | 9.6 | 31.9 | 4.7 | NR | NR | NR |
| Volpe 2008 | Exercise only | 34 | 34 | 50 | 43.5 | 7.5 | 30.5 | 3.1 | NR | NR | NR |
|  | Diet only | 28 | 28 | 53.6 | 44.0 | 6.2 | 30.9 | 2.8 | NR | NR | NR |
|  | Combination of diet and exercise | 28 | 28 | 50 | 45.7 | 7.4 | 30.5 | 2.6 | NR | NR | NR |
| von Gruenigen 2012 | Control | 34 | 34 | 100 | 58.9 | 10.9 | 36.5 | 9.6 | NR | 26.5 | 35.3 |
|  | Intervention | 41 | 41 | 100 | 57.0 | 8.6 | 36.4 | 5.5 | NR | 14.1 | 31.8 |
| von Gruenigen 2008 | Control, Usual care | 22 | 22 | 100 | 55.4 | 7.5 | 41.1 | 10.3 | NR | NR | NR |
|  | Lifestyle intervention | 23 | 23 | 100 | 54.0 | 9.6 | 43.5 | 10.1 | NR | NR | NR |
| Wadden 1994 | Balanced deficit diet | 21 | 21 | 100 | 42.9 | 10.1 | 38.8 | 5.4 | NR | NR | NR |
|  | Very low-calorie diet | 28 | 28 | 100 | 36.8 | 8.9 | 40.0 | 5.7 | NR | NR | NR |
| Wadden 1998 | Diet alone, Control | NR | 29 | 100 | 41 | 8.8 | NR | NR | NR | 0.0 | NR |
|  | Diet plus aerobic exercise | NR | 31 | 100 | 40.8 | 7.9 | NR | NR | NR | 0.0 | NR |
|  | Diet plus strength training | NR | 31 | 100 | 40 | 9.1 | NR | NR | NR | 0.0 | NR |
|  | Diet plus aerobic and strength training | NR | 29 | 100 | 42.8 | 8.3 | NR | NR | NR | 0.0 | NR |
| Wadden 2004 | Nondieting approach | 39 | 39 | 100 | 43.9 | 10.2 | 35.5 | 4.3 | NR | NR | NR |
|  | Balanced-deficit diet | 43 | 43 | 100 | 45.6 | 9.2 | 36.3 | 4.9 | NR | NR | NR |
|  | Meal replacement plan | 41 | 41 | 100 | 43 | 10.5 | 36 | 4.2 | NR | NR | NR |
| Waleekhachonloet 2007 | Individual behavior therapy | 67 | 67 | 100 | 38.6 | 7.7 | 28.9 | 3.2 | NR | NR | NR |
|  | Group behavior therapy | 65 | 65 | 100 | 38.3 | 8.2 | 28.8 | 2.5 | NR | NR | NR |
| Weinstock 2013 | Conference Call DPP | 128 | 128 | 71.9 | 52.7 | 12.8 | 39.7 | 8.3 | NR | 0.0 | 66.1 |
|  | Individual Call DPP | 129 | 129 | 78.3 | 50.7 | 13.1 | 38.9 | 7.6 | NR |  |  |
| West 2007 | Attention control | 108 | 108 | NR | 52 | 10 | 36.5 | 5.4 | NR | 100.0 | NR |
|  | Motivational interviewing | 109 | 109 | NR | 54 | 10 | 36.5 | 5.5 | NR | 100.0 | NR |
| West 2011 | Control | 112 | 112 | 77 | 71.9 | 6.6 | 35.0 | 4.2 | NR | NR | NR |
|  | Lifestyle Intervention | 116 | 116 | 91 | 70.6 | 6.6 | 37.1 | 5.7 | NR | NR | NR |
| Whelton 1998 | Non-weight loss (Usual lifestyle, control group plus sodium reduction) | NR | NR | 57 | NR | NR | 31.1 | 2.4 | NR | NR | 100.0 |
|  | Weight loss (Weight loss alone plus weight loss and sodium reduction combined intervention) | NR | NR | 47 | NR | NR | 31.2 | 2.2 | NR | NR | 100.0 |
| Wilson 2016 | Control - Self Study Group | 242 | 147 | 59.9 | 46.6 | NR | 34.5 | NR | NR | NR | NR |
|  | Phone Fuel Your Life | 182 | 106 | 67.9 | 47.8 | NR | 33.6 | NR | NR | NR | NR |
|  | Group Fuel Your Life | 236 | 165 | 58.2 | 45.9 | NR | 32.7 | NR | NR | NR | NR |
| Wilson 2016b | Control | 457 | 457 | 6.3 | 47 | NR | 29.9 | 5.6 | NR | NR | NR |
|  | FUEL Your Life peer health coaches + nurse education | 459 | 459 | 5.4 | 44 | NR | 31.9 | 5.4 | NR | NR | NR |
| Wing 1988 | Diet plus placebo exercise | 13 | 13 | 84 | 52.5 | 8.9 | 37.5 | 6.2 | NR | 100.0 | NR |
|  | Diet plus moderate exercise | 12 | 12 |  | 56.2 | 7.5 | 38.1 | 6.4 | NR | 100.0 | NR |
| Wing 1988b | Diet only | 15 | 15 | 70.0 | 55.1 | 7.2 | 37.9 | 6.5 | NR | 100.0 | NR |
|  | Diet plus exercise | 15 | 15 |  | 56.1 | 6.4 | 38.2 | 6.6 | NR |  | NR |
| Wing 1998 | Control | 40 | 40 | 80 | 45.3 | 4.9 | 36.0 | 5.4 | NR | 0.0 | NR |
|  | Diet | 37 | 37 | 78 | 45.0 | 4.7 | 36.1 | 4.1 | NR | 0.0 | NR |
|  | Exercise | 37 | 37 | 81 | 46.4 | 4.5 | 36 | 3.7 | NR | 0.0 | NR |
|  | Diet plus exercise | 40 | 40 | 77 | 46.3 | 3.8 | 35.7 | 4.1 | NR | 0.0 | NR |
| Wing 2010 | Structured Education Program | 112 | 112 | 100 | 53 | 10 | 36 | 5 | NR | 0.9 | NR |
|  | Weight Loss Intervention (Skills Based maintenance) | 113 | 113 | 100 | 52 | 10 | 37 | 6 | NR | NR | NR |
|  | Weight Loss Intervention (Motivation Based maintenance) | 113 | 113 | 100 | 53 | 11 | 36 | 5 | NR | NR | NR |
| Yannakoulia 2008 | Usual care group | 15 | 15 | 53.3 | 56.9 | 10 | 31.6 | 5 | NR | 100 | NR |
|  | Intensive care group | 15 | 15 | 40.0 | 56.3 | 8.8 | 32.2 | 4.1 | NR | 100 | NR |
| Yardley 2014 | Usual care | 43 | 43 | 65.1 | 49.9 | 13.8 | 36.2 | 4.9 | NR | NR | NR |
|  | Web-based only | 45 | 45 | 68.9 | 51.2 | 13.9 | 34.8 | 4.4 | NR | NR | NR |
|  | Basic nurse support | 44 | 44 | 65.9 | 51.4 | 13.0 | 36.4 | 6.5 | NR | NR | NR |
|  | Regular nurse support | 47 | 47 | 63.8 | 52.1 | 12.7 | 35.4 | 6.0 | NR | NR | NR |
| Yates 2018 | Placebo + no lifestyle | 8 | 8 | 100 | 60 | 4.5 | 36.7 | 5.5 | 3.4 | NR | 50.0 |
|  | Metformin + no lifestyle (N/A) | 7 | 7 |  | N/A | N/A | N/A | N/A |  | N/A | N/A |
|  | Placebo + lifestyle | 7 | 7 |  | 57.1 | 3.3 | 39.7 | 5.1 |  | NR | 28.6 |
|  | Metformin + lifestyle (N/A) | 7 | 7 |  | N/A | N/A | N/A | N/A |  | N/A | N/A |
| Yeh 2003 | Counseling based intervention | 40 | 37 | 100 | 51 | 11 | 36.3 | 5.4 | NR | 0.0 | NR |
|  | Skills based intervention | 40 | 35 | 100 | 48 | 9 | 37.9 | 6.7 | NR | 0.0 | NR |
| Yin 2018 | Comparison-Control Group | 75 | 75 | 100 | 53.27 | 7.17 | 27.43 | 2.75 | NR | NR | 24.0 |
|  | Intervention Group | 109 | 109 | 100 | 51.06 | 7.15 | 27.42 | 2.91 | NR | NR | 27.5 |
| Zhang 2016 | Control | 74 | 74 | 62.2 | 54 | 6.8 | 28 | 2.7 | NR | NR | NR |
|  | Moderate exercise | 73 | 73 | 69.9 | 54.4 | 7.4 | 28.1 | 3.3 | NR | NR | NR |
|  | Vigorous-moderate exercise | 73 | 73 | 71.2 | 53.2 | 7.1 | 27.9 | 2.7 | NR | NR | NR |
| **CBT:** Cognitive Behaviour Therapy; **CV** = Cardiovascular; **DM:** Diabetes Mellitus; **DPP:** Diabetes Prevention Program**; N/A** = Not applicable; **NR** = Not reported; **VLCD:** Very low calorie diet  * Comorbidity definitions varied for each study; ^#^Unclear whether DM percentage listed includes Type II and Type I  ^a^ Median (IQR); ^b^ Standard error; ^c^ Range; ^d^ 95% Confidence intervals  ^Study only included in the CNMA involving intensity components. | | | | | | | | | | | |

## **Supplemental Table S5.** Intervention characteristics

| **Study ID** | **Groups:** | **Intervention type** | **Intervention faded in intensity** | **Features**  (meal replacements, nutrition education, financial incentives, intermittent fasting, content designed to help participants following programme end) | **Provider ^a^** | **Provider training received** | **Delivery** | | **Intervention setting** | **Intervention timing (months)** | | **Sessions** | | | **Intervention personalised, titrated or adapted** |
| --- | --- | --- | --- | --- | --- | --- | --- | --- | --- | --- | --- | --- | --- | --- | --- |
|  |  |  |  |  |  |  | **Mode** | **Format** |  | **Last contact** | **End**  **(step change in intensity)** | **N^c^** | **Frequency** | **Length per session with description for varying lengths.  (minutes** ^b^**)** |  |
| Abed 2013 | Control | Control | No | Nutrition Edu.; Help following programme end |  | No |  |  | Health Care |  |  | 0 |  |  | No |
|  | Weight Management | Diet and exercise | Yes | MR-P; Nutrition Edu.; Help following programme end | Physician | No | Individual | Face to Face; Print | Health Care | 15 | 2 | 5 | Every 3 months | 20 - 40 mins; Additional goal-directed face-to-face clinic visits were scheduled as required;  8 weeks VLCD. 3m to 15m low GI meals. Exercise plan of increasing intensity.  Exercise 3 per week for 15 months; 24-hour e-mail and telephone support provided as required. | Yes |
| Ackermann 2011 | Standard advice alone (controls) | Diet and exercise | No | Nutrition Edu. |  |  | Individual | Face to Face | Community | 0 | 0 | 1 | Once | 5 | Yes |
|  | YMCA DPP intervention | Diet and exercise | No | Nutrition Edu. |  |  | Individual and Group | Face to Face | Community | 5 | 5 | 16 | Weekly | 60 – 90 | Yes |
| Agras 1990 | Computer alone | Diet and exercise | No | Fin. Incentives |  | No | Other - remote, plus one group session | Other | Home | 3 | 3 |  | Daily | N/A as computer which they had on them at all times | Yes |
|  | Computer + group support | Diet and exercise | Yes | Fin. Incentives |  | Unclear | Other - computer and group support | Face to Face; Other | Community; Home | 3 | 3 | 4 | Every second week | 2, 4, 6 and 8 weeks | Yes |
|  | Behaviour therapy | Diet and exercise | No | Fin. Incentives |  | No | Group | Face to Face | Community | 3 | 3 | 10 | Approx. weekly | 10 sessions over 12 weeks | No |
| Ahern 2017 | Brief intervention | Control | No | Nutrition Edu. | Other | Unclear | Individual | Face to Face; Print | Community | 1 | 1 | 1 | Once |  | No |
|  | 12-week behavioural weight-loss programme | Diet and exercise | No | Nutrition Edu. | Health Trainer | Yes | Group | Face to Face; Internet | Community | 12 | 12 | 12 | Weekly | 30 | No |
|  | 52-week behavioural weight-loss programme | Diet and exercise | No | Nutrition Edu. | Health Trainer | Yes | Group | Face to Face; Internet | Community | 52 | 52 | 52 | Weekly | 30 | No |
| Almanza -Aguilera 2018 | Control (general recommendations) | Control | No | Nutrition Edu. | Nutritionist | No | Unclear | Face to Face; Other |  | 12 |  | 2 | 3m, 12m |  | No |
|  | Treatment (lifestyle weight loss intervention) | Diet and exercise | No | Nutrition Edu. | Nutritionist | No | Unclear | Face to Face; Other |  | 12 | 3 | 13 | Weekly (0-3m); Once at 12m |  | No |
| Anderson 2014 | Control (weight loss booklet only) | Control | No | Nutrition Edu.; Help following programme end |  | No | Individual | Print | Home | 12 | 12 | 0 |  |  | No |
|  | Intervention (BeWEL) | Diet and exercise | Yes | Nutrition Edu.; Help following programme end | Psychologist/ Counsellor | No | Individual | Face to Face; Telephone; Print | Health Care; Home | 3 | 12 | 12 | Monthly | The 3 counsellor sessions were each 1 hour. The 9 phone calls were each 15 minutes. | Yes |
| Annesi 2016 | Comparison treatment | Diet and exercise | No | Nutrition Edu. |  | Yes | Individual | Telephone; Print | Community | 24 | 6 | 12 | Every 2 weeks | 15 | Yes |
|  | Experimental treatment | Diet and exercise | No | Nutrition Edu.; Help following programme end | Psychologist/ Counsellor | Yes | Individual and Group | Face to Face; Internet | Community | 24 | 6.5 | 32 | Exercise support sessions: over 6.5 months; Nutrition sessions: every 2 weeks | Six 45-minute individual exercise meetings; 10 nutrition sessions of 60 minutes | Yes |
| Annesi 2017 | Control comparison group | Diet and exercise | No | Nutrition Edu.; Inter. Fasting |  | Yes | Individual | Telephone; Print | Community | 6 | 6 | 17 | Every second week (24 weeks in total) | Lesson followed by 15 min phone conversation | Yes |
|  | Experimental group | Diet and exercise | Yes | Nutrition Edu.; Help following programme end | Health Trainer | Yes | Individual and Group | Face to Face | Community | 12 | 6 | 35 | Monthly 1:1 for 6m. Biweekly from weeks 10-52 | 45 mins for 1:1 sessions; Group length not stated | Yes |
| Appel 2011 | Control (Self-directed) | Control | No | Nutrition Edu. | Health Trainer | Unclear | Individual | Face to Face; Print | Health Care |  |  | 1 | 0, 24m |  |  |
|  | Remote Support Only (N/A) | N/A | N/A | N/A | N/A | N/A | N/A | N/A | N/A | N/A | N/A | N/A | N/A | N/A | N/A |
|  | In-Person Support | Diet and exercise | Yes | Nutrition Edu. | Other AHPs; Health Trainer | Yes | Individual and Group | Face to Face; Telephone; Internet; Other | Health Care | 24 | 6 | 57 | Weekly (0-3m); Three monthly contacts over the next 3 months; Two monthly contacts for the remainder of the study. | Individual sessions approx. 20 mins; In-person group sessions: 90 mins | Yes |
| Ard 2018 | Exercise Only | Exercise only | No |  |  | Unclear | Group | Face to Face; Print | Community | 12 | 6 | 38 | Weekly 0-24 weeks and then biweekly until 12m | 1 hour | No |
|  | Exercise + Diet Quality + Weight Maintenance | Diet and exercise | No | Nutrition Edu. |  | Unclear | Group | Face to Face; Print; | Community | 12 | 6 | 38 | Weekly 0-24 weeks and then biweekly until 12m | 1 hour | No |
|  | Exercise + Diet Quality + Weight Loss | Diet and exercise | No | Nutrition Edu. |  | Unclear | Group | Face to Face; Print | Community | 12 | 6 | 38 | Weekly 0-24 weeks and then biweekly until 12 m | 1 hour | No |
| Ash 2006 | Control Group - Booklet only | Control | No | Nutrition Edu. |  | Yes | Other – booklet only | Print | Community |  |  | 0 |  |  | No |
|  | Individualised Dietetic Treatment | Diet and exercise | Yes | Nutrition Edu. | Dietitian | Yes | Individual | Face to Face; Telephone; Print | Health Care; Community | 6 | 2 | 12 | Weekly for 8 weeks, monthly from week 8 until 6m. | 1 hour initial individual consultation, followed by seven 20-min weekly review sessions and four monthly follow-up sessions | Yes |
|  | Fat Booters Incorporated | Diet and exercise | Yes | Nutrition Edu.; Help following programme end | Dietitian; Nutritionist | Yes | Group | Face to Face; Telephone; Print | Health Care; Community | 6 | 2 | 11 | Weekly for 6 weeks with monthly follow up until 6m. | 1.5 hrs for 6 sessions. Not stated for 5 follow up visits. | No |
| Ashley 2001 | Control, Diet | Diet and exercise | Yes | Nutrition Edu.; Help following programme end | Dietitian | Unclear | Group | Face to Face; Print | Community | 24 | 12 | 37 | Year 1: weekly for 3m, biweekly for 3m, monthly for 6m. Year 2: monthly | 60 | No |
|  | MR - Physician/Nurse led | Diet and exercise | Yes | MR-P; Nutrition Edu.; Help following programme end | Nurse (General); Physician | Unclear | Group | Face to Face; Print | Community | 24 | 12 | 37 | Year 1: every other week.  Year 2: monthly | 15 | Yes |
|  | MR - Dietician lead | Diet and exercise | Yes | MR-P; Nutrition Edu.; Help following programme end | Dietitian | Unclear | Group | Face to Face; Print | Community | 24 | 12 | 37 | Year 1: weekly for 3m, biweekly for 3m, monthly for 6m. Year 2: monthly | 60 | Yes |
| Ashley 2007 | Control, TFG - Traditional Food Group | Diet only | Yes | Nutrition Edu. | Dietitian | Yes | Group | Face to Face; Print | Community | 12 | 6 | 18 | 0- 6m: 2 x per month  6-12m: monthly |  | Yes |
|  | MRG - Meal Replacement Group | Diet only | Yes | MR-P; Nutrition Edu. | Dietitian | Yes | Group | Face to Face; Print | Community | 12 | 6 | 18 | 0- 6m: 2 x per month 6-12m: monthly |  | Yes |
| Aveyard 2016 | Advice only | Control | No |  | GP | Yes | Individual | Face to Face | Health Care | 0 | 0 | 1 |  | 30 seconds | No |
|  | Advice plus weight loss programme | Diet and exercise | No | Nutrition Edu. | GP; Health Trainer | Yes | Individual | Face to Face | Health Care; Community | 3 | 3 | 12 | Weekly | 60 | No |
| Bacon 2002 | Health at Every Size - control | Diet and exercise | No | Nutrition Edu. | Psychologist/ Counsellor |  | Group | Face to Face | Community | 12 | 6 | 30 | Weekly for first 6m, then monthly | 1.5 hour | No |
|  | Diet Group - intervention | Diet and exercise | No | Nutrition Edu. | Dietitian |  | Group | Face to Face | Community | 12 | 6 | 30 | Weekly for first 6m, then monthly | 1.5 hour | No |
| Bartels 2015 | Control, Fitness club membership | Exercise only | No |  |  | No | Individual | Face to Face | Community |  |  | 1 |  |  | No |
|  | IN SHAPE | Diet and exercise | No | Nutrition Edu. | Health Trainer; Personal Trainer | Yes | Individual | Face to Face | Community | 12 | 12 | 52 | Weekly | 45 – 60 | Yes |
| Beeken 2017 | Usual care | Diet and exercise | No | Nutrition Edu. | Health care professional (not specified) | No | Individual and Group | Other | Health Care; Community | Varied (12 weeks, min. 2 appts, 12 weekly sessions, monthly appts) | varied | Varied |  |  | Yes |
|  | 10TT | Diet and exercise | No | Nutrition Edu.; Help following programme end | Nurse (General) | Yes | Unclear | Face to Face; Telephone; Print | Health Care | 3 | 3 | 1 | Single session | 30 | No |
| Bennett 2012 | Control, Usual care | Diet only | No |  |  | No | Other – Print only | Print |  |  |  |  |  |  | No |
|  | Be Fit, Be Well | Diet and exercise | Yes | Nutrition Edu. | Health care professional (not specified) | Yes | Individual and Group | Face to Face; Telephone; Internet | Community; Home | 24 | 12 | 30 | Monthly for the first year and bimonthly for the second year. Additional 12 optional monthly group sessions | 15-20 mins;  Telephone counselling sessions were held monthly for the first year and bimonthly for the second year. There were an additional 12 optional monthly group sessions | Yes |
| Bennett 2013 | Control, usual care | Control | No |  |  | No |  |  |  | 12 |  |  |  |  | No |
|  | Weight gain prevention intervention | Diet and exercise | No | Nutrition Edu. |  | Yes | Individual | Telephone; | Community; Home | 12 | 12 | 64 | Weekly (52) and monthly (12) | 10 mins (52 weekly IVR (interactive voice response calls))  12 monthly 20 min calls | Yes |
| Berry 2014 | Control | Control | No |  |  | No |  |  |  |  |  |  |  |  | No |
|  | Family based. Nutrition, exercise and coping skills intervention | Diet and exercise | No | Nutrition Edu. | Nurse (General); Dietitian; Personal Trainer | No | Group | Face to Face | Community | 12 | 3 | 21 | Weekly for 3m; Monthly until 12m | Weekly for 12 weeks. 60 mins nutrition and exercise education and coping skills followed by 45 mins of exercise. Monthly for 9m 60 mins class and 45 mins exercise. | No |
| Bertz 2012 | Control | Control | No |  |  | No |  |  |  |  |  |  |  |  | No |
|  | Diet Only | Diet only | No | Nutrition Edu. | Dietitian | No | Individual | Face to Face; Telephone; SMS | Health Care; Community; Home | 3 | 3 | 2 | Week 0, 6 | 1.5 hrs at start of intervention, 1hr at week 6 | Yes |
|  | Exercise only | Exercise only | No |  | Physiotherapist | No | Individual | Face to Face; Telephone; SMS | Health Care; Community; Home | 3 | 3 | 2 | Week 0, 6 | 1.5 hours at start of intervention, 1 hour at week 6 | No |
|  | Intervention | Diet and exercise | No | Nutrition Edu. | Dietitian; Physiotherapist | No | Individual | Face to Face; Telephone; SMS | Health Care; Community; Home | 3 | 3 | 4 | Week 0, 6 | 2 x 1.5 hours at start of intervention,  2 x 1 hour at week 6 | Yes |
| Beutel 2006 | Behavioural therapy | Diet and exercise | No | Help following programme end | Physician; Psychologist/ Counsellor | Yes | Group | Face to Face | Inpatient | 1.5 | 1.5 |  |  | 40 | Yes |
|  | Psychodynamic treatment | Diet and exercise | No |  | Physician; Psychologist/ Counsellor | Yes | Individual and Group | Face to Face | Inpatient | 1.5 | 1.5 |  |  | 40 | Yes |
| Bliddal 2011 | Control, low-energy diet | Diet only | Yes | Nutrition Edu. | Dietitian | Unclear | Group | Face to Face | Community |  |  | 5 | Weeks: 0, 8, 32, 36, 52 | 2 hours | No |
|  | Intensive low-energy diet | Diet only | Yes | MR-F; Nutrition Edu. | Dietitian | Unclear | Group | Face to Face | Community; Home | 12 | 8.3 | 44 | Baseline, weekly for 32 weeks then every 2 weeks | 1.5 hours | No |
| Bo 2007 | Control standard care | Control | No | Nutrition Edu. | GP | Yes | Individual | Face to Face | Health Care | 0 |  | 1 |  |  | No |
|  | Intervention lifestyle by trained professional | Diet and exercise | No | Nutrition Edu. | Physician; Nutritionist | Yes | Individual and Group | Face to Face; Print | Health Care; Community | 12 | 12 | 6 |  | 60 | Yes |
| Brown 2014 | Control | Control | No |  |  | No |  |  |  |  |  |  |  |  | No |
|  | RENEW | Diet and exercise | Yes | MR-P; Nutrition Edu. | Nurse (General); Other AHPs; Personal Trainer | No | Group | Face to Face; Telephone; Print | Health Care; Home | 12 | 3 | 63 | Weekly | Weeks 1-12 intensive intervention (weekly 3 hour intervention; 2 meal replacements per day, 1 hour exercise 2 x per week).  Weeks 13-24 maintenance phase (3 hour monthly session, 1 hour exercise 2 x per week, weekly phone calls, weekly newsletter). Weeks 25-52 intermittent support (weekly phone calls and monthly mailings). | No |
| Burke 2015 | Standard behavioural weight loss treatment | Diet and exercise | Yes | Nutrition Edu. | Health care professional (not specified) | No | Group | Face to Face | Community; Home | 18 | 12 | 20 | Weekly the first month, biweekly the second month, monthly for next 10 months, and every 6 weeks for 13-18m. | 1 hour group sessions |  |
|  | Self-efficacy enhancement plus standard behavioural weight loss treatment | Diet and exercise | Yes | Nutrition Edu. | Health care professional (not specified) | No | Individual and Group | Face to Face; Telephone | Community; Home | 18 | 12 | 50 | SE - 1:1 every 2 weeks for first 12m. Then at least monthly. SBT: weekly the first month, biweekly the second month, monthly for next 10 months, and every 6 weeks for months 13-18. | 1 hour group session. 1:1 sessions: 23 mins. |  |
| Chee 2017 | Usual Care | Diet and exercise | Yes | Nutrition Edu. | Dietitian | Unclear | Unclear | Face to Face; Print | Health Care | 6 | 12 | 4 | Every 3m |  | Yes |
|  | tDNA Conventional Counseling | Diet and exercise | Yes | MR-P; Nutrition Edu. | Physician; Dietitian | Unclear | Unclear | Face to Face; Print | Health Care | 6 | 12 | 8 | Monthly 0-6m then every 3m |  | Yes |
|  | tDNA Motivational Interviewing | Diet and exercise | Yes | MR-P; Nutrition Edu. | Physician; Dietitian | Unclear | Unclear | Face to Face; Print | Health Care | 6 | 12 | 8 | Monthly 0-6m then every 3m |  | Yes |
| Cheyette 2007 | Control | Diet only | No | Nutrition Edu. | Dietitian | No | Individual | Face to Face | Health Care |  |  | 1 minimum | Annually |  | No |
|  | Weight No More intervention group | Diet and exercise | No | Nutrition Edu.; Help following programme end | Dietitian; Physiotherapist | Unclear | Group | Face to Face; Print | Community | 4 | 4 | 8 | Fortnightly | 1.5 hours | No |
| Christensen 2012 | Reference group | Control | No | Nutrition Edu. |  | No | Group | Face to Face |  | 12 |  | 12 | Monthly | 2 hours | No |
|  | Intervention group | Diet and exercise | No | Nutrition Edu.; Help following programme end |  | No | Group | Face to Face | Workplace | 12 | 3 | 48 | Weekly | 1 hour; Participants also instructed to spend additional personal time doing physical exercise (see 'Procedures') | Yes |
| Cleo 2018 | TTT Top Ten Tips habit formation | Diet and exercise | No |  |  | No | Group | Face to Face; Print | Community; Home | 3 | 3 | 13 | Weekly phone calls | 2 hrs group induction; Call length not stated. | No |
|  | DSD Do Something Different online software | Diet and exercise | No |  |  | No | Individual and Group | Face to Face; Telephone; Internet | Community; Home | 3 | 3 | 13 | Weekly tasks and phone calls | 2 hrs group induction. Call length not stated. tasks, length not stated | Yes |
|  | Wait list control (N/A) | N/A | N/A | N/A | N/A | N/A | N/A | N/A | N/A | N/A | N/A | N/A | N/A | N/A | N/A |
| Cole 2013 | Control - individualised counselling | Diet and exercise | No | Nutrition Edu. | Dietitian | No | Individual | Face to Face | Health Care | 3 | 3 | 1 | At least 1 session over 3m | 45 – 60 | No |
|  | Intervention- shared medical appointment | Diet and exercise | No | Nutrition Edu. | Nurse (General); Dietitian; Nutritionist | No | Group | Face to Face | Health Care | 3 | 3 | 3 | 3 sessions over 3m | 90 | No |
| Conroy 2015 | Self-guided | Diet and exercise | No | Nutrition Edu. | Other | No | Other - Self-guided manual | Print | Home | 3 | 3 |  |  | 12-week self-guided manual | No |
|  | Interventionist led | Diet and exercise | No | Nutrition Edu. | Physician; Other | No | Group | Face to Face | Health Care | 3 | 3 | 12 | Weekly | 60 mins | No |
| Cooper 2010 | Guided Self-Help Control | Control | No | Nutrition Edu. | Psychologist/ Counsellor | Yes | Individual | Face to Face; Telephone | Health Care; Home | 5.5 | 5.5 | 17 |  | 20 mins;  'lasted 24 weeks and involved two initial face-to-face sessions with a therapist followed by up to 15 20-min telephone sessions.' |  |
|  | Behaviour Therapy | Diet only | Yes | Nutrition Edu.; Help following programme end | Physician; Psychologist/ Counsellor; Dietitian | Yes | Individual | Face to Face | Health Care | 10 | 10 | 24 | Weekly for the first 7 weeks and every 2 weeks from week 8 to 44. | 50 mins; Weight loss phase lasted until week 24-30. |  |
|  | Cognitive Behaviour Therapy | Diet and exercise | Yes | Nutrition Edu; Help following programme end | Physician; Psychologist/ Counsellor; Dietitian | Yes | Individual | Face to Face | Health Care | 10 | 10 | 24 | Weekly for the first 7 weeks and every 2 weeks from 8 to week 44. | 50 mins;  Weight loss phase lasted until week 24-30. |  |
| Cousins 1992 | Control | Diet and exercise | No | Nutrition Edu.; Help following programme end |  | No | Other | Print | Home |  |  |  |  |  | No |
|  | Individual | Diet and exercise | Yes | Nutrition Edu.; Help following programme end | Dietitian | No | Group | Face to Face; Print | Community | 12 | 6 | 30 | 24 x weekly, then 6 x monthly |  | Yes |
|  | Family | Diet and exercise | Yes | Nutrition Edu. | Dietitian | No | Group | Face to Face; Print | Community | 12 | 6 | 30 | 24 x weekly, then 6 x monthly |  | Yes |
| Craighead 1989 | Control, minimal contact | Diet and exercise | No | Nutrition Edu. |  | No | Other - 12 written lessons with feedback | Print | Home | 12 | 6 |  |  |  | No |
|  | Contracted Exercise | Diet and exercise | No | Nutrition Edu. | Other | No | Group | Face to Face; Print | Community | 12 | 6 | 12 | Weekly | 60 | No |
|  | Supervised Exercise | Diet and exercise | No | Nutrition Edu. | Psychologist/ Counsellor; Other | No | Group | Face to Face; Print | Community | 12 | 6 | 33 | Weekly group meetings from week 5. 3 x per week from week 5 to 12 | 60 mins 1 x per week for 12 weeks. 40 mins 3 x per week for 8 weeks (from week 5 to week 12). | Yes |
| Crowley 2017 | Group Medical Visit |  | Yes |  | Nurse (General); GP; Physician; Dietitian | Unclear | Individual and Group | Face to Face; Print | Health Care | 11.1 | 3.7 | 8 | Every 4 weeks for 16 weeks and every 8 weeks until week 48 | 1.5-2 hours | Yes |
|  | Intensive Weight Management Group Medical Visit | Diet and exercise | Yes | Nutrition Edu.; Help following programme end | Nurse (General); GP; Physician; Dietitian | Unclear | Individual and Group | Face to Face; Print | Health Care | 11.1 | 3.7 | 16 | Every 2 weeks for 16 weeks and every 8 weeks until week 48 | 1.5-2 hours | Yes |
| Dale 2009 | Control | Control | No |  |  | No |  |  |  |  |  |  |  |  | No |
|  | Modest | Diet and exercise | No | MR-F; Nutrition Edu. | Dietitian; Exercise physiologist; | Unclear | Individual and Group | Face to Face; Telephone; | Community | 4 | 4 | 36 | Twice weekly |  | Yes |
|  | Intensive intervention | Diet and exercise | No | Nutrition Edu. | Dietitian; Exercise physiologist | Unclear | Individual and Group | Face to Face; Telephone | Community | 4 | 4 | 36 | Twice weekly |  | Yes |
| Dalziel 2006 | Control | Control | No | Nutrition Edu. | Physician; Dietitian | No | Unclear | Face to Face | Health Care |  |  |  |  |  | No |
|  | Experimental | Diet only | No | Nutrition Edu. | Physician; Dietitian | No | Unclear | Face to Face | Health Care | 24 | 2 | 3 | At 8 weeks, then annually from baseline | 1 hour first session, length of follow-up sessions not reported. | No |
| Damschroder 2014 | Control, MOVE - usual care | Diet and exercise | Yes | Nutrition Edu. | Nurse (General); Psychologist/ Counsellor; Dietitian; Physiotherapist | Unclear | Group | Face to Face | Health Care | 24 | 3 | 58 | Weekly for 3m, then either quarterly or twice monthly | 11-12 weekly open-group sessions of 90 mins each over 3 months. During months 4-12, one group met quarterly for 90 minutes and the other groups met twice a month for 60 minutes. Some participants had the option of re-enrolling in the initial series of weekly sessions. Total hours over the year ranged from 22 to 35 hours. 12-24 mths as above for mths 4-12. | Yes |
|  | ASPIRE group, individual telephone counselling | Diet and exercise | Yes | Nutrition Edu. | Health Trainer | Yes | Individual | Telephone | Home | 24 | 3 | 34 | Same as ASPIRE-Group but duration of sessions varied | Up to 30 mins for the first 3 mths and 20 mins for the remaining 9 mths, totalling 11 hours across the year. 12-24 mths coaching every other mth, 6 sessions. | Yes |
|  | ASPIRE group, group counselling | Diet and exercise | Yes | Nutrition Edu. Help following programme end | Health Trainer | Yes | Group | Face to Face | Health Care | 24 | 3 | 34 | Both ASPIRE small-changes treatment arms consisted of weekly sessions for 3m, followed by 6m of sessions every other week, and then 3 monthly sessions over 12 months, for a total of 28 sessions. | Up to 90 mins for the first 3 mths and 60 mins for the remaining 9 mths, totalling 33 hours across the year. 12-24 mths coaching every other mth, 6 sessions. | Yes |
| Daubenmier 2016^ | Active control intervention | Diet and exercise | Yes | Nutrition Edu. | Dietitian | Unclear | Group | Face to Face; Print | Community | 5.5 | 5.5 | 16 | 12 weekly then biweekly for 3 sessions, and then one session one month later plus a single all-day weekend session | 16 sessions: 2 hours; 5-hour all-day session | Yes |
|  | Mindfulness Intervention | Diet and exercise | Yes | Nutrition Edu.; Help following programme end | Dietitian | Yes | Group | Face to Face; Print | Community | 5.5 | 5.5 | 16 | 12 weekly then biweekly for 3 sessions, and then one session one month later plus a single all-day weekend session | 16 sessions: 2.5 hours; 6.5 hour all day session | Yes |
| Delahanty 2015 | Dietitian Referral group | Diet and exercise | No | Nutrition Edu. |  | Unclear | Individual | Face to Face; Print | Health Care |  |  | 1 + | Individualised – 1 session plus follow-up sessions on individual basis at dietitian’s discretion | Initial session (1 hour); follow-up sessions (20-40 minutes) | Yes |
|  | Group lifestyle intervention | Diet and exercise | No | MR-P; Nutrition Edu. | Physician; Dietitian | Yes | Group | Face to Face; Print | Health Care | 6 | 6 | 19 | Weekly | 1.5 hours | No |
| deRoon 2017 | Control | Control | No |  |  | No |  | Telephone | Home | 4 |  |  |  |  | No |
|  | Diet | Diet only | No | Nutrition Edu. | Dietitian | No | Individual and Group | Face to Face; Telephone; | Community; Home | 12 | 4 |  | Weekly |  | No |
|  | Exercise | Diet and exercise | No |  | Dietitian; Physiotherapist | No | Group | Face to Face; Telephone | Community; Home | 12 | 4 | 32 | 2 x week groups sessions; 2 x Nordic walking | 4 hours per week exercise program;  two x 1 hour group sessions;  2 x 1 hour sessions Nordic walking per week - individual home-based exercise with ‘Supervised lessons of Nordic walking by instructors are organised to increase motivation  and compliance.” (4 hours per week for 16 weeks); Unclear if all Nordic walking sessions are organised - did not contribute to total N of sessions | No |
| deVos 2016 | Control | Control | No |  |  | No |  |  |  |  |  |  |  |  |  |
|  | Tailor-made lifestyle intervention | Diet and exercise | No | Nutrition Edu. | Dietitian; Physiotherapist | No | Individual and Group | Face to Face | Community | 30 | 6 | 23 + |  | First 3 dietician appointments were biweekly, after that the frequency of visits was determined by mutual agreement. Invited to attend 20 weekly physical activity classes.  "...definition of compliance as attendance at ≥ 6 dietitian visits and ≥7 physical activity classes." | Yes |
| Diabetes Prevention Program R G 2009 | Placebo | Diet and exercise | No | Nutrition Edu. | Other |  | Individual | Face to Face; Print |  | 36 | 36 | 4 | Annually | 20 – 30 |  |
|  | Metformin (N/A) | N/A | N/A | N/A | N/A | N/A | N/A | N/A | N/A | N/A | N/A | N/A | N/A | N/A | N/A |
|  | Lifestyle | Diet and exercise | Yes | MR-P; Nutrition Edu.; Help following programme end | Psychologist/ Counsellor; Dietitian; Exercise physiologist; Health Trainer | Yes | Individual and Group | Face to Face; Telephone; Print | Community | 36 | 6 | 358 | 16 sessions in first 24 weeks then monthly. [At least 2 exercise classes per week] | 45 mins;  Core curriculum sessions 30-60 mins;  Sessions: 16+6+12+12=46. [Physical activity 2x52 x 3 years = 312] | Yes |
| Djuric 2002 | Control | Control | No | Nutrition Edu. | Dietitian | No | Unclear | Print |  | 0 | 0 | 1 |  |  |  |
|  | Weight Watchers | Diet and exercise | No | Nutrition Edu. | Dietitian | Yes | Group | Face to Face | Community | 12 | 12 | 52 | Weekly |  |  |
|  | Individualized group | Diet and exercise | Yes | Nutrition Edu. | Dietitian |  | Individual and Group | Face to Face; Telephone; Print | Community | 12 | 3 | 21 | 0-3m: weekly;  3-6m: every other week;  6-12m: monthly |  | Yes |
|  | Comprehensive group | Diet and exercise | Yes | Nutrition Edu. | Dietitian |  | Individual and Group | Face to Face; Telephone | Community | 12 | 3 | 73 | Weekly |  | Yes |
| Duncan 2016 | Control | Control | No |  | GP | No | Individual | Face to Face | Health Care |  |  | 1 |  |  | No |
|  | Intervention | Diet and exercise | No | Nutrition Edu.; Help following programme end | Health Trainer | Yes | Individual | Face to Face | Home | 4 | 4 | 6 | Ranged from 1-4 weeks | 60 | Yes |
| Eaton 2016 | Control, Standard Intervention | Diet and exercise | Yes | Nutrition Edu. | Physician; Psychologist/ Counsellor | Yes | Individual | Face to Face; Print | Community; Home | 18 | 4 | 3 | In person sessions at 0, 6, 12m. Mailing at 1, 2, 4, 15 & 18m | 90 | No |
|  | Enhanced Intervention | Diet and exercise | Yes | Nutrition Edu. | Physician; Psychologist/ Counsellor; Dietitian | Yes | Individual | Face to Face; Telephone; Print; Video | Community; Home | 24 | 6 | 30 | 0-6m: weekly feedback & mailings, monthly phone, 2 DVDs.  6-12m: weekly mailings, bi-monthly phone.  12-18m: bimonthly mailings, 2 DVDs 1 phone.  18-24m: monthly mailing. | 90 | Yes |
| Fahey 2018 | Self-paced condition | Diet and exercise | Yes | Nutrition Edu. |  | Yes | Individual | Telephone; Internet; Print | Home | 12 | 4 | 28 | Same schedule as per intervention group available, however upon self-initiation. | Telephone call length not stated; Aimed for 225-250 mins of weekly exercise goal | Yes |
|  | Counselor- initiated condition | Diet and exercise | Yes | MR-P; Nutrition Edu. |  | Yes | Individual | Telephone; Internet | Home | 12 | 4 | Up to 28 | 0-4m: weekly  5-8m: 2 x per month  9-12m: 1 x per month | Telephone call length not stated; Aimed for 225-250 minutes of weekly exercise goal | Yes |
| Fernandez-Ruiz 2018 | Control | Control | No |  |  | Unclear |  |  |  |  |  |  |  |  | No |
|  | Intervention (healthy eating, exercise & CBT) | Diet and exercise | No | Nutrition Edu. | Nurse (General); Physician; Psychologist/ Counsellor; Nutritionist; Exercise physiologist | Unclear | Individual and Group | Face to Face | Health Care | 12 | 12 | 232 | 4 x per week physical activity; monthly CBT & health ed. | 208 exercise sessions: 4 x per week, 40 mins. CBT 12 sessions, 1 per month, 60 mins. Health education (nurse) 12 sessions, 1 per month, 60 mins. | Yes |
| Finkelstein 2017 | Control | Diet and exercise | No | Nutrition Edu. | Physician; Dietitian; Physiotherapist | No | Individual and Group | Face to Face | Health Care; Home | 4 | 4 | 8 | Monthly (?) for exercise and diet. | Lengths of sessions not stated.  Individualised exercise prescription + 4 physiotherapist led gym session.  Dietician led individual and group sessions in weeks 4,8,12 and 16. This may represent 4 sessions or more than 4 sessions - not clear. | Yes |
|  | Financial Reward | Diet and exercise | Yes | Nutrition Edu. Fin. Incentives | Physician; Dietitian; Physiotherapist | No | Individual and Group | Face to Face | Health Care; Home | 8 | 4 | 18 | Weigh-ins biweekly for 2m; weekly for 6m; Monthly (?) for exercise and diet. | Lengths of sessions not stated.  Individualised exercise prescription + 4 physiotherapist led gym session.  Dietician led individual and group sessions in weeks 4,8,12 and 16. This may represent 4 sessions or more than 4 sessions - not clear. | Yes |
| Fisher 2011 | Diet only | Diet only | No | MR-F |  | No |  | Face to Face | Health Care | 6 | 6 | 0 |  |  | No |
|  | Diet + aerobic training | Diet and exercise | No | MR-F | Exercise physiologist | No | Group | Face to Face | Health Care; Community | 6 | 6 | 78 |  | 50 | No |
|  | Diet + resistance training | Diet and exercise | No | MR-F | Exercise physiologist | No | Group | Face to Face | Health Care; Community | 6 | 6 | 78 |  | 50 | No |
| Foley 2016 | Usual care (Control) | Control | No |  |  | No | Other | Print | Health Care |  |  |  |  |  | No |
|  | Weight loss intervention | Diet and exercise | Yes | Nutrition Edu. | Psychologist/ Counsellor; Health care professional (not specified); Other | No | Individual | Face to Face; Telephone; Internet; App; Print; SMS | Health Care; Home | 12 | 3 | 18 | Calls 1-4: weekly;  Calls 5-10: biweekly  Calls 11-18: monthly |  | No |
| Foreyt 1993 | Control (N/A) | N/A | N/A | N/A | N/A | N/A | N/A | N/A | N/A | N/A | N/A | N/A | N/A | N/A | N/A |
|  | Exercise only | Exercise only | Yes | MR-F; Help following programme end |  | Unclear | Group | Face to Face | Community; Home | 12 | 3 | 23 | 12 weekly sessions, then 3 fortnightly sessions then 8 monthly sessions. | 60 | Yes |
|  | Diet only | Diet only | Yes | Nutrition Edu.; Help following programme end | Dietitian | Unclear | Group | Face to Face | Community | 12 | 3 | 23 | 12 weekly sessions, then 3 fortnightly sessions then 8 monthly sessions. | 60 | Yes |
|  | Exercise plus diet | Diet and exercise | Yes | Nutrition Edu.; Help following programme end | Dietitian | Unclear | Group | Face to Face | Community; Home | 12 | 3 | 23 | 12 weekly sessions, then 3 fortnightly sessions then 8 monthly sessions. | 60 | Yes |
| Foster-Schubert 2012 | Control- usual care | Control | No |  |  | No | Other – no contact |  |  |  |  |  |  |  |  |
|  | Calorie reduced diet | Diet only | Yes | Nutrition Edu. | Dietitian | No | Individual and Group | Face to Face; Telephone; Internet | Community; Home | 12 | 6 | 38 | 2 + 24 weekly 0-24. Then 2 per month (12) during weeks 24 – 52 |  | Yes |
|  | Aerobic exercise (N/A) | N/A | N/A | N/A | N/A | N/A | N/A | N/A | N/A | N/A | N/A | N/A | N/A | N/A | N/A |
|  | Intervention - diet and exercise | Diet and exercise | Yes | Nutrition Edu. | Dietitian; Exercise physiologist; | No | Individual and Group | Face to Face; Telephone; Internet | Community; Home | 12 | 6 | 194 | 3 per week exercise. Plus 38 diet sessions. | 45 | Yes |
| Freitas 2017 | Weight loss program + Sham | Diet only | No | Nutrition Edu. | Psychologist/ Counsellor; Nutritionist; Physiotherapist | Unclear | Individual and Group | Face to Face | Community | 3 | 3 | 36 | Weekly therapy sessions; two weekly exercises sessions | 60 | Yes |
|  | Weight loss program + Exercise | Diet and exercise | No | Nutrition Edu. | Psychologist/ Counsellor; Nutritionist; Physiotherapist | Unclear | Individual and Group | Face to Face | Community | 3 | 3 | 36 | Weekly therapy sessions; two weekly exercises sessions | 60 | Yes |
| Fuller 2012 | Western diet group | Diet and exercise | No |  | Dietitian | No | Individual | Face to Face | Health Care; Community | 3 | 3 | 1 |  |  | No |
|  | Korean diet group | Diet and exercise | No | MR-F | Dietitian | No | Individual | Face to Face | Health Care; Community | 3 | 3 | 1 |  |  | No |
| Gold 2007 | Commercial programme eDiets | Diet and exercise | Yes | Nutrition Edu. |  |  | Other - online weight loss programme | Internet | Home | 0 | 12 | 1 |  | 1 x intro session.  Weekly online exercise journal  Online meetings, chat room, mentor system | Yes |
|  | Structured VTrim | Diet and exercise | Yes | Nutrition Edu. |  |  | Individual | Internet | Home | 12 | 6 | 39 |  | 6M of weekly 1 hour sessions = 26 (Weekly self-reported weight. Weekly homework and weekly feedback. Weekly feedback on journal entries.) 6 – 12m Biweekly meetings = 13 | Yes |
| Goodwin 2014 | Mailed-based intervention | Diet and exercise | No | Nutrition Edu. |  | No | Individual | Print | Community | 12 | 12 | 2 | 0, 12m |  | No |
|  | Individual lifestyle intervention | Diet and exercise | Yes | Nutrition Edu.; Help following programme end | Health Trainer | Unclear | Individual | Telephone; Print | Community | 12 | 6 | 19 | Weekly (0-1m); Biweekly (2-3m); Monthly (4-6m); every 2 months (7-12m); every 3 months (13-24m) | 30 – 60 | Yes |
| Hakala 1993 | Individual community-based counselling | Diet only | Yes | Nutrition Edu. |  | Unclear | Individual | Face to Face; Print | Health Care | 24 | 12 | 15 | Year 1: Monthly  Year 2: 4-monthly | 20 | No |
|  | Group in-patient rehabilitation | Diet and exercise | Yes | Nutrition Edu. | Physician; Nutritionist; Physiotherapist; Other AHPs | Unclear | Individual and Group | Face to Face | Residential; Health Care | 24 | 12 | 84 | 0-2 weeks: daily (inpatient 44 sessions over 2 weeks).  Weeks 2-8: weekly. Months 2-12: fortnightly.  Months: 12-24: monthly | 1 hour | No |
| Hanson 1976 | No treatment control condition (N/A) | N/A | N/A | N/A | N/A | N/A | N/A | N/A | N/A | N/A | N/A | N/A | N/A | N/A | N/A |
|  | Attention-placebo control condition | Control | No |  | Psychologist/ Counsellor | No |  |  |  |  |  | 0 |  |  | No |
|  | Conventional self-management condition | Diet only | No |  | Psychologist/ Counsellor | No | Group | Face to Face; Print |  | 2.5 | 2.5 | 10 | Weekly | 1 hour | No |
|  | Programmed text with low therapist-group contact | Diet only | No |  | Psychologist/ Counsellor | No | Group | Face to Face; Print |  | 2.5 | 2.5 | 3 | First meeting week 1, second meeting week 5, and 3 meeting week 10 |  | No |
|  | Programmed text with high therapist-group contact | Diet only | No |  | Psychologist/ Counsellor | No | Group | Face to Face; Print |  | 2.5 | 2.5 | 10 | Weekly | 1 hour | No |
| Harrigan 2016 | Usual Care Group | Diet and exercise | No |  |  | Unclear | Unclear | Unclear; | Health Care |  |  | 2 |  |  | No |
|  | Telephone Weight Loss Counseling | Diet and exercise | Yes |  | Dietitian | No | Individual | Telephone | Home | 6 | 6 | 11 | Once per week (month 1), then every two weeks (months 2 and 3), and once per month (months 4, 5, and 6) | 30 | No |
|  | In-Person Weight Loss Counseling | Diet and exercise | Yes |  | Dietitian | No | Individual | Face to Face | Health Care | 6 | 6 | 11 | Once per week (month 1), then every two weeks (months 2 and 3), and once per month (months 4, 5, and 6) | 30 | No |
| Hunt 2014 | Control, Wait-list | Control | No | Nutrition Edu. |  |  |  | Print |  |  |  |  |  |  |  |
|  | FFIT | Diet and exercise | Yes | Nutrition Edu.; Help following programme end | Personal Trainer | Yes | Group | Face to Face; Print | Community | 12 | 3 | 19 | Weekly for 12 weeks. 6 emails over 9 months. 1 reunion at 6 months. | 1.5 hours for 12 weekly sessions. Then 6 emails over 9m and 1 x reunion at 6m. | Yes |
| Huseinovic 2016 | Control Group | Diet only | No | Nutrition Edu. |  | No | Other - leaflet at baseline | Print; | Health Care | 0 | 0 |  |  |  | No |
|  | Diet behaviour modification Group | Diet and exercise | No | Nutrition Edu.; Help following programme end | Dietitian | Unclear | Individual | Face to Face; Telephone; Internet; Print; SMS | Health Care | 12 | 3 | 4 | Single face-to-face; biweekly SMS (3) followed by biweekly calls (3); monthly emails (9) | 1.5 hours single face-to-face session; Call length not stated. | Yes |
| Irwin 2003 | Control Group | Control | No |  |  |  |  |  |  | 0 | 0 | 0 | 0 | 0 | No |
|  | Exercise group | Exercise onl | Yes | Help following programme end | Exercise physiologist |  | Group | Face to Face | Community; Home | 12 | 3 | 36 | 3 times per week for the first 3 months and one per week the rest 9 months | 45 | No |
| Jakicic 2011 | Self Help Group | Exercise only | No |  |  | No | Other - provided manual and newsletters | Print; | Community | 18 |  | 1 |  | Monthly newsletter. | No |
|  | Moderate Physical Activity | Exercise only | Yes | Nutrition Edu. | Personal Trainer | Unclear | Individual and Group | Face to Face; Telephone; Print | Community | 18 | 6 | 156 | 4 x per week for 6 months, weekly contact between months 7 - 18 | Length of face-to-face sessions not reported; Telephone calls (< 10 mins) | No |
|  | High Physical Activity | Exercise only | Yes | Nutrition Edu. | Personal Trainer | Unclear | Individual and Group | Face to Face; Telephone; Print | Community | 18 | 6 | 156 | 4 x per week for 6 months, weekly contact between months 7 - 18 | Length of face-to-face sessions not reported; Telephone calls (< 10 mins)) | No |
| Jakicic 2015 | Standard behavior weight loss interventions group | Diet and exercise | Yes | Nutrition Edu. | Other | Unclear | Group | Face to Face; Print | Community | 18 | 6 | 52 | Sessions were conducted weekly for months 1–6 and every other week during months 7–18. | Group-based intervention sessions: approximately 45 min | Yes |
|  | ADOPT group | Diet and exercise | Yes | Nutrition Edu. | Other | Unclear | Group | Face to Face; Telephone; Print | Community | 18 | 6 | 86 | Sessions conducted weekly for months 1–6 and every other week during months 7–18. Biweekly phone contact; Weekly supervised exercise sessions for months 1–6; two 12-wk campaigns to promote physical activity. | Group-based intervention sessions: approximately 45 min; 10-min telephone call; Supervised physical activity sessions: A minimum of 30 min per session was encouraged. two 12-wk physical activity campaigns. | Yes |
|  | MAINTAIN Group (N/A) | N/A | N/A | N/A | N/A | N/A | N/A | N/A | N/A | N/A | N/A | N/A | N/A | N/A | N/A |
| Jebb 2011 | Standard care | Diet and exercise | No | Nutrition Edu. | GP | Yes | Individual | Face to Face | Health Care | 0 | 0 | 1 | Single session |  |  |
|  | Commercial programme | Diet and exercise | No | Nutrition Edu. | Health Trainer | Unclear | Group | Face to Face; Internet | Community | 12 | 12 | 52 | Weekly | 60 | Yes |
| Jebb 2017 | Usual care | Diet only |  | Nutrition Edu. | Nurse (General) | No | Individual | Face to Face; Print | Health Care | 3 | 3 | 6 to 12 | Weekly or biweekly |  | Yes |
|  | Low energy total diet replacement programme | Diet only | Yes | MR-F; Nutrition Edu.; Help following programme end | Health Trainer | Yes | Individual | Face to Face | Community | 6 | 6 | 15 | Weekly for first 12 weeks, then monthly |  | Yes |
| Jeffery 2003 | Standard behaviour therapy | Diet and exercise | Yes | Nutrition Edu.; Help following programme end | Health Trainer | Unclear | Group | Face to Face; Print | Community | 18 | 12 | 45 | Weekly from 0-6 months; biweekly from 6-12 months; monthly from 12-18 months |  | No |
|  | High physical activity | Diet and exercise | Yes | Nutrition Edu.; Fin. Incentives; Help following programme end | Personal Trainer | Unclear | Group | Face to Face; Print | Community | 18 | 12 | 90 | Weekly from 0-6 months; biweekly from 6-12 months; monthly from 12-18 months; Exercise coaches met with small groups before or after each group session. | Exercise coaches met with participants as an adjunct to regular standard behavioral treatment for 15–20 min in small groups. | Yes |
| Jolly 2011 | Minimal intervention comparator | Exercise only | No |  |  |  | Individual | Other | Community |  |  | 12 |  |  | No |
|  | Choice (N/A) | N/A | N/A | N/A | N/A | N/A | N/A | N/A | N/A | N/A | N/A | N/A | N/A | N/A | N/A |
|  | Pharmacy | Diet and exercise | No | Nutrition Edu.; Help following programme end | Other AHPs | No | Individual | Face to Face | Health Care | 3 | 3 | 12 | Weekly (although may not have taken place weekly in all cases.) | 1st session 30 mins, follow up sessions 15-20 mins | Yes |
|  | General practice | Diet and exercise | No | Nutrition Edu.; Help following programme end | Nurse (General); GP | No | Individual | Face to Face | Health Care | 3 | 3 | 12 | Weekly (although may not have taken place weekly in all cases.) | 1st session 30 mins, follow up sessions 15-20 mins | Yes |
|  | Weight Watchers | Diet and exercise | No | Nutrition Edu. | Other | Yes | Individual and Group | Face to Face | Community | 3 | 3 | 12 | Weekly | 1 hour | No |
|  | NHS Size Down | Diet and exercise | Yes | Nutrition Edu. | Health Trainer | Yes | Group | Face to Face | Community | 3 | 3 | 8 | Weekly × 6 weeks; drop-in at 9 and 12 weeks. | 2 hours for weeks 1-6; Duration of drop in sessions is unclear. | No |
|  | Rosemary Conley | Diet and exercise | No | Nutrition Edu. | Other | Yes | Individual and Group | Face to Face; Telephone; Internet | Community | 3 | 3 | 12 | Weekly | 1.5 hours | Yes |
|  | Slimming world | Diet and exercise | No | Nutrition Edu. | Other | Yes | Individual and Group | Face to Face; Telephone; Internet; Print | Community | 3 | 3 | 12 | Weekly | 1.5 hours; Duration and frequency of telephone support unclear. | Yes |
| Jones 1999 | Control Group | Control | No |  | Nurse (General) | Unclear | Individual | Face to Face | Community | 0 | 0 | 1 | Single session |  | No |
|  | Weight Loss Group | Diet only | No | Nutrition Edu. | Dietitian | Unclear | Individual and Group | Face to Face | Community | 30 | 3 | 8 + 4 to 9 | Initial session, second session at 2-4 weeks, two monthly group sessions up to 3 months, following which group sessions are every 3-6 months |  | Yes |
| Katula 2013 | Enhanced Usual Care Comparison Condition | Diet and exercise | No | Nutrition Edu. | Dietitian | Unclear | Individual | Face to Face; Print | Community | 24 |  | 2 | Both sessions during first 3 months |  | Yes |
|  | Lifestyle Weight-Loss Intervention | Diet and exercise | Yes | Nutrition Edu.; Help following programme end | Dietitian; Health Trainer | Yes | Individual and Group | Face to Face; Telephone | Community | 24 | 6 | 65 | Months 1-6:  Weekly group sessions plus “All participants received three personalized consultations with an RD (during Months 1, 3, and 6).”;  Months 7-24:  2 contacts per month, one group session and one phone contact |  | Yes |
| Keogh 2014 | Intermittent dieting | Diet only | No | Nutrition Edu.; Inter. Fasting | Dietitian | No | Group | Face to Face |  | 2 | 2 | 5 | Every second week | The first visit will take approx. an hour; follow up visits will be shorter | No |
|  | Continuous dieting | Diet only | No | Nutrition Edu. | Dietitian | No | Group | Face to Face |  | 2 | 2 | 5 | Every second week | The first visit will take approx. an hour; follow up visits will be shorter | No |
| Keranen 2009 | Short-term counselling | Diet only | No | Nutrition Edu. | Nurse (Specialist) |  | Individual | Face to Face | Health Care | 1 | 1 | 2 | Fortnightly |  | Yes |
|  | Intensive counselling | Diet only | No | Nutrition Edu.; Help following programme end | Dietitian | Yes | Individual and Group | Face to Face | Health Care | 4.6 | 4.6 | 10 | Fortnightly |  | Yes |
| King 1989 | Control (N/A) | N/A | N/A | N/A | N/A | N/A | N/A | N/A | N/A | N/A | N/A | N/A | N/A | N/A | N/A |
|  | Exercise only | Exercise only |  |  |  | Unclear | Unclear | Face to Face; Print | Community |  | 12 |  |  |  | Yes |
|  | Diet only | Diet only |  | Nutrition Edu. | Nutritionist | Unclear | Individual and Group | Face to Face | Community |  | 12 |  |  |  | Yes |
| Knauper 2018 | Standard DPP | Diet and exercise | Yes | Nutrition Edu. | Health Trainer | Yes | Group | Face to Face | Community | 12 | 3 | 22 | 12 weekly core sessions, 4 transitional sessions over 3 months, and 6 monthly support sessions | 1 hour |  |
|  | Enhanced DPP | Diet and exercise | Yes | Nutrition Edu.; Help following programme end | Health Trainer | Yes | Group | Face to Face; Print | Community | 12 | 3 | 22 | 12 weekly core sessions, 4 transitional sessions over 3 months, and 6 monthly support sessions | 1 hour | Yes |
| Kumanyika 2012 | Basic programme | Diet and exercise | No | Nutrition Edu.; Help following programme end | Nurse (General); Physician; Other AHPs | Yes | Individual | Face to Face; Print | Health Care; Home | 12 | 12 | 3 | PCP every 4 months. 12 printed session in year 1. 2 printed sessions in year 2. | 12.5 | Yes |
|  | Basic plus programme | Diet and exercise | Yes | Nutrition Edu.; Help following programme end | Nurse (General); Physician; Other AHPs; Health Trainer | Yes | Individual | Face to Face; Print | Health Care; Home | 12 | 12 | 15 | PCP every 4 months. LC monthly year 1, every other month year 2. 12 printed session in year 1. | 12.5 | Yes |
| Leahey 2014 | SURI alone | Diet and exercise | No |  |  |  | Other – internet | Internet; Print |  | 3 | 3 | 0 |  |  | No |
|  | SURI plus Internet behavioral weight loss program | Diet and exercise | No | Nutrition Edu. |  |  | Other - internet | Face to Face; Internet; Print | Home | 3 | 3 | 12 | Weekly | 10 – 15 | No |
|  | SURI plus Internet behavioral weight loss program plus optional group sessions | Diet and exercise | No | Nutrition Edu. | Health Trainer | No | Group | Face to Face; Internet; Print; | Community; Home | 3 | 3 | 12 | Weekly | 10 – 15 | No |
| Leahey 2015 | SURI1 Internet behavioral weight loss | Diet and exercise | No | Nutrition Edu. |  | No | Individual and Group | Face to Face; Internet; Print; Video | Health Care | 3 | 3 | 13 | Weekly |  | Yes |
|  | SURI1 Internet behavioral weight loss1incentives | Diet and exercise | No | Nutrition Edu.; Fin. Incentives |  | No | Individual and Group | Face to Face; Internet; Print; Video | Health Care | 3 | 3 | 13 | Weekly |  | Yes |
|  | SURI1 Internet behavioral weight loss 1 group option | Diet and exercise | No | Nutrition Edu. | Dietitian; Exercise physiologist | No | Individual and Group | Face to Face; Internet; Print; Video | Health Care | 3 | 3 | 26 | Weekly group sessions; Weekly videos |  | Yes |
| Lejeune 2003 | Diet | Diet only | Yes | MR-P; MR-F; Nutrition Edu. |  | Unclear | Unclear | Unclear | Community | 13 | 13 |  |  |  | No |
|  | Diet plus exercise | Diet and exercise | Yes | MR-P; MR-F; Nutrition Edu.; Help following programme end | Personal Trainer | Unclear | Unclear | Face to Face; Unclear | Community; Home | 13 | 13 | 159 | 4 x per week | Exercise training programme: 4 x 1 hour/week (They trained four times 1 h/week, three times at the laboratory under the supervision of a professional trainer and once at home.) | No |
| Ley 2004 | Control diet | Control | No |  |  |  |  |  |  |  |  |  |  |  |  |
|  | Reduced-fat | Diet only |  | Nutrition Edu. |  |  |  | Face to Face |  | 12 | 12 | 12 |  |  |  |
| Li 2016 | Usual care group | Control | Yes |  | Dietitian; Other | Yes |  | Print | Inpatient | 12 | 1 | 0 |  |  | Yes |
|  | Diet group | Diet only | Yes | Help following programme end | Dietitian; Other | Yes | Group | Face to Face; Telephone; Internet; Print | Inpatient | 12 | 1 | 36 | Six weekly sessions (Month 1); Monthly sessions (Months 1 - 12) |  | Yes |
|  | 50g-oats group | Diet only | Yes | Help following programme end | Dietitian; Other | Yes | Group | Face to Face; Telephone; Internet; Print | Inpatient | 12 | 1 | 36 | Six weekly sessions (Month 1); Monthly sessions (Months 1 - 12) |  | Yes |
|  | 100g-oats group | Diet only | Yes | Help following programme end | Dietitian; Other | Yes | Group | Face to Face; Telephone; Internet; Print | Inpatient | 12 | 1 | 36 | Six weekly sessions (Month 1); Monthly sessions (Months 1 - 12) |  | Yes |
| Li 2005 | Individualized diet plan | Diet only | Yes | Nutrition Edu.; Help following programme end | Dietitian | Unclear | Individual | Face to Face | Community | 12 | 2 | 15 | Months 1-2: Every 2 weeks; Months 2-12: Monthly |  | Yes |
|  | Soy-based meal replacement | Diet only | Yes | MR-P; MR-F; Nutrition Edu.; Help following programme end | Dietitian | Unclear | Individual | Face to Face; Other | Community | 12 | 3 | 15 | Months 1-2: Every 2 weeks; Months 2-12: Monthly |  | Yes |
| Lindstrom 2003 | Control | Control | No | Nutrition Edu. | Nurse (General); Physician; Nutritionist | Unclear | Other - group or individual | Face to Face; Print | Health Care |  |  | 1 |  | 30 mins to 1 hour | No |
|  | Intervention | Diet and exercise | Yes | MR-P; MR-F; Nutrition Edu.; Help following programme end | Nurse (General); Physician; Nutritionist; Physiotherapist | Unclear | Individual and Group | Face to Face; Telephone; Print | Health Care; Community | 48 | 12 | 19 | 7 sessions in first year then every 3 months | 30 mins to 1 hour | Yes |
| Liss 2016 | Standard care arm | Diet and exercise | No | Nutrition Edu. | Health care professional (not specified); Other | No | Individual | Face to Face; Print | Health Care | 12 |  | 3 | Every 6m | "brief" | Yes |
|  | Standard care plus group-based lifestyle intervention | Diet and exercise | Yes | Nutrition Edu.; Help following programme end | Health Trainer | Yes | Individual and Group | Face to Face; Print | Health Care; Community | 12 | 6 | 39 | Weekly for 6m; biweekly for next 6m | 3 brief; 60-to-90-minute intervention sessions | Yes |
| Little 2016 | Control, Nurse follow-up | Diet only | No | Nutrition Edu. | Nurse (General) | No | Other | Internet | Home |  |  |  | Data collection only at 6 and 12 months |  | No |
|  | Web-based support with minimal support (Remote) | Diet and exercise | No | Nutrition Edu.; Help following programme end | Nurse (General) | Unclear | Individual | Telephone; Internet; Other | Home | 6 | 6 | 29 | 24 web-based sessions designed to be used over 6 months. Three scheduled phone or email contacts and up to two optional phone or email contacts in the first 6 months |  | Yes |
|  | Web-based + nurse support (face to face) | Diet and exercise | No | Nutrition Edu.; Help following programme end | Nurse (General) | Unclear | Individual | Face to Face; Telephone; Internet; Other | Health Care; Home | 6 | 6 | 31 | 24 web-based sessions designed to be used over 6 months. Three scheduled face-to-face appointments in the first 3 months, and then up to four more appointments during a further 3 months if needed. |  | Yes |
| Lowe 2018 | Behavior therapy | Diet and exercise |  | Nutrition Edu.; Help following programme end |  | No | Group | Face to Face; Telephone |  | 12 | 12 | 39 | Weekly for 6m, then biweekly for 6m | 75 | No |
|  | Behavior therapy plus meal replacements | Diet and exercise |  | MR-P; Nutrition Edu.; Help following programme end |  | No | Group | Face to Face; Telephone |  | 12 | 12 | 39 | Weekly for 6 m, then biweekly for 6m | 75 | No |
|  | Home food environment | Diet and exercise |  | Nutrition Edu.; Help following programme end | Other | No | Group | Face to Face; Telephone |  | 12 | 12 | 39 | Weekly for 6 m, then biweekly for 6m | 75 | No |
| Ma 2015 | Control, Enhanced usual care | Control | No |  | Other AHPs | No | Individual | Print | Health Care |  |  | 0 |  |  | No |
|  | Diet and counselling | Diet and exercise | Yes | Nutrition Edu.; Help following programme end | Dietitian; Other AHPs; Personal Trainer | Yes | Individual and Group | Face to Face; Telephone | Health Care | 12 | 6 | 18 | Intensive stage: 13 weekly small group sessions over 4 months; Transitional stage: 1 individual counselling session in month 5 and another in month 6; Extended stage: 3 bi-monthly or more phone (participants can initiate contact with interventionists at any point and participants with weight gains of 1.4 to 2.2kg will be telephoned on a biweekly basis until they return to a stable, lower weight). | Group sessions 90-120 mins, transitional phase contacts 30-60 mins; extended sessions variable | Yes |
| Manning 1994 | Clinic visit | Diet only | Yes | Nutrition Edu. | Dietitian | No | Individual | Face to Face | Health Care | 12 | 6 | 7 | 6 weekly intervals for the first 6 months and then 2 monthly for the remainder of the year. |  | Yes |
|  | Behavioural | Diet only | Yes | Nutrition Edu. | Psychologist/ Counsellor; Dietitian; Physiotherapist | No | Group | Face to Face | Health Care | 12 | 3 | 10 | Fortnightly intervals initially for 3 months and then at 2 monthly intervals for the remainder of the year. |  | Yes |
|  | Home visits | Diet only | Yes | Nutrition Edu. | Dietitian | No | Individual | Face to Face | Health Care; Home | 12 | 6 | 7 | 6 weekly intervals for the first 6 months and then 2 monthly for the remainder of the year. |  | Yes |
|  | Dexfenfluramine (N/A) | N/A | N/A | N/A | N/A | N/A | N/A | N/A | N/A | N/A | N/A | N/A | N/A | N/A | N/A |
|  | Routine usual care (N/A) | N/A | N/A | N/A | N/A | N/A | N/A | N/A | N/A | N/A | N/A | N/A | N/A | N/A | N/A |
| Manzoni 2016 | Control, Standard behavioral inpatient program | Diet and exercise | No | Nutrition Edu. | Health care professional (not specified) | Unclear | Individual and Group | Face to Face; Telephone; Internet | Inpatient | 1.4 | 1.4 | 6 | Weekly nutritional groups held by dietitians |  | Yes |
|  | Cognitive–behavioral therapy | Diet and exercise | No | Nutrition Edu.; Help following programme end | Psychologist/ Counsellor; Dietitian; Health care professional (not specified) | Yes | Individual and Group | Face to Face; Telephone; Internet | Inpatient | 1.4 | 1.4 | 21 | Weekly and biweekly |  | Yes |
|  | CBT + Virtual reality | Diet and exercise | No | Nutrition Edu.; Help following programme end | Psychologist/ Counsellor; Dietitian; Health care professional (not specified) | Yes | Individual and Group | Face to Face; Telephone; Internet; Other | Inpatient | 1.4 | 1.4 | 36 | Weekly and biweekly | 60 | Yes |
| Marniemi 1990 | Control group | Control | No |  |  | No |  |  |  |  |  | 0 |  |  | No |
|  | Lactovegetarian weight reduction group | Diet only | No | Nutrition Edu. | Dietitian | No | Group | Face to Face |  | 12 | 2.5 | 15 | 10 weekly for 2.5 months and 5 throughout the year |  | No |
|  | Mixed diet weight reduction | Diet only | No | Nutrition Edu. | Dietitian | No | Group | Face to Face; |  | 12 | 2.5 | 15 | 10 weekly for 2.5 months and 5 throughout the year |  | No |
| Martin 2008 | Control, Standard Care | Control | No |  | Physician | Yes |  |  |  |  |  | 0 |  |  | Yes |
|  | Tailored physician/lifestyle counselling | Diet and exercise | No | Nutrition Edu.; Help following programme end | Physician | Yes | Individual | Face to Face; Print | Health Care | 6 | 6 | 6 | Monthly | 15 | Yes |
| Mefferd 2007 | Control | Control | No |  |  | Unclear | Unclear |  |  |  |  |  |  |  |  |
|  | Intervention | Diet and exercise | Yes | Nutrition Edu.; Help following programme end |  | Unclear | Individual and Group | Face to Face; Telephone | Health Care | 4 | 12 | 78 | 16 weeks of weekly closed group sessions followed by once-monthly sessions and then monthly sessions for an additional 6 months; Telephone contact: twice weekly during initial two weeks; weekly thereafter |  | Yes |
| Mengham 1999 | Control |  | No | Nutrition Edu. | Dietitian | Unclear | Unclear | Face to Face | Health Care | 12 | 12 | 3 | Six-monthly | 15 | Yes |
|  | Intervention |  | No | Nutrition Edu.; Help following programme end | Dietitian | Unclear | Individual and Group | Face to Face | Health Care | 6 | 12 | 16 | Fortnightly up to 6 months plus 6 monthly sessions | 15 mins standard care sessions; Patients in the intervention group typically received input from the dietitian amounting to 3hrs over the twelve months of the study. | Yes |
| Messier 2013 | Exercise only | Exercise only | Yes | Help following programme end | Personal Trainer | Yes | Unclear | Face to Face; Telephone | Community | 18 | 6 | 78 | 3 days per week | 60 | Yes |
|  | Diet-induced weight loss only | Diet only | Yes | MR-P; Nutrition Edu.; Help following programme end | Nutritionist | Yes | Individual and Group | Face to Face; Print | Community | 18 | 6 | 30 | 1-6 Months: individual session and 3 group sessions per month;  7-18 Months: biweekly group sessions and an individual session every 2 months |  | Yes |
|  | Diet-induced weight loss plus exercise | Diet and exercise | Yes | MR-P; Nutrition Edu.; Help following programme end | Nutritionist | Yes | Individual and Group | Face to Face; Telephone; Print | Community | 18 | 6 | 108 | 1-6 Months: individual session and 3 group sessions per month; 7-18 Months: biweekly group sessions and an individual session every 2 months plus 3 days/week (exercise) | 60 mins exercise; diet group sessions not reported | Yes |
| Miller 2002 | Control Group (Monitoring) | Control | No |  |  |  |  |  |  |  |  |  |  |  |  |
|  | Lifestyle Intervention | Diet and exercise | No | MR-P; Nutrition Edu.; Help following programme end | Personal Trainer |  | Group | Face to Face | Health Care; Community | 2 | 2 |  |  | 30 to 45 min for the exercise sessions | No |
| Mitsui 2008 | Control | Control | No |  |  | Unclear | Unclear |  | Health Care |  |  | 0 |  |  | No |
|  | Intervention | Diet and exercise | Yes | Nutrition Edu. | Dietitian | Unclear | Individual and Group | Face to Face | Health Care | 3 | 12 | 25 | Weekly: 0-12; Every other week: 13-26; Monthly 26-52 weeks | Exercise training: 40 minutes; Individual counselling sessions: not reported | Yes |
| Molenaar 2010 | Nutritional counselling group (diet D, group) | Diet only | No | Nutrition Edu. | Dietitian | No | Individual | Face to Face | Health Care | 12 | 6 | 8 | 7 sessions during the 6 months and 1 follow-up session at 12 months | The initial sessions lasted 40 mins and the rest 20 mins. | Yes |
|  | Nutritional plus exercise counselling group (diet + exercise (D + E) group) | Diet and exercise | No | Nutrition Edu. | Dietitian; Physiotherapist | No | Individual | Face to Face | Health Care | 12 | 6 | 15 | Similar to D group PLUS six exercise counselling sessions during the first 6 months and a follow-up session at 12 months. | The duration of the diet sessions were identical to D groups plus exercise sessions: The duration of the initial counselling session was assumed to be 45 to 60 minutes and later sessions 30 minutes. | Yes |
| Moreno 2014 | Low-calorie diet | Diet and exercise | Yes | Nutrition Edu. | Physician; Dietitian; Other | No | Individual and Group | Face to Face; Telephone | Health Care | 12 | 12 | 9 | LC diet. Group meetings took place at 0.5, 2, 4, 6, 8, 10, and 12 months |  | Yes |
|  | Very low-calorie-ketogenic diet | Diet and exercise | Yes | MR-P; Nutrition Edu. | Physician; Dietitian; Other | No | Other | Face to Face; Telephone | Health Care | 12 | 2 | 9 | VLCK diet up to 2m (45-60 days). Meetings same as LCD group |  | Yes |
| Morgan 2010 | Control (Information and self-help) | Control | No | Nutrition Edu. | Other | Unclear | Group | Face to Face; Print | Community |  |  | 1 | Once | 60 | No |
|  | SHED-IT (Internet) group | Diet and exercise | Yes | Nutrition Edu. | Other | Unclear | Individual and Group | Face to Face; Internet; Print | Community; Home | 3 | 3 | 8 | Submit online daily eating and exercise diaries for the first 4 weeks, for 2 weeks in the second month and for 1 week in the third month. 7 x feedback | 1st session face to face group- 75 mins. The rest internet.  7 feedback sessions. Submit online daily eating and exercise diaries for the first 4 weeks, for 2 weeks in the second month and for 1 week in the third month. 28 + 14 + 7 = 49 | Yes |
| Muggia 2014 | Standard care group | Diet only | No | Nutrition Edu. |  | No | Individual | Print | Home | 12 | 6 | 7 | Control meetings every 3 months during the first year and every 6 months during the second year | 30 | No |
|  | Brief CBT group | Diet only | No | Nutrition Edu. |  | No | Group | Face to Face; Print | Health Care | 12 | 6 | 14 | 7 treatment sessions in a monthly basis. Then control meetings every 3 months during the first year and every 6 months during the second year | 90 | No |
| Ng 2015 | Control group | Control | No | Nutrition Edu. | Physician | No | Individual | Face to Face | Health Care | 6 |  | 2 | Single sessions at baseline and at 6 months |  |  |
|  | Lifestyle modification program | Diet and exercise | Yes | Nutrition Edu.; Help following programme end | Dietitian | No | Individual | Face to Face | Health Care | 12 | 4 | 24 | Weekly (Months 1-4); Monthly (Months 5-12) | Encouraged to see an exercise instructor at least once during the program and perform 30 min of aerobic exercise two to three times a week. |  |
| Nicklas 2009 | Calorie restriction (CR) Only | Diet only | No | MR-P; Nutrition Edu.; Help following programme end | Dietitian; | Unclear | Individual | Face to Face; Other | Community | 4.6 | 17 | 1 |  |  | Yes |
|  | CR + Moderate-Intensity | Diet and exercise | No | MR-P; Nutrition Edu.; Help following programme end | Dietitian; Exercise physiologist | Unclear | Unclear | Face to Face; Other | Community | 4.6 | 17 | 60 | 3 sessions per week | 20–25 min the first week to 55 min by the end of the sixth week and thereafter. | Yes |
|  | CR + Vigorous-Intensity | Diet and exercise | No | MR-P; Nutrition Edu.; Help following programme end | Dietitian; Exercise physiologist | Unclear | Unclear | Face to Face; Other | Community | 4.6 | 17 | 60 | 3 sessions per week | 10–15 min the first week to 30 min by the end of the sixth week and thereafter | Yes |
| Nilsen 2011 | Control, Individual Physician Group | Diet and exercise | No | Nutrition Edu. | Physician |  | Individual | Face to Face | Health Care | 18 | 18 | 3 | 6 monthly |  |  |
|  | Individual Plus Interdisciplinary Group | Diet and exercise | Yes | Nutrition Edu.; Help following programme end | Nurse (General); Physician; Dietitian; Physiotherapist; Other |  | Individual and Group | Face to Face | Health Care; Community | 18 | 6 | 11 | Weekly (Weeks 5 to 10. Other sessions at week 3, 16, 20, 26 52 and 78 | 7 sessions of 5 hours; 1 x individual session 30 mins; 3 x physician consultation (30 mins? [3 x exercise test] | Yes |
| Oldroyd 2006 | Control group | Control | No |  |  |  |  |  |  |  |  |  |  |  |  |
|  | Intervention group | Diet and exercise | No | Nutrition Edu. | Dietitian; Physiotherapist | No | Individual | Face to Face; Print | Community | 24 | 6 | 12 | In the first 6 months there were three such appointments at two weekly intervals, followed by three at monthly intervals. There was one after 9 months and five at two monthly intervals between 12 and 24 months. | 15 – 20 | Yes |
| Parikh 2010 | Control | Control |  |  |  |  | Unclear |  | Health Care |  |  |  |  |  |  |
|  | Intervention | Diet and exercise | No | Nutrition Edu. |  | Yes | Group | Face to Face; Print | Community | 3 | 3 | 8 | 8 sessions over 10 weeks | 1.5 hours | No |
| Pedersen 2013 | Aerobic interval training | Exercise only | Yes | Nutrition Edu.; Help following programme end | Physiotherapist | Unclear | Group | Face to Face | Community | 12 | 3 | 116 | Three times a week for 12 weeks; twice weekly for 40 weeks. | 38 |  |
|  | Low energy diet | Diet and exercise | Yes | MR-P; Nutrition Edu.; Help following programme end | Dietitian | Unclear | Individual and Group | Face to Face | Community | 12 | 3 | 96 | Fortnightly dietitian sessions (Weeks 1-12); Monthly (Weeks 12 – 52); Twice weekly exercise sessions for 40 weeks. | Exercise sessions: 38 mins; Dietitian sessions not reported |  |
| Perri 1986 | Behavior therapy | Diet and exercise | No | Nutrition Edu.; Fin. Incentives | Psychologist/ Counsellor | Yes | Group | Face to Face; Print | Community | 4.6 | 4.6 | 20 | Weeks 1-20: Weekly | Group therapy sessions: 2 hours | No |
|  | Behavior therapy plus maintenance | Diet and exercise | Yes | Nutrition Edu.; Fin. Incentives; Help following programme end | Nurse (General); Psychologist/ Counsellor | Yes | Individual and Group | Face to Face; Telephone; Print | Community | 16.6 | 16.6 | 98 | Weekly | Group therapy sessions: 2 hours Client-therapist telephone contacts were scheduled to occur weekly during the 12 months following treatment. Buddy groups were encouraged to meet twice a month during the year following treatment. | Yes |
|  | Behavior therapy plus aerobic exercise | Diet and exercise | No | Nutrition Edu.; Fin. Incentives | Nurse (General); Psychologist/ Counsellor | Yes | Group | Face to Face Print | Community | 4.6 | 4.6 | 32 | Weeks 1-20: Weekly; Exercise program: weekly | Group therapy sessions: 2 hours Exercise program goals: Initial levels of exercise were set at a minimum of 32 min per week (8 min per day of stationary cycling, 4 days per week). Weekly increases of 4 rain were scheduled over 12 consecutive weeks to a target level of 80 min per week (20 rain per day, 4 days per week). | Yes |
|  | Behavior therapy behavior therapy plus aerobic exercise plus maintenance | Diet and exercise | Yes | Nutrition Edu.; Fin. Incentives; Help following programme end | Nurse (General); Psychologist/ Counsellor | Yes | Individual and Group | Face to Face; Telephone; Print | Community | 16.6 | 16.6 | 110 | Weekly | Group therapy sessions: 2 hours Exercise program goals: Initial levels of exercise were set at a minimum of 32 min per week (8 min per day of stationary cycling, 4 days per week). Weekly increases of 4 rain were scheduled over 12 consecutive weeks to a target level of 80 min per week (20 rain per day, 4 days per week). | Yes |
| Perri 1997 | WL + Home-based exercise | Diet and exercise | Yes | Nutrition Edu.; Help following programme end | Psychologist/ Counsellor | Yes | Group | Face to Face; Print | Community | 12 | 12 | 39 | WL sessions (26 weekly, 13 biweekly) | 2-hour WL sessions. 30 min of exercise on 5 days per week | No |
|  | WL + Group-based exercise | Diet and exercise | Yes | Nutrition Edu.; Help following programme end | Psychologist/ Counsellor; Health Trainer | Yes | Group | Face to Face | Community | 12 | 12 | 169 | WL sessions (26 weekly, 13 biweekly); Exercise sessions: 3 x weekly (0-26 weeks); 2 x weekly sessions (27 -52 week) | 2-hour WL sessions; 30 min of exercise on 5 days per week | No |
| Perri 2001 | Control, Standard Behavioural Therapy (BT) | Diet and exercise | No | Nutrition Edu. | Psychologist/ Counsellor |  | Group | Face to Face | Community | 5 | 5 | 20 | Weekly | 2 hours |  |
|  | BT + Relapse prevention training | Diet and exercise | Yes | Nutrition Edu.; Help following programme end | Psychologist/ Counsellor |  | Group | Face to Face; Print | Community | 17 | 5 | 46 | Weekly and biweekly | 2 hours | Yes |
|  | BT + problem-solving therapy | Diet and exercise | Yes | Help following programme end | Psychologist/ Counsellor |  | Group | Face to Face; Print | Community | 17 | 5 | 46 | Weekly and biweekly | 2 hours | Yes |
| Pettman 2009 | Control | Control | No | Nutrition Edu. |  | No | Individual | Print |  |  |  |  |  |  | No |
|  | Intervention B - Passive follow-up | Diet and exercise | No | Nutrition Edu. | Health Trainer | Unclear | Group | Face to Face; Telephone; Other | Community | 4 | 12 | 32 | Weekly group session and exercise session | 2 hour group sessions: 1 hour exercise session. | No |
|  | Intervention A - Active follow-up | Diet and exercise | Yes | Nutrition Edu.; Help following programme end | Health Trainer | Unclear | Group | Face to Face; Telephone; Other | Community | 4 | 12 | 40 | Weekly group session and exercise session | 2 hour group sessions;  1 hour exercise session. | No |
| Poelman 2015 | Control Condition | Control | No |  |  |  |  |  |  |  |  | 0 |  |  | No |
|  | Intervention condition | Diet only | No | Nutrition Edu. | Dietitian; Health care professional (not specified) | No | Individual and Group | Face to Face; Internet | Community | 12 | 3 | 3 | Biweekly | 3 hour cooking class, 8-minute video | No |
| Promrat 2010 | Control | Diet and exercise | No | Nutrition Edu. | Nutritionist; Health Trainer | Unclear | Group | Face to Face | Health Care | 12 | 12 | 4 | Once every 12 weeks. |  |  |
|  | Lifestyle Intervention | Diet and exercise | Yes | Nutrition Edu.; Help following programme end | Nutritionist; Health Trainer | Unclear | Individual and Group | Face to Face | Health Care | 12 | 6 | 36 | Months 1-6: weekly; Months 7-12: biweekly |  | Yes |
| Provencher 2009 | Control group | Control | No |  |  |  |  |  |  |  |  |  |  |  |  |
|  | Social support | Diet only | No | Nutrition Edu. | Psychologist/ Counsellor; Dietitian | Yes | Group | Face to Face | Community | 4 | 4 | 14 | Weekly | 2 hours | Yes |
|  | Health-At-Every-Size | Diet and exercise | No | Nutrition Edu. | Psychologist/ Counsellor; Dietitian | Yes | Group | Face to Face; Print | Community | 4 | 4 | 14 | Weekly | "13 three-hour evening sessions and 1 intensive-day session of 6 hours)." | Yes |
| Rock 2015 | Control | Diet and exercise | Yes | Nutrition Edu. | Other | Unclear | Individual | Face to Face; Telephone; Internet | Community | 6 | 12 | 14 | 2 individual sessions (baseline and 6 months); monthly telephone calls and/or e-mails |  | Yes |
|  | Intervention | Diet and exercise | Yes | Nutrition Edu.; Help following programme end | Other | Unclear | Individual and Group | Face to Face; Telephone; Internet | Community | 6 | 12 | 73 | Group sessions: weekly for 4 months; biweekly for two months; monthly for 6 months; Newsletters: quarterly from 6 to 24 months. | 1 hour group sessions; Group sessions were reinforced by brief (10- to 15-minute) personalized guidance delivered by telephone and/or e-mail. | Yes |
| Rolls 2005 | Comparison-control | Control | No |  |  | No |  |  |  |  |  |  |  |  |  |
|  | Two snacks | Diet and exercise | No | MR-P; Nutrition Edu.; Help following programme end | Dietitian | No | Individual | Face to Face |  | 12 | 6 | 24 | Weekly from 1 to 3 months, fortnightly 4 to 6 months, and monthly from 7 to 12 months | 15 – 30 | No |
|  | One soup | Diet and exercise | No | MR-P; Nutrition Edu.; Help following programme end | Dietitian | No | Individual | Face to Face |  | 12 | 6 | 24 | Weekly from 1 to 3 months, fortnightly 4 to 6 months, and monthly from 7 to 12 months | 15 – 30 | No |
|  | Two soups | Diet and exercise | No | MR-P; Nutrition Edu.; Help following programme end | Dietitian | No | Individual | Face to Face |  | 12 | 6 | 24 | Weekly from 1 to 3 months, fortnightly 4 to 6 months, and monthly from 7 to 12 months | 15 - 30 | No |
| Rolls 2017 | Standard advice | Diet and exercise | Yes | Nutrition Edu. | Dietitian; Other | Yes | Individual | Face to Face; Print | Community | 12 | 1 | 19 | Weekly in month 1 and biweekly in months 2–6, and 1-hour sessions were scheduled monthly in months 7–12 | Thirty-min weekly and biweekly sessions; 1-hour monthly sessions. | Yes |
|  | Pre-portioned foods group | Diet and exercise | Yes | MR-P; Nutrition Edu.; Help following programme end | Dietitian; Other | Yes | Individual | Face to Face; Print | Community | 12 | 1 | 19 | Weekly in month 1 and biweekly in months 2–6, and 1-hour sessions were scheduled monthly in months 7–12 | Thirty-min weekly and biweekly sessions; 1-hour monthly sessions. | Yes |
|  | Portion selection group | Diet and exercise | Yes | Nutrition Edu.; Help following programme end | Dietitian; Other | Yes | Individual | Face to Face; Print | Community | 12 | 1 | 19 | Weekly in month 1 and biweekly in months 2–6, and 1-hour sessions were scheduled monthly in months 7–12 | Thirty-min weekly and biweekly sessions; 1-hour monthly sessions. | Yes |
| Rosas 2015 | Usual care |  | No |  | GP | No | Individual | Face to Face | Health Care |  |  |  |  |  | No |
|  | Case-management intervention |  | Yes | Nutrition Edu.; Help following programme end | Health Trainer | No | Individual and Group | Face to Face | Health Care | 24 | 6 | 20 | 16 sessions from 0-12 months. 4 sessions from 12-24 months. | Group sessions last 2 hours, individual sessions last 30 minutes. | Yes |
|  | Case-management + Community health worker intervention |  | Yes | Nutrition Edu.; Help following programme end | Health Trainer | No | Individual and Group | Face to Face | Health Care; Community;Home | 24 | 6 | 27 | Same as CM group, with additional 5 home visits from 0-12 months and 2 home visits from 12-24 months. | Same as CM group. The length of the additional CHW home visits is not clear. | Yes |
| Ross 2012 | Control condition | Control | No | Nutrition Edu. | Physician | No | Unclear | Face to Face | Health Care |  |  |  | Usual schedule (typically once a year). |  | Yes |
|  | Behavioral intervention group | Diet and exercise | No | Nutrition Edu.; Help following programme end | Health Trainer | Yes | Individual and Group | Face to Face |  | 24 | 6 | 33 | First 6m: 8 sessions in first 6 weeks, then every 2 weeks. Months 7-24, monthly sessions. | (0–6 months, 15 sessions, 15 hours); Months 7–12 (6 sessions, 3–6 hours); Months 13–24 (12 sessions, 6–12 hours) | Yes |
| Samaras 1997 | Control | Control | No |  |  |  |  |  |  |  |  |  |  |  |  |
|  | Intervention | Exercise only | No | Help following programme end | Nurse (General); Physician; Dietitian; Exercise physiologist; Other | Yes | Group | Face to Face; Print; Video | Community | 6 | 6 | 6 | Monthly | 1 hour | Yes |
| Santanasto 2011 | Physical Activity plus Successful Ageing | Diet only | Yes | Nutrition Edu.; Help following programme end |  | Unclear | Individual and Group | Face to Face | Community; Home | 12 | 6 | 68. | Monthly SA session; Exercise sessions: 3 x sessions/week (Weeks 1 – 8); two sessions/week (weeks 9–24); optional exercise session at the center once per week (weeks 25–52) | 60 min exercise sessions | No |
|  | Physical Activity plus Weight Loss | Diet and exercise | Yes | Nutrition Edu.; Help following programme end | Nutritionist | Unclear | Individual and Group | Face to Face | Community; Home | 12 | 6 | 87 | Nutrition sessions: 24 weekly, 2 bimonthly, and 5 monthly; Exercise sessions: 3 x sessions/week (Weeks 1 – 8); two sessions/week (weeks 9–24); optional exercise session at the center once per week (weeks 25–52) | 60 minutes exercise sessions | No |
| Sattin 2016 | Health Education intervention | Control | No | Nutrition Edu. | Health care professional (not specified) | Yes | Group | Face to Face | Community | 9 | 3 | 18 | Weekly for the first 12 weeks and monthly for the remaining 6m |  | Yes |
|  | Fit body and soul intervention | Diet and exercise | No | Nutrition Edu.; Help following programme end | Health care professional (not specified) | Yes | Group | Face to Face | Community | 9 | 3 | 18 | Weekly for the first 12 weeks and monthly for the remaining 6m |  | Yes |
| Schubel 2016 | Control group | Control | Yes | Nutrition Edu. | Dietitian; Nutritionist | Yes | Individual | Face to Face; Telephone | Community | 11.5 | 3 | 8 | Biweekly phone calls (Week 1-12); Two single sessions at the beginning and end of the Intervention phase | "The number of personal contacts and counseling sessions was the same for all study participants overall, but individuals in the ICR and CCR arms received longer and more comprehensive counseling sessions with personalized dietary plans, specific for the ICR or CCR regimens." | No |
|  | Continuous Calorie Restriction | Diet only | Yes | Nutrition Edu.; Help following programme end | Dietitian; Nutritionist | Yes | Individual | Face to Face; Telephone; Print | Community | 11.5 | 3 | 8 | Biweekly phone calls (Week 1-12); Two single sessions at the beginning and end of the Intervention phase | "The number of personal contacts and counseling sessions was the same for all study participants overall, but individuals in the ICR and CCR arms received longer and more comprehensive counseling sessions with personalized dietary plans, specific for the ICR or CCR regimens." | Yes |
|  | Intermittent Calorie Restriction | Diet only | Yes | Nutrition Edu.; Inter. Fasting; Help following programme end | Dietitian; Nutritionist | Yes | Individual | Face to Face; Telephone; Print | Community | 11.5 | 3 | 8 | Biweekly phone calls (Week 1-12); Two single sessions at the beginning and end of the Intervention phase | "The number of personal contacts and counseling sessions was the same for all study participants overall, but individuals in the ICR and CCR arms received longer and more comprehensive counseling sessions with personalized dietary plans, specific for the ICR or CCR regimens." | Yes |
| Seligman 2011 | Standard-of-care strategy | Diet only |  | Nutrition Edu. |  | Unclear | Individual | Print |  | 3 | 3 |  |  |  | Yes |
|  | Healthy diet and step counter | Diet and exercise |  | Nutrition Edu. |  | Unclear | Individual | Face to Face; Print |  | 3 | 3 | 2 |  |  | No |
|  | Healthy diet and fitness | Diet and exercise |  | Nutrition Edu. |  | Unclear | Individual | Face to Face; Print |  | 3 | 3 | 2 |  |  | Yes |
| Shikany 2013 | Food-based diet | Diet only | Yes | Nutrition Edu. |  | No | Individual | Face to Face; Telephone; Internet; Print | Health Care; Home | 12 | 6 | 10 | Fortnightly until week 4, monthly until week 20, then six weeks, followed by one fortnight, followed by one month to week 32, two months to week 40 and then six weekly until week 52. |  | Yes |
|  | Meal replacement | Diet only | Yes | MR-P; Nutrition Edu. | Dietitian; Health Trainer | No | Individual | Face to Face; Telephone; Internet | Health Care; Home | 12 | 6 | 10 | Fortnightly until week 4, monthly until week 20, then six weeks, followed by one fortnight, followed by one month to week 32, two months to week 40 and then six weekly until week 52. |  | Yes |
| Silva 2010 | Comparison group |  | No | Nutrition Edu. | Psychologist/ Counsellor; Dietitian; Nutritionist; Exercise physiologist | Unclear | Group | Face to Face | Community | 12 | 12 | 29 |  |  |  |
|  | Intervention | Diet and exercise | Yes | Nutrition Edu.; Help following programme end | Psychologist/ Counsellor; Dietitian; Nutritionist; Exercise physiologist | Unclear | Group | Face to Face; Print | Community | 12 | 12 | 30 | Weekly or twice a month | 120 |  |
| Solbrig 2019 | Motivational interviewing | Diet and exercise | Yes |  | Psychologist/ Counsellor | No | Individual | Face to Face; Telephone; Print | Community | 6 | 6 | 13 | 2 sessions after baseline assessment and fortnightly calls up to 6 months | Session 1: 1 hour Session 2: 35min phone calls: 5–15 min | Yes |
|  | Functional imagery training | Diet and exercise | Yes |  | Psychologist/ Counsellor | No | Individual | Face to Face; Telephone; App | Community | 6 | 6 | 13 | 2 sessions after baseline assessment and fortnightly calls up to 6 months | Session 1: 1 hour Session 2: 35min phone calls: 5–15 min | Yes |
| Somers 2012 | Standard Care | Control | No |  |  | No | Unclear | Unclear | Health Care |  |  |  |  |  |  |
|  | Lifestyle behavioral weight management intervention only | Diet and exercise | Yes | Nutrition Edu.; Help following programme end | Psychologist/ Counsellor; Exercise physiologist | Yes | Group | Face to Face; Telephone; Unclear | Community | 12 | 6 | 21 | Group sessions (12 weekly, 12 biweekly); 3 exercise sessions in 12 weeks; 6 monthly maintenance calls | Group sessions (60 minutes); Exercise sessions (90 minutes); Maintenance calls (20 minutes) | Yes |
|  | Lifestyle behavioral weight management intervention + Pain Coping Skills Training | Diet and exercise | Yes | Nutrition Edu.; Help following programme end | Psychologist/ Counsellor; Exercise physiologist | Yes | Group | Face to Face; Telephone; Unclear | Community | 12 | 6 | 21 | Group sessions (12 weekly, 12 biweekly); 3 exercise sessions in 12 weeks; 6 monthly maintenance calls | Group sessions (120 mins); Exercise sessions (90 mins); Maintenance calls (20 mins) | Yes |
|  | Pain Coping Skills Training only (N/A) | N/A | N/A | N/A | N/A | N/A | N/A | N/A | N/A | N/A | N/A | N/A | N/A | N/A | N/A |
| Spring 2013 | MOVE standard care | Diet and exercise | Yes | Nutrition Edu. | Physician; Psychologist/ Counsellor; Dietitian; Other AHPs | Unclear | Group | Face to Face | Community | 12 | 6 | 19 | Biweekly | 1.5 hours | Yes |
|  | MOVE + personal digital assistant | Diet and exercise | Yes | Nutrition Edu. | Physician; Psychologist/ Counsellor; Dietitian | Unclear | Individual and Group | Face to Face; Telephone; Other | Community; Home | 12 | 6 | 32 | Biweekly | 1.5 hours | Yes |
| Spring 2017 | Control self-guided program | Diet and exercise | Yes | Nutrition Edu.; Fin. Incentives; |  | Unclear | Group | Face to Face; Print; Video | Community; Home | 6 | 6 | 1 | Single session | 60 | No |
|  | Standard weight loss program | Diet and exercise | Yes | Nutrition Edu.; Fin. Incentives | Psychologist/ Counsellor; Exercise physiologist | Unclear | Individual and Group | Face to Face; Telephone; Print | Community | 6 | 6 | 19 | Weekly group sessions and calls (Months 1-2); Monthly calls (Months 3-6) | Group session (90 mins); Guided walking exercise (30 mins); Telephone calls (10-15 mins) | Yes |
|  | Technology-supported | Diet and exercise | Yes | Nutrition Edu.; Fin. Incentives | Psychologist/ Counsellor; Exercise physiologist | Unclear | Individual and Group | Face to Face; Telephone; Internet; App; Print; SMS | Community; Home | 6 | 6 | 19 | Weekly group sessions and calls (Months 1-2); Monthly calls (Months 3-6) | Group session (90 mins); Guided walking exercise (30 mins); Telephone calls (10-15 mins) | Yes |
| Stahre 2005 | Control | Control | No |  |  |  |  |  |  |  |  |  |  |  |  |
|  | Cognitive treatment | Diet only | No | Nutrition Edu. |  |  | Group | Face to Face | Health Care | 2.3 | 6 | 10 | Weekly | 3 hours | Yes |
| Stenius-Aarniala 2000 | Control | Diet only | No | Nutrition Edu. |  | No | Group | Face to Face | Health Care | 3.2 | 3.2 | 12 | Weekly | 30 |  |
|  | Treatment with VLCD | Diet only | No | MR-F; Nutrition Edu. |  | Yes | Group | Face to Face | Health Care; Home | 3.2 | 3.2 | 12 | Weekly sessions for 14 weeks, 8 weeks VLCD |  |  |
| Stevens 1993 | Control | Control | No |  |  | No |  |  |  | 0 | 0 | 0 |  |  | No |
|  | Intervention | Diet and exercise | Yes | Nutrition Edu.; Help following programme end | Psychologist/ Counsellor; Dietitian; Exercise physiologist | Unclear | Individual and Group | Face to Face; Telephone; Internet; Print | Community | 18 | 3 | 54 |  | 90 minutes group, individual length not reported.  'The intervention started with an individual counseling session, followed by 14 weekly group meetings led by dietitians or health educators. After this 14-week intensive phase, participants attended six biweekly group meetings and then monthly group meetings. Beginning in the 18th month, participants were offered a variety of options to keep them involved in the intervention, including individual counseling sessions and special group sessions focused on selected weight loss topics.' | Yes |
| Stevens 2001 | Control | Control | No |  |  | Unclear |  |  |  |  |  | 0 |  |  |  |
|  | Intervention | Diet and exercise | Yes | Nutrition Edu.; Help following programme end | Dietitian; Health Trainer | Unclear | Individual and Group | Face to Face; Telephone; Print; Other | Community | 36 | 6 | 51 | Intensive phase (0-6 Months): 1 individual, 14 weekly, 6 biweekly sessions. Extended phase (7-36 Months): biweekly contacts with monthly face-to-face meetings until the intensive intervention is completed for the first cohort then mini-modules to be offered with continued biweekly contact. Specifically tailored follow-up where indicated. | 90 mins in first phase | Yes |
|  | Sodium only intervention (N/A) | N/A | N/A | N/A | N/A | N/A | N/A | N/A | N/A | N/A | N/A | N/A | N/A | N/A | N/A |
|  | Combined intervention (N/A) | N/A | N/A | N/A | N/A | N/A | N/A | N/A | N/A | N/A | N/A | N/A | N/A | N/A | N/A |
| Strobl 2013 | Control, Usual care | Diet and exercise | No | Nutrition Edu. |  | Yes | Individual and Group | Face to Face | Inpatient | 0.7 | 0.7 | NR |  | 3-week treatment (nutrition therapy, physical exercise, and psychoeducation), number and length of sessions not stated. | Yes |
|  | Telephone aftercare | Diet and exercise | Yes | Nutrition Edu. | Personal Trainer | Yes | Individual and Group | Face to Face; Telephone | Inpatient; Health Care; Home | 6 | 0.7 | NR + 8 |  | 3-week treatment (nutrition therapy, physical exercise, and psychoeducation), number and length of sessions not stated. PLUS 8 sessions [1 x 50 min group session; 1 x 10 min individual; 6 x 5-10 min telephone call]. | Yes |
| Sundfor 2018 | Continuous energy restriction | Diet only | Yes | Nutrition Edu.; Help following programme end | Dietitian | Unclear | Individual | Face to Face; Telephone; Print; Internet | Health Care | 12 | 6 | 10 | "Follow-up visits were scheduled at biweekly intervals up to eight weeks, and thereafter monthly up to six months for a total of 10 visits." |  | Yes |
|  | Intermittent energy restriction | Diet only | Yes | Nutrition Edu.; Inter. Fasting; Help following programme end | Dietitian | Unclear | Individual | Face to Face; Telephone; Print; Internet | Health Care | 12 | 6 | 10 | "Follow-up visits were scheduled at biweekly intervals up to eight weeks, and thereafter monthly up to six months for a total of 10 visits. |  | Yes |
| Tapsell 2017 | Usual care (Control) | Diet and exercise | Yes | Nutrition Edu. | Nurse (General) | Unclear | Individual | Face to Face; Telephone; Print | Health Care | 12 | 3 | 11 | Months 1-3: Monthly;  Months 1 – 12: Quarterly;  Phone calls: Quarterly | 30 mins clinics; 15 min phone calls | Yes |
|  | Intervention Group | Diet and exercise | Yes | Nutrition Edu. | Dietitian; Health Trainer | Yes | Individual | Face to Face; Telephone; Print | Health Care | 12 | 3 | 11 | Months 1-3: Monthly;  Months 1 – 12: Quarterly;  Phone calls: Quarterly | 1 hour clinics; 15 min phone calls | Yes |
|  | Intervention plus food supplement group (N/A) | N/A | N/A | N/A | N/A | N/A | N/A | N/A | N/A | N/A | N/A | N/A | N/A | N/A | N/A |
| TarragaMarcos 2017 | G3 | Diet and exercise | No | Nutrition Edu. |  | Unclear | Group | Face to Face | Health Care |  |  | 1 |  |  | No |
|  | G2 | Diet and exercise | Yes | Nutrition Edu. |  | Unclear | Group | Face to Face; Internet; Other | Health Care; Home | 12 | 3 | 6 | After the initial visit, visits were scheduled after 15 days, 1m, 3m, 6m and one year. |  | No |
|  | G1 | Diet and exercise | Yes | Nutrition Edu.; Help following programme end | Nurse (General) | Unclear | Group | Face to Face | Health Care | 8 | 3 | 10 | Every two weeks from weeks, 1 to 12 and then monthly from weeks 13 to 32 | 1 hour | No |
| Teeriniemi 2018 | Control | Control | No | Nutrition Edu. |  | No | Other | Print | Health Care |  |  | 0 |  |  | Yes |
|  | SHG Counselling | Diet and exercise | No | Nutrition Edu. | Nurse (General) | Yes | Group | Face to Face | Community | 0.7 | 0.7 | 2 |  | 90 | No |
|  | CBT Counselling | Diet and exercise | Yes | Nutrition Edu. | Nutritionist | No | Group | Face to Face | Community | 4.1 | 4.1 | 8 | 7 sessions every second week, last session after 1 month | 90 | No |
|  | Control plus HBCSS | Diet and exercise | No | Nutrition Edu.; Help following programme end |  | No | Other | Internet; Print | Health Care; Community | 12 | 12 | 0 |  | 52-week access to Web-based HBCSS | Yes |
|  | SHG Counselling plus HBCSS | Diet and exercise | No | Nutrition Edu.; Help following programme end | Nurse (General) | Yes | Group | Face to Face; Internet | Community | 12 | 12 | 2 |  | 90 minutes 52-week access to Web-based HBCSS | Yes |
|  | CBT Counselling plus HBCSS | Diet and exercise | Yes | Nutrition Edu.; Help following programme end | Nutritionist | No | Group | Face to Face; Internet | Community | 12 | 12 | 8 | 7 sessions every second week, last session after 1 month | 90 minutes 52-week access to Web-based HBCSS | Yes |
| ter Bogt 2009 | GP usual care | Control | No |  | GP | Unclear | Individual | Face to Face | Health Care |  |  | 1 |  | 10 | No |
|  | Lifestyle counselling from NP | Diet and exercise | Yes | Nutrition Edu. | Nurse (General) | Yes | Individual | Face to Face; Telephone | Health Care | 36 | 8 | 11 | Four visits (at months 1, 2, 3, 8); 1 telephone call (5 months) in the first year; one visit and one telephone call per year (year 2, 3) | Average duration of the visits was 35 minutes for the first and second visit (range 15–60 minutes) and 25 minutes for the third visit (range 15–40 minutes). | Yes |
| The Look AHEAD Research Group 2010 | Diabetes support and education | Diet and exercise | No | Nutrition Edu.; Help following programme end | Nurse (General); Dietitian; Health Trainer; Personal Trainer | Yes | Group | Face to Face; Telephone; Print; Other | Community |  | 48 | 22 | 3 sessions annually for the first 4 years of follow-up; thereafter, one session was provided annually | 60 – 90 | No |
|  | Intensive lifestyle intervention | Diet and exercise | Yes | MR-P; Nutrition Edu.; Help following programme end | Nurse (General); Physician; Psychologist/ Counsellor; Dietitian; Personal Trainer | Yes | Individual and Group | Face to Face; Telephone; Internet; Print; Other | Community | 115 | 12 | 134 | Months: 1-6: weekly;  Months 7-12: 3/month;  Years 2-4: Minimum of 1/month;  Year 5+: Monthly recommended. | Months 1-6: Group sessions: 60 to 75 minutes; Individual sessions: 20 to 30 minutes. | Yes |
| Trepanowski 2017 | No-intervention control group | Control | No |  |  | Unclear |  |  | Community |  |  | 0 |  |  | No |
|  | Daily calorie restriction group | Diet only | No | Nutrition Edu.; Help following programme end | Dietitian; Nutritionist | Unclear | Individual | Face to Face; Other | Community | 12 | 6 | 14 | Counselling: Months 4 – 6: weekly;  Months 6 – 12: monthly |  | Yes |
|  | Alternate-day fasting group | Diet only | No | Nutrition Edu.; Inter. Fasting; Help following programme end | Dietitian; Nutritionist | Unclear | Individual | Face to Face; Other | Community | 12 | 6 | 14 | Counselling: Months 4 – 6: weekly;  Months 6 – 12: monthly |  | Yes |
| Tsai 2010 | Control | Control | No | Nutrition Edu. | GP | No | Individual | Face to Face; Print | Health Care | 12 | 12 | 4 | Quarterly | 2 – 3 | No |
|  | Brief counselling | Diet and exercise | No | Nutrition Edu. | Other AHPs; Health care professional (not specified) | Yes | Individual | Face to Face; Telephone; Print | Health Care | 12 | 6 | 12 | PCP visits: quarterly. MA visits: weeks 0, 2, 4, 8, 12, 16, 20, 24 | 15 – 20 | Yes |
| Tuomilehto 2009 | Control | Diet and exercise | No | Nutrition Edu. | Nurse (General); Physician | Yes | Individual |  |  | 12 |  | 3 | At baseline, 3ms and 12m |  | No |
|  | Intervention | Diet and exercise | Yes | MR-F; Nutrition Edu. | Nutritionist; Physiotherapist | Unclear | Individual and Group | Face to Face | Health Care; Home | 12 | 3 | 14 | Every 2 weeks until week 12 then monthly | 60 – 90 | Yes |
| van de Glind 2017 | Comparison group | Control | No | Nutrition Edu. |  | No |  | Print | Community |  |  |  |  |  | No |
|  | EuroFIT group | Diet and exercise | No | Nutrition Edu.; Help following programme end | Health Trainer | Yes | Group | Face to Face; App; Print; Other | Community | 6 - 9 | 3 | 13 | Weekly for Weeks 1 to 12; One reunion meeting held 6–9 months after the program end. | 90 | Yes |
| Viegener 1990 | Intermittent diet | Diet and exercise | No | Nutrition Edu.; Fin. Incentives; Help following programme end | Psychologist/ Counsellor | No | Group | Face to Face |  | 12 | 6 | 26 | Weekly | 2 hours | No |
|  | Standard treatment | Diet and exercise | No | Nutrition Edu.; Fin. Incentives; Inter. Fasting; Help following programme end | Psychologist/ Counsellor | No | Group | Face to Face |  | 12 | 6 | 26 | Weekly | 2 hours | No |
| Vissers 2010 | Control | Control | No |  |  | No |  |  |  |  |  |  |  |  |  |
|  | Diet only group (Diet) | Diet only | Yes | Nutrition Edu. | Dietitian | Unclear | Individual | Face to Face | Community | 12 | 3 | 12 | During the first 3 months participants had a dietary counseling every fortnight. During the next 3 months there was a dietary counseling once a month. 3 more visits months 6-12 |  | Yes |
|  | Diet + fitness training group (Fitness) | Diet and exercise | Yes | Nutrition Edu. | Dietitian; Physiotherapist | Unclear | Individual and Group | Face to Face | Health Care; Community; Home | 12 | 3 | 51 | As per diet only plus 2 x week for first 3m, 1 x week for second 3m |  | Yes |
|  | Diet + WBV group (Vibration) | Diet and exercise | Yes | Nutrition Edu. | Dietitian; Physiotherapist | Unclear | Individual and Group | Face to Face | Community; Home | 12 | 3 | 51 | As per diet only plus 2 x week for first 3m, 1 x week for second 3m |  | Yes |
| Volpe 2008 | Exercise only | Exercise only | Yes | Help following programme end | Other | Yes | Group | Face to Face; Telephone; Internet | Community; Home | 12 | 6 | 96 | 3/4/5 days/week exercise sessions; Monthly and periodic phone/email contact | 30 |  |
|  | Diet only | Diet only | Yes | Nutrition Edu.; Help following programme end |  | Unclear | Group | Face to Face; Telephone; Internet | Community | 12 | 6 | 18 | Weekly; biweekly nutrition sessions; Monthly; periodically phone/email contact |  |  |
|  | Combination of diet and exercise | Diet and exercise | Yes | Nutrition Edu.; Help following programme end | Other | Yes | Group | Face to Face; Telephone; Internet | Community; Home | 12 | 6 | 114 | 3/4/5 days/week exercise sessions; Weekly; biweekly nutrition sessions; Monthly; periodically phone/email contact | 30 mins exercise sessions; duration of nutritional sessions not reported. |  |
| von Gruenigen 2012 | Control | Control | No | Nutrition Edu.; |  | Unclear | Individual | Face to Face; Print | Health Care |  |  | 1 | Once |  | No |
|  | Intervention | Diet and exercise | Yes | Nutrition Edu. | Physician; Psychologist/ Counsellor; Dietitian; Physiotherapist | Unclear | Individual and Group | Face to Face; Print; Internet | Health Care | 12 | 6 | 16 | Group sessions (10 weekly followed by 6 bi-weekly); Physician face-to-face counseling visits occurred at 3, 6 and 12 months | Group sessions were 60 min | Yes |
| von Gruenigen 2008 | Control, Usual care | Control | No | Nutrition Edu. |  |  | Unclear | Print | Health Care |  |  | 0 |  |  | No |
|  | Lifestyle intervention | Diet and exercise | Yes | Nutrition Edu.; Help following programme end | Psychologist/ Counsellor; Dietitian; Other |  | Individual and Group | Face to Face; Telephone; Print | Health Care | 6 | 6 | 24 | Weekly for 6 weeks, bi-weekly for I month, and monthly for 3 months. |  | Yes |
| Wadden 1994 | Balanced deficit diet | Diet and exercise | Yes | Nutrition Edu.; Help following programme end | Psychologist/ Counsellor; Dietitian | Yes | Group | Face to Face |  | 18 | 12 | 65 | First 52 weeks: weekly. Weeks 53-78; fortnightly | 1.5 hours | Yes |
|  | Very low-calorie diet | Diet and exercise | Yes | MR-P; MR-F; Nutrition Edu.; Help following programme end | Psychologist/ Counsellor; Dietitian | Yes | Group | Face to Face |  | 18 | 12 | 65 | First 52 weeks: weekly. Weeks 53-78; fortnightly | 1.5 hours | Yes |
| Wadden 1998 | Diet alone, Control | Diet only | Yes | MR-P; Nutrition Edu. | Psychologist/ Counsellor |  | Group | Face to Face; Print | Community; Home | 11.1 | 3.9 | 38 | First 28 weeks: weekly. Then fortnightly for 20 weeks. Once every 3 months in 2nd year. | 1.5 | No |
|  | Diet plus aerobic exercise | Diet and exercise | Yes | MR-P; Nutrition Edu. | Psychologist/ Counsellor | Yes | Group | Face to Face; Print | Community; Home | 11.1 | 3.9 | 160 | First 28 weeks: weekly. Then fortnightly for 20 weeks. Once every 3 months in 2nd year. | 1.5 | No |
|  | Diet plus strength training | Diet and exercise | Yes | MR-P; Nutrition Edu. | Psychologist/ Counsellor | Yes | Group | Face to Face; Print | Community; Home | 11.1 | 3.9 | 160 | First 28 weeks: weekly. Then fortnightly for 20 weeks. Once every 3 months in 2nd year. | 1.5 | No |
|  | Diet plus aerobic and strength training | Diet and exercise | Yes | MR-P; Nutrition Edu. | Psychologist/ Counsellor | Yes | Group | Face to Face; Print | Community; Home | 11.1 | 3.9 | 160 | First 28 weeks: weekly. Then fortnightly for 20 weeks. Once every 3 months in 2nd year. | 1.5 | No |
| Wadden 2004 | Nondieting approach | Diet and exercise | Yes | Nutrition Edu. | Psychologist/ Counsellor; Dietitian | No | Group | Face to Face |  | 14 | 10 | 31 | Weekly for 20 weeks, every other week for weeks 22-40, follow up sessions 52 and 65 | 90 | No |
|  | Balanced-deficit diet | Diet and exercise | Yes | Nutrition Edu.; Help following programme end | Psychologist/ Counsellor; Dietitian | No | Group | Face to Face |  | 14 | 10 | 31 | Weekly for 20 weeks, every other week for weeks 22-40, follow up sessions 52 and 65 | 90 | No |
|  | Meal replacement plan | Diet and exercise | Yes | MR-P; Nutrition Edu.; Help following programme end | Psychologist/ Counsellor; Dietitian | No | Group | Face to Face |  | 14 | 10 | 31 | Weekly for 20 weeks, every other week weeks 22-40, follow up sessions 52 and 65 | 90 | No |
| Waleekhachonloet 2007 | Individual behavior therapy | Diet only | No | Nutrition Edu. | Health Trainer |  | Individual | Face to Face | Community | 3 | 3 | 5 | One per two weeks | First session: 2h all the rest: 30 min. | Yes |
|  | Group behavior therapy | Diet only | No | Nutrition Edu. | Health Trainer |  | Group | Face to Face | Community | 3 | 3 | 5 | One per two weeks | First session: 2h all the rest: 60 min. | Yes |
| Weinstock 2013 | Conference Call DPP | Diet and exercise | Yes | Nutrition Edu. | Nurse (General); Dietitian; Other | Yes | Group | Telephone; Print | Health Care | 24 | 24 | 40 plus 6 optional | Educators: weekly-5 weeks, monthly-1 year; Coaches: Monthly (Year 1) |  | Yes |
|  | Individual Call DPP | Diet and exercise | Yes | Nutrition Edu. | Nurse (General); Dietitian; Other | Yes | Individual | Telephone; Print | Health Care | 24 | 24 | 40 + 6 optional | Educators: weekly-5 weeks, monthly-1 year; Coaches: Monthly (Year 1) |  | Yes |
| West 2007 | Attention control | Diet and exercise | Yes | Nutrition Edu.; Help following programme end | Nutritionist; Exercise physiologist; Health Trainer | No | Group | Face to Face |  | 18 | 6 | 47 | Weekly for 6m, Biweekly for 6m, and then monthly for 6m. | 45 | No |
|  | Motivational interviewing | Diet and exercise | Yes | Nutrition Edu.; Help following programme end | Psychologist/ Counsellor; Nutritionist; Exercise physiologist; Health Trainer | No | Individual and Group | Face to Face |  | 18 | 6 | 47 | Weekly for 6m, Biweekly for 6m, and then monthly for 6m. Five individual motivational interviewing sessions were offered, with the first session before starting group therapy and then at 3, 6, 9, and 12m. | 45 | Yes |
| West 2011 | Control | Control | No |  |  |  |  |  |  |  |  |  |  |  | No |
|  | Lifestyle Intervention | Diet and exercise | No | Nutrition Edu.; Help following programme end | Health Trainer | Yes | Group | Face to Face; Print | Community | 12 | 4 | 20 | Weekly for first 4m (12 weeks?), then monthly for 8m | 60 | Yes |
| Whelton 1998 | Non-weight loss (Usual lifestyle, control group plus sodium reduction) | Control | No |  |  | Unclear | Group | Face to Face | Community | 12 | 12 | 3 | Quarterly for 1 year |  | No |
|  | Weight loss (Weight loss alone plus weight loss and sodium reduction combined intervention) | Diet and exercise | Yes | Nutrition Edu.; | Nutritionist; Personal Trainer | Unclear | Individual and Group | Face to Face; Telephone; Print | Community | 30 | 8 | 46 | Weekly during the intensive phase (Months 0-4); Biweekly during the extended phase (Months 5-8); Monthly during the maintenance phase. |  | Yes |
| Wilson 2016 | Control - Self Study Group | Diet and exercise | No | Nutrition Edu. |  | No | Other – self-study | Print | Home |  |  | 1 |  |  | No |
|  | Phone Fuel Your Life | Diet and exercise | Yes | Nutrition Edu. | Health Trainer | No | Individual | Telephone; Print | Home | 12 | 6 | 11 | 0-2m: biweekly.  2-6m: monthly.  6-12m: bimonthly. | 20 mins' 8 sessions with a health coach | Yes |
|  | Group Fuel Your Life | Diet and exercise | Yes | Nutrition Edu. | Health Trainer | No | Group | Face to Face; Print | Workplace | 12 | 6 | 11 | 0-2m: biweekly.  2-6m: monthly.  6-12m: bimonthly. | 60 mins' 8 sessions with a health coach | No |
| Wilson 2016b | Control | Control | No |  |  | No |  |  |  |  |  |  |  |  | No |
|  | FUEL Your Life peer health coaches + nurse education | Diet and exercise | No | Nutrition Edu. | Nurse (General); Dietitian; Health Trainer | Yes | Individual and Group | Face to Face; Print | Workplace | 6 | 6 | 6 | Monthly | Baseline: initial 1:1 session 0-6m: 6 x 10 min group sessions and weekly announcements. | Yes |
| Wing 1988 | Diet plus placebo exercise | Diet only | Yes | Nutrition Edu.; Fin. Incentives; Help following programme end |  |  | Group | Face to Face | Health Care; Home | 8.5 | 2.5 | 26 | Both groups participated in a behavioural weight control programme, with group meetings held twice a week for 10 weeks and monthly for the following 6m. | 1 hour | No |
|  | Diet plus moderate exercise | Diet and exercise | Yes | Nutrition Edu.; Fin. Incentives; Help following programme end |  |  | Group | Face to Face | Health Care; Home | 8.5 | 2.5 | 26 | Both groups participated in a behavioural weight control programme, with group meetings held twice a week for 10 weeks and monthly for the following 6m. | 1 hour | No |
| Wing 1988b | Diet only | Diet only | Yes | Nutrition Edu.; Fin. Incentives; ; Help following programme end | Psychologist/ Counsellor | No | Group | Face to Face | Health Care; Home | 14 | 2.5 | 52 | Both groups attended treatment sessions 3 times/week (versus 2 times/week in Study 1) for 10 weeks. After this intensive training period, subjects met weekly for an additional 10 weeks and then monthly for a year. | 1 hour | No |
|  | Diet plus exercise | Diet and exercise | Yes | Nutrition Edu.; Fin. Incentives; Help following programme end | Psychologist/ Counsellor | No | Group | Face to Face | Health Care; Home | 14 | 2.5 | 52 | Both groups attended treatment sessions 3 times/week (versus 2 times/week in Study 1) for 10 weeks. After this intensive training period, subjects met weekly for an additional 10 weeks and then monthly for a year. | 1 hour | No |
| Wing 1998 | Control | Diet and exercise | No | Nutrition Edu. |  | No |  | Print |  |  |  |  |  |  | No |
|  | Diet | Diet only | No | Nutrition Edu.; Help following programme end | Psychologist/ Counsellor; Dietitian | No | Group | Face to Face | Community; Home | 24 | 6 | 51 | Weekly for the first 6m;  Biweekly for the next 6m |  | No |
|  | Exercise | Exercise only | No |  | Psychologist/ Counsellor; Exercise physiologist | No | Group | Face to Face | Community; Home | 24 | 6 | 51 | Weekly for the first 6m;  Biweekly for the next 6m | 50 – 60 min walk with the therapist at each of these weekly meetings. | No |
|  | Diet plus exercise | Diet and exercise | No | Nutrition Edu.; Help following programme end | Psychologist/ Counsellor; Dietitian; Exercise physiologist | No | Group | Face to Face | Community; Home | 24 | 6 | 51 | Weekly for the first 6m;  Biweekly for the next 6m |  | No |
| Wing 2010 | Structured Education Program | Diet and exercise | Yes | Nutrition Edu. | Psychologist/ Counsellor | Unclear | Group | Face to Face | Community | 15 | 6 | 7 | Months 1, 2, 3, 4, 6, 9, and 15 | 1 hour | No |
|  | Weight Loss Intervention (Skills Based maintenance) | Diet and exercise | Yes | MR-P; Nutrition Edu.; Help following programme end | Psychologist/ Counsellor | Yes | Group | Face to Face | Community | 18 | 6 | 50 | Weekly for 6 months and every other week for 12 months | 1 hour | Yes |
|  | Weight Loss Intervention (Motivation Based maintenance) | Diet and exercise | Yes | MR-P; Nutrition Edu.; Help following programme end | Psychologist/ Counsellor | Yes | Group | Face to Face | Community | 18 | 6 | 50 | Weekly for 6 months and every other week for 12 months | 1 hour | Yes |
| Yannakoulia 2008 | Usual care group | Control | No | Nutrition Edu. | Dietitian | No | Individual | Face to Face | Health Care |  |  | 1 |  |  | Yes |
|  | Intensive care group | Diet and exercise | No | Nutrition Edu. | Dietitian | No | Individual | Face to Face | Health Care | 2 | 2 | 5 | Every two weeks |  | Yes |
| Yardley 2014 | Usual care | Control | No |  | Other AHPs | No |  |  | Health Care |  |  | 0 |  |  |  |
|  | Web-based only | Diet and exercise | No | Nutrition Edu.; Help following programme end |  | No | Individual | Internet; Other | Health Care; Home | 3 | 3 | 0 | Instructed to access website weekly |  | Yes |
|  | Basic nurse support | Diet and exercise | Yes | Nutrition Edu.; Help following programme end | Nurse (General) | No | Individual | Face to Face; Telephone; Internet | Health Care; Home | 3 | 3 | 3 | 2 weeks, 1m, 3m | 15 – 20 | Yes |
|  | Regular nurse support | Diet and exercise | No | Nutrition Edu.; Help following programme end | Nurse (General) | No | Individual | Face to Face; Telephone; Internet | Health Care; Home | 6 | 6 | 7 | 2 weeks, and then monthly for the first 6m | 15 – 20 | Yes |
| Yates 2018 | Placebo + no lifestyle | Control | No |  |  |  |  |  |  |  |  |  |  |  |  |
|  | Metformin + no lifestyle (N/A) | N/A | N/A | N/A | N/A | N/A | N/A | N/A | N/A | N/A | N/A | N/A | N/A | N/A | N/A |
|  | Placebo + lifestyle | Diet and exercise | No | Nutrition Edu. |  | No | Individual and Group | Face to Face |  | 4 | 4 | 16 | Weekly |  | Yes |
|  | Metformin + lifestyle (N/A) | N/A | N/A | N/A | N/A | N/A | N/A | N/A | N/A | N/A | N/A | N/A | N/A | N/A | N/A |
| Yeh 2003 | Counseling based intervention | Diet and exercise | Yes | Nutrition Edu.; Help following programme end | Dietitian | No | Individual | Face to Face | Community | 6 | 6 | 6 | Monthly | 2 x 1 hour. 4 x 30 min. | Yes |
|  | Skills based intervention | Diet only | No | Nutrition Edu.; Help following programme end | Dietitian | No | Individual and Group | Face to Face; Telephone; Internet | Community; Home | 6 | 6 | 7 | Monthly | 2 x 90 mins 2 x 2 hour (supermarket) 2 x 90 mins (restaurant) 1 x 2 hr (home) | Yes |
| Yin 2018 | Comparison-Control Group | Diet and exercise | No | Nutrition Edu. | Nurse (General) | No | Individual and Group | Face to Face | Health Care | 6 | 6 | 7 | Every three-four weeks | Participants in the comparison group received a counselling session and were invited to attend 6 general health education classes on PA, nutrition, chronic diseases (obesity, diabetes, heart diseases) and menopause at the same venue as the intervention group. |  |
|  | Intervention Group | Diet and exercise | No | Nutrition Edu. | Health Trainer | Yes | Individual and Group | Face to Face; Telephone | Health Care; Home | 6 | 6 | 22 | Weekly | 1 hour | Yes |
| Zhang 2016 | Control | Control | Yes |  | Other | No | Group | Face to Face |  | 12 | 12 | 18 | Biweekly | All participants attended group health education sessions, which were held biweekly in the first 6m and monthly in the last 6m of the intervention. | No |
|  | Moderate exercise | Exercise only | No |  | Other | No | Other - education sessions: group-based; moderate-exercise sessions unsupervised | Face to Face; Telephone | Home | 12 | 12 | 18 | Biweekly and weekly | Participants were instructed to briskly walk at approximately 120 steps per minute for 30 minutes per session and 5 sessions per week.  All participants attended group health education sessions, which were held biweekly in the first 6m and monthly in the last 6m of the intervention.  plus, Participants in the moderate exercise program were required to wear pedometers and record their daily exercise in a log, which was reviewed weekly by study staff. | No |
|  | Vigorous-moderate exercise | Exercise only | No |  | Physician; Other | No | Individual and Group | Face to Face; Telephone | Community; Home | 12 | 12 | 138 | Biweekly and weekly | Participants were required to participate in 5, 30-min., vigorous exercise sessions each week supervised by a study physician at a local community health center.  All participants attended group health education sessions, which were held biweekly in the first 6m and monthly in the last 6m of the intervention.  Participants were required to participate in 5 vigorous exercise sessions each week supervised by a study physician at a local community health center. After 6 months of vigorous exercise, participants switched to moderate exercise for another 6 months.  plus, Participants in the moderate exercise program were required to wear pedometers and record their daily exercise in a log, which was reviewed weekly by study staff. | No |
| **Approx.:** Approximately; **Appt.**: Appointment/s; **Fin. Incentives;** Financial Incentives**; GP:** General Practitioner **Inter. Fasting:** Intermittent Fasting; **Min/s:** Minute/s **M/Mths:** month/s**; MR – F =** Meal replacement (Full); **MR – P =** Meal replacement (Partial); **N:** Number; **N/A:** Not applicable; **NR:** Nor reported; **Nutrition Edu.** = Nutrition Education; **PA:** Physical Activity**; SMS**: Short Message Service; **VLCD:** Very low-calorie diet  ^a^ See table below for Provider category descriptions ^b^ Unless otherwise stated; ^c^ Exercise sessions were assumed to be unsupervised and did not contribute to the number of sessions unless otherwise stated. ^Study only included in the CNMA involving intensity components.   \| **Provider** \| **Provider descriptions as reported in included studies** \| \| --- \| --- \| \| Nurse (Specialist) \|  \| \| Nurse (General) \| Nurse educator;  RNS; \| \| GP \| General internists \| \| Physician  (Any doctor not a GP) \| Medical doctors; Specialists in endocrinology, and internal medicine; Clinicians; Endocrinologists; Graduates in medicine; Research cardiologist; Doctoral-level clinicians (with an average of 4.8 years of experience delivering behavioral weight loss treatment); Occupational doctor. \| \| Psychologist/ Counsellor \| Therapist; Masters-level counseling psychology students; MA in behavioural psychology; Lifestyle counsellor; Graduates In psychology; Psychology graduate students; Advanced degree in behavioral psychology; Mental health counsellor; Wellness counsellors; Professional Counsellor; Psychotherapist; Psychotherapists and masters students graduate students in clinical psychology; Clinical psychology graduate students; Lifestyle counsellor; Clinical psychology graduate students; Experienced behavioural weight control counsellors; Behavior therapist; Counsellor with a degree in nutrition or physical activity \| \| Dietitian \| Dietitian; Masters of Dietetics Students \| \| Nutritionist \| Provider described by authors as nutritionist; Nutrition technician; Graduates in nutrition; Advanced degree in nutrition; Nutritional interventionist; Nutritionist (MSc in nutrition); Nutrition/Diet interventionists; Two qualified or student clinical nutritionists \| \| Physiotherapist \| Physical therapist; Physical/recreational therapists \| \| Exercise physiologist \| Exercise consultants; MA in exercise physiology; Graduates in physical activity and sport science (SPAS); Advanced degree in exercise physiology; Exercise counsellors \| \| Other Allied Health Professionals \| Occupational therapist; Pharmacist; Nurses/physician assistants; Hospital staff; Social worker with special competence in CT; Medical-assistant \| \| Health trainer \| Lifestyle coaches; Mindfulness meditation instructors; Community health educator; MA in health education; Behavioural consultant; Health educator; Telephone counsellors; Trained lifestyle coaches; Health Promotion coaches; Weight loss coaches; Wellness leader; Weight Watchers leader; Trained interventionists with expertise in both content area (i.e., physical activity and nutrition) and behavioral therapy;  Food advisors recruited from local community; Community Health Workers; Lifestyle activity consultant; Trained lifestyle coaches; Lifestyle Coach/ medical assistant; Masters-level staff with extensive training in behavioral weight loss; Nutrition health educator; IHM health staff graduates; 6 trained CAMWEL advisors recruited from various occupational backgrounds including healthcare, in line with the NHS health trainers initiative; Weight loss group leaders supervised by an exercise physiologist; Study coordinator (with health/nutrition background) together with a peer leader/study coordinator (experienced in adult training and self-management programs); Health educator; Degree in health sciences; Trainers (for meal replacement group); Health coach and health practitioner backgrounds and trained by the senior psychologists; Diabetes educators; EuroFIT coaches; Program providers who were trained in nutrition, education, and behavioral interventions; Masters degree–level health educators delivered health education sessions; Behaviorist;  Trained lay health educators (LHEs) (community volunteers or existing senior center staff); Peer health coach; Educators held an undergraduate degree in a relevant discipline (dietician, sports scientist) \| \| Personal Trainer \| Certified exercise trainer; Trained fitness instructor; Physical activity specialist; Football coaching staff; Physical Activity Counselor; Trained interventionist and exercise coaches who were skilled in exercise science; Exercise programme supervised by a professional trainer; Fitness professional; Exercise interventionists; Exercised in a supervised setting; Trained certified technicians assessed each participant;  Sports therapist; Exercise specialists; \| \| Health care professional (not specified) \| Church health advisors (CHAs) were members of their respective church’s health ministry (e.g., nurses, pharmacists, physicians) and were trained by a co-investigator certified to perform GLB training;  Standard clinical care provider; Hospital based care; Primary care providers; Master's trained health professionals; health professional \| \| Other \| Research staff; Behavioral specialist; PhD-level interventionists; Doctoral level graduate students; Research assistant; Case manager; Coaches; YMCA staff; Peer leader; Teacher; Interventionist; Successful group members selected through interview; Varied, may be successful slimmers; Well-trained investigators; Research assistant; PhD holders or PhD candidates in at least their third year of study; BE WELL intervention staff; Physical activity, psychological support male researcher; The tutors; Study investigator; Ergonomist; Study coordinator; Interventionist; Cooperative Extension Service Family and Consumer Sciences Agents or individuals with bachelors or masters degrees in nutrition, exercise science, or psychology; Study partner; Trained interventionists; Group facilitator; External people representing diverse areas of expertise;  Two experienced coleaders; Administrative study staff (not intervention staff); Trained graduate or undergraduate students; Had backgrounds in dietetics, psychology and/or exercise physiology; Primary investigator; Study staff \| \| If it was an OR between providers, both were listed \| \| | | | | | | | | | | | | | | | |

## **Supplemental Table S6.** Component effect estimates (MD in kg, 95% CrI) across models^[[1]](#footnote-1)^

|  | | **Component Network Meta-Analysis (CNMA)** | | | | **Component Network Meta-Regression (CNMR)** | | | |
| --- | --- | --- | --- | --- | --- | --- | --- | --- | --- |
|  |  | **Additive model Excluding intensity components** | **Additive model Excluding intensity components and excluding % weight change** | **Additive model Including intensity components** | **Interaction model Excluding intensity components** | **Control group mean weight change** | **Mean  baseline BMI** | **Selection based on disease status** | **Recruitment method (prompted vs self-initiated)** |
| Group | Component | (n studies: 169, n arms: 382) | (n studies: 155, n arms: 350) | (n studies: 48, n arms: 105) | (n studies: 169, n arms: 382) | (n studies: 169, n arms: 382) | (n studies: 159, n arms: 358) | (n studies: 168, n arms: 380) | (n studies: 139, n arms: 312) |
| Type | Diet | -1.84 (-2.91 to -0.80) | -1.95 (-2.98 to -0.90) | -0.83 (-3.38 to 1.70) | -1.74 (-3.10 to -0.35) | -1.96 (-2.62 to -1.31) | -1.99 (-2.69 to -1.27) | -1.84 (-2.56 to -1.13) | -1.79 (-2.61 to -1.01) |
| Type | Exercise | 0.10 (-0.86 to 1.08) | 0.30 (-0.70 to 1.28) | -0.66 (-3.28 to 1.85) | 0.44 (-1.29 to 2.20) | -0.04 (-0.86 to 0.78) | -0.01 (-0.89 to 0.86) | -0.04 (-0.86 to 0.78) | -0.16 (-1.07 to 0.76) |
| Content | Meal Replacement Partial | -2.12 (-3.39 to -0.89) | -1.24 (-2.63 to 0.15) | -2.83 (-4.86 to -0.96) | -2.16 (-3.42 to -0.91) | -0.94 (-2.17 to 0.30) | -1.26 (-2.65 to 0.14) | -0.93 (-2.18 to 0.30) | -0.97 (-2.36 to 0.40) |
| Content | Meal Replacement Full | -2.63 (-4.58 to -0.73) | -2.92 (-4.82 to -1.04) | 0.13 (-6.13 to 6.37) | -2.82 (-4.86 to -0.76) | -1.90 (-3.69 to -0.10) | -1.80 (-3.73 to 0.09) | -1.96 (-3.79 to -0.11) | -3.09 (-5.17 to -0.92) |
| Content | Financial Incentives | -1.61 (-4.09 to 0.91) | -2.27 (-5.50 to 0.92) | - | -1.60 (-4.14 to 0.89) | 0.19 (-2.22 to 2.61) | 0.10 (-2.46 to 2.62) | 0.02 (-2.39 to 2.45) | 0.01 (-2.34 to 2.39) |
| Content | Intermittent Fasting | 0.65 (-1.52 to 2.82) | 0.99 (-1.90 to 3.90) | -0.01 (-5.02 to 5.02) | 0.59 (-1.61 to 2.76) | 0.41 (-1.72 to 2.55) | 0.24 (-2.04 to 2.50) | 0.51 (-1.60 to 2.60) | 0.57 (-1.52 to 2.63) |
| Provider | Nurse | 1.04 (-0.17 to 2.24) | 0.95 (-0.28 to 2.21) | 0.12 (-2.32 to 2.53) | 0.96 (-0.27 to 2.17) | 0.94 (-0.17 to 2.06) | 0.96 (-0.20 to 2.14) | 0.81 (-0.35 to 1.96) | 1.03 (-0.19 to 2.26) |
| Provider | Physician/GP | -0.84 (-2.09 to 0.41) | -0.76 (-2.09 to 0.56) | -0.58 (-3.60 to 2.46) | -0.88 (-2.11 to 0.39) | -0.45 (-1.60 to 0.70) | -0.37 (-1.55 to 0.83) | -0.48 (-1.63 to 0.68) | -0.46 (-1.66 to 0.76) |
| Provider | Psychologist/ Counsellor | -1.45 (-2.81 to -0.06) | -1.15 (-2.54 to 0.27) | -2.11 (-4.77 to 0.68) | -1.67 (-3.04 to -0.23) | -1.03 (-2.31 to 0.27) | -0.81 (-2.22 to 0.60) | -1.09 (-2.37 to 0.21) | -1.02 (-2.41 to 0.36) |
| Provider | Dietitian | -1.31 (-2.40 to -0.24) | -1.28 (-2.39 to -0.17) | -3.54 (-6.94 to -0.31) | -0.17 (-2.59 to 2.31) | -1.32 (-2.30 to -0.33) | -1.24 (-2.29 to -0.19) | -1.45 (-2.50 to -0.40) | -1.27 (-2.43 to -0.10) |
| Provider | Nutritionist | -0.97 (-2.50 to 0.57) | -0.83 (-2.43 to 0.77) | 0.33 (-2.60 to 3.33) | -2.39 (-6.01 to 1.21) | -0.79 (-2.23 to 0.65) | -0.69 (-2.25 to 0.82) | -0.81 (-2.25 to 0.62) | -0.68 (-2.28 to 0.91) |
| Provider | Exercise specialist | 0.23 (-0.91 to 1.38) | 0.21 (-0.96 to 1.39) | 3.17 (-0.93 to 7.40) | 2.74 (-0.16 to 5.67) | 0.52 (-0.51 to 1.57) | 0.52 (-0.55 to 1.59) | 0.44 (-0.62 to 1.51) | 0.64 (-0.52 to 1.82) |
| Provider | Health Trainer | -0.44 (-1.59 to 0.77) | -0.68 (-1.89 to 0.56) | 0.80 (-1.79 to 3.71) | -0.72 (-4.12 to 2.78) | 0.48 (-0.69 to 1.65) | 0.59 (-0.61 to 1.80) | 0.42 (-0.74 to 1.61) | 0.42 (-0.87 to 1.75) |
| Provider | Other provider | 0.18 (-1.09 to 1.47) | 0.24 (-1.08 to 1.58) | -1.02 (-3.74 to 1.79) | 0.06 (-1.23 to 1.36) | 0.44 (-0.75 to 1.65) | 0.39 (-0.99 to 1.73) | 0.36 (-0.83 to 1.57) | 0.02 (-1.34 to 1.35) |
| Delivery | Individual | 0.20 (-0.62 to 1.02) | 0.40 (-0.43 to 1.22) | -0.39 (-2.83 to 1.99) | 0.24 (-0.58 to 1.06) | 0.08 (-0.67 to 0.83) | 0.19 (-0.62 to 1.01) | 0.07 (-0.68 to 0.82) | 0.40 (-0.40 to 1.21) |
| Delivery | Group | -0.61 (-1.63 to 0.40) | -0.46 (-1.52 to 0.60) | -1.02 (-3.43 to 1.31) | -0.68 (-1.75 to 0.38) | -0.49 (-1.44 to 0.47) | -0.49 (-1.47 to 0.50) | -0.49 (-1.43 to 0.46) | -0.29 (-1.27 to 0.73) |
| Delivery | Face to Face | -0.54 (-1.56 to 0.48) | -0.56 (-1.63 to 0.48) | -0.50 (-3.77 to 3.14) | -0.46 (-1.52 to 0.61) | -0.27 (-1.22 to 0.68) | -0.30 (-1.31 to 0.70) | -0.30 (-1.27 to 0.65) | -0.65 (-1.66 to 0.34) |
| Delivery | Telephone | -0.16 (-1.05 to 0.75) | -0.34 (-1.25 to 0.57) | -0.07 (-3.21 to 2.97) | -0.20 (-1.10 to 0.70) | 0.30 (-0.55 to 1.13) | 0.28 (-0.60 to 1.15) | 0.23 (-0.63 to 1.08) | 0.58 (-0.40 to 1.55) |
| Delivery | Internet | -0.14 (-1.34 to 1.08) | 0.12 (-1.07 to 1.33) | 0.45 (-4.89 to 5.83) | -0.06 (-1.27 to 1.15) | 0.06 (-1.04 to 1.19) | 0.13 (-1.04 to 1.29) | 0.01 (-1.09 to 1.14) | -0.10 (-1.39 to 1.19) |
| Delivery | App | 0.99 (-2.48 to 4.42) | 0.78 (-2.65 to 4.21) | -8.20 (-16.63 to 0.61) | 1.05 (-2.40 to 4.54) | 0.23 (-2.99 to 3.48) | 0.08 (-3.27 to 3.35) | 0.11 (-3.12 to 3.31) | 0.06 (-3.28 to 3.45) |
| Delivery | Print | -0.24 (-1.09 to 0.61) | -0.17 (-1.01 to 0.68) | -2.37 (-6.12 to 1.66) | -0.25 (-1.11 to 0.61) | -0.52 (-1.31 to 0.27) | -0.47 (-1.32 to 0.39) | -0.57 (-1.37 to 0.22) | -0.87 (-1.74 to -0.01) |
| Delivery | Video | 0.45 (-1.62 to 2.50) | 0.39 (-1.62 to 2.40) | 1.80 (-2.18 to 5.92) | 0.28 (-1.81 to 2.37) | 0.60 (-1.32 to 2.51) | 0.63 (-1.36 to 2.61) | 0.64 (-1.25 to 2.54) | 0.67 (-1.41 to 2.76) |
| Delivery | SMS | -0.80 (-4.08 to 2.47) | -1.03 (-4.21 to 2.21) | 9.81 (-1.72 to 20.9) | -0.93 (-4.25 to 2.37) | -0.56 (-3.66 to 2.53) | -0.68 (-3.82 to 2.46) | -0.51 (-3.64 to 2.58) | -0.05 (-3.73 to 3.58) |
| Delivery | Other/Unclear | 0.06 (-1.34 to 1.50) | 0.15 (-1.34 to 1.62) | -2.64 (-9.58 to 4.14) | -0.02 (-1.45 to 1.41) | 0.05 (-1.26 to 1.36) | 0.08 (-1.28 to 1.43) | 0 (-1.32 to 1.32) | -0.93 (-2.51 to 0.69) |
| Setting | Health Care | 0.51 (-0.65 to 1.66) | 0.34 (-0.80 to 1.50) | 0.95 (-3.54 to 5) | 0.51 (-0.65 to 1.66) | 0.43 (-0.65 to 1.51) | 0.40 (-0.71 to 1.51) | 0.47 (-0.59 to 1.55) | 0.85 (-0.31 to 2.02) |
| Setting | Community | 0.11 (-1.03 to 1.27) | -0.19 (-1.38 to 0.99) | -0.84 (-4.94 to 2.87) | 0.11 (-1.12 to 1.31) | -0.29 (-1.40 to 0.81) | -0.35 (-1.50 to 0.79) | -0.29 (-1.38 to 0.78) | 0.09 (-1.09 to 1.25) |
| Setting | Workplace | 0.37 (-3.59 to 4.28) | 0.27 (-3.63 to 4.10) | 0.19 (-6 to 6.09) | 0.30 (-3.66 to 4.18) | 0.24 (-3.52 to 3.98) | 0.32 (-3.52 to 4.08) | 0.40 (-3.31 to 4.18) | 0.51 (-3.17 to 4.23) |
| Setting | Home | -1.05 (-2.02 to -0.09) | -1.04 (-2.01 to -0.07) | -0.12 (-2.27 to 1.92) | -1.01 (-2 to -0.02) | -0.80 (-1.70 to 0.10) | -0.75 (-1.73 to 0.22) | -0.78 (-1.67 to 0.12) | -0.72 (-1.68 to 0.24) |
| Interaction | Diet*Exercise | - | - | - | -0.26 (-2.23 to 1.70) | - | - | - | - |
| Interaction | Diet*Nutritionist | - | - | - | 1.50 (-1.92 to 4.84) | - | - | - | - |
| Interaction | Diet*Dietitian | - | - | - | -1.12 (-3.51 to 1.23) | - | - | - | - |
| Interaction | Exercise*Exercise specialist | - | - | - | -2.81 (-5.75 to 0.13) | - | - | - | - |
| Interaction | Exercise*Health Trainer | - | - | - | 0.21 (-3.26 to 3.58) | - | - | - | - |
|  | | | | | | | | | |
|  | Covariate effect | - | - | - |  | 0.002 (-0.006 to 0.010) | 0.01 (-0.01 to 0.03) | 0.05 (-0.05 to 0.14) | 0.04 (-0.06 to 0.14) |
|  | Between studies SD | 2.09 (1.76 to 2.48) | 2.01 (1.66 to 2.43) | 1.65 (0.76 to 2.80) | 2.08 (1.74 to 2.47) | 1.88 (1.56 to 2.25) | 1.95 (1.62 to 2.35) | 1.87 (1.55 to 2.24) | 1.79 (1.43 to 2.21) |
|  | Deviance | 1173 (1122 to 1227) | 1105 (1057 to 1158) | 314.5 (288.7 to 345.4) | 1174 (1123 to 1229) | 1174 (1125 to 1229) | 1095 (1046 to 1148) | 1166 (1117 to 1221) | 952.9 (908.4 to 1004) |
|  | pD | 300.032 | 270.679 | 89.853 | 301.048 |  | 294.208 | 279.243 | 292.068 |
|  | DIC | 1473.32 | 1376.47 | 405.01 | 1475.47 |  | 1469.38 | 1374.49 | 1459.12 |

1. Terms not included in cells where results are not reported [↑](#footnote-ref-1)
